# Aryl diazonium intermediates enable mild DNA-compatible C–C bond formation for medicinally relevant combinatorial library synthesis†

**DOI:** 10.1039/d2sc04482j

**Published:** 2022-10-25

**Authors:** Xianfeng Li, Juan Zhang, Changyang Liu, Jie Sun, Yangfeng Li, Gong Zhang, Yizhou Li

**Affiliations:** Chongqing Key Laboratory of Natural Product Synthesis and Drug Research, School of Pharmaceutical Sciences, Chongqing University China yizhouli@cqu.edu.cn gongzhang@cqu.edu.cn; Chemical Biology Research Center, School of Pharmaceutical Sciences, Chongqing University 401331 Chongqing P. R. China; Key Laboratory of Biorheological Science and Technology, Ministry of Education, College of Bioengineering, Chongqing University P. R. China; Beijing National Laboratory for Molecular Sciences Beijing 100190 P. R. China

## Abstract

Forging carbon–carbon (C–C) linkage in DNA-encoded combinatorial library synthesis represents a fundamental task for drug discovery, especially with broad substrate scope and exquisite functional group tolerance. Here we reported the palladium-catalyzed Suzuki–Miyaura, Heck and Hiyama type cross-coupling *via* DNA-conjugated aryl diazonium intermediates for DNA-encoded chemical library (DEL) synthesis. Starting from commodity arylamines, this synthetic route facilely delivers vast chemical diversity at a mild temperature and pH, thus circumventing damage to fragile functional groups. Given its orthogonality with traditional aryl halide-based cross-coupling, the aryl diazonium-centered strategy expands the compatible synthesis of complex C–C bond-connected scaffolds. In addition, DNA-tethered pharmaceutical compounds (*e.g.*, HDAC inhibitor) are constructed without decomposition of susceptible bioactive warheads (*e.g.*, hydroxamic acid), emphasizing the superiority of the aryl diazonium-based approach. Together with the convenient transformation into an aryl azide photo-crosslinker, aryl diazonium's DNA-compatible diversification synergistically demonstrated its competence to create medicinally relevant combinatorial libraries and investigate protein–ligand interactions in pharmaceutical research.

## Abstract 2

Taking advantage of aryl diazonium intermediates, this work reported a DNA-compatible C–C bond formation strategy, achieving broad substrate scope, exquisite functional group tolerance, and orthogonality to aryl halide-based coupling reactions.

## Graphical abstract

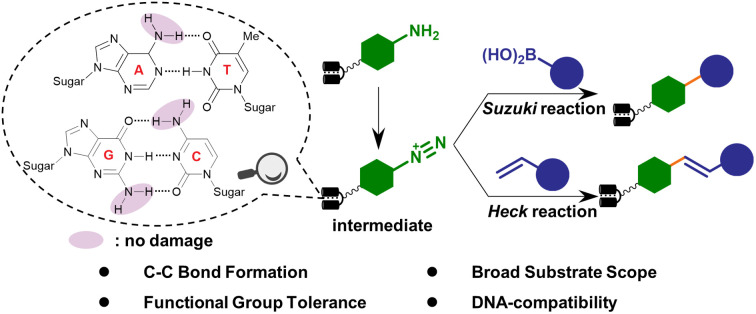

## Introduction

Carbon–carbon (C–C) bond formation is a fundamental process in all facets of organic and bioorganic chemistry, in which palladium (Pd)-catalyzed cross-coupling reactions are integral.^1–4^ These palladium-catalyzed cross-coupling reactions mainly focus on building C(sp^2^)–C(sp^2^) connections that feature structural rigidity and increase drug-likeness. The importance of C(sp^2^)–C(sp^2^) bonds, together with the broad substrate of Pd-catalyzed cross-coupling, render Suzuki–Miyaura, Heck and Hiyama reactions the most widely adopted synthetic approaches in medicinal chemistry.^5,6^ Meanwhile, metal-catalyzed C–C bond formation has emerged as an important topic in chemical biology, as evidenced by the adaptation of Pd-catalyzed cross-coupling for chemoselective modification of biomacromolecules. For example, iodo/bromo-functionalized unnatural bases allowed fluorogenic labeling of DNA/RNA molecules;^7–10^ proteins bearing a site-specifically incorporated iodophenyl handle could undergo bioorthogonal labeling with boronic acid-conjugated probes.^11,12^ Consequently, expanding the scope of Pd-catalyzed C–C coupling reactions, especially in a biocompatible manner, is of great significance to medicinal chemistry and chemical biology.

From the chemistry–biology interface, a drug interrogation technique named DNA-encoded chemical library (DEL) has emerged. Since its sprouting concept was proposed by Lerner and Brenner in 1992, three decades of efforts have proved the DEL to be an economical and efficient platform to expedite bioactive hit discovery.^13–17^ Compared with the resource-intensive high-throughput screening (HTS) that contains separately synthesized and deposited library members, a DEL allows the miniature-scale synthesis, maintenance, and selection of all library members simultaneously in a single pool. Typically, each diverse building block (BB) is conjugated to the starting DNA fragment, accompanied by the enzymatic ligation of a corresponding DNA barcode. In this fashion, the molecular identity of each chemical subunit incorporated is recorded by using the tethered DNA sequence. By integrating the power of combinatorial synthesis with genetic barcoding in iterative “split-and-pool” cycles, libraries comprising an exceptional magnitude (10^6^–10^12^) of members could be achieved. When the library is selected against the target protein, the molecular identity of positive binders is then interpreted *via* PCR and sequencing. Attracted by the DEL's cost and time efficiency, both academia and pharmaceutical companies have advanced DEL in terms of synthetic methodology and selection strategy.^18–31^ Currently, although very few drug candidates in late-stage clinical trials stemmed from DEL, such as the soluble epoxide hydrolase (sEH) inhibitor GSK2256294 and the receptor-interacting protein (RIP1) kinase inhibitor GSK2982772,^32–35^ DELs have shown their potential in high-throughput discovery of preliminary bioactive hits for defined targets, which might lead to the development of bioactive compounds or probes with structural optimization.

Successful hit discovery lies in the DEL's vast chemical space and abundant structural diversity, which in turn depends on the DEL synthetic reactions employed. First, DEL-compatible reactions should be performed in aqueous solutions and circumvent inherent restrictions posed by DNA's physicochemical properties. Second, DEL synthesis should either create meaningful drug-like structures such as (hetero)aromatic cycles and natural products,^36–39^ or link pharmacophore-containing building blocks with chemically stable and structurally rigid scaffolds, rather than merely build loose and flexible connections between DNA-tagged chemical subunits. Notably, C(sp^2^)–C(sp^2^) bond formation is a major approach to assembling and decorating aromatic BBs with structurally confined and less rotatable linkage. Therefore, forging C(sp^2^)–C(sp^2^) linkage in DEL synthesis is a meaningful task, especially under mild conditions, and having broad substrate scope, and remarkable functional group tolerance.

**Fig. 1:**
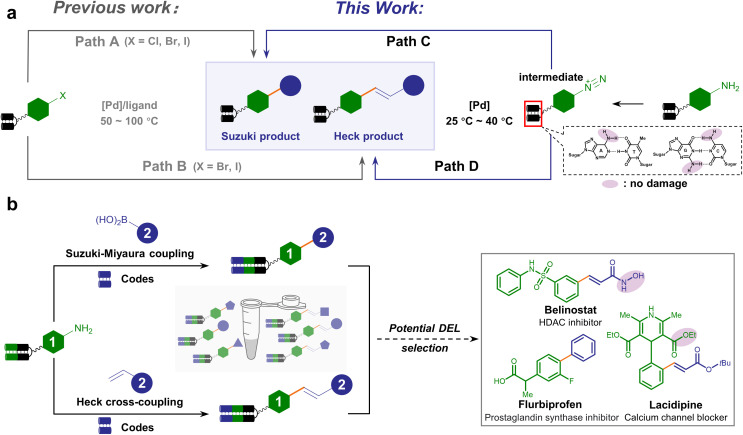
Scheme of the DNA-encoded C–C cross-coupling reaction based on aryl diazonium intermediates. (a) Comparison between divergent on-DNA synthetic routes of Pd-catalyzed cross-coupling, either starting from aryl halides (path A and B, previous work) or aryl diazonium salts (path C and D, this work). (b) The proposed workflow to synthesize medicinally relevant C–C conjugated DELs uniformly starting from arylamines. Typical bioactive compounds containing essential C–C linkages are listed.

Previous efforts have generated a few DNA-compatible Pd-catalyzed C–C cross-coupling reactions.^40–55^ A suite of synthetic methodologies realized robust Suzuki–Miyaura coupling between a diverse set of DNA-tethered aryl halides (X = I, Br, or Cl) and boronic acids/esters.^40–45,50–53,55^ Besides, efficient Pd-catalyzed DNA-compatible Heck coupling reactions between olefins and aryl halides were exploited.^46,47^ However, most approaches generally employ harsh conditions such as elevated temperatures (>50 °C) and strong bases, which might cause DNA and functional group damage (Fig. 1a, path A and B). Recently, aryl fluorosulfonate electrophiles were explored for Suzuki–Miyaura coupling under milder conditions, thus having better functional group tolerance, expanding the substrate scope to a panel of DNA-tagged phenols.^48^ Alternative strategies could afford C(sp^2^) linkage beyond Pd catalysis, such as harnessing the power of photochemistry.^56–61^ Nevertheless, concise, versatile and mild-condition approaches to synthesizing C–C connections from abundant and commercially available BBs are still in urgent demand.

Compared with aryl halides, aryl diazonium salts serve as versatile reagents for transition metal-catalyzed cross-coupling.^62–64^ The higher reactivity of aryl diazonium permits the Suzuki–Miyaura or Heck cross-coupling reaction to occur at a lower temperature, under milder pH conditions, free of additional ligands, and in an environment-friendly fashion.^65,66^ In addition, aryl diazonium salts can be readily prepared from abundant and inexpensive arylamines preferred in combinatorial chemistry.^17^ As a consequence, we envision that the on-DNA aryl diazonium-based cross-coupling offers a complementary or even superior approach to forging C–C linkage (Fig. 1a, paths C and D), which could facilitate DELs to expedite bioactive compound discovery. Typical examples containing C–C bonds include flurbiprofen, belinostat, and lacidipine, which contain hydrolyzable esters or hydroxamic acids (Fig. 1b).

## Results and discussion

**Fig. 2:**
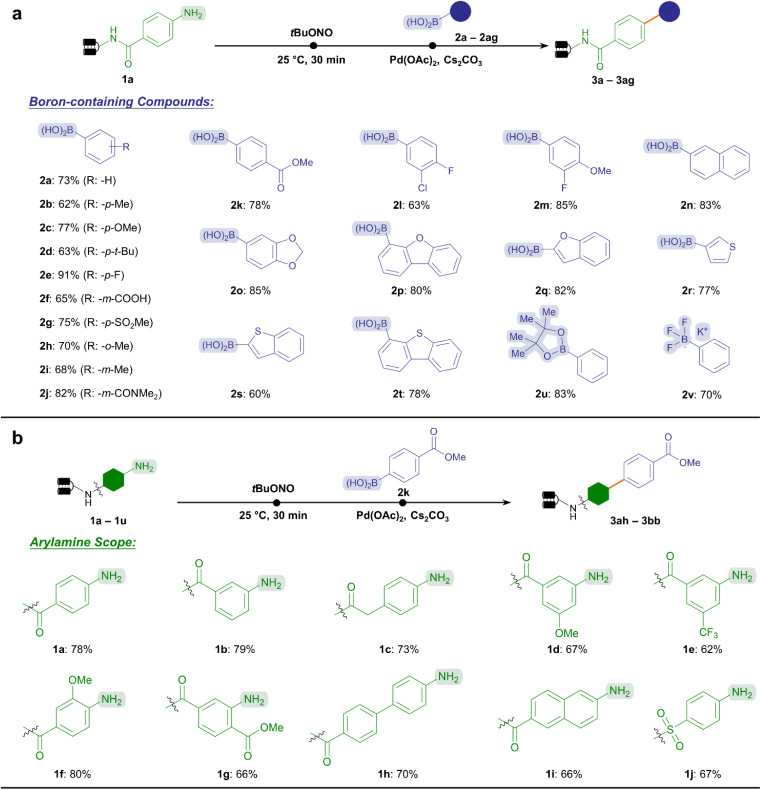
Substrate scope of on-DNA Suzuki–Miyaura coupling *via* aryl diazonium intermediates. (a) Representative boron-containing compounds were cross-coupled with the standard DNA–aniline conjugate 1a. (b) Various DNA-conjugated arylamines were examined with aryl boronic acid 2k. Reactive handles were highlighted. Reaction conditions: step 1, DNA-conjugated arylamine (0.2 nmol, 8 μL, 25 μM in H_2_O), *t*BuONO (100 nmol, 2 μL, 50 mM in DMA), and H_2_O (10 μL), 25 °C for 0.5 h; step 2, further add Pd(OAc)_2_ (40 nmol, 2 μL, 20 mM in DMA), boronic acid (1000 nmol, 2 μL, 500 mM in DMA), Cs_2_CO_3_ (1600 nmol, 2 μL, 800 mM in H_2_O), and H_2_O up to a total volume of 30 μL, 40 °C for 2 h.

**Fig. 3:**
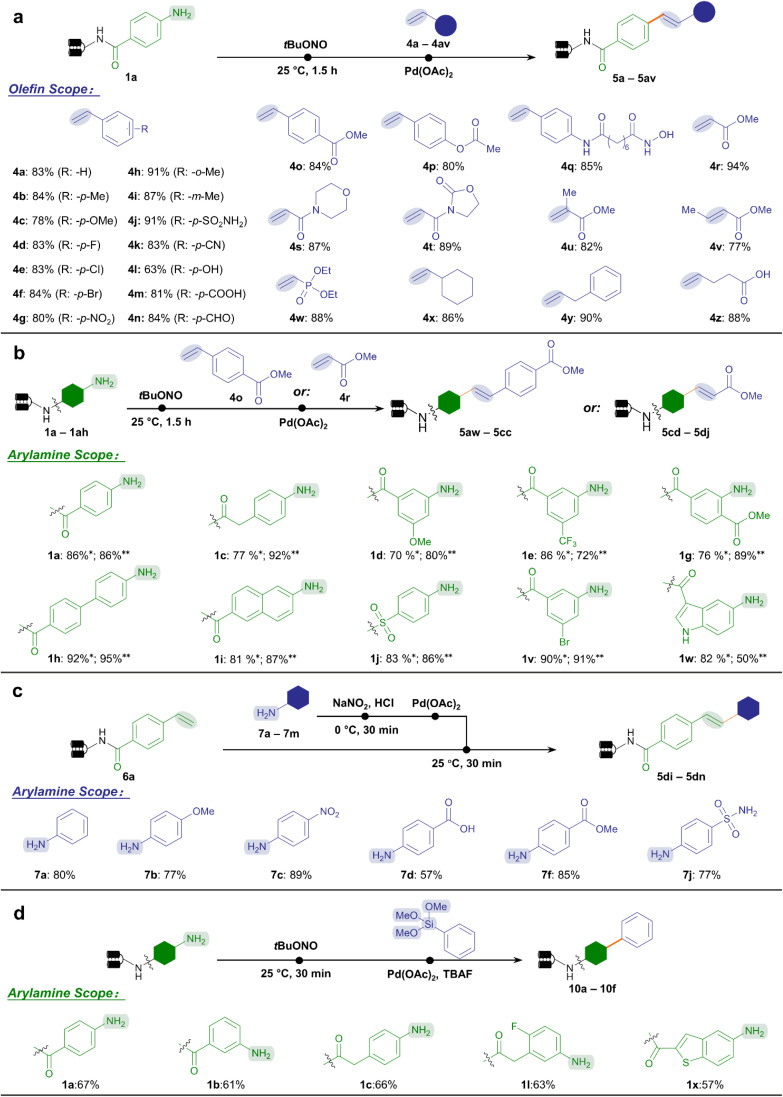
Substrate scope of the on-DNA aryl diazonium-based Heck and Hiyama reactions. (a) A diverse range of olefins were coupled to the DNA–aniline conjugate 1a. (b) Starting from different DNA-conjugated arylamines, Heck reactions with styrene 4o (yield with *) and methyl acrylate 4r (yield with **) were investigated. (c) Reverse on-DNA Heck reaction allowed the coupling of on-DNA styrene 6a with a panel of arylamines. Reactive handles are highlighted. (d) Substrate scope of the Hiyama reaction. Several DNA-conjugated arylamines were tested. Reaction conditions in (a) and (b): step 1, DNA-conjugated arylamine (0.2 nmol, 8 μL, 25 μM in H_2_O), *t*BuONO (100 nmol, 2 μL, 50 mM in DMA), and H_2_O (10 μL), 25 °C for 1.5 h; step 2, further add Pd(OAc)_2_ (40 nmol, 2 μL, 20 mM in DMA), phosphate buffer (3 μL, 250 mM in H_2_O, pH = 5.5), olefin (1000 nmol, 2 μL, 500 mM in DMA), and H_2_O up to a total volume of 30 μL, 25 °C for 0.5 h. Reaction conditions in (c): step 1, arylamine (100 nmol, 2 μL, 50 mM in DMA), HCl (200 nmol, 2 μL, 100 mM in H_2_O), NaNO_2_ (100 nmol, 2 μL, 50 mM in H_2_O), 0 °C for 0.5 h; step 2, further add Pd(Oac)_2_ (100 nmol, 2 μL, 50 mM in DMA), phosphate buffer (6 μL, 250 mM in H_2_O, pH = 5.5), DNA-conjugated styrene (0.2 nmol, 4 μL, 50 μM in H_2_O), and H_2_O up to a total volume of 30 μL, 25 °C for 0.5 h. Reaction conditions in (d): step 1, DNA-conjugated arylamine (0.2 nmol, 8 μL, 25 μM in H_2_O), *t*BuONO (100 nmol, 2 μL, 50 mM in DMA), and H_2_O (10 μL), 25 °C for 0.5 h; step 2, further add Pd(OAc)_2_ (40 nmol, 2 μL, 20 mM in DMA), phosphate buffer (6 μL, 250 mM in H_2_O, pH = 5.5), TBAF (100 nmol, 2 μL, 50 mM in H_2_O), phenyltrimethoxysilane (1000 nmol, 2 μL, 500 mM in DMA), and H_2_O up to a total volume of 100 μL, 40 °C for 1 h.

We set out to establish a standard condition for DNA-compatible diazotization and cross-coupling starting from the aniline-conjugated DNA headpiece (HP) 1a. Although aryl diazonium could be readily generated *in situ* by treating arylamine with nitrous acid,^67^ nitrous acid (HNO_2_) and nitrite (NO_2_^−^) at high concentrations are notoriously known as carcinogens. While they are transformed into *N*-nitrosamine derivatives in the liver as a strong DNA-alkylating species, they can also directly cause hydrolytic deamination of nucleobases through diazonium intermediates *in vitro*, leading to mutations of adenine, guanine and cytosine.^68,69^ Therefore, the diazotization reagent's preferred reactivity to the DNA-tethered aniline group rather than to native nucleobases is obligatory. Due to the instability of aryl diazonium salts in LC-MS detection, diazotization was monitored after further nucleophilic substitution to afford phenyl azide 1a′. We compared two diazotization reagents, nitrous acid (NaNO_2_/HCl) and *tert*-butyl nitrite (*t*BuONO). While prolonged NaNO_2_/HCl treatment caused partial nucleobase deamination, good to excellent conversion was observed without detectable DNA damage by *t*BuONO treatment (final conc. 5 mM, 25 °C), indicating that *t*BuONO was a DNA-compatible diazotization reagent (ESI Table 1 and Fig. 1–3†). Following the aryl azide transformation of several DNA-conjugated arylamines, subsequent copper-catalyzed azide–alkyne cycloaddition (CuAAC) reactions were performed with phenylacetylene to form 1,2,3-triazoles (ESI Fig. 4†). This diversification demonstrated that arylamine building blocks could serve as masked precursors to generate active aryl diazonium intermediates, and further showed potential to synthesize DELs with click chemistry from diverse aryl azides facilely generated from arylamines.

Following the consideration of diazotization conditions, we next investigated the subsequent Suzuki–Miyaura coupling from aryl diazonium intermediates. When palladium(ii) acetate and phenylboronic acid 2a were sequentially added to the aryl diazonium intermediate, the biphenyl coupling product 3a was observed with 60% conversion (Table 1, entry 1). To reduce deamination/hydrolysis of diazonium intermediates and increase the efficiency of the coupling reaction, the stability of aryl diazoniums and the reactivity of boronic acids required elegant balancing. In detail, in the first step, a weak acidic environment enhanced the stability of the diazonium intermediate, consistent with the fact that diazonium was prone to hydrolysis under basic conditions. In the second step, we examined different bases to activate phenylboronic acid, and found that cesium carbonate (Cs_2_CO_3_) yielded better conversion (Table 1, entries 2–6 and ESI Table 2†). A panel of Pd catalysts displayed varying performances, showing that palladium(ii) acetate was the best (Table 1, entries 7–9 and ESI Table 2†). To further improve conversion, different temperatures were investigated, and 40 °C was found to be optimal (Table 1, entries 10–12 and ESI Table 2†). Therefore, the optimized condition reconciled reactivity with stability by fine-tuning the pH and temperature, providing 73% conversion (Table 1, entry 10). To verify the product's absolute structure, we conjugated the off-DNA synthesized and characterized small molecule 4-phenyl-benzoic acid S3a to HP-DNA as the standard. Identical retention times were observed in co-injection assays between the on-DNA generated product and off-DNA prepared standard (ESI Fig. 5†). Altogether, our optimized approach allowed the on-DNA architecture of the C–C bond *via* aryl diazonium-based Suzuki–Miyaura coupling (Fig. 1b).

**Table 1:** Optimization of on-DNA Suzuki–Miyaura coupling *via* aryl diazonium intermediates^a^

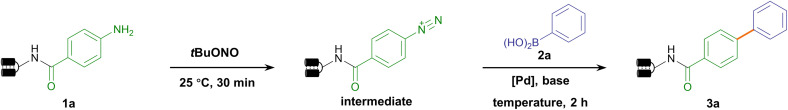				
Entry	Catalyst	Base	Temperature (°C)	Conversion (%)
1	Pd(OAc)_2_	—	25	60
2	Pd(OAc)_2_	NaOH	25	64
3	Pd(OAc)_2_	Cs_2_CO_3_	25	68
4	Pd(OAc)_2_	DIPEA	25	46
5	Pd(OAc)_2_	Et_3_N	25	62
6	Pd(OAc)_2_	NaOAc	25	60
7	PdCl_2_(COD)	Cs_2_CO_3_	25	<10
8	sSPhos-Pd-G2	Cs_2_CO_3_	25	<10
9	PdCl_2_	Cs_2_CO_3_	25	54
**10**	**Pd(OAc)**_**2**_	**Cs**_**2**_ **CO**_**3**_	**40**	**73**
11	Pd(OAc)_2_	Cs_2_CO_3_	60	68
12	Pd(OAc)_2_	Cs_2_CO_3_	80	67

a Conditions for Suzuki–Miyaura coupling. Step 1: mix 1a (0.2 nmol, 8 μL, 25 μM in H_2_O), *t*BuONO (100 nmol, 2 μL, 50 mM in DMA), and H_2_O (10 μL), 25 °C for 0.5 h; Step 2: further add catalyst (40 nmol, 2 μL, 20 mM in DMA), boronic acid (1000 nmol, 2 μL, 500 mM in DMA), base (1600 nmol, 2 μL, 800 mM in H_2_O), and H_2_O up to a total volume of 30 μL, indicated temperature for 2 h.

In this vein, we inspected the substrate scope of Suzuki–Miyaura coupling by testing a wide variety of boronic acids or boron-containing chemical subunits according to the established protocol (Fig. 2a). To our delight, this reaction exhibited good conversion (60–91%) towards a broad range of electron-rich and electron-deficient substituted phenylboronic acids (2b–2k), including multi-substituted substrates (2l and 2m). Notably, labile functional groups such as the ester (2k) were well-tolerated, together with reactive handles permitting further diversification such as carboxylic acid (2f). In addition, heterocyclic and polycyclic boronic acids yielded cross-coupling products with good to excellent conversion (60–85%, 2n–2t), offering an opportunity to assemble pharmaceutically privileged aromatic scaffolds (*e.g.*, benzofuran and thiophene) by usin C(sp^2^)–C(sp^2^) linkage. Furthermore, the reactivity of boronic ester (2u) and potassium trifluoroborate salt (2v) was demonstrated. This significantly expanded the chemical space of the aryl diazonium-based Suzuki–Miyaura C–C coupling reaction by accommodating available suites of boron-containing compounds in combinatorial synthesis. Altogether, 20 boronic-containing subunits provided good to excellent conversions (>70%), while 13 resulted in acceptable conversions (50–70%) among the 33 BBs tested (ESI Table 3†).

Besides, we explored the substrate scope regarding the DNA-conjugated arylamines (Fig. 2b). The ester bond-containing 2k was chosen as the standard substrate. As expected, a variety of substituted anilines, whether with electron-rich or electron-withdrawing substituents provided good conversion rates without ester hydrolysis (62–80%, 1a–1g).

In addition, other DNA-tethered arylamines containing naphthalene, biphenyl, or sulfonamide groups yielded satisfactory conversions (66–70%, 1h–1j). Supported by the abundant commercial availability of bifunctional amine BBs^17^ and 22 representative substrates tested here (ESI Table 4†), we could anticipate that a wide range of DNA-conjugated arylamines would increase the diversity of synthesized libraries.

Encouraged by the accomplishment of the aryl diazonium-based Suzuki–Miyaura coupling reaction which proceeded under mild conditions, we accordingly managed to adapt analogous conditions for the Heck coupling reaction between aryl diazonium intermediates and olefins. To our delight, in the presence of palladium(ii) acetate and phosphate buffer (pH 5.5), the Heck coupling reaction with styrene 4a yielded stilbene product 5a with good efficiency (83%) at 25 °C (Fig. 3a). Similarly, the on-DNA Heck coupling product's structure was validated by off-DNA synthesis and co-injection experiments (ESI Fig. 6†). Therefore, we demonstrated the capability to forge C–C bonds starting from arylamine BBs and olefins in DEL synthesis under exceptionally mild conditions (Fig. 1b).

Next, we comprehensively studied the chemical space of two categories of substrates, namely styrenes and α,β-unsaturated carbonyl species (Fig. 3a). Gratifyingly, for styrene-type substrates, various substituents on styrene were allowed with excellent conversion to stilbenes (63–91%, 4a–4q), including the halide (4d–4f), nitro group (4g), cyano group (4k), hydroxyl group (4l), carboxylic acid (4m), and aldehyde (4n), leaving ample space for late-stage modifications. Notably, bioactive functional moieties such as benzenesulfonamide (4j) reacted efficiently, while esters (4o–4p) and hydroxamic acid (4q) were well-tolerated without hydrolysis or decomposition. Meanwhile, most of the acrylate ester and acrylamide substrates achieved excellent efficiency (77–94%, 4r–4v) to afford cinnamic acid derivatives, an important class of natural products and bioactive compounds. Again, no hydrolysis byproducts were observed for esters (4r, 4u, 4v) and oxazolidone (4t). This highly desired property allowed the incorporation of ester-containing BBs, which would otherwise hydrolyze under the conventional Heck coupling condition that employed strong bases and high temperatures.^47^ Other categories of olefin substrates also demonstrated excellent conversion (4w–4z). Altogether, 43 out of the 48 olefin BBs tested achieved good conversion (>70%, ESI Table 5†).

Subsequently, we performed the DNA-conjugated arylamine scope study. We tested the conversion of a series of arylamines reacting with ester bond-containing styrene 4o and methyl acrylate 4r, respectively (Fig. 3b). Similarly with the arylamines' scope for Suzuki–Miyaura coupling, among the 34 DNA-tagged substrates tested, 29 substrates achieved good conversions (>70%) with styrene (ESI Table 6†), while 31 afforded good conversions with methyl acrylate (ESI Table 7†). As a complement to the Heck reaction above, we took advantage of the abundant arylamine BBs to reversely investigate their reactivity profiles with the DNA-conjugated styrene 6a (Fig. 3c). According to our previous work,^27^ a panel of arylamines (7a–7m) was treated with NaNO_2_/HCl to readily form aryl diazonium salts *in situ*. The mixture reacted smoothly with 6a to afford the expected products with high conversion without detectable damage (ESI Table 8†), no matter with electron-rich (7b), electron-deficient (7c), or reactive (7d, 7f) substituents. Altogether, Heck coupling between aryl diazoniums and olefins was achieved regardless of which end was in conjugation with DNA. This offered various methods to generate cinnamic acid-derived and stilbene-like chemical scaffolds in DEL synthesis.

Besides the widely explored Suzuki–Miyaura and Heck reactions, we were interested in exploring more C–C bond coupling reaction types enabled by the aryl diazonium intermediates. Considering the possible Pd-catalyzed Hiyama reaction between aryl diazonium salts and organosilanes,^70,71^ we tested and optimized the Hiyama reaction condition on DNA-conjugated aryl diazonium intermediates. A panel of DNA-conjugated arylamines reacted with phenyltrimethoxysilane to afford C(sp^2^)–C(sp^2^)-conjugated products in moderate conversion (51–67%, Fig. 3d, ESI Table 9 and Fig. 7†). To our knowledge, this represented the first-case exploration of Hiyama reaction on DNA, offering an alternative and supplementary approach to architect C(sp^2^)–C(sp^2^) linkages in DELs using organosilicon compounds. We also tested the on-DNA reactions on a large scale exemplified by Suzuki–Miyaura and Heck reactions, demonstrating their applicability in industrialized DEL construction (ESI Fig. 8 and 9†).

**Fig. 4:**
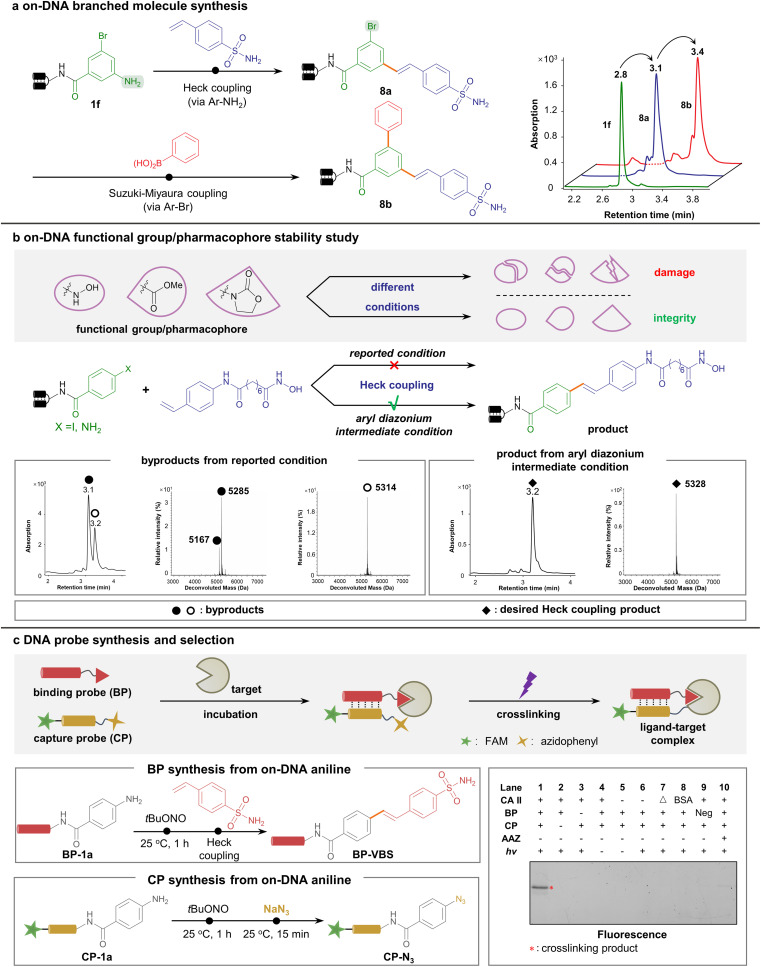
Aryl diazonium intermediates facilitated multi-dimensional C–C-assembled DEL construction and a DNA-conjugated bioactive chemical probe study. (a) Synthesis of the branched C–C-linked scaffold *via* orthogonal aryl diazonium-based and aryl halide-based cross-coupling reactions. (b) Aryl diazonium-based Heck reaction enabled the installation of fragile functional groups. The parallel synthesis of hydroxamic acid onto DNA to generate the HDAC inhibitor indicated no hydrolysis in our aryl diazonium-based approach. (c) DNA-conjugated chemical probe synthesis and subsequent covalent crosslinking/selection by DNA-programmed affinity labeling. Top: BP-VBS, CP-N_3_, and target protein CA II were incubated and photo-crosslinked, and subjected to gel analysis. Left: BP-VBS and CP-N_3_ were both obtained from DNA-tethered aniline starting materials *via* Pd-catalyzed Heck reaction or nucleophilic substitution, respectively. Right: fluorescence imaging results of the assay. The crosslinked bands indicated the presence of both bioactive sulfonamide in BP-VBS and phenyl azide in CP-N_3_. Δ: denatured CA II, BSA: bovine serum albumin, Neg: BP without a ligand.

To demonstrate the wide applicability of the new methodology, diversification to multi-dimensional C–C-assembled DELs was performed. Considering the different conditions of aryl diazonium-based and aryl halide-based coupling approaches, we predicted them to be orthogonal. To illustrate this viewpoint, we started with a 3-bromo-5-aminobenzoic acid-conjugated DNA headpiece 1f, a bifunctional starting DNA bearing both aryl bromide and arylamine handles. As expected, 1f underwent the first round of the Heck coupling reaction on the arylamine handle (*via* aryl diazonium intermediate) to afford 8a with high efficiency, leaving aryl bromide intact. Next, the aryl bromide further underwent the second step Suzuki–Miyaura coupling reaction, generating branched C–C-connected scaffold 8b (Fig. 4a). Likewise, a linear topological scaffold was constructed. Bromo-substituted styrene was tolerated to react with the DNA-linked aryl diazonium, followed by a second-round Suzuki–Miyaura coupling *via* aryl bromide (ESI Fig. 10†). These data together proved the capability to construct various C–C-assembled scaffolds in a DEL *via* orthogonal Pd-catalyzed cross-coupling.

Furthermore, the compatibility of this suite of Pd-catalyzed reactions with DEL synthesis was carefully examined. First, enzymatic DNA ligation was assessed to rule out any possible impairment by metal ions or nitrous species. Starting from the uniformed DNA-conjugated aniline HP-P-1a, different reaction conditions reported above (including Suzuki–Miyaura and Heck cross-coupling reactions) were applied, and then the ligation assay was performed on the reaction product subsequently (ESI Fig. 11†). To our delight, all sets of enzymatic DNA barcode ligation proceeded smoothly, as shown by the polyacrylamide gel electrophoresis (PAGE) analysis (ESI Fig. 12†). In addition, we investigated the compatibility with the decoding process by preparing a longer aniline-conjugated DNA named PCR-1 resembling the length of a fully encoded DEL (ESI Fig. 13 and 14†). PCR-1 was subjected to various reaction conditions (including azide transformation, Suzuki–Miyaura coupling, and the Heck coupling reaction), and the resulted products, as PCR templates, underwent PCR amplification and Sanger sequencing (ESI Fig. 15†). If obvious DNA damage occurred, either the PCR process would be hampered, or the sequencing results would lose fidelity. Nevertheless, no detectable mutation or increased sequencing noise was observed (ESI Fig. 16†), indicating that DNA was tolerable in these combinatorial synthetic procedures. With these data, we inferred that the construction of multi-dimensional DNA-encoded C–C-assembled combinatorial libraries *via* diazonium intermediates was conceivable.

With the potential to construct multi-dimensional DELs, we further sought to strengthen the utility by demonstrating the efficient synthesis of medicinally relevant and biologically active compounds on DNA. Since the condition of the aryl diazonium-based Heck coupling reaction was extremely mild, we envisioned that various bioactive warheads would remain intact *via* the aryl diazonium-based synthesis.

Given the evidence that susceptible functional groups such as esters and oxazolidones were elegantly preserved in our synthetic strategy (see Fig. 2 and 3), we launched a comparative study to incorporate a variety of functional groups into DNA conjugates (Fig. 4b, ESI Fig. 17–20†). Notably, the HDAC inhibitor bearing an easily hydrolysable hydroxamic acid warhead was on-DNA synthesized *via* our aryl diazonium-based approach in parallel with the traditional aryl halide-based approach. Starting from the arylamine- or aryl iodide-conjugated DNA, a HDAC ligand unit containing a styrene moiety was cross-coupled in parallel (see the ESI† for detailed methods). As shown by the LC-MS data, the high temperature and strong base required for the aryl iodide-based reaction caused almost complete hydrolysis of the hydroxamic acid warhead. In contrast, the ambient temperature and mild pH conditions in the aryl diazonium group yielded the desired product, leaving the hydroxamic acid warhead intact (Fig. 4b). These data emphasized our approach's excellent functional group tolerance.

In addition, aryl diazonium could be readily transformed into aryl azide (as shown in Table 1), a canonical photo-crosslinking probe. To prove its utility, we chose the model system of sulfonamide binding to the carbonic anhydrase CA II to perform the DNA-programmed photoaffinity labeling (DPAL) assay^72,73^ that permits facile identification of target-binding ligands (Fig. 4c). Starting from the versatile DNA-conjugated aniline, divergent functionalization was implemented. A binding probe (BP) bearing an aniline handle was conjugated with 4-vinyl benzenesulfonamide to form BP-VBS, while a capture probe (CP) bearing an aniline moiety was transformed into the phenyl azide-containing CP-N_3_ with photo-crosslinking properties. Upon the formation of a ternary complex among BP-CBS, CP-N_3_, and CA II, proximity-enabled covalent crosslinking occurred upon UV irradiation, suggesting the robust binding of the sulfonamide ligand and efficient crosslinking of the phenyl azide warhead (Fig. 4c). Altogether, these experiments indicated that the on-DNA aryl diazonium-centered synthesis was a versatile platform to afford various pharmaceutically active compounds and valuable chemical probes.

## Conclusion

In conclusion, we have implemented the DNA-compatible Suzuki–Miyaura, Heck and Hiyama coupling reactions that employ aryl diazonium salts as the intermediates, enabling the facile architecture of C–C linkage in medicinally relevant DEL synthesis under mild condition, and having extensive substrate scope and exceptional functional group tolerance. Starting from DNA-tagged arylamines, we examined the diazotization condition to realize chemo-selective modification of the arylamine in preference to native nucleotides. We fine-tuned the buffer to realize efficient Suzuki–Miyaura, Heck and Hiyama cross-coupling across a diverse suite of chemical subunits, and then investigated the substrate scope of arylamines, boronic acids/esters, and olefins. Moreover, the diazonium-based approach adopted mild conditions and proved orthogonal with traditional aryl halide groups, providing an avenue to assemble branched or linear multi-dimensional drug-like libraries with structurally confined C–C connections. Remarkably, the aryl diazonium-based Heck coupling reaction was carried out under ambient room temperature and mild pH conditions, allowing the on-DNA synthesis of pharmaceutical compounds bearing susceptible bioactive warheads that would otherwise decompose (*e.g.*, HDAC inhibitor with the hydroxamic acid group). In addition, aryl diazonium could be readily transformed into aryl azide as a covalent biomacromolecule photo-crosslinker. These altogether demonstrated the versatility of aryl diazonium to create medicinally relevant combinatorial libraries and the potential of resulting DNA probes to investigate ligand–protein interaction under physiological conditions. Finally, the DNA-compatible aryl diazonium-based synthetic strategies developed here may allow a broader panel of diversity-oriented transformations, providing potential solutions to medicinal chemistry and chemical biology in the future.

## Author contributions

X. L. and J. Z. performed key experiments and analyzed the data. C. L., J. S., and Y.-F. L. helped with the experiments and data analysis. Y.-Z. L. and G. Z. conceived the study and wrote the manuscript. All authors discussed the results and gave suggestions during manuscript preparation.

## Conflicts of interest

The authors declare that this technology is covered by a patent application (CN112851733A).

## Supplementary Material

SC-013-D2SC04482J-s001

## References

[cit1] Seechurn C. C. J., Kitching M. O., Colacot T. J., Snieckus V. (2012). Angew. Chem., Int. Ed..

[cit2] Beletskaya I. P., Cheprakov A. V. (2000). Chem. Rev..

[cit3] Spicer C. D., Davis B. G. (2014). Nat. Commun..

[cit4] Schneider N., Lowe D. M., Sayle R. A., Tarselli M. A., Landrum G. A. (2016). J. Med. Chem..

[cit5] Roughley S. D., Jordan A. M. (2011). J. Med. Chem..

[cit6] Brown D. G., Bostrom J. (2016). J. Med. Chem..

[cit7] Omumi A., Beach D. G., Baker M., Gabryelski W., Manderville R. A. (2011). J. Am. Chem. Soc..

[cit8] Lercher L., McGouran J. F., Kessler B. M., Schofield C. J., Davis B. G. (2013). Angew. Chem., Int. Ed..

[cit9] Shaughnessy K. H. (2015). Molecules.

[cit10] Walunj M. B., Tanpure A. A., Srivatsan S. G. (2018). Nucleic Acids Res..

[cit11] Spicer C. D., Triemer T., Davis B. G. (2012). J. Am. Chem. Soc..

[cit12] Gao Z., Gouverneur V. r., Davis B. G. (2013). J. Am. Chem. Soc..

[cit13] Brenner S., Lerner R. A. (1992). Proc. Natl. Acad. Sci. U. S. A..

[cit14] Zhao G., Huang Y., Zhou Y., Li Y., Li X. (2019). Expert Opin. Drug Discovery.

[cit15] Goodnow R. A., Dumelin C. E., Keefe A. D. (2017). Nat. Rev. Drug Discovery.

[cit16] Franzini R. M., Randolph C. (2016). J. Med. Chem..

[cit17] Zabolotna Y., Volochnyuk D. M., Ryabukhin S. V., Horvath D., Gavrilenko K. S., Marcou G., Moroz Y. S., Oksiuta O., Varnek A. (2022). J. Chem. Inf. Model..

[cit18] Huang Y., Meng L., Nie Q., Zhou Y., Chen L., Yang S., Fung Y. M. E., Li X., Huang C., Cao Y., Li Y., Li X. (2021). Nat. Chem..

[cit19] Li Y., De Luca R., Cazzamalli S., Pretto F., Bajic D., Scheuermann J., Neri D. (2018). Nat. Chem..

[cit20] Favalli N., Bassi G., Pellegrino C., Millul J., De Luca R., Cazzamalli S., Yang S., Trenner A., Mozaffari N. L., Myburgh R. (2021). Nat. Chem..

[cit21] Clark M. A., Acharya R. A., Arico-Muendel C. C., Belyanskaya S. L., Benjamin D. R., Carlson N. R., Centrella P. A., Chiu C. H., Creaser S. P., Cuozzo J. W. (2009). Nat. Chem. Biol..

[cit22] Vummidi B. R., Farrera-Soler L., Daguer J.-P., Dockerill M., Barluenga S., Winssinger N. (2022). Nat. Chem..

[cit23] Wichert M., Krall N., Decurtins W., Franzini R. M., Pretto F., Schneider P., Neri D., Scheuermann J. (2015). Nat. Chem..

[cit24] Machutta C. A., Kollmann C. S., Lind K. E., Bai X., Chan P. F., Huang J., Ballell L., Belyanskaya S., Besra G. S., Barros-Aguirre D. (2017). Nat. Commun..

[cit25] Reddavide F. V., Lin W., Lehnert S., Zhang Y. (2015). Angew. Chem., Int. Ed..

[cit26] Yang S., Zhao G., Gao Y., Sun Y., Zhang G., Fan X., Li Y., Li Y. (2022). Chem. Sci..

[cit27] Zhang J., Li X., Wei H., Li Y., Zhang G., Li Y. (2021). Org. Lett..

[cit28] Zhong S., Fang X., Wang Y., Zhang G., Li Y., Li Y. (2022). Org. Lett..

[cit29] Fang X., Wang Y., He P., Liao H., Zhang G., Li Y., Li Y. (2022). Org. Lett..

[cit30] Sun J., Nie Q., Fang X., He Z., Zhang G., Li Y., Li Y. (2022). Org. Biomol. Chem..

[cit31] Gao Y., Sun Y., Fang X., Zhao G., Li X., Zhang G., Li Y., Li Y. (2022). Org. Chem. Front..

[cit32] Cuozzo J. W., Clark M. A., Keefe A. D., Kohlmann A., Mulvihill M., Ni H., Renzetti L. M., Resnicow D. I., Ruebsam F., Sigel E. A., Thomson H. A., Wang C., Xie Z., Zhang Y. (2020). J. Med. Chem..

[cit33] Harris P. A., King B. W., Bandyopadhyay D., Berger S. B., Campobasso N., Capriotti C. A., Cox J. A., Dare L., Dong X., Finger J. N. (2016). J. Med. Chem..

[cit34] Harris P. A., Berger S. B., Jeong J. U., Nagilla R., Bandyopadhyay D., Campobasso N., Capriotti C. A., Cox J. A., Dare L., Dong X., Eidam P. M., Finger J. N., Hoffman S. J., Kang J., Kasparcova V., King B. W., Lehr R., Lan Y., Leister L. K., Lich J. D., MacDonald T. T., Miller N. A., Ouellette M. T., Pao C. S., Rahman A., Reilly M. A., Rendina A. R., Rivera E. J., Schaeffer M. C., Sehon C. A., Singhaus R. R., Sun H. H., Swift B. A., Totoritis R. D., Vossenkamper A., Ward P., Wisnoski D. D., Zhang D., Marquis R. W., Gough P. J., Bertin J. (2017). J. Med. Chem..

[cit35] Belyanskaya S. L., Ding Y., Callahan J. F., Lazaar A. L., Israel D. I. (2017). ChemBioChem.

[cit36] Shi Y., Wu Y., Yu J., Zhang W., Zhuang C. (2021). RSC Adv..

[cit37] Ertl P., Altmann E., McKenna J. M. (2020). J. Med. Chem..

[cit38] Wu R., Du T., Sun W., Shaginian A., Gao S., Li J., Wan J., Liu G. (2021). Org. Lett..

[cit39] Xu H., Tan T., Zhang Y., Wang Y., Pan K., Yao Y., Zhang S., Gu Y., Chen W., Li J. (2022). Adv. Sci..

[cit40] Ding Y., Clark M. A. (2015). ACS Comb. Sci..

[cit41] Ding Y., DeLorey J. L., Clark M. A. (2016). Bioconjugate Chem..

[cit42] Ding Y., Franklin G. J., DeLorey J. L., Centrella P. A., Mataruse S., Clark M. A., Skinner S. R., Belyanskaya S. (2016). ACS Comb. Sci..

[cit43] Li J. Y., Huang H. (2018). Bioconjugate Chem..

[cit44] Favalli N., Bassi G., Zanetti T., Scheuermann J., Neri D. (2019). Helv. Chim. Acta.

[cit45] Hunter J. H., Prendergast L., Valente L. F., Madin A., Pairaudeau G., Waring M. J. (2020). Bioconjugate Chem..

[cit46] Gartner Z. J., Kanan M. W., Liu D. R. (2002). Angew. Chem., Int. Ed..

[cit47] Wang X., Sun H., Liu J., Zhong W., Zhang M., Zhou H., Dai D., Lu X. (2019). Org. Lett..

[cit48] Xu H., Ma F., Wang N., Hou W., Xiong H., Lu F., Li J., Wang S., Ma P., Yang G., Lerner R. A. (2019). Adv. Sci..

[cit49] Chheda P. R., Simmons N., Schuman D. P., Shi Z. (2022). Org. Lett..

[cit50] Favalli N., Bassi G., Bianchi D., Scheuermann J., Neri D. (2021). Bioorg. Med. Chem..

[cit51] Siripuram V. K., Sunkari Y. K., Nguyen T.-L., Flajolet M. (2022). Front. Chem..

[cit52] Hunter J. H., Potowski M., Stanway-Gordon H. A., Madin A., Pairaudeau G., Brunschweiger A., Waring M. J. (2021). J. Org. Chem..

[cit53] Satz A. L., Cai J., Chen Y., Goodnow R., Gruber F., Kowalczyk A., Petersen A., Naderi-Oboodi G., Orzechowski L., Strebel Q. (2015). Bioconjugate Chem..

[cit54] Ma F., Li J., Zhang S., Gu Y., Tan T., Chen W., Wang S., Xu H., Yang G., Lerner R. A. (2022). ACS Catal..

[cit55] Qu Y., Liu S., Wen H., Xu Y., An Y., Li K., Ni M., Shen Y., Shi X., Su W. (2020). Biochem. Biophys. Res. Commun..

[cit56] Kolmel D. K., Meng J., Tsai M. H., Que J., Loach R. P., Knauber T., Wan J., Flanagan M. E. (2019). ACS Comb. Sci..

[cit57] Wen X., Duan Z., Liu J., Lu W., Lu X. (2020). Org. Lett..

[cit58] Cai P., Yang G., Zhao L., Wan J., Li J., Liu G. (2019). Org. Lett..

[cit59] Wang X., Sun H., Liu J., Dai D., Zhang M., Zhou H., Zhong W., Lu X. (2018). Org. Lett..

[cit60] Krumb M., Kammer L. M., Badir S. O., Cabrera-Afonso M. J., Wu V. E., Huang M., Csakai A., Marcaurelle L. A., Molander G. A. (2022). Chem. Sci..

[cit61] Phelan J. P., Lang S. B., Sim J., Berritt S., Peat A. J., Billings K., Fan L., Molander G. A. (2019). J. Am. Chem. Soc..

[cit62] Bonin H., Fouquet E., Felpin F. X. (2011). Adv. Synth. Catal..

[cit63] Oger N., d'Halluin M., Le Grognec E., Felpin F.-X. (2014). Org. Process Res. Dev..

[cit64] Felpin F.-X., Sengupta S. (2019). Chem. Soc. Rev..

[cit65] Pastre J. C., Correia C. R. D. (2009). Adv. Synth. Catal..

[cit66] Stern T., Rueckbrod S., Czekelius C., Donner C., Brunner H. (2010). Adv. Synth. Catal..

[cit67] Mo F., Qiu D., Zhang L., Wang J. (2021). Chem. Rev..

[cit68] Zimmermann F. K. (1977). Mutat. Res..

[cit69] Frankel A., Duncan B., Hartman P. (1980). J. Bacteriol..

[cit70] Cheng K., Wang C., Ding Y., Song Q., Qi C., Zhang X.-M. (2011). J. Org. Chem..

[cit71] Cheng K., Zhao B., Hu S., Zhang X.-M., Qi C. (2013). Tetrahedron Lett..

[cit72] Li G., Liu Y., Liu Y., Chen L., Wu S., Liu Y., Li X. (2013). Angew. Chem., Int. Ed..

[cit73] Zhao G., Zhong S., Zhang G., Li Y., Li Y. (2022). Angew. Chem., Int. Ed..

