# Supplementary material for: Aryl diazonium intermediates enable mild DNA-compatible C–C bond formation for medicinally relevant combinatorial library synthesis
Source: Chem Sci. 2022 Oct 25;13(44):13100–9. doi: 10.1039/d2sc04482j (PMC9667928; doi:10.1039/d2sc04482j)
Supplement: SC-013-D2SC04482J-s001 [file SC-013-D2SC04482J-s001.pdf]

Electronic Supplementary Material (ESI) for Chemical Science.
This journal is © The Royal Society of Chemistry 2022

Supplementary Information

Aryl diazonium intermediates enable mild DNA-compatible C-C bond formation for medicinally relevant combinatorial library synthesis

Xianfeng Li,‡[a] Juan Zhang,‡[a] Changyang Liu,[a] Jie Sun,[a] Yangfeng Li,[ab] Gong Zhang*[ab] and Yizhou Li*[abcd]

Affiliation

[a] *Chongqing Key Laboratory of Natural Product Synthesis and Drug Research, Innovative Drug Research Center, School of Pharmaceutical Sciences, Chongqing University, Chongqing 401331, P. R. China.*

[b] *Chemical Biology Research Center, School of Pharmaceutical Sciences, Chongqing University, 401331, Chongqing, P. R. China.*

[c] *Key Laboratory of Biorheological Science and Technology, Ministry of Education, College of Bioengineering, Chongqing University, Chongqing, 400044, P. R. China*

[d] *Beijing National Laboratory for Molecular Sciences, 100190 Beijing, P. R. China*

‡ These authors contributed equally to this work.

* Corresponding authors:

E-mail address: gongzhang@cqu.edu.cn (Gong Zhang); yizhouli@cqu.edu.cn (Yizhou Li)

Contents

1. Abbreviations....4

2. Materials and General Methods....5

2.1 Materials and equipment....5

2.2 DNA sequences and characterization....6

2.3 General methods for monitoring and characterization of small molecules....6

2.4 General methods for DNA purification and quantification....6

2.5 General procedure for DNA ligation....8

2.6 General procedure for polyacrylamide gel electrophoresis....8

2.7 General procedure for agarose gel electrophoresis....8

3. Investigation of on-DNA diazotization conditions by azide transformation....10

3.1 General procedure for on-DNA amidation....10

3.2 Optimization of diazotization reagent and the formation of on-DNA phenyl azide....10

3.3 Demonstration of substrate scope of tBuONO diazotization condition....15

4. Standard reaction establishment of on-DNA Pd-catalyzed coupling via aryl diazonium intermediates....17

4.1 General methods for on-DNA Suzuki-Miyaura coupling....17

4.2 General methods for on-DNA Heck reaction....18

4.3 General methods for on-DNA Hiyama reaction....19

4.4 Optimization of the model reaction....20

4.5 Structure validation of on-DNA synthesized products....21

4.5.1 Validation of on-DNA Suzuki-Miyaura coupling product 3a by co-injection assay..21

4.5.2 Off-DNA synthesis of authentic S5a....22

4.5.3 Validation of on-DNA Heck coupling product 5a by co-injection assay....24

4.5.4 Validation of On-DNA Hiyama coupling product 10a by co-injection assay....25

5. Substrate scopes of on-DNA Suzuki-Miyaura, Heck reaction and Hiyama reaction....26

5.1 Substrate scope of boronic acids for the on-DNA Suzuki-Miyaura coupling....26

5.2 Substrate scope of DNA-conjugated arylamines for the on-DNA Suzuki-Miyaura coupling....29

5.3 Substrate scope of olefins for the on-DNA Heck reaction....31

5.4 Substrate scope of DNA-conjugated arylamines for the on-DNA Heck reaction....35

5.5 Substrate scope of arylamines for the reverse on-DNA Heck reaction....41

5.6 Substrate scope of DNA-conjugated arylamines for the on-DNA Hiyama reaction 43
5.7 Scale-up reactions 44
6. Application of on-DNA carbon-carbon bond formation in DEL construction 46
6.1 on-DNA branched molecule synthesis 46
6.2 on-DNA linear molecule synthesis 47
6.2 Verification of DNA enzymatic ligation compatibility 48
6.3 Verification of PCR and sequencing compatibility of DNA barcodes 49
6.4 DNA Sequences 52
7. Application of on-DNA carbon-carbon bond formation in DNA-encoded pharmaceutical compound and chemical probe synthesis 53
7.1 Comparative study of on-DNA functional group synthesis 53
7.2 Comparative study of on-DNA pharmacophore (HDAC inhibitor) synthesis 57
7.3 General method for DNA Programmed Affinity Labeling (DPAL) 60
8. UPLC chromatograph and deconvoluted MS 61
8.1 Substrate scope of DNA-conjugated arylamines for the On-DNA azide transformation ..61
8.2 Substrate scope of DNA-conjugated azide for the On-DNA CuAAC reaction 65
8.3 Substrate scope of boronic acids for the on-DNA Suzuki-Miyaura coupling with 1a 68
8.4 Substrate scope of DNA-conjugated arylamines for the On-DNA Suzuki-Miyaura coupling 85
8.5 Substrate scope of olefins for the on-DNA Heck reaction with 1a 96
8.6 Substrate scope of DNA-conjugated arylamines for the on-DNA Heck reaction with 4o 123
8.7 Substrate scope of DNA-conjugated arylamines for the on-DNA Heck reaction with 4r 140
8.8 Substrate scope of anilines for the reverse on-DNA Heck reaction with 6a 157
8.9 Substrate scope of DNA-conjugated arylamines for the On-DNA Hiyama coupling 164
9. Reference 170

1. Abbreviations

BP: binding probe

CP: capture probe

CuAAC: Copper-catalyzed azide-alkyne cycloaddition

DCM: dichloromethane

DIPEA: *N, N*-diisopropylethylamine

DMA: *N, N*-dimethyl acetamide

DMT-MM: 4-(4, 6-dimethoxy-1,3,5-triazin-2-yl)-4-methylmorpholinium chloride

HFIP: 1, 1, 1, 3, 3, 3-hexafluoro-2-propanol

HP: headpiece

HP-P: headpiece primer

HPLC: high performance liquid chromatography

NMR: nuclear magnetic resonance

PAGE: polyacrylamide gel electrophoresis

PB: phosphate buffer

PBS: phosphate buffered saline

TEAA: triethylammonium acetate

TEA: trimethylamine

TLC: thin layer chromatography

TIC: total ion chromatogram

UPLC-MS: Ultra performance liquid chromatography- mass spectrum

UV: ultraviolet

2. Materials and General Methods

2.1 Materials and equipment

Unless otherwise noted, all reagents and solvents were purchased from commercial sources and used as received. Chemicals and reagents were purchased from several commercial suppliers including Bidepharm, Adamas and Sigma-Aldrich, etc., and were generally used without further purification. T4 DNA ligase and 10× ligation buffer (500 mM Tris pH 7.5, 500 mM NaCl, 100 mM $MgCl_2$, 100 mM DTT and 25 mM ATP) were purchased from HitGen Inc.. All buffers and aqueous solutions were prepared in-house. Protein samples were separated and analyzed by SDS-PAGE. All gel images were captured by a Bio-Rad Chemidoc^{TM} imaging system. Photo-crosslinking experiments were conducted by a UVP CL-1000 Ultraviolet crosslinker at 365 nm wavelengths. Concentrations of oligonucleotides were determined by the absorbance at 260 nm with a BioTek Epoch 2 plate reader.

Experimental data were achieved with the equipment listed below: NMR Spectrometers (Agilent 400 MHz), UHPLC-MS (Agilent 1290 and Agilent 6230 Time-of-Flight), HPLC (Waters 1575EF Series), UV-Vis spectrometer (BioTek Epoch), centrifuge (Eppendorf 5424 R), DNA synthesizer (Applied Biosystems 3400) and other instruments.

2.2 DNA sequences and characterization

a) DNA sequences

All the DNA sequences were written in 5’-to 3’-orientation unless otherwise noted.

Headpiece (HP):

5’-/5Phos/GAGTCA/iSp9/iUniAmM/iSp9 /TGACTCCC-3’

Headpiece-primer (HP-P):

5’-/5Phos/ACCTTCGGTCGGGAGTCA/iSp9/iUniAmM

/iSp9/TGACTCCCGACCGAAGGTTG-3’

Binding Probe (BP): 5’-NH_2-TGA CTC CCG ACC GAA GGT TG-3’

Capture Probe (CP): 5’-FAM-ACC TTC GGT CGG GAG TCA-NH_2-3’

b) Mass spectrometry characterization

DNA	expected mass	observed mass
HP	4937	4937
HP-P	12408	12408
BP	6284	6284
CP	6249	6249

2.3 General methods for monitoring and characterization of small molecules

Reactions were monitored by TLC or UPLC-MS. TLC analysis was visualized by using general staining reagents or UV light (λ = 254 nm). Novel synthetic compounds were characterized by ^{1}H-NMR, ^{13}C-NMR, and HRMS. NMR spectrum was recorded on Agilent 400 MHz spectrometer using residual non-deuterated solvent (DMSO-d_6) as the internal standard. Multiplicity abbreviations are as follows: s = singlet, brs = broad singlet, d = doublet (dd = doublet of doublets), t = triplet, q = quartet, m = multiplet. Unless otherwise noted, all deuterated solvents were purchased from Adamas.

2.4 General methods for DNA purification and quantification

General method for ethanol precipitation: To the DNA reaction mixture was added 10% (v/v) 3 M acetate buffer (pH 5.2) and 3 times the volume of cold (-20 ^{o}C) ethanol subsequently. The solution was maintained at -80 ^{o}C for over 2 h and then centrifuged

at 13,500 rpm for 30 min at 4 °C. The supernatant was discarded and the pellet was rinsed once with cold 70% ethanol. After centrifuging at 13,500 rpm for another 10 min at 4 °C, the supernatant was discarded and the pellet was dried by a speedvac. The recovered sample was then dissolved in an appropriate solvent for subsequent analysis or experiments. Eppendorf 5424R centrifuge is used for ethanol precipitation of all the DNA samples.

General method for HPLC purification: Preparative reversed-phase high-pressure liquid chromatography (RP-HPLC) for the DNA conjugate was performed on a Waters 1575EF Series with a reversed-phase HPLC column (Eclipse-XDB C18, 5 μM, 9.4×250 mm) using eluent A (100 mM TEAA in H_2O) and eluent B (100 mM TEAA in 80% MeCN) with gradient: 10% B (0 to 1 min), 10% to 30% B (1 to 11min), 30% to 100% B (11 to 11.1 min), 100% B (11.1 to 12 min), 100% to 10% B (12 to 12.1 min), 10% B (12.1 to 16 min). The fractions containing the product were combined and lyophilized overnight.

General methods for DNA analysis: Analysis of on-DNA reactions by UPLC-MS: After the reaction, an aliquot of the reaction mixture was diluted with H_2O to approximately 1 μM, 10-20 μL sample was injected into a reversed-phase UPLC column (Agilent, AdvanceBio Oligonucleotide, C18, 2.1×50 mm, 2.7 μm, maintained at 60 °C) at a flow rate of 0.3 mL/min. (Solvent A: 200 mM HFIP and 8 mM TEA in H_2O; Solvent B: MeOH). The effluent was detected by absorbance at 260 nm, and analyzed on Agilent TOF (6230 B) in negative ion mode.

Method I: 5% to 15% B (0 to 1 min), 15% to 25% B (1 to 2 min), 25% to 30% B (2 to 5.5 min), 30% to 90% B (5.5 to 6 min), 90% B (6 to 6.5 min), 90% to 5% B (6.5 to7 min), 5% B (7 to 8 min).

Method II: 5% to 15% B (0 to 1 min), 15% to 30% B (1 to 12 min), 30% to 90% B (12 to 12.1 min), 90% B (12.1 to 13 min), 90% to 5% B (13 to 13.1 min), 5% B (13.1 to 14 min).

Method III: 3% to 12% B (0 to 1 min), 12% to 18% B (1 to 2.5 min), 18% to 20% B (2.5 to 4 min), 20% to 22% B (4 to 6 min), 22% to 30% B (6 to 9 min), 30% to 85% B

(9 to 10 min), 85% B (10 to 11 min), 85% to 3% B (11 to 12 min)

Conversion calculation: The conversion of on-DNA product was determined from UV absorbance trace (260 nm) peak area by using the equation: conversion% = UV (product)/UV (total DNA recovered), ignoring the UV coefficient difference among all the DNA products and assuming 100% DNA recovery. Any non-oligo material that had an absorbance at UV 260 nm was subtracted from the conversion calculation.

Analysis of molecular mass: Observed m/z could be calculated as m/z = (m - z)/z. BioConfirm software (Agilent, v10.0) was used to deconvolute the multiple charge states.

2.5 General procedure for DNA ligation

This reaction contained variably derivatized **HP-P** starting material (10 nmol in H_2O, 1 equiv.), Code (12 nmol in H_2O, 1.2 equiv.), 10 × ligation buffer (4 μL), T4 DNA ligase (1 μL, 1000 units/μL) and nuclease-free H_2O (to the total volume of 40 μL). The reaction mixture was incubated at 20 ^{o}C for overnight before performing gel analysis. The crude material was purified by ethanol precipitation and subjected to the next step of synthesis without further purification.

2.6 General procedure for polyacrylamide gel electrophoresis

Ligation reactions were monitored by gel electrophoresis with 20% urea polyacrylamide gel in 1× TBE buffer (89 mM Tris-Borate, 2 mM EDTA, pH 8.3) system referenced by a 20 bp DNA ladder (Takara, Japan). Before gel loading, the DNA samples were denatured at 95 ^{o}C for 10 min and mixed with 0.20 volume of the 6× gel loading buffer. Then, 10 pmol of treated DNA samples were loaded on the gel, and the gel was run at 200 V for 50-60 min. DNA fragments were visualized and analyzed by Bio-Rad Chemidoc^{TM} Image System (Bio-Rad, CA, USA).

2.7 General procedure for agarose gel electrophoresis

Ligation reactions were monitored by 3% Agarose gel electrophoresis in 1× TBE buffer (89 mM Tris-Borate, 2 mM EDTA, pH 8.3) system referenced by a 20 bp DNA

ladder (Takara, Japan). Before gel loading, the DNA samples mixed with 0.20 volume of the 6× gel loading buffer. Then, 10 pmol of treated DNA samples were loaded on the gel, and the gel was run at 100 V for 30-45 min. DNA fragments were visualized and analyzed by Bio-Rad Chemidoc™ Image System (Bio-Rad, CA, USA).

3. Investigation of on-DNA diazotization conditions by azide transformation

3.1 General procedure for on-DNA amidation

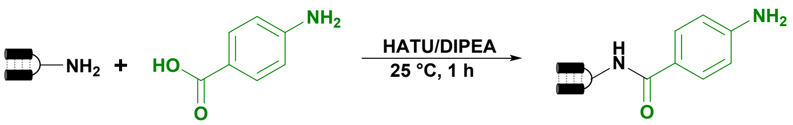

Pre-mixed solution: 4-aminobenzoic acid (4 μL, 200 mM in DMA), HATU (4 μL, 200 mM in DMA), and DIPEA (4 μL, 200 mM in DMA).

To a solution of **HP** in pH 9.4 borate buffer (10 μL, 1 mM in 250mM sodium borate buffer) was added 6 μL pre-mixed solution. The mixture was vortexed and reacted at room temperature for 10 min, then was added another 4 μL of pre-mixed solution. The mixture was vortexed and reacted at room temperature for 1 h, followed by ethanol precipitation.[1] The product was purified by preparative HPLC and quantified by UV absorption at 260 nm to give a conversion of >95%. The conjugate was characterized by UPLC-MS. Deconvoluted molecular mass: predicted: 5056; found: 5056.

3.2 Optimization of diazotization reagent and the formation of on-DNA phenyl azide

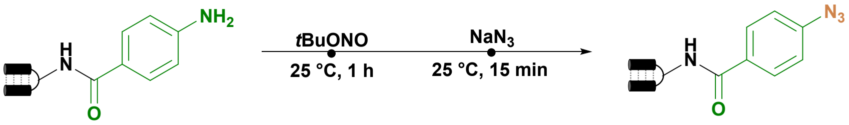

On-DNA aryl diazonium salt formation: To the DNA conjugate **1a** (8 μL, 0.025 mM in H_2O, 0.2 nmol) was added *t*BuONO (2 μL, 50 mM in DMA, 100 nmol) and H_2O (10 μL). Then the resulting mixture was vortexed and allowed to react at 25 °C for 1 h.

On-DNA phenyl azide formation: To the solution of aryl diazonium intermediate was added NaN_3 (2 μL, 500 mM in H_2O, 1000 nmol). The reaction system was vortexed and stood at 25 °C for another 15 min. The product was ethanol precipitated and subjected to UPLC-MS analysis.

Supplementary Table 1. Investigation of DNA compatibility of diazotization

	Entry	DNA	Nitrite	Acid	Conversion (%)
**Condition 1**	1	HP-ph-NH_2	$NaNO_2$	HCl	>95
	2	HP-ph	$NaNO_2$	HCl	0
	3	HP	$NaNO_2$	HCl	0
**Condition 2**	4	HP-ph-NH_2	*t*BuONO	/	>95
	5	HP-ph	*t*BuONO	/	0
	6	HP	*t*BuONO	/	0

Condition 1: **1a** (8 μL, 25 μM in H_2O, 0.2 nmol), $NaNO_2$ (2 μL, 50 mM in H_2O, 100 nmol), HCl (2 μL, 100 mM in H_2O, 200 nmol), and H_2O (8 μL), 25 °C for 1 h. **Condition 2**: **1a** (8 μL, 25 μM in H_2O, 0.2 nmol), *t*BuONO (2 μL, 50 mM in DMA, 100 nmol) and H_2O (10 μL), 25 °C for 1 h.

When using $NaNO_2$, HCl or *t*BuONO as the diazotization reagent, the formation of the corresponding aryl azides was observed in both reaction conditions shown by Method ***I***. But when Method ***II*** (an elution method with the more gradual gradient) was used for detection, damage was detected in the $NaNO_2$, HCl diazotization group. In detail, a tail peak was noticed after the major product peak, with the deconvoluted mass corresponding to a +1 Da shifted byproduct (Supplementary Fig. 1). Combining our data with the previously reported literature, we inferred that the byproduct matched the molecular weight of the nucleobase deamination side reaction (Supplementary Fig. 2). On the contrary, no obvious byproduct was observed in the *t*BuONO diazotization group.

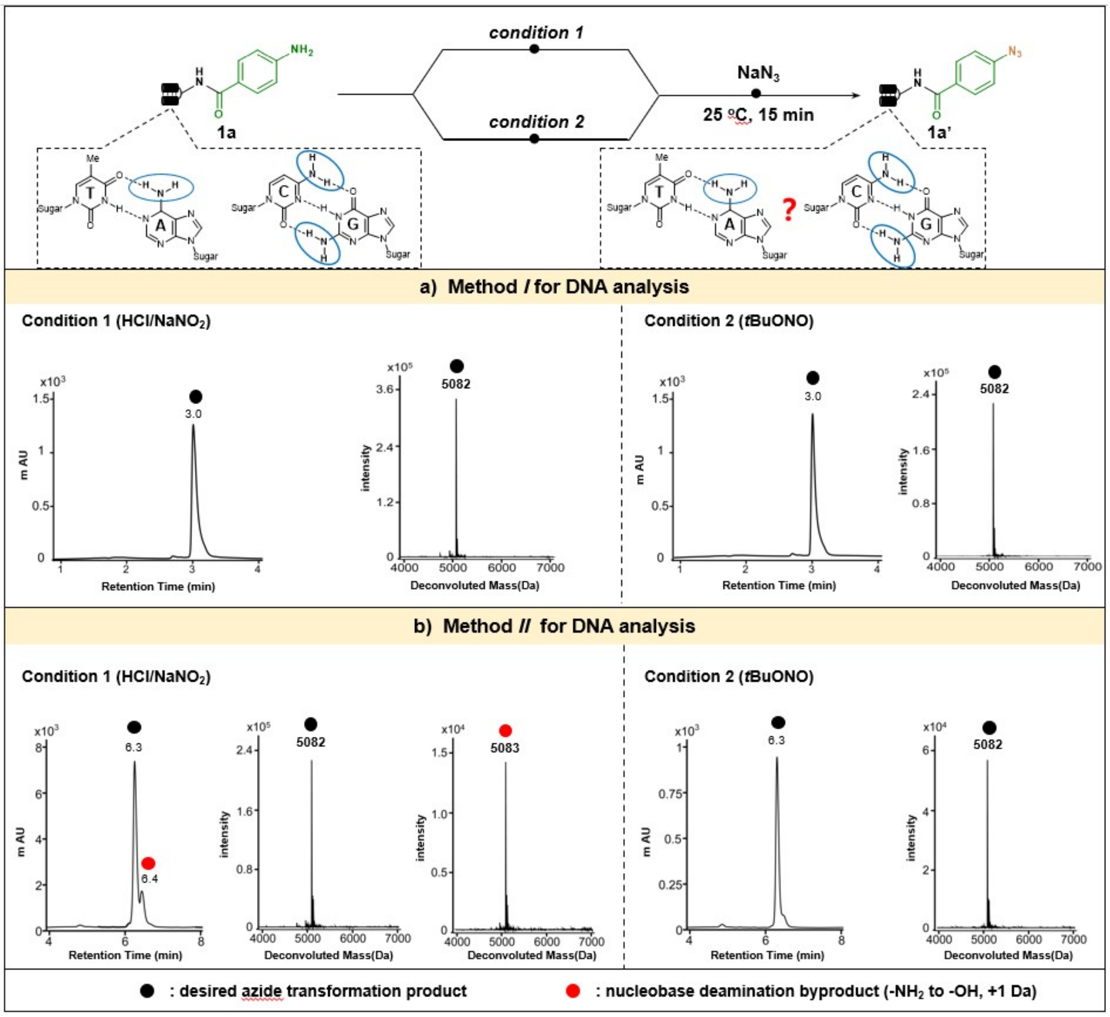

Supplementary Fig.1 Investigation of DNA-compatible diazotization condition using Method *I* and *II* for UPLC-MS analysis

No difference was observed between $NaNO_2$, HCl group and *t*BuONO group in aspects of chromatography and mass spectrometry by Method ***I***. To examine the results carefully, we applied the method ***II*** with a different elution gradient. DNA damage was observed with $NaNO_2$, HCl treatment in aspects of chromatography and mass spectrometry by Method ***II***. According to the literature, it is speculated that the DNA damage is caused by the diazotization of bases on DNA, including three bases containing arylamine-like structures, that is, Guanine, Adenine and Cytosine (Supplementary Fig. 2).

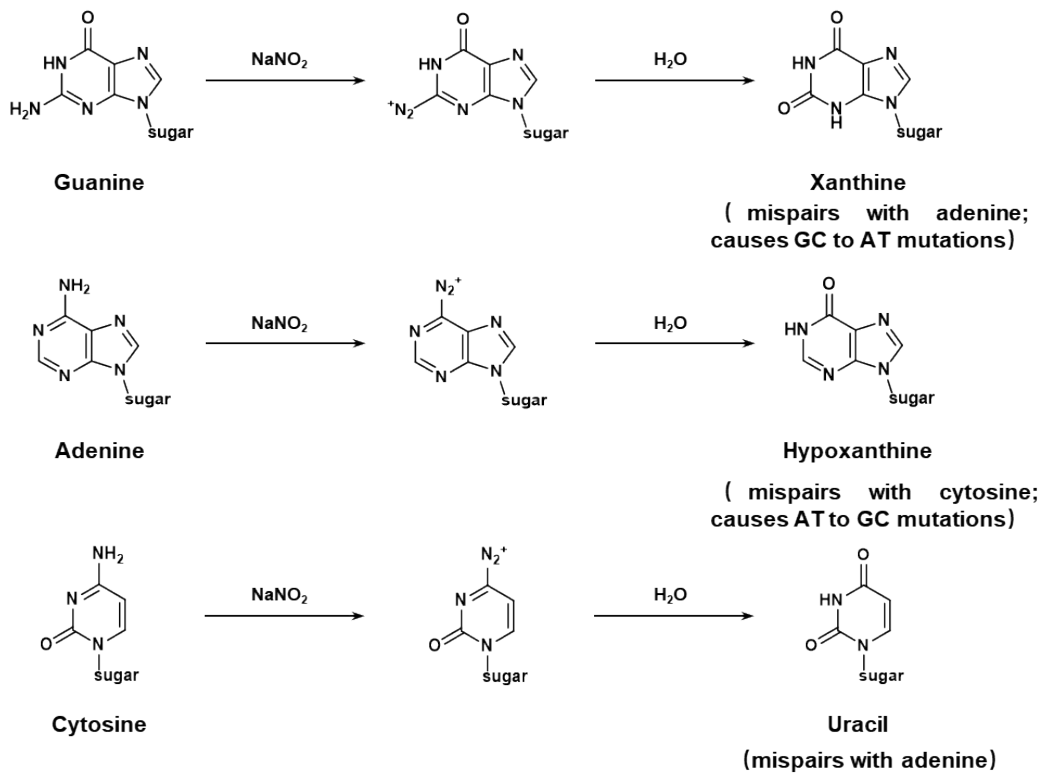

Supplementary Fig. 2 Reported Mechanisms of nitrous acid-induced DNA mutation

Next, to further validate our diazotization strategy was compatible with DEL construction, we tried to evaluate the influence of the diazotization conditions to the enzymatic ligation process of DNA barcodes. For this experiment, we treated the DNA Headpiece (HP) with different diazotization reagents (*t*BuONO or $NaNO_2$/HCl) of different concentrations (5 mM or 50 mM final concentration), respectively, and then performed T4 ligase-assisted enzymatic ligation experiments. By this way, we aimed to assess whether the diazotization strategy would potentially affect the construction of DELs.

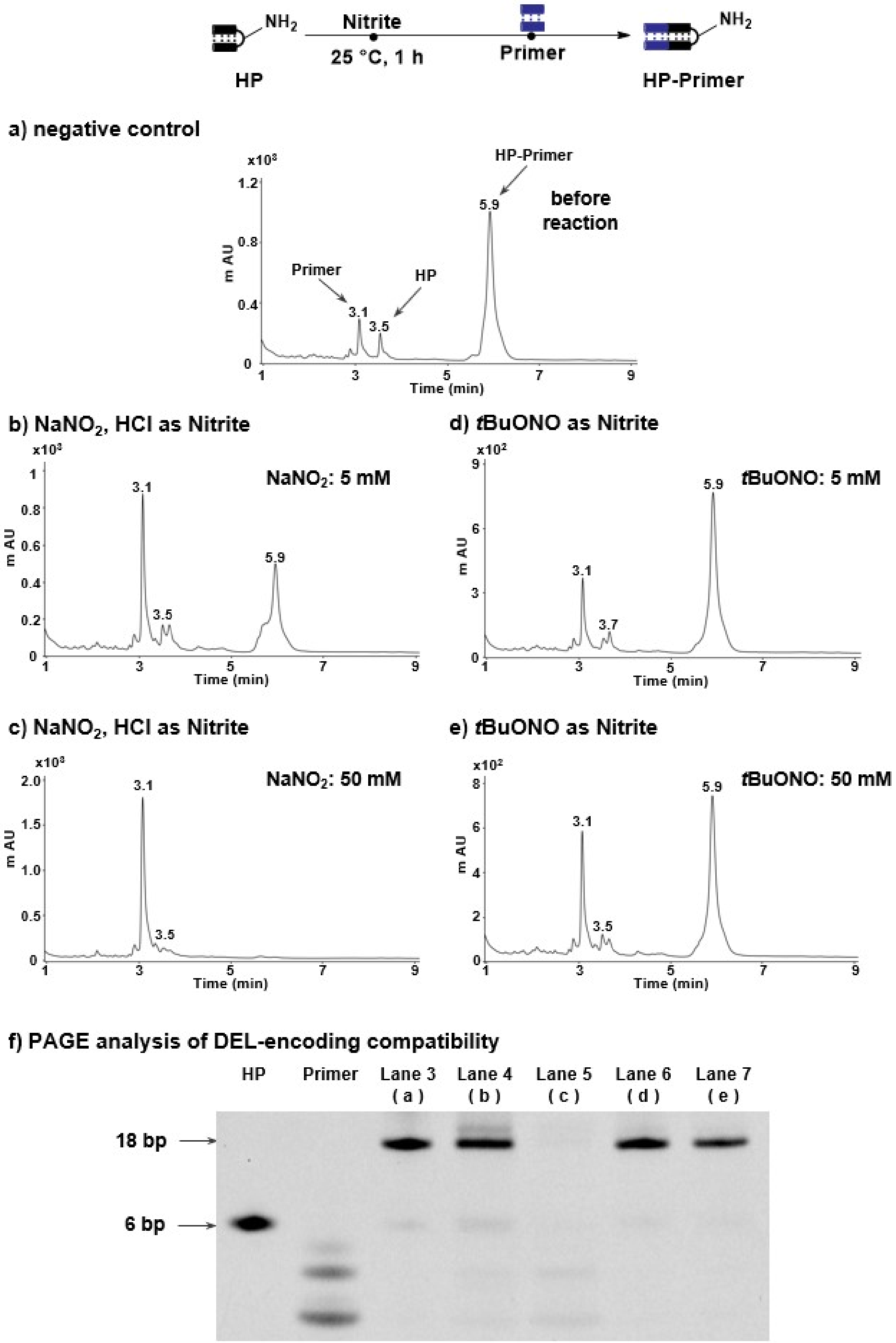

Supplementary Fig. 3 Evaluation of DEL-encoding compatibility with different diazotization strategies. (a-e) LC chromatograms of different diazotization conditions. **(f)** PAGE analysis of enzymatic ligation results**. Lane 1**: HP; **Lane 2**: primer; **Lane 3**: HP + primer, sample from **Fig. 3 a)**; **Lane 4**: HP ($NaNO_2$, 5 mM) + primer, sample from **Fig. 3 b)**; **Lane 5**: HP ($NaNO_2$, 50 mM) + primer, sample from **Fig. 3 c)**; **Lane 6**: HP (*t*BuONO, 5 mM) + primer, sample from **Fig. 3 d); Lane 7** HP (*t*BuONO, 50 mM) + primer, sample from **Fig. 3 e)**.

According to mass spectrometry data and enzymatic ligation results, we could reach the conclusion that *t*BuONO had better compatibility with DEL construction compared with the same concentration of $NaNO_2$, HCl reagent. Therefore, we chose *t*BuONO as the diazotization reagent for further experiments.

3.3 Demonstration of substrate scope of tBuONO diazotization condition

Next, to prove the generality of *t*BuONO as a diazotization reagent on DNA, we used *t*BuONO to treat a diverse range of DNA-conjugated anilines, followed by NaN_3 nucleophilic substitution. Results demonstrated that the typical DNA-conjugated aniline substrates gained good to excellent conversion. Then, we attempted to convert the aryl azide generated in the previous step into the corresponding triazole product by CuAAC reaction. All the aryl azide yielded 1,2,3-triazole products with good to excellent conversion (Supplementary Fig. 4).

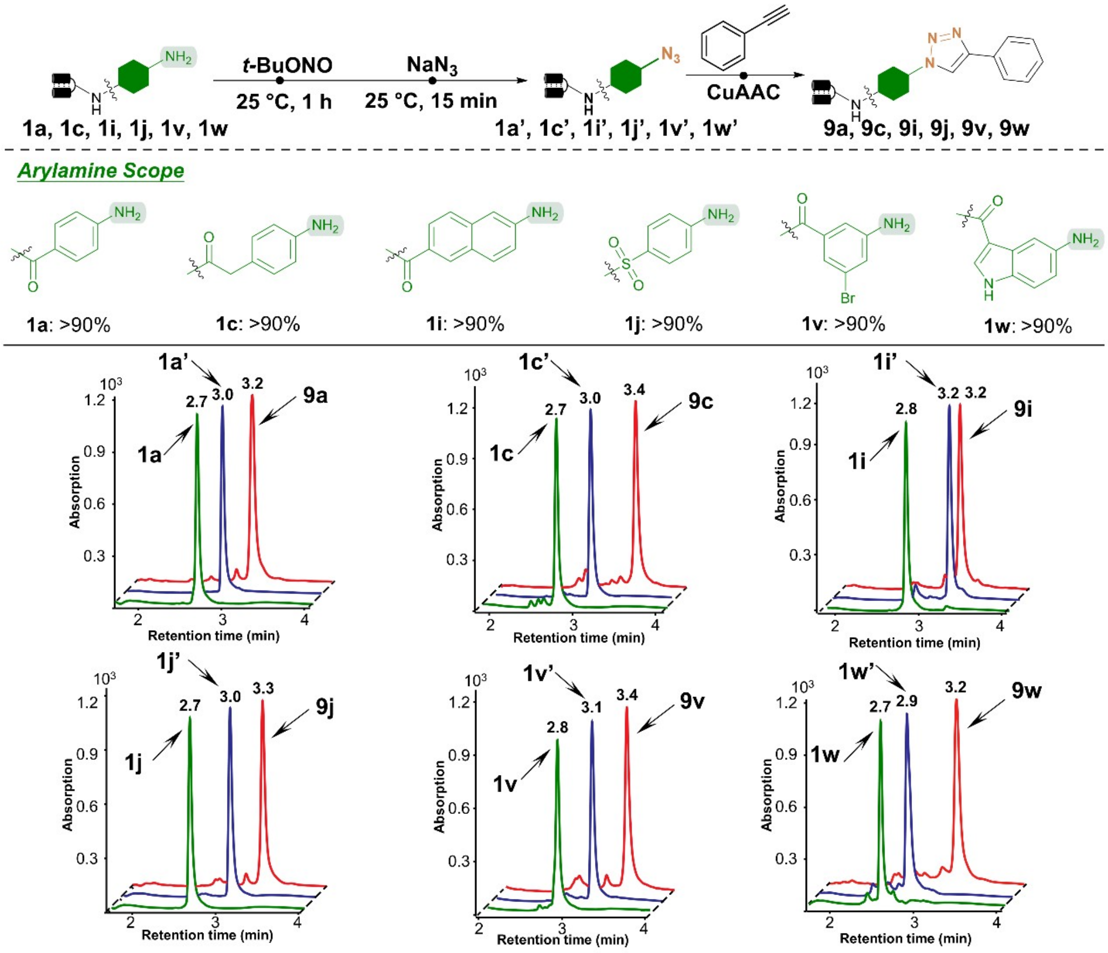

Supplementary Fig. 4 Substrate scope of DNA-conjugated arylamines.

The "green" curves indicate the arylamine starting materials, the "blue" curves represent the aryl azide product, while the "red" curves represent the final CuAAC products.

4. Standard reaction establishment of on-DNA Pd-catalyzed coupling via aryl diazonium intermediates

4.1 General methods for on-DNA Suzuki-Miyaura coupling

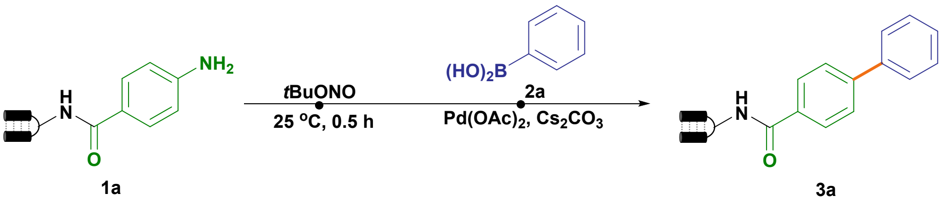

On-DNA aryl diazonium salt formation: To the DNA conjugate **1a** (8 µL, 25 µM in H_2O, 0.2 nmol) was added *t*BuONO (2 µL, 50 mM in DMA, 100 nmol) and H_2O (10 µL). Then the resulting mixture was vortexed and stood at 25 °C for 0.5 h.

On-DNA Suzuki-Miyaura Coupling: After the aryl diazonium intermediate was generated according to the above method, $Pd(OAc)_2$ (2 µL, 20 mM in DMA, 40 nmol) and boronic acid (2 µL, 500 mM in DMA, 1000 nmol) were added. The mixture was vortexed and finally supplemented with Cs_2CO_3 (2 µL, 800 mM in H_2O, 1600 nmol) and 4 µL H_2O, and allowed for reaction at 40 °C for 2 h. After reaction, 30 equiv. of sodium diethyldithiocarbamic acid compared with $Pd(OAc)_2$ were added to the mixture to scavenge Pd, and the reaction mixture was stood at 25 °C for 30 minutes. The mixture was centrifuged at 25 °C for 10 min at 13,500 rpm, and the resultant supernatant was collected. The product was obtained by ethanol precipitation as described above and characterized by UPLC-MS. All the DNA-aniline conjugates and boronic acids in substrate scope study were performed using the method above. During the reaction, attention must be paid to: the sequence of adding reagents should not be reversed.

4.2 General methods for on-DNA Heck reaction

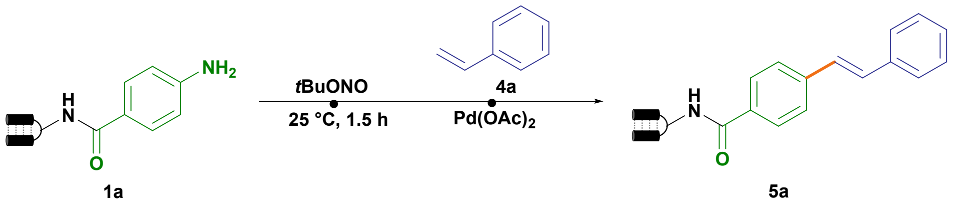

On-DNA aryl diazonium salt formation: To the DNA conjugate **1a** (8 μL, 25 μM in H_2O, 0.2 nmol) was added with *t*BuONO (2 μL, 50 mM in DMA, 100 nmol) and H_2O (10 μL). Then the resulting mixture was vortexed and stood at 25 °C for 1.5 h.

On-DNA Heck reaction: After the aryl diazonium intermediate was generated according to the above method, $Pd(OAc)_2$ (2 μL, 20 mM in DMA, 40 nmol) was added. The mixture was vortexed and added a mixed solution of phosphate buffer (3 μL, 250 mM in H_2O, pH 5.5, 750 nmol), H_2O (3 μL) and styrene (2 μL, 500 mM in DMA, 1000 nmol), then stood for reaction at 25 °C for 30 min. After reaction, 30 equiv. of sodium diethyldithiocarbamic acid (compared with $Pd(OAc)_2$) was added to the mixture, and the reaction mixture was stood at 25 °C for 30 minutes. The mixture was centrifuged at 25 °C for 10 min at 13,500 rpm, and the resultant supernatant was collected. The product was obtained by ethanol precipitation as described above and characterized by UPLC-MS. All the DNA-aniline conjugates and olefins were performed using the method above. During the reaction, attention must be paid to: the sequence of adding reagents should not be reversed.

4.3 General methods for on-DNA Hiyama reaction

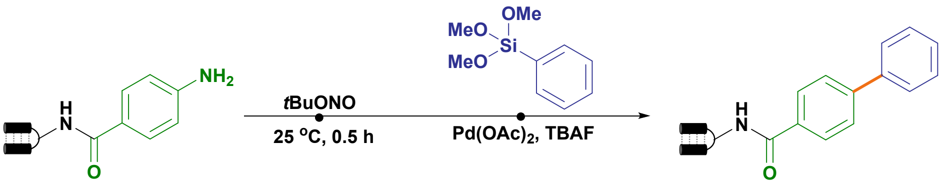

1a 3a

On-DNA aryl diazonium salt formation: To the DNA conjugate **1a** (8 μL, 25 μM in H_2O, 0.2 nmol) was added *t*BuONO (2 μL, 50 mM in DMA, 100 nmol) and H_2O (10 μL). Then the resulting mixture was vortexed and stood at 25 °C for 0.5 h.

On-DNA Hiyama Coupling: After the aryl diazonium intermediate was generated according to the above method, $Pd(OAc)_2$ (2 μL, 20 mM in DMA, 40 nmol) was added. The mixture was vortexed and added a mixed solution of phosphate buffer (6 μL, 250 mM in H_2O, pH 5.5, 1500 nmol), phenyltrimethoxysilane (2 μL, 500 mM in DMA, 1000 nmol) were added. The mixture was vortexed and finally supplemented with TABF (2 μL, 50 mM in H_2O, 100 nmol) , H_2O to 100 μL , and allowed for reaction at 40 °C for 1 h. After reaction, 30 equiv. of sodium diethyldithiocarbamic acid compared with $Pd(OAc)_2$ were added to the mixture to scavenge Pd, and the reaction mixture was stood at 25 °C for 30 minutes. The mixture was centrifuged at 25 °C for 10 min at 13,500 rpm, and the resultant supernatant was collected. The product was obtained by ethanol precipitation as described above and characterized by UPLC-MS. All the DNA-aniline conjugates and boronic acids in substrate scope study were performed using the method above. During the reaction, attention must be paid to: the sequence of adding reagents should not be reversed.

4.4 Optimization of the model reaction

Supplementary Table 2. Optimization of Suzuki-Miyaura coupling

Entry	Catalyst	Base	Temperature (°C)	Conversion (%)
1	$Pd(OAc)_2$	——	25	60
2	$Pd(OAc)_2$	NaOH	25	64
3	$Pd(OAc)_2$	CsOH	25	59
4	$Pd(OAc)_2$	Cs_2CO_3	25	68
5	$Pd(OAc)_2$	Et_3N	25	62
6	$Pd(OAc)_2$	NaOAc	25	60
7	$Pd(OAc)_2$	DIPEA	25	46
8	$Pd(OAc)_2$	Cs_2CO_3	25	68
9	$PdCl_2$	Cs_2CO_3	25	54
10	$PdCl_2(COD)$	Cs_2CO_3	25	<10
11	$PdCl_2(MeCN)_2$	Cs_2CO_3	25	57
12	sSPhos-Pd-G2	Cs_2CO_3	25	<10
13	$Pd(CF_3COO)_2$	Cs_2CO_3	25	65
**14**	**$Pd(OAc)_2$**	**Cs_2CO_3**	**40**	**73**
15	$Pd(OAc)_2$	Cs_2CO_3	60	68
16	$Pd(OAc)_2$	Cs_2CO_3	80	67

Standard reaction conditions: After the aryl diazonium intermediate was generated according to the above method, $Pd(OAc)_2$ (2 µL, 20 mM in DMA, 40 nmol) and boronic acid (2 µL, 500 mM in DMA, 1000 nmol) were added. The mixture was vortexed and finally added base solution (2 µL, 800 mM in H_2O, 1600 nmol), 4 µL H_2O, and let for reaction at indicated temperature for 2 h. After reaction, 30 equiv. of sodium diethyldithiocarbamic acid (compared with $Pd(OAc)_2$ were added to the mixture to scavenge Pd, and the reaction mixture was stood at 25 °C for 30 minutes. The mixture was centrifuged at 25 °C for 10 min at 13,500 rpm, and the resultant supernatant was collected. The product was obtained by ethanol precipitation as described above and characterized by UPLC-MS.

4.5 Structure validation of on-DNA synthesized products

4.5.1 Validation of on-DNA Suzuki-Miyaura coupling product 3a by co-injection assay

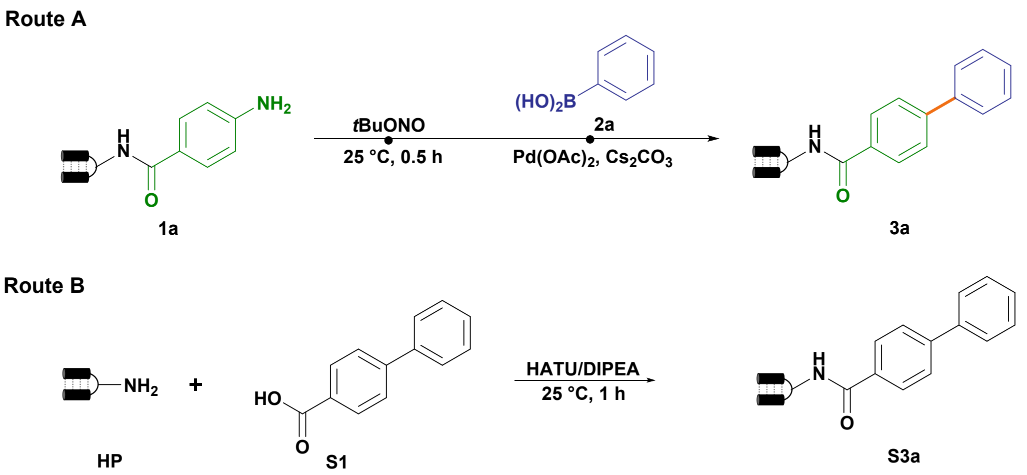

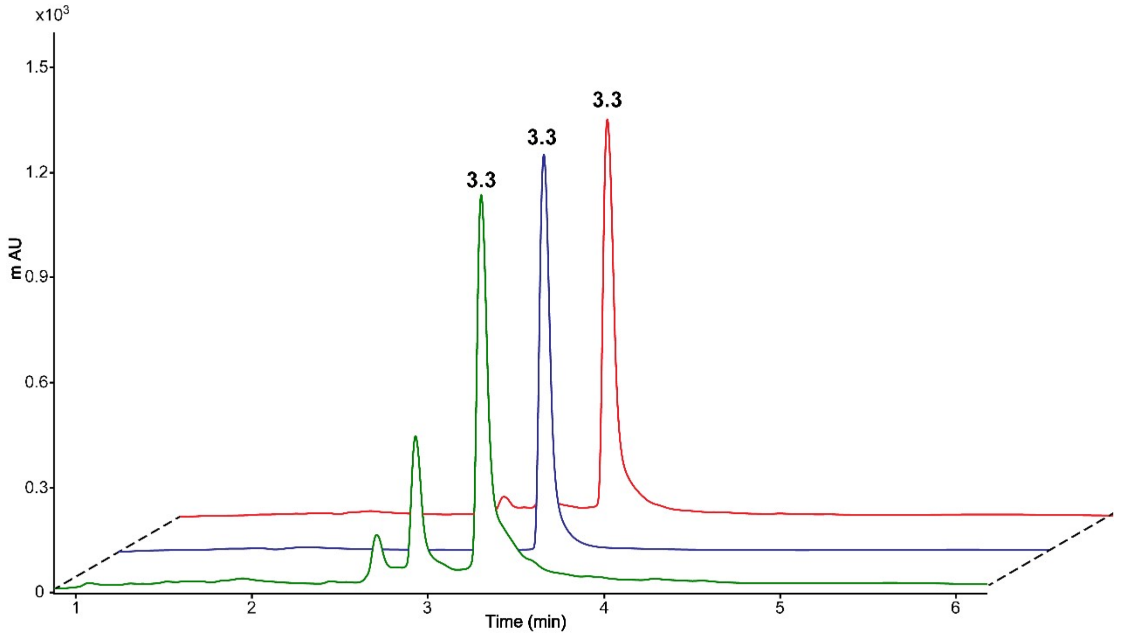

Supplementary Fig. 5 The co-injection experiment of 3a and S3a by UPLC-MS. The "red" curve is the co-injection sample mixing **3a** and **S3a**; The "green" curve is **3a**; The "blue" curve is **S3a**.

The on-DNA synthesized **3a** sample was co-eluted with the off-DNA synthesized and characterized **S3a** sample, indicating their structures were identical.

4.5.2 Off-DNA synthesis of authentic S5a

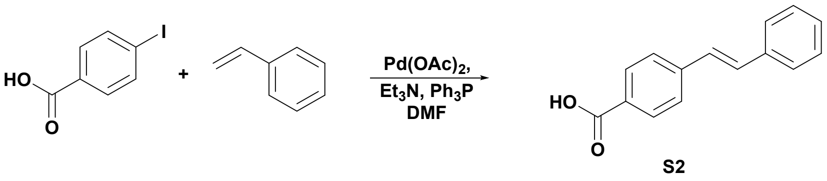

4-stillbene carboxylic acid (S2). Styrene (135 mg, 1.3 mmol) and 4-iodobenzoic acid (248 mg, 1 mmol) were dissolved in *N,N*-dimethylformamide (DMF, 2 mL) and triethylamine (2 mL). The solution was refluxed in the presence of triphenylphosphine (9.38 mg, 0.0358 mmol) and palladium (II) acetate (6.85 mg, 0.0305 mmol) for 24 h. After the removal of the solvent, ethyl acetate (20 mL) was added, and the soluble part was washed with three portions of H_2O (6 mL). The organic layer was reprecipitated in hexane (20 mL) to give 4-stilbene carboxylic acid (pale brown powder, 87 mg, 38.8%). ^{1}H NMR (400 MHz, DMSO-d_6) δ 12.94 (s, 1H), 7.94 (d, J = 8.3 Hz, 2H), 7.72 (d, J = 8.3 Hz, 3H), 7.64 (d, J = 7.4 Hz, 3H), 7.45 – 7.27 (m, 7H). ^{13}C NMR (101 MHz, DMSO-d_6) δ 167.12 (s), 141.44 (s), 136.64 (s), 130.99 (s), 129.81 (s), 129.48 (s), 128.82 (s), 128.24 (s), 127.41 (s), 126.85 (s), 126.51 (s).[2]

^{1}H-NMR of **S2**

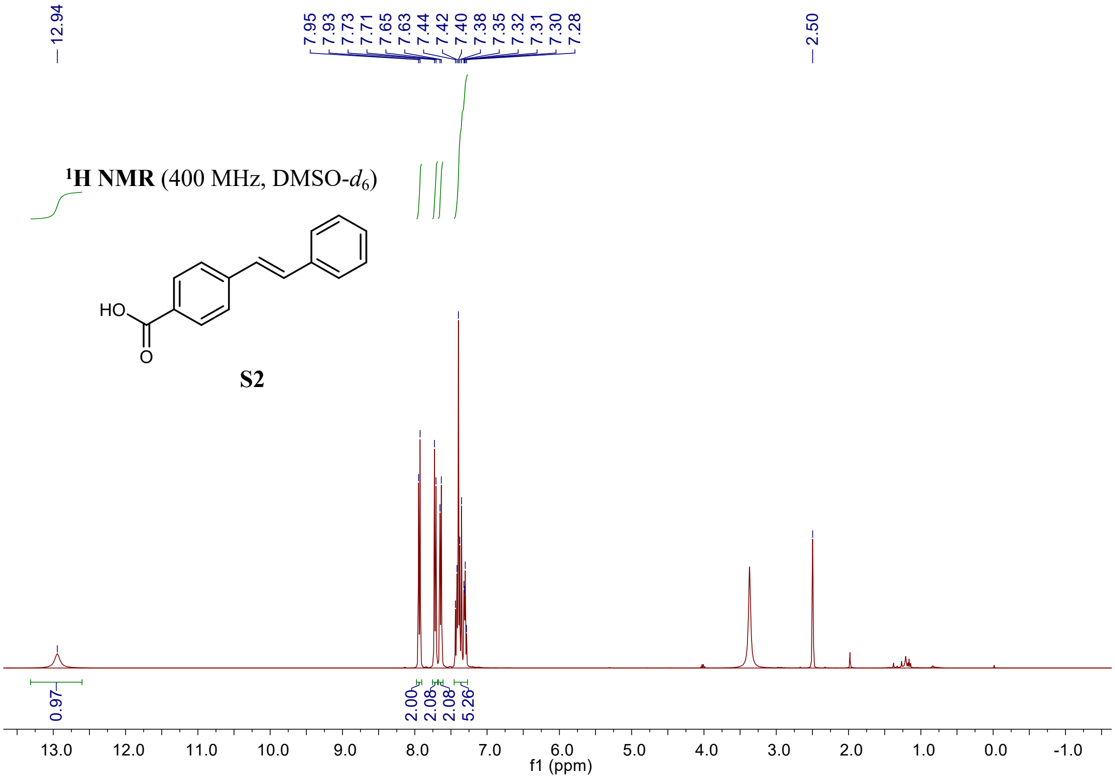

^{13}C-NMR of **S2**

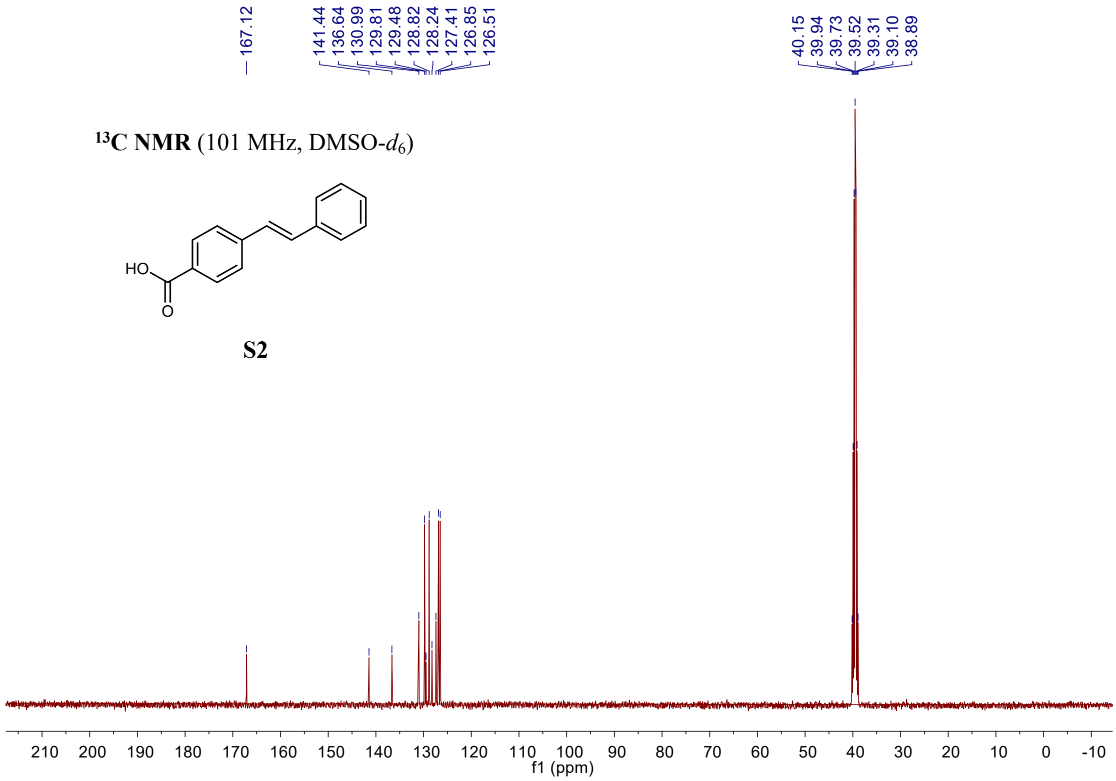

167.12
141.44
136.64
130.99
129.81
129.48
128.82
128.24
127.41
126.85
126.51
40.15
39.94
39.73
39.52
39.31
39.10
38.89
13C NMR (101 MHz, DMSO-d6)
HO
O
S2
210 200 190 180 170 160 150 140 130 120 110 100 90 80 70 60 50 40 30 20 10 0 -10
f1 (ppm)

4.5.3 Validation of on-DNA Heck coupling product 5a by co-injection assay

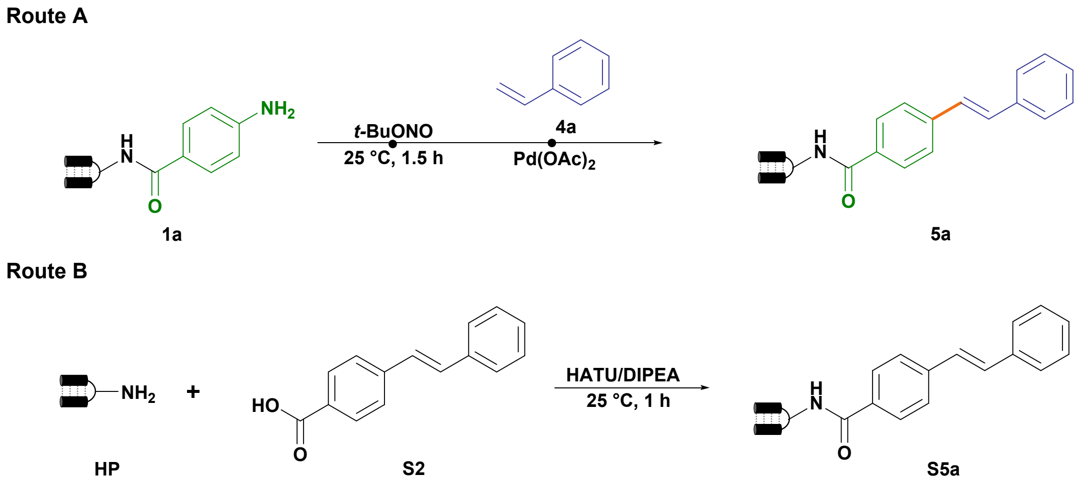

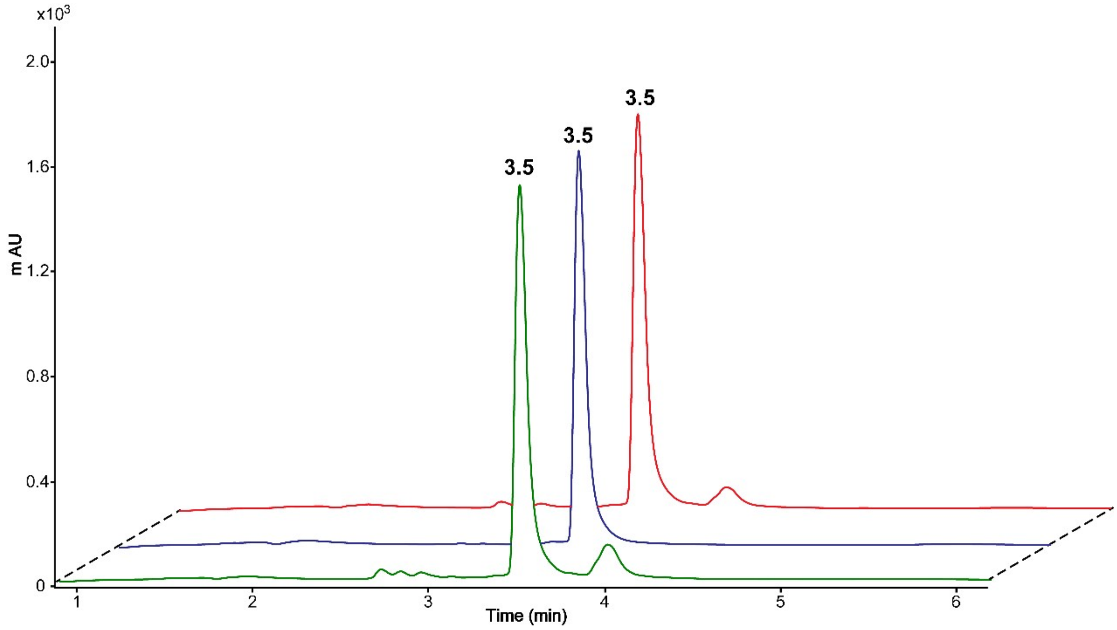

Supplementary Fig. 6 The co-injection experiment of 5a and S5a by UPLC-MS. The “red” curve is the co-injection sample mixing **5a** and **S5a**; The “green” curve is **5a**; The “blue” curve is **S5a**.

The on-DNA synthesized **5a** sample was co-eluted with the off-DNA synthesized and characterized **S5a** sample, indicating their structures were identical.

4.5.4 Validation of On-DNA Hiyama coupling product 10a by co-injection assay

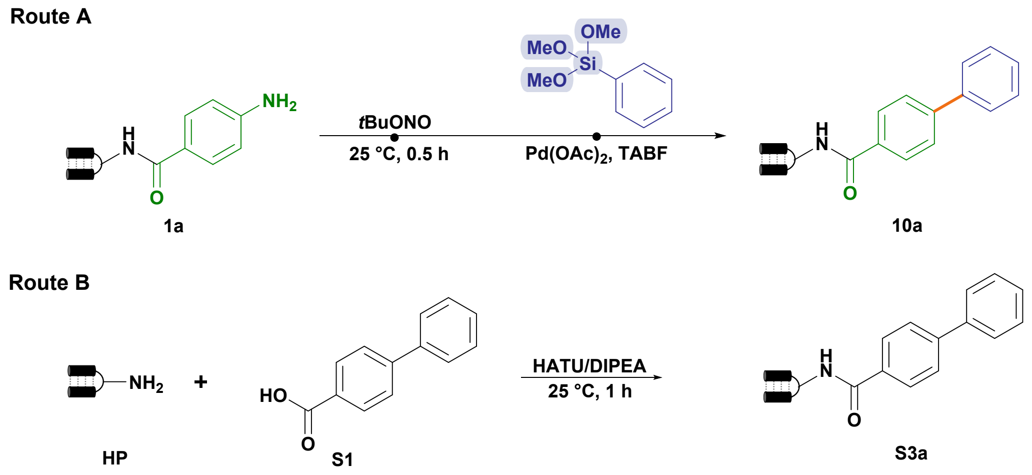

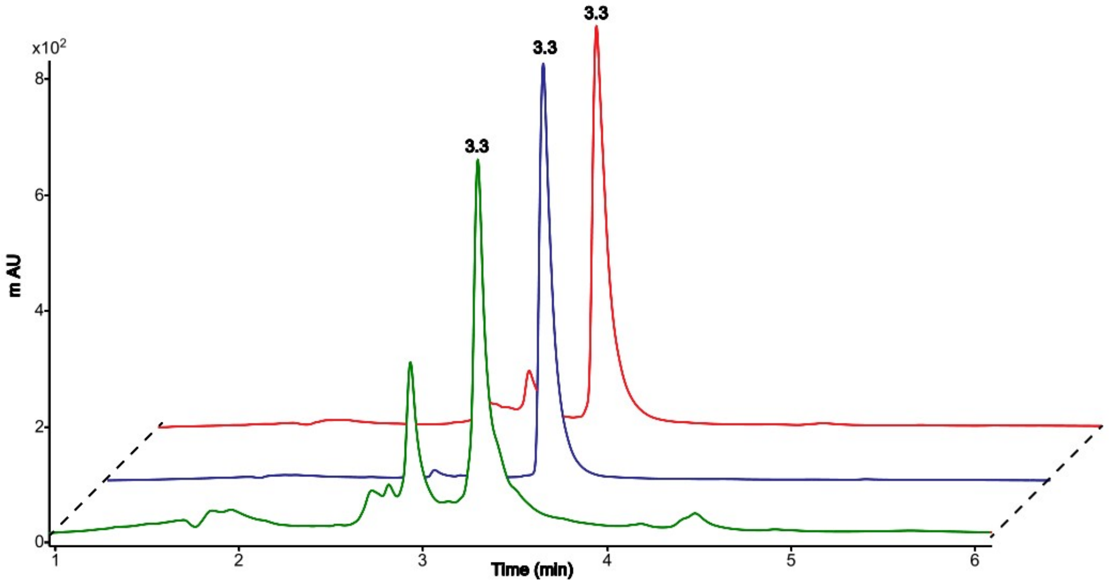

Supplementary Fig. 7 The co-injection experiment of 10a and S3a by UPLC-MS. The “red” curve is the co-injection sample mixing **10a** and **S3a**; The “green” curve is **10a**; The “blue” curve is **S3a**.

The on-DNA synthesized **10a** sample was co-eluted with the off-DNA synthesized and characterized **S3a** sample, indicating their structures were identical.

5. Substrate scopes of on-DNA Suzuki-Miyaura, Heck reaction and Hiyama reaction

5.1 Substrate scope of boronic acids for the on-DNA Suzuki-Miyaura coupling

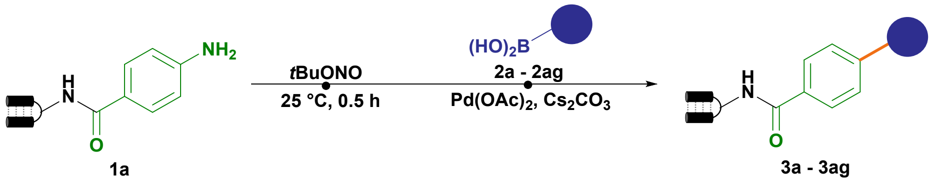

Supplementary Table 3. Substrate scope of boronic acids

compound	structure	product	calculated mass [Da]	found mass [Da]	conversion [%]
**2a**	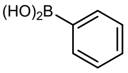	**3a**	5117	5117	73
**2b**	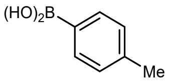	**3b**	5131	5131	62
**2c**	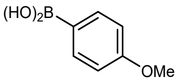	**3c**	5147	5147	77
**2d**	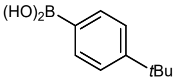	**3d**	5173	5173	63
**2e**	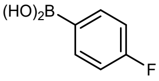	**3e**	5135	5135	91
**2f**	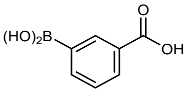	**3f**	5161	5161	65
**2g**	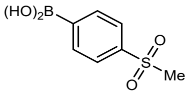	**3g**	5195	5195	75
**2h**	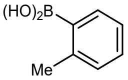	**3h**	5131	5131	70
**2i**	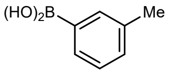	**3i**	5131	5131	68
**2j**	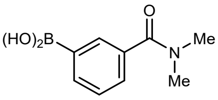	**3j**	5188	5188	82
**2k**	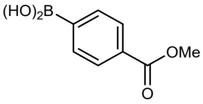	**3k**	5175	5175	78

**2l**	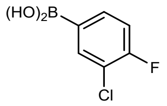	**3l**	5169	5169	63
**2m**	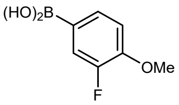	**3m**	5165	5165	85
**2n**	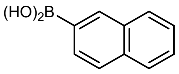	**3n**	5167	5167	83
**2o**	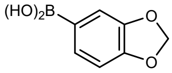	**3o**	5161	5161	85
**2p**	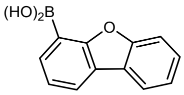	**3p**	5207	5207	80
**2q**	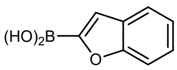	**3q**	5157	5157	82
**2r**	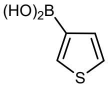	**3r**	5123	5123	77
**2s**	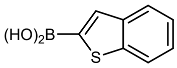	**3s**	5173	5173	60
**2t**	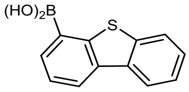	**3t**	5223	5223	78
**2u**	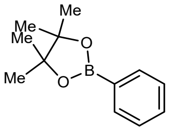	**3u**	5117	5117	83
**2v**	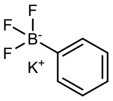	**3v**	5117	5117	70
**2w**	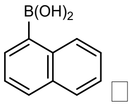	**3w**	5167	5167	83
**2x**	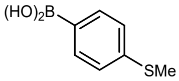	**3x**	5163	5163	50
**2y**	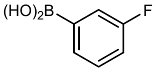	**3y**	5135	5135	79
**2z**	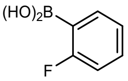	**3z**	5135	5135	66
**2aa**	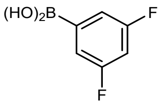	**3aa**	5153	5153	86

**2ab**	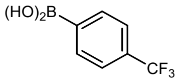	**3ab**	5185	5185	80
**2ac**	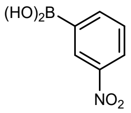	**3ac**	5162	5162	59
**2ad**	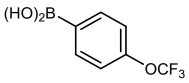	**3ad**	5201	5201	65
**2ae**	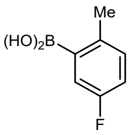	**3ae**	5149	5149	57
**2af**	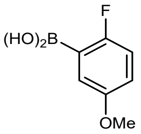	**3af**	5165	5165	67
**2ag**	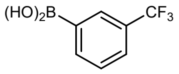	**3ag**	5185	5185	75

5.2 Substrate scope of DNA-conjugated arylamines for the on-DNA Suzuki-Miyaura coupling

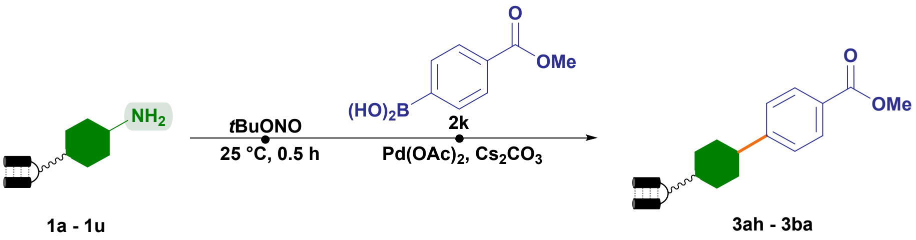

Supplementary Table 4. Substrate scope of DNA-conjugated arylamines

compound	structure	product	calculated mass [Da]	found mass [Da]	conversion [%]
**1a**	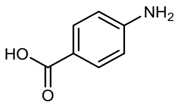	**3k**	5175	5175	78
**1b**	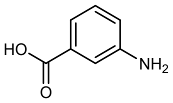	**3ah**	5175	5175	79
**1c**	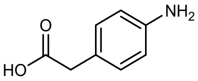	**3ai**	5189	5189	73
**1d**	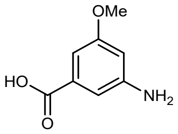	**3aj**	5205	5205	67
**1e**	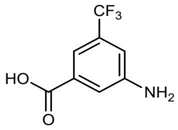	**3ak**	5243	5243	62
**1f**	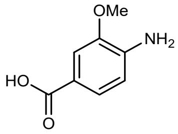	**3al**	5205	5205	80
**1g**	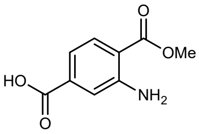	**3am**	5233	5233	66
**1h**	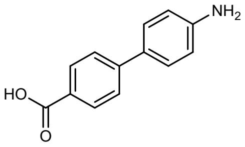	**3an**	5251	5251	70
**1i**	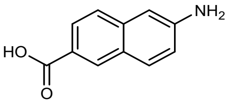	**3ao**	5225	5225	66

**1j**	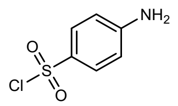	**3ap**	5211	5211	67
**1k**	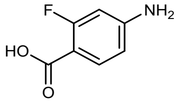	**3aq**	5193	5193	85
**1l**	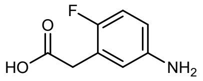	**3ar**	5207	5207	72
**1m**	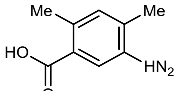	**3as**	5203	5203	56
**1n**	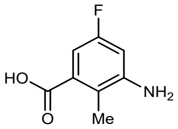	**3at**	5207	5207	51
**1o**	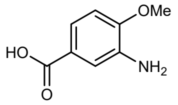	**3au**	5205	5205	61
**1p**	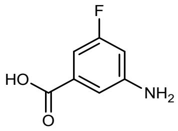	**3av**	5193	5193	65
**1q**	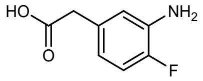	**3aw**	5207	5207	54
**1r**	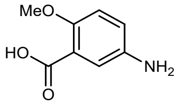	**3ax**	5205	5205	67
**1s**	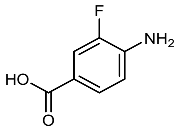	**3ay**	5193	5193	56
**1t**	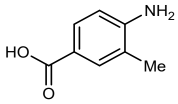	**3az**	5189	5189	79
**1u**	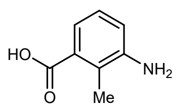	**3ba**	5189	5189	51
**1v**	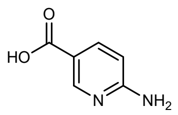	**3bb**	5118	5057 (starting material only)	0

5.3 Substrate scope of olefins for the on-DNA Heck reaction

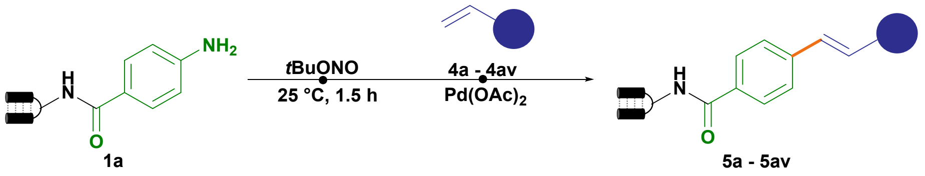

Supplementary Table 5. Substrate scope of olefins for the on-DNA Heck reaction

compound	structure	product	calculated mass [Da]	found mass [Da]	conversion [%]
**4a**	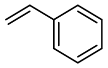	**5a**	5143	5143	83
**4b**	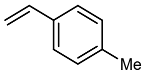	**5b**	5157	5157	84
**4c**	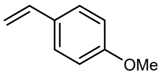	**5c**	5173	5173	78
**4d**	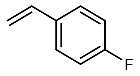	**5d**	5161	5161	83
**4e**	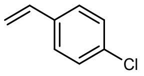	**5e**	5177	5177	83
**4f**	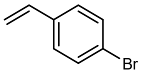	**5f**	5222	5222	84
**4g**	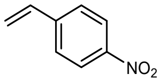	**5g**	5188	5188	80
**4h**	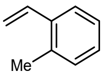	**5h**	5157	5157	91
**4i**	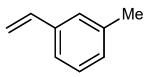	**5i**	5157	5157	87
**4j**	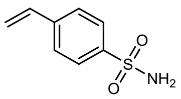	**5j**	5222	5222	91
**4k**	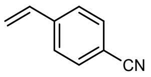	**5k**	5168	5168	83
**4l**	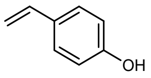	**5l**	5159	5159	63

**4m**	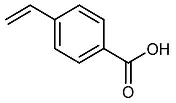	**5m**	5187	5187	81
**4n**	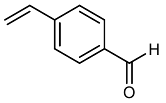	**5n**	5171	5171	84
**4o**	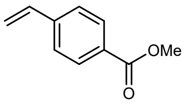	**5o**	5201	5201	84
**4p**	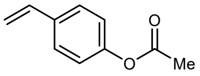	**5p**	5201	5201	80
**4q**	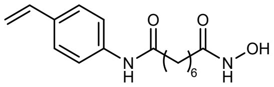	**5q**	5329	5329	85
**4r**	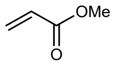	**5r**	5125	5125	94
**4s**	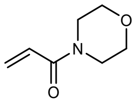	**5s**	5180	5180	87
**4t**	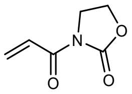	**5t**	5180	5180	89
**4u**	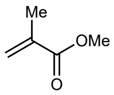	**5u**	5139	5139	82
**4v**	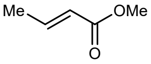	**5v**	5139	5139	77
**4w**	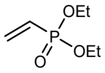	**5w**	5203	5203	88
**4x**	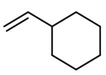	**5x**	5149	5149	86
**4y**	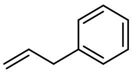	**5y**	5157	5157	90
**4z**	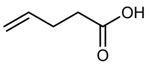	**5z**	5139	5139	88
**4aa**	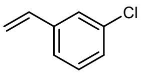	**5aa**	5177	5177	85

**4ab**	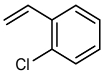	**5ab**	5177	5177	91
**4ac**	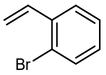	**5ac**	5222	5222	70
**4ad**	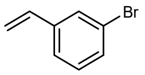	**5ad**	5222	5222	83
**4ae**	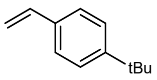	**5ae**	5199	5199	75
**4af**	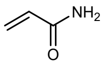	**5af**	5110	5110	89
**4ag**	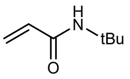	**5ag**	5166	5166	90
**4ah**	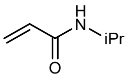	**5ah**	5152	5152	85
**4ai**	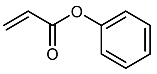	**5ai**	5187	5187	87
**4aj**	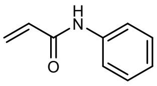	**5aj**	5186	5186	87
**4ak**	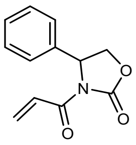	**5ak**	5256	5256	80
**4al**	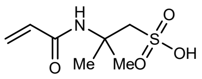	**5al**	5246	5246	83
**4am**	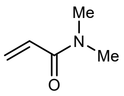	**5am**	5138	5138	87
**4an**	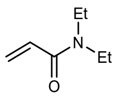	**5an**	5166	5166	87
**4ao**	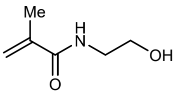	**5ao**	5169	5169	86
**4ap**	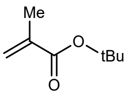	**5ap**	5181	5181	68

**4aq**	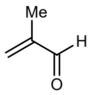	**5aq**	5109	5109	62
**4ar**	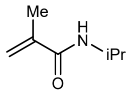	**5ar**	5166	5166	82
**4as**	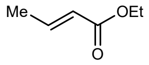	**5as**	5153	5153	58
**4at**	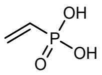	**5at**	5147	5147	84
**4au**	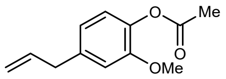	**5au**	5245	5245	82
**4av**	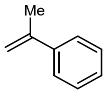	**5av**	5157	5157	78

5.4 Substrate scope of DNA-conjugated arylamines for the on-DNA Heck reaction

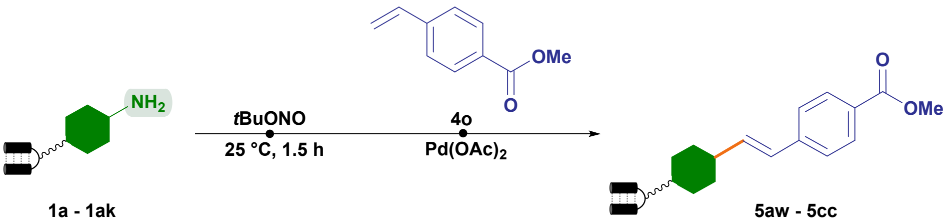

1a - 1ak   5aw - 5cc

Supplementary Table 6. Substrate scope of DNA-conjugated arylamines for Heck reaction with styrene

compound	structure	product	calculated mass [Da]	found mass [Da]	conversion [%]
**1a**	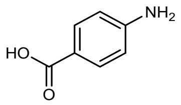	**5o**	5201	5201	86
**1b**	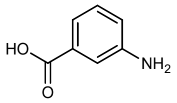	**5aw**	5201	5201	90
**1c**	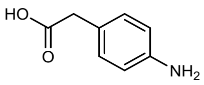	**5ax**	5215	5215	77
**1d**	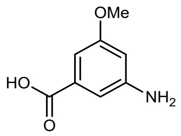	**5ay**	5231	5231	70
**1e**	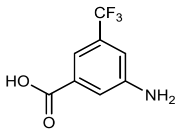	**5az**	5269	5269	86
**1f**	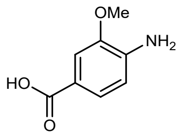	**5ba**	5231	5231	92
**1g**	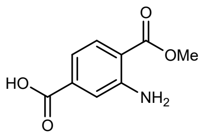	**5bb**	5259	5259	76
**1h**	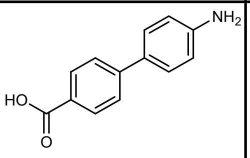	**5bc**	5277	5277	92

**1i**	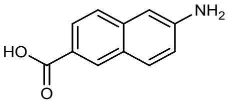	**5bd**	5251	5251	81
**1j**	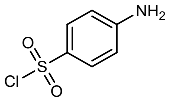	**5be**	5237	5237	83
**1k**	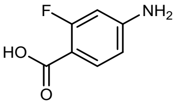	**5bf**	5219	5219	86
**1l**	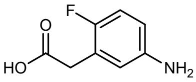	**5bg**	5233	5233	89
**1m**	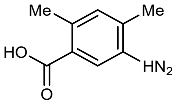	**5bh**	5229	5229	96
**1n**	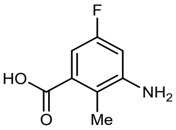	**5bi**	5233	5233	84
**1o**	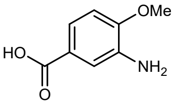	**5bj**	5231	5231	82
**1p**	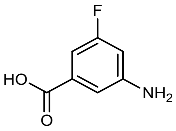	**5bk**	5219	5219	64
**1q**	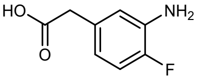	**5bl**	5233	5233	84
**1r**	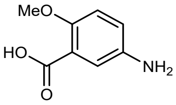	**5bm**	5231	5231	90
**1s**	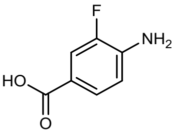	**5bn**	5219	5219	50
**1t**	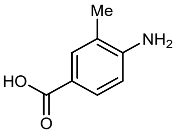	**5bo**	5215	5215	87
**1u**	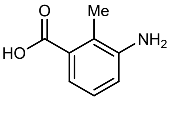	**5bp**	5215	5215	84

**1v**	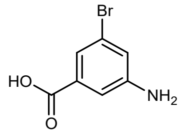	**5bq**	5280	5280	90
**1w**	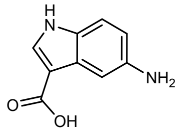	**5br**	5240	5240	82
**1x**	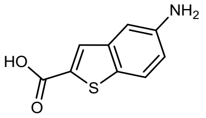	**5bs**	5257	5257	70
**1y**	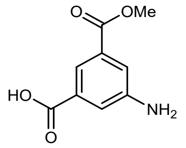	**5bt**	5245	5245	73
**1z**	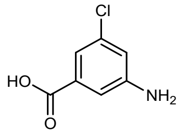	**5bu**	5236	5236	81
**1aa**	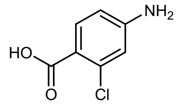	**5bv**	5236	5236	84
**1ab**	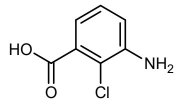	**5bw**	5236	5236	88
**1ac**	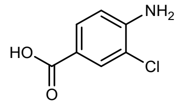	**5bx**	5236	5236	82
**1ad**	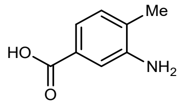	**5by**	5215	5215	62
**1ae**	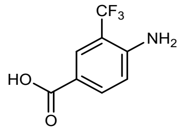	**5bz**	5268	5268	80
**1af**	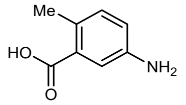	**5ca**	5214	5214	81
**1ag**	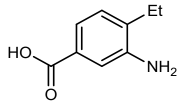	**5cb**	5229	5229	43
**1ah**	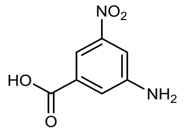	**5cc**	5246	5246	67

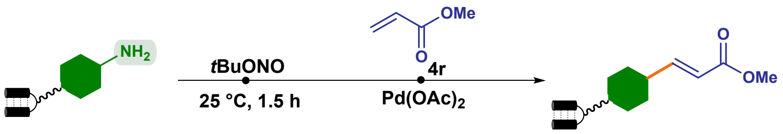

Supplementary Table 7. Substrate scope of DNA-conjugated arylamines for Heck reaction with methyl acrylate

compound	structure	product	calculated mass [Da]	found mass [Da]	conversion [%]
**1a**	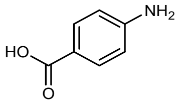	**5r**	5125	5125	86
**1b**	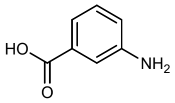	**5cd**	5125	5125	84
**1c**	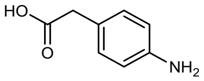	**5ce**	5139	5139	92
**1d**	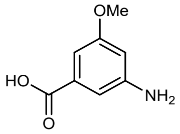	**5cf**	5155	5155	80
**1e**	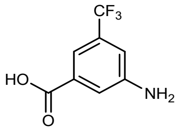	**5cg**	5193	5193	72
**1f**	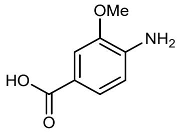	**5ch**	5155	5155	92
**1g**	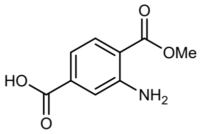	**5ci**	5183	5183	89
**1h**	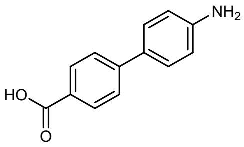	**5cj**	5201	5201	95
**1i**	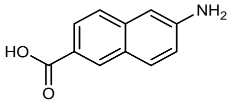	**5ck**	5175	5175	87

**1j**	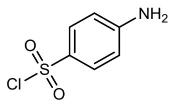	**5cl**	5161	5161	86
**1k**	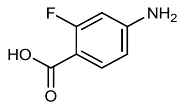	**5cm**	5143	5143	86
**1l**	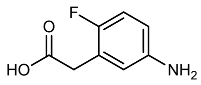	**5cn**	5157	5157	88
**1m**	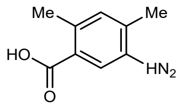	**5co**	5153	5153	93
**1n**	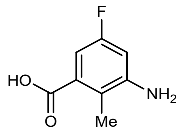	**5cp**	5157	5157	88
**1o**	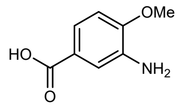	**5cq**	5155	5155	94
**1p**	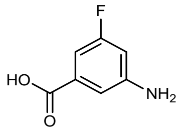	**5cr**	5143	5143	75
**1q**	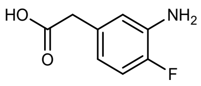	**5cs**	5157	5157	85
**1r**	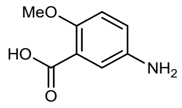	**5ct**	5155	5155	87
**1s**	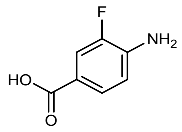	**5cu**	5143	5143	75
**1t**	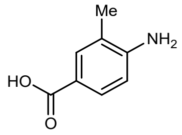	**5cv**	5139	5139	92
**1u**	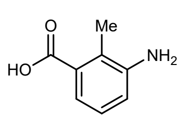	**5cw**	5139	5139	85
**1v**	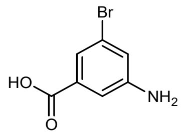	**5cx**	5204	5204	91

**1w**	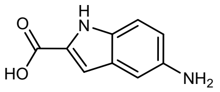	**5cy**	5164	5164	50
**1x**	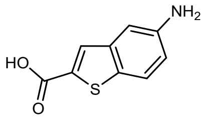	**5cz**	5181	5181	75
**1y**	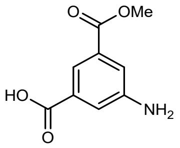	**5da**	5169	5169	79
**1z**	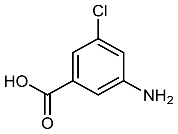	**5db**	5160	5160	89
**1aa**	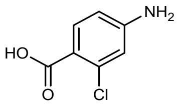	**5dc**	5160	5160	90
**1ab**	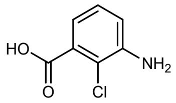	**5dd**	5160	5160	92
**1ac**	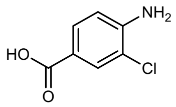	**5de**	5160	5160	90
**1ad**	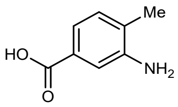	**5df**	5139	5139	63
**1ae**	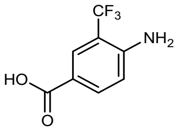	**5dg**	5194	5194	73
**1af**	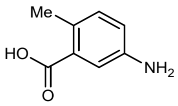	**5dh**	5139	5139	92
**1ag**	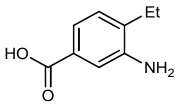	**5di**	5153	5153	67
**1ah**	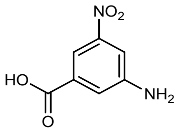	**5dj**	5170	5170	76

5.5 Substrate scope of arylamines for the reverse on-DNA Heck reaction

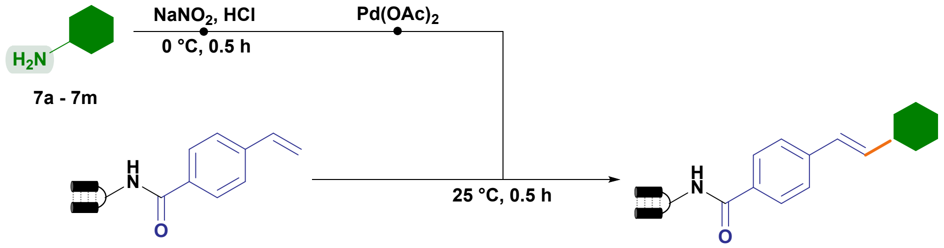

Supplementary Table 8. Substrate scope of arylamines for the reverse on-DNA Heck reaction

compound	structure	product	calculated mass [Da]	found mass [Da]	conversion [%]
**7a**	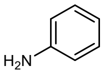	**5a**	5143	5143	80
**7b**	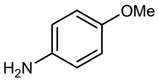	**5c**	5173	5173	77
**7c**	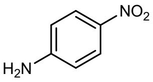	**5g**	5188	5188	89
**7d**	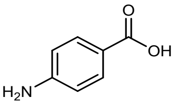	**5m**	5187	5187	57
**7e**	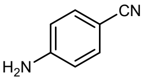	**5k**	5168	5168	55
**7f**	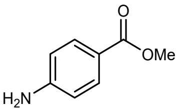	**5o**	5201	5201	85
**7g**	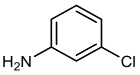	**5aa**	5177	5177	72
**7h**	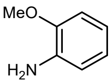	**5di**	5173	5173	70
**7i**	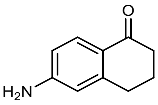	**5dj**	5211	5211	89
**7j**	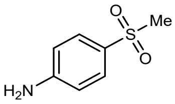	**5dk**	5221	5221	77

**7k**	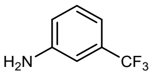	**5dl**	5211	5211	64
**7l**	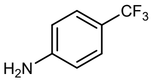	**5dm**	5211	5211	68
**7m**	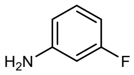	**5dn**	5161	5161	76

5.6 Substrate scope of DNA-conjugated arylamines for the on-DNA Hiyama reaction

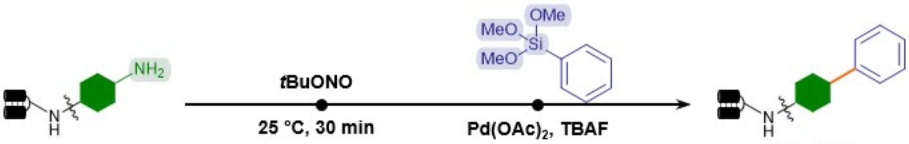

10a – 10f

Supplementary Table 9. Substrate scope of arylamines for the on-DNA Hiyama reaction

compound	structure	product	calculated mass [Da]	found mass [Da]	conversion [%]
**1a**	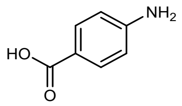	**10a**	5117	5117	67
**1b**	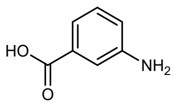	**10b**	5117	5117	61
**1c**	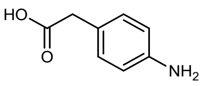	**10c**	5131	5131	66
**1l**	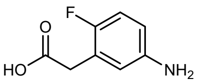	**10d**	5149	5149	63
**1x**	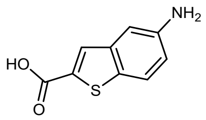	**10e**	5173	5173	57
**1r**	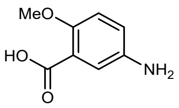	**10f**	5147	5147	51

*Note that partial DNA damage was found in the Hiyama reaction condition. The DNA damage type was mainly depurination (-117 Da), and consist a 10-17% proportion in the desired final products. Please see the supplementary spectrum data for details.

5.7 Scale-up reactions

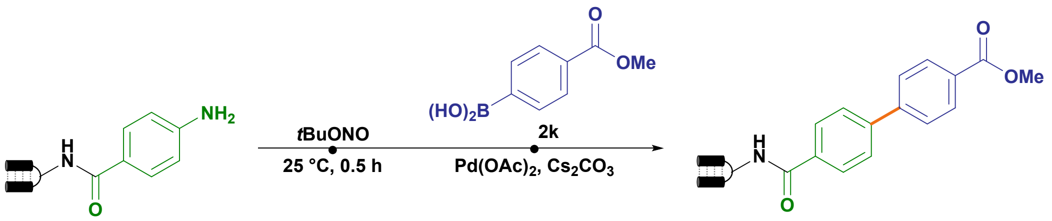

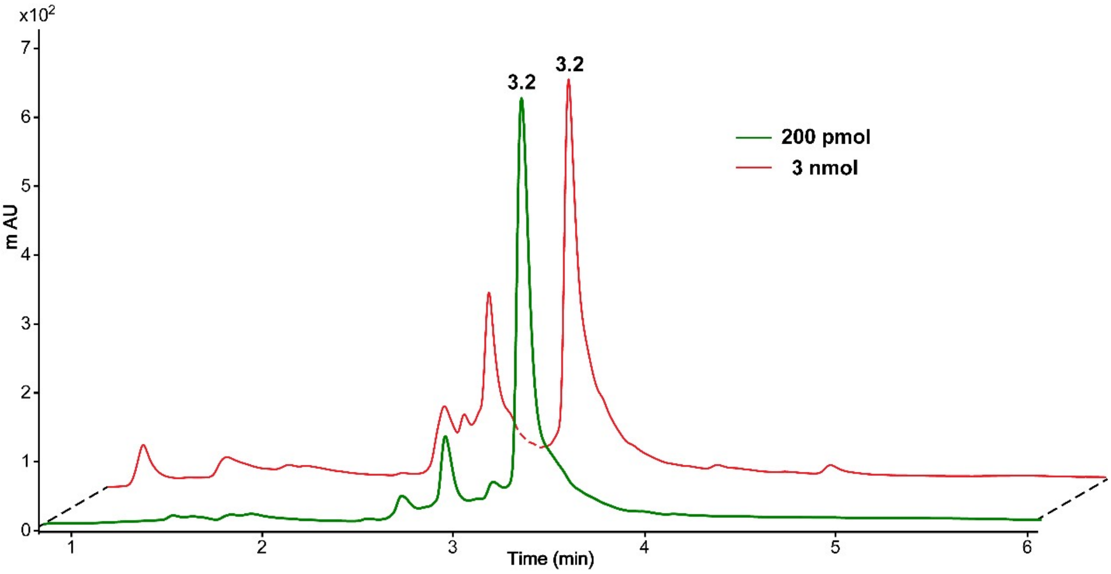

Supplementary Fig. 8 UPLC chromatograph of 3k at 200 pmol and 3 nmol scale

On-DNA aryl diazonium salt formation: To the DNA conjugate **1a** (16 μL, 187 μM in H_2O, 3 nmol) was added *t*BuONO (4 μL, 50 mM in DMA, 200 nmol) and H_2O (20 μL). Then the resulting mixture was vortexed and stood at 25 °C for 0.5 h.

On-DNA Suzuki-Miyaura Coupling: After the aryl diazonium intermediate was generated according to the above method, $Pd(OAc)_2$ (4 μL, 20 mM in DMA, 80 nmol) and boronic acid (4 μL, 500 mM in DMA, 2000 nmol) were added. The mixture was vortexed and finally supplemented with Cs_2CO_3 (4 μL, 800 mM in H_2O, 3200 nmol) and 8 μL H_2O, and allowed for reaction at 40 °C for 2 h. After reaction, 30 equiv. of sodium diethyldithiocarbamic acid (compared with $Pd(OAc)_2$ were added to the mixture to scavenge Pd, and the reaction mixture was stood at 25 °C for 30 minutes. The mixture was centrifuged at 25 °C for 10 min at 13,500 rpm, and the resultant supernatant was collected. The product was obtained by ethanol precipitation as described above and characterized by UPLC-MS. During the reaction, attention must

be paid to: the sequence of adding reagents should not be reversed.

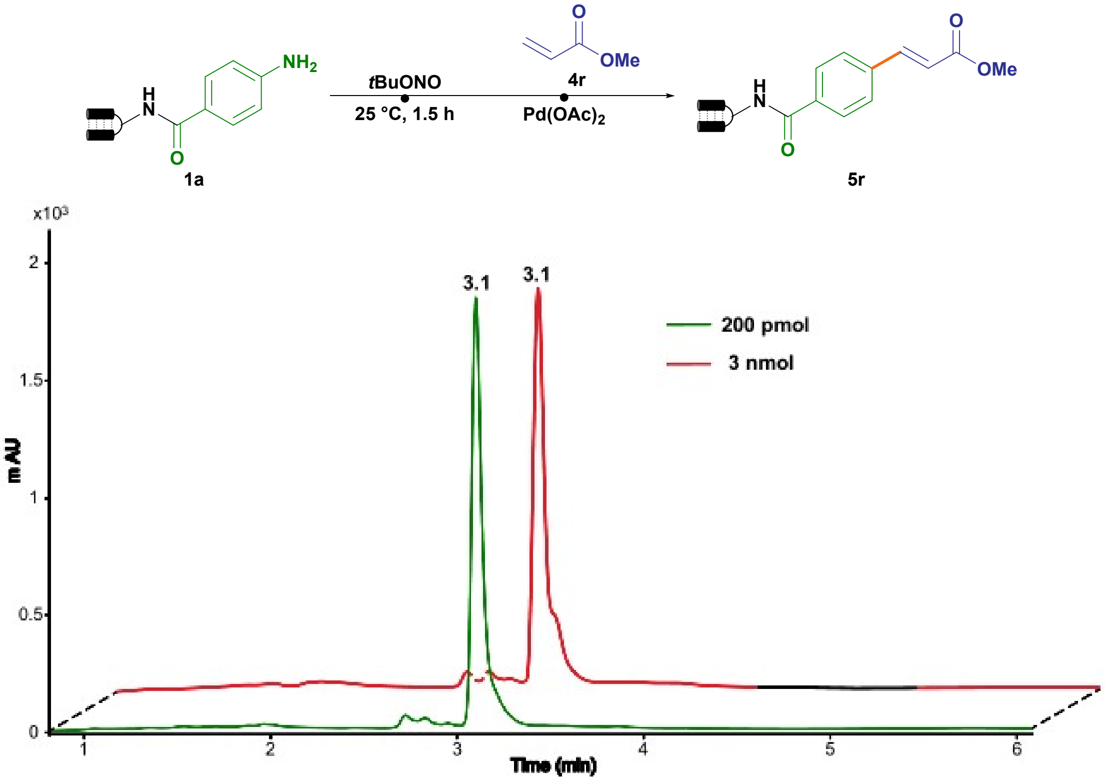

Supplementary Fig. 9 UPLC chromatograph of 3r at 200 pmol and 3 nmol scale

On-DNA aryl diazonium salt formation: To the DNA conjugate **1a** (8 μL, 375 μM in H_2O, 3 nmol) was added *t*BuONO (2 μL, 50 mM in DMA, 100 nmol) and H_2O (10 μL). Then the resulting mixture was vortexed and stood at 25 °C for 1.5 h.

On-DNA Heck reaction: After the aryl diazonium intermediate was generated according to the above method, $Pd(OAc)_2$ (2 μL, 20 mM in DMA, 40 nmol) were added. The mixture was vortexed and added a mixed solution of PB (3 μL, 200 mM in H_2O, pH 5.5, 600 nmol), H_2O (3 μL) and styrene (2 μL, 500 mM in DMA, 1000 nmol), and allowed for reaction at 25 °C for 30 min. After reaction, 30 equiv. of scavenger sodium diethyldithiocarbamic acid (compared with $Pd(OAc)_2$) was added to the mixture, and the reaction mixture was stood at 25 °C for 30 minutes. The mixture was centrifuged at 25 °C for 10 min at 13,500 rpm, and the resultant supernatant was collected. The product was obtained by ethanol precipitation as described above and characterized by UPLC-MS.

6. Application of on-DNA carbon-carbon bond formation in DEL construction

6.1 on-DNA branched molecule synthesis

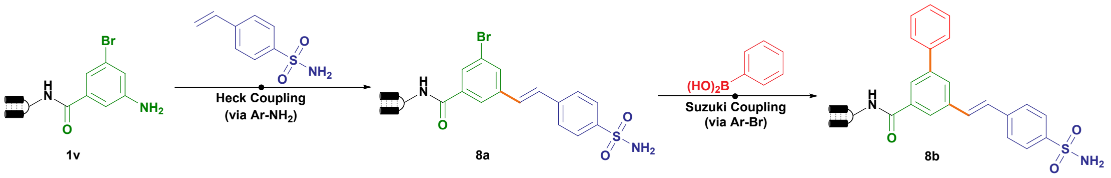

8a: A solution of **1f** in H_2O (8 μL, 250 μM, 2 nmol) was added with *t*BuONO (2 μL, 50 mM in H_2O, 100 nmol) and H_2O (10 μL). Then the resulting mixture was vortexed and stood at 25 °C for 1.5 h.

After the aryl diazonium salt was generated according to the above method, $Pd(OAc)_2$ (2 μL, 20 mM in DMA, 40 nmol) was added. The mixture was vortexed and added a mixed solution of PB (3 μL, 200 mM in H_2O, pH 5.5, 600 nmol), H_2O (3 μL) and 4-vinylbenzenesulfonamide (2 μL, 500 mM in DMA, 1000 nmol), and allowed for reaction at 25 °C for 30 min. After reaction, 30 equiv. of scavenger sodium diethyldithiocarbamic acid (compared with $Pd(OAc)_2$) was added to the mixture, and the reaction mixture was stood at 25 °C for 30 minutes. The mixture was centrifuged at 25 °C for 10 min at 13,500 rpm, and the resultant supernatant was collected. The product **8a** was obtained by ethanol precipitation as described above and characterized by UPLC-MS.

8b: To the DNA conjugate **8a** (8 μL, 250 μM in H_2O, 2 nmol) was added phenylboronic acid (1 μL, 500 mM in DMA, 500 nmol) and Cs_2CO_3 (1 μL, 800 mM in H_2O, 800 nmol), then the pre-mixture of ligand ([[1,1'-Biphenyl]-3-sulfonic acid, 2'-(dicyclohexylphosphino)-2,6-dimethoxy-, sodium salt, hydrate, CAS: 1049726-96-6) (2 μL, 20 mM in H_2O, 40 nmol) and $Pd(OAc)_2$ (1 μL, 10 mM in MeCN, 10 nmol) was added. The reaction was allowed to proceed at 80 °C for 3.5 h. After reaction, 30 equiv. of scavenger sodium diethyldithiocarbamic acid (compared with $Pd(OAc)_2$) were added to the mixture, and the reaction mixture was stood at 25 °C for 30 min. The mixture

was centrifuged at 25 °C for 10 min at 13,500 rpm, and the resultant supernatant was collected. The product **8b** was obtained by ethanol precipitation as described above and characterized by UPLC-MS.[3]

6.2 on-DNA linear molecule synthesis

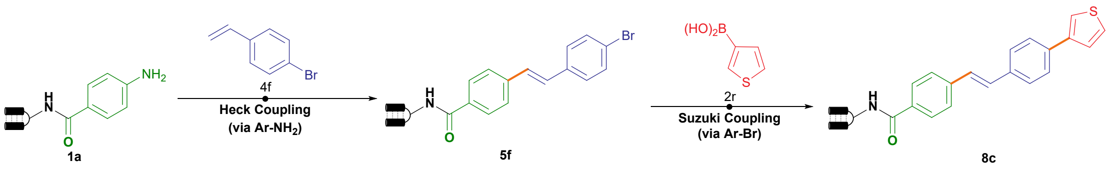

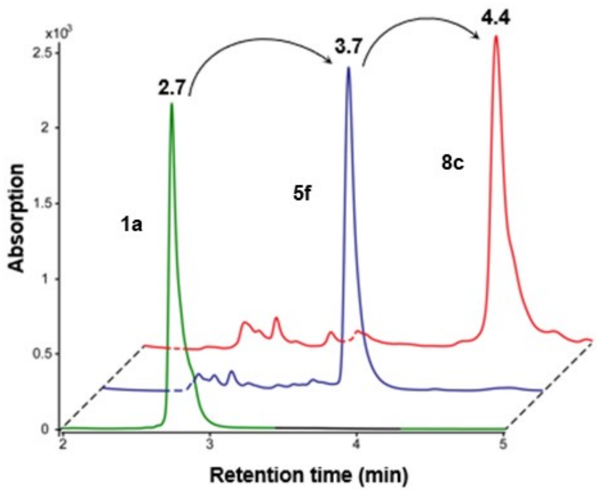

Supplementary Fig. 10 Synthesis of on-DNA linear molecule 8c

5f: To the DNA conjugate **1a** (8 µL, 0.25 mM in H_2O, 2 nmol) was added with *t*BuONO (2 µL, 50 mM in H_2O, 100 nmol) and H_2O (10 µL). Then the resulting mixture was vortexed and stood at 25 °C for 1.5 h.

After the aryl diazonium salt was generated according to the above method, $Pd(OAc)_2$ (2 µL, 20 mM in DMA, 40 nmol) was added. The mixture was vortexed and added a mixed solution of PB (3 µL, 200 mM in H_2O, pH 5.5, 600 nmol), H_2O (3 µL) and 4-Bromostyrene (2 µL, 500 mM in DMA, 1000 nmol), and allowed for reaction at 25 °C for 30 min. After reaction, 30 equiv. of scavenger sodium diethyldithiocarbamic acid (compared with $Pd(OAc)_2$) was added to the mixture, and the reaction mixture was stood at 25 °C for 30 minutes. The mixture was centrifuged at 25 °C for 10 min at 13,500 rpm, and the resultant supernatant was collected. The product **5f** was obtained

by ethanol precipitation as described above and characterized by UPLC-MS.

8c: To the DNA conjugate **5f** (8 μL, 250 μM in H_2O, 2 nmol) was added the boronate (1 μL, 500 mM in DMA, 500 nmol) and Cs_2CO_3 (1 μL, 800 mM in H_2O, 800 nmol), then the pre-mixture of ligand 1 (2 μL, 20 mM in H_2O, 40 nmol) and $Pd(OAc)_2$ (1 μL, 10 mM in MeCN, 10 nmol) was added. The reaction was allowed to proceed at 80 °C for 3.5 h. After reaction, 30 equiv. of scavenger sodium diethyldithiocarbamic acid (compared with $Pd(OAc)_2$) were added to the mixture, and the reaction mixture was stood at 25 °C for 30 min. The mixture was centrifuged at 25 °C for 10 min at 13,500 rpm, and the resultant supernatant was collected. The product **8c** was obtained by ethanol precipitation as described above and characterized by UPLC-MS.

6.2 Verification of DNA enzymatic ligation compatibility

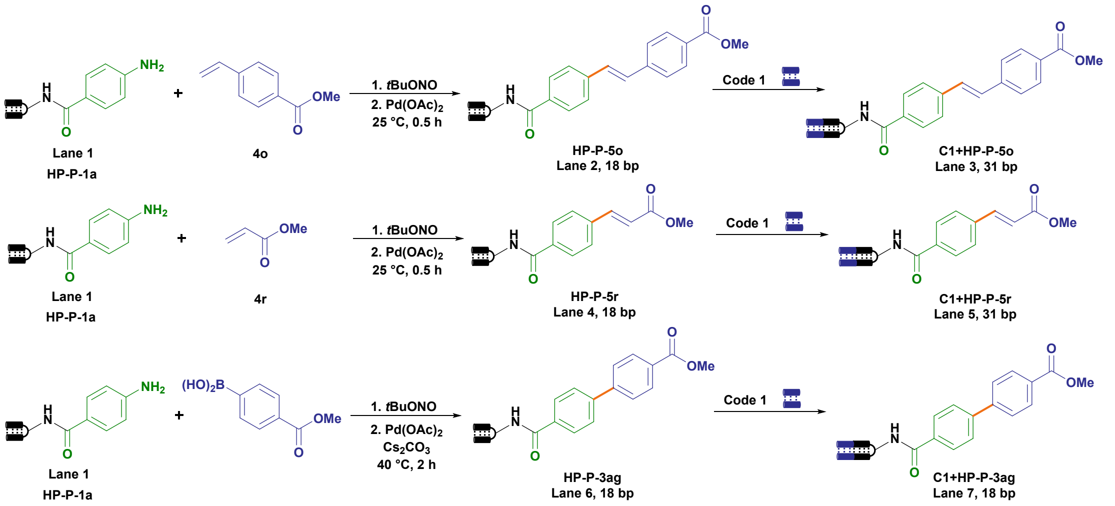

Supplementary Fig. 11 Protocol of on-DNA C-C bond formation and subsequent enzymatic ligation.

HP-P-1a, **HP-P-5o**, **HP-P-5r**, and **HP-P-3ag** were synthesized according to the above method. (See chapters **4.1** and **4.2** for details)

To the DNA conjugate **HP-P-5o**, **HP-P-5r**, or **HP-P-3ag** (5 μL, 0.5 nmol), was added with code 1 (6 μL, 0.6 nmol, 1.2 equiv.) and 10× ligation buffer (2 μL) in the 0.6 mL tube and thoroughly mixed by vortex, then T4 DNA ligase (1 μL, 350 units/μL) and H_2O (6 μL) were added and mixed gently. The reaction mixture was incubated at 20 °C overnight. After ligation confirmation by UPLC-MS analysis, the reaction system

was denatured by boiling at 95 °C for 10 min, and the ligation product was isolated by ethanol precipitation. The resulting pellets were vacuum-dried and dissolved in nuclease-free water, and subjected to PAGE analysis.

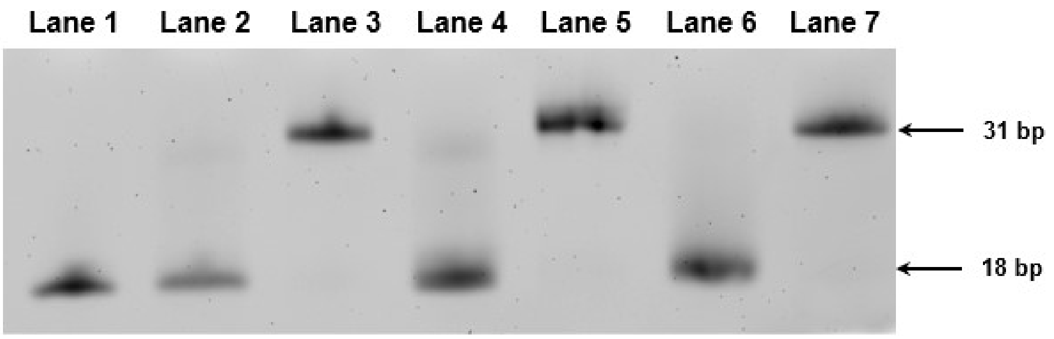

Supplementary Fig. 12 20% denatured PAGE analysis of DEL-encoding compatibility of on-DNA C-C bond formation.

6.3 Verification of PCR and sequencing compatibility of DNA barcodes

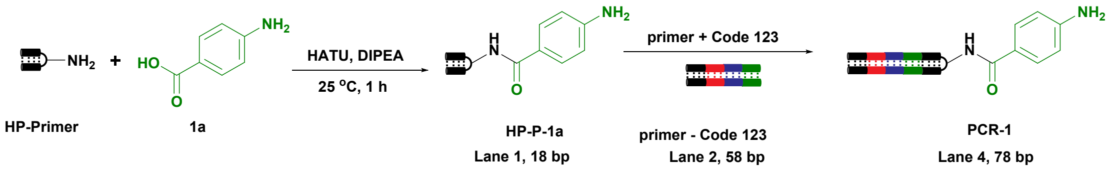

Supplementary Fig. 13 Scheme of preparing the amplifiable PCR substrate PCR-1.

Pre-mixed solution: 4-aminobenzoic acid (4 μL, 200 mM in DMA), HATU (4 μL, 200 mM in DMA), and DIPEA (4 μL, 200 mM in DMA).

A solution of **HP-Primer** in pH 9.4 borate buffer (10 μL, 1 mM in 250 mM sodium borate buffer) was added 6 μL pre-mixed solution. The mixture was vortexed and reacted at room temperature for 10 min, then was added another 4 μL of pre-mixed solution. The mixture was vortexed and reacted at room temperature for 1 h, followed by ethanol precipitation. The resulting pellet was vacuum-dried and dissolved in nuclease-free water (200 μL). The reaction was analyzed by UPLC-MS.

DNA conjugate **HP-P-1a** (10 μL, 500 pmol), code 123 (5 μL, 500 pmol, 1.0 equiv.) and 10× ligation buffer (2 μL) were mixed in the 0.6 mL tube, then T4 DNA ligase (1 μL, 350 units/μL) and H_2O (2 μL) was added and mixed gently. The reaction

mixture was vortexed, centrifuged, and incubated at 20 °C for overnight. After ligation confirmation by UPLC-MS analysis, the reaction system was denatured by boiling at 95 °C for 10 min, and the ligation product was isolated by ethanol precipitation. The resulting pellets were vacuum-dried and dissolved in nuclease-free water. The resulting enzymatic ligation product **PCR-1** was checked by PAGE analysis.

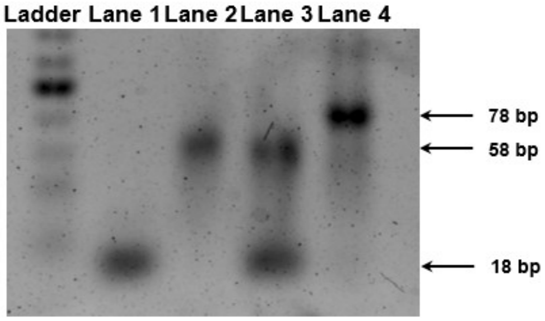

Supplementary Fig. 14 3% Agarose gel analysis of the amplifiable PCR substrate PCR-1. Lane 1: HP-P-1a, **Lane 2**: Code 123-Primer, **Lane 3**: HP-P/Code, without T4 DNA ligase, **Lane 4**: HP-P/Code, with T4 DNA ligase .

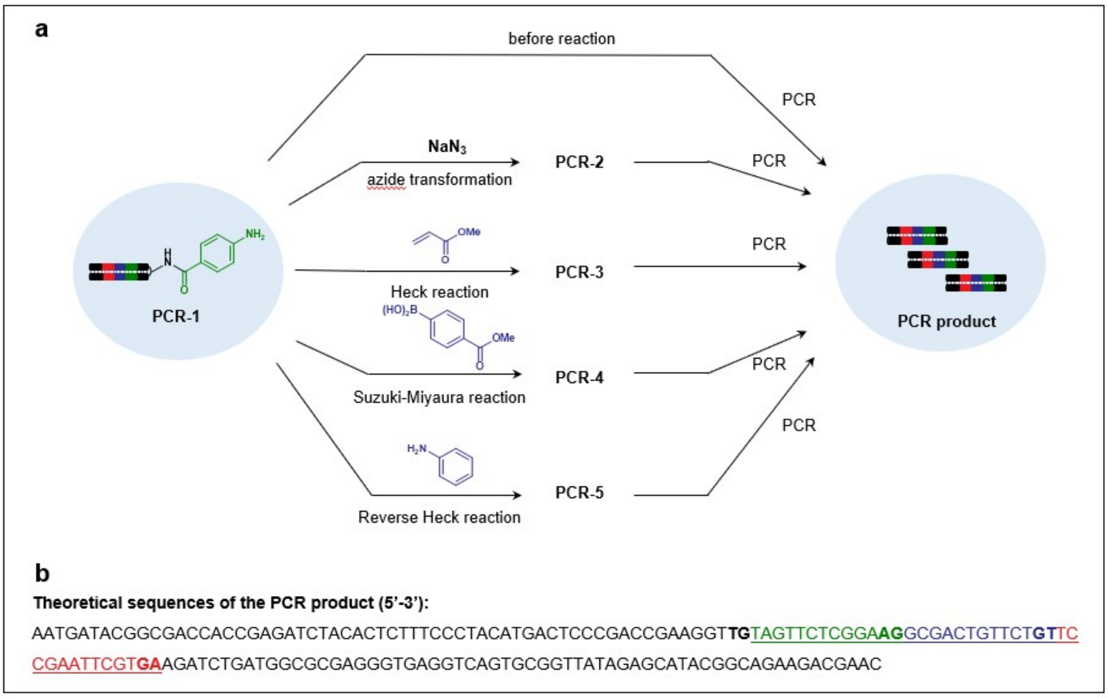

Supplementary Fig. 15 Scheme of sample preparation for PCR and Sanger sequencing

The **PCR-1** amplifiable DNA substrate was subjected to various chemical reactions developed in this work. As representatives, **PCR-2**, **PCR-3**, **PCR-4**, and

PCR-5 were synthesized according to the above method. (See chapters **3.2**, **4.1**, **4.2** for details)

After **PCR-1**, **PCR-2**, **PCR-3**, **PCR-4,** and **PCR-5** were obtained, the samples were subjected to PCR amplification and Sanger sequencing. By comparing the sequencing results of the starting material before reaction (**PCR-1**) with the chemical reaction products (**PCR-2**, **PCR-3**, **PCR-4**, and **PCR-5**), we could reach the conclusion that the method we developed here was compatible with DNA PCR and sequencing, that is, compatible with the DEL decoding procedure.

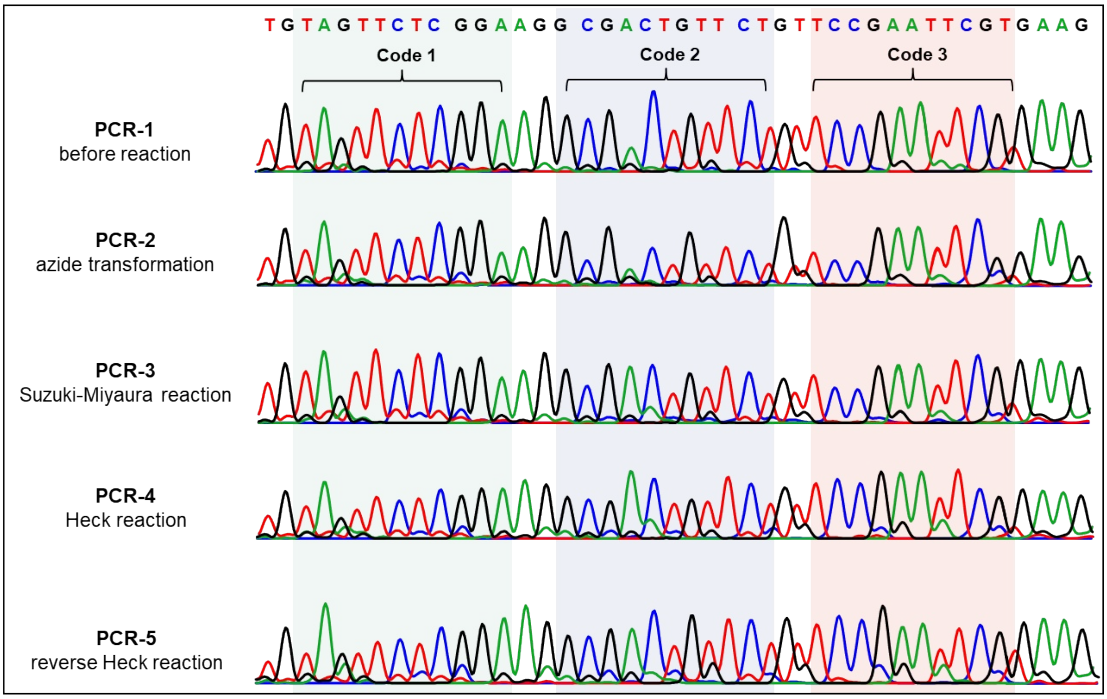

Supplementary Fig. 16 Sanger sequencing results of samples before and after chemical reactions

Seen from the sequencing results, the sequence of encoding regions in the four DNA sequences can be accurately decoded without mutation. In addition, the base peak noises were not significantly increased.

6.4 DNA Sequences

Supplementary Table 10. DNA Sequences of ligation, PCR and sequencing

Name	Code sequence
Code 1	ACGGACTAACAAG
	TGTTAGTCCGTCA
Code 123	TAGTTCTCGGAAGGCGACTGTTCTGTTCCGAATTCGTGAAGATCTGATGGCGCGAGGG
	CCCTCGCGCCATCAGATCTTCACGAATTCGGAACAGAACAGTCGCCTTCCGAGAACTACA
PCR product	AATGATACGGCGACCACCGAGATCTACACTCTTTCCCTACATGACTCCCGACCGAA GGTTGTAGTTCTCGGAAGGCGACTGTTCTGTTCCGAATTCGTGAAGATCTGATGGCG CGAGGGTGAGGTCAGTGCGGTTATAGAGCATA CGGCAGAAGACGAAC
amplification primers	Forward Primer: AATGATACGGCGACCACCGAGATCTACACTCTTTCCCTACATGACTCCCGACCGAAGGT
	Reverse Primer: CAAGCAGAAGACGGCATACGAGATATTGGCGTGACTGGAGTC CCTCGCGCCATCAGATC
sequencing primers	Forward Primer: CGGCGACCACCGAGATCTACACTC
	Reverse Primer: AAGACGGCATACGAGATATTGGCG

All the DNA sequences were written in 5’-to 3’-orientation unless otherwise noted.

7. Application of on-DNA carbon-carbon bond formation in DNA-encoded pharmaceutical compound and chemical probe synthesis

7.1 Comparative study of on-DNA functional group synthesis

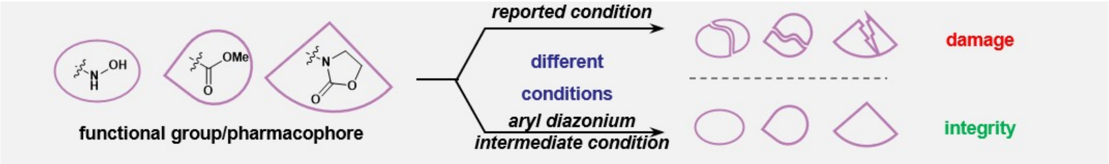

Supplementary Fig. 17 on-DNA bioactive compound synthesis

Reported condition: To the DNA conjugate aryl iodide (5 μL, 1 mM in H_2O, 5 nmol,) were added 5 μL DMA, 500 equiv of styrene (5 μL, 500 mM in DMA, 2500 nmol), 2 equiv of $PdCl_2$ (COD) (1 μL, 10 mM in DMA, 10 nmol) and 500 equiv of K_2CO_3 (5 μL, 500 mM in H_2O, 2500 nmol). The mixture was vortexed. The reaction was proceeded at 80 °C for 6 h. Next, 5 M NaCl solution (10 % by volume) and cold ethanol (2.5 times by volume, ethanol stored at -20 °C) was added. The mixture was stored at a -80 °C freezer for more than 30 minutes. Then the sample was centrifuged for 30 min at 4 °C in a microcentrifuge at 10000 rpm. The above supernatant was removed and the pellet (precipitate) was cooled in liquid nitrogen and then placed on a lyophilizer. After lyophilization, the dry pellet was recovered.[4]

Aryl diazonium intermediate condition: **On-DNA aryl diazonium salt formation:** To the DNA conjugate **1a** (8 μL, 25 μM in H_2O, 0.2 nmol) was added with *t*BuONO (2 μL, 50 mM in DMA, 100 nmol) and H_2O (10 μL). Then the resulting mixture was vortexed and stood at 25 °C for 1.5 h.

On-DNA Heck reaction: After the aryl diazonium intermediate was generated according to the above method, $Pd(OAc)_2$ (2 μL, 20 mM in DMA) was added. The mixture was vortexed and added a mixed solution of phosphate buffer (3 μL, 250 mM in H_2O, pH 5.5), H_2O (3 μL) and styrene (2 μL, 500 mM in DMA), then stood for reaction at 25 °C for 30 min. After reaction, 30 equiv. of sodium diethyldithiocarbamic acid (compared with $Pd(OAc)_2$) was added to the mixture, and the reaction mixture was stood at 25 °C for 30 minutes. The mixture was centrifuged at 25 °C for 10 min at 13,500 rpm, and the resultant supernatant was collected. The product was obtained by

ethanol precipitation as described above and characterized by UPLC-MS. All the DNA-aniline conjugates and olefins were performed using the method above. During the reaction, attention must be paid to: the sequence of adding reagents should not be reversed.

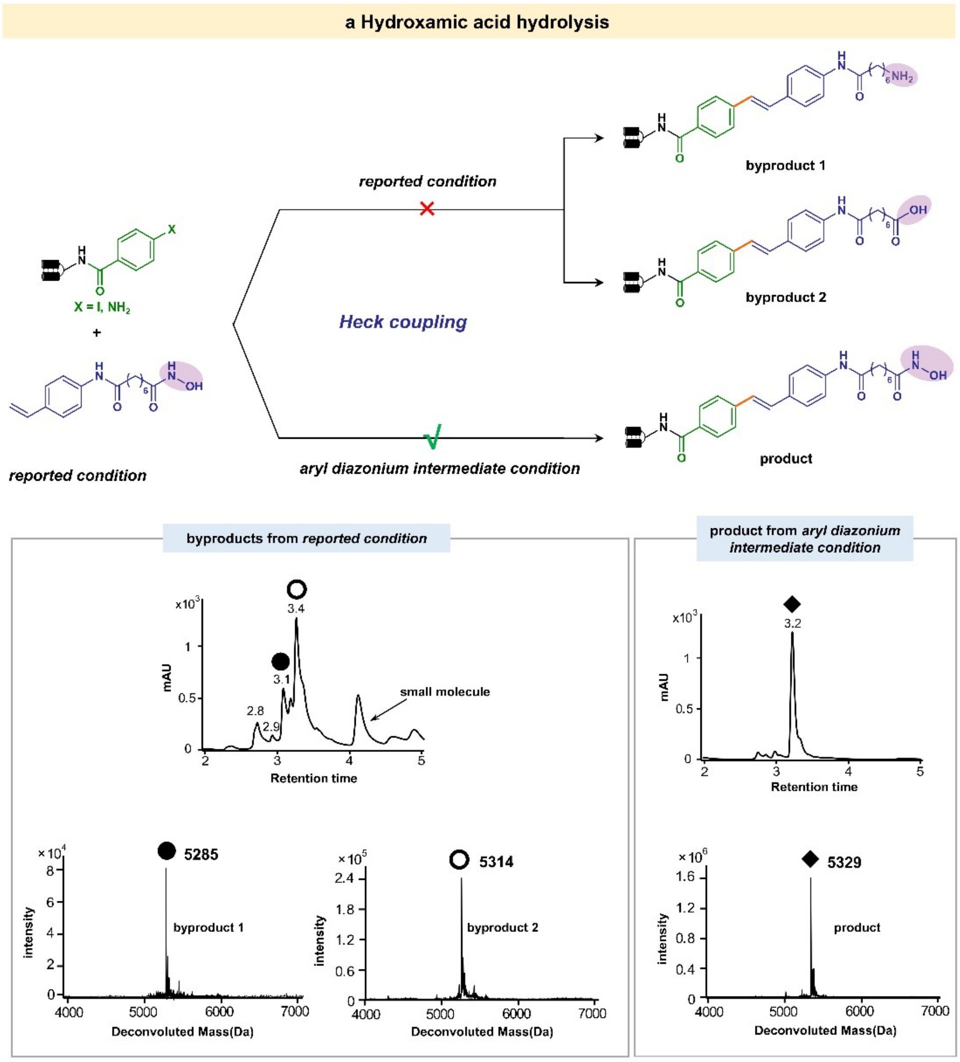

Supplementary Fig. 18 The effects of two different Heck reactions on the hydroxamic acid. Calculated Mass: 5329 Da; **Found Mass**: 5285 Da (byproducts), 5314 Da (byproducts); 5329 Da (product).

According to the mass data, the previously reported condition failed to generate the target product containing the hydroxamic acid pharmacophore. On the contrary, two by-products were obtained. By speculating from the mass spectrometry data and literatures, we inferred that byproducts containing an amine group and an carboxylic acid group were generated under basic and heating conditions, respectively.[5]

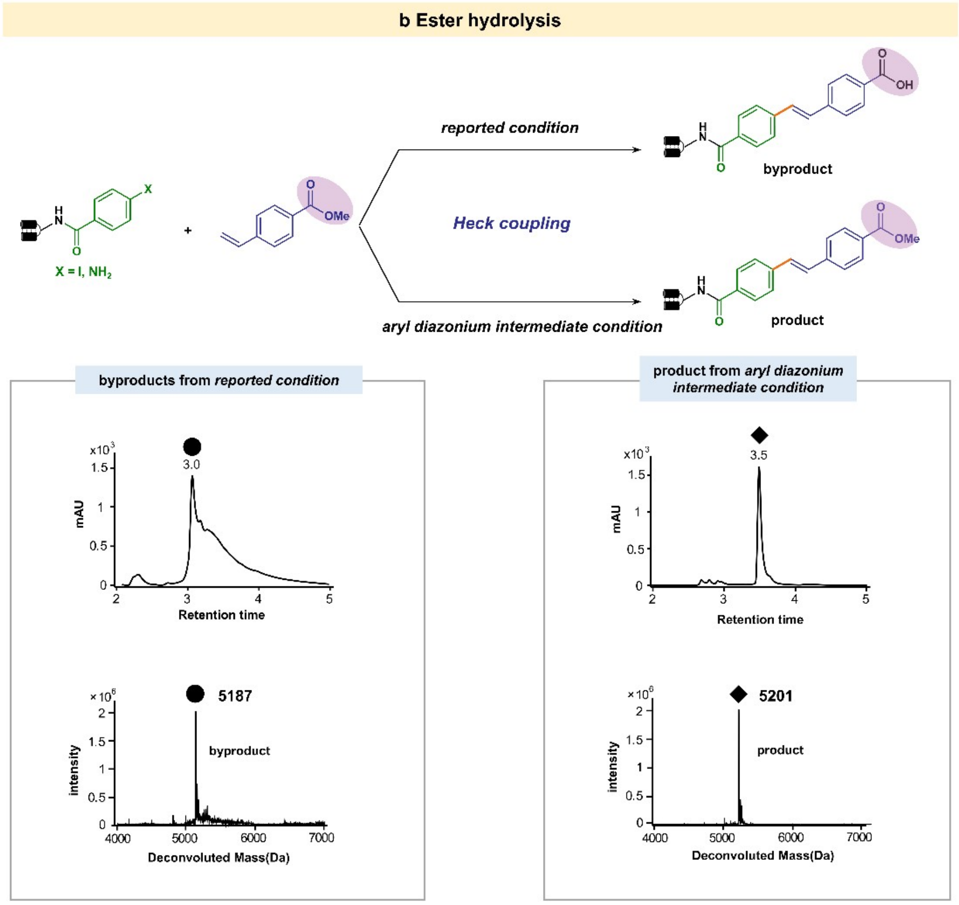

Supplementary Fig. 19 The effects of two different Heck reactions on the ester group. Calculated Mass: 5201 Da; **Found Mass**: 5187 Da (byproduct), 5201 Da (product).

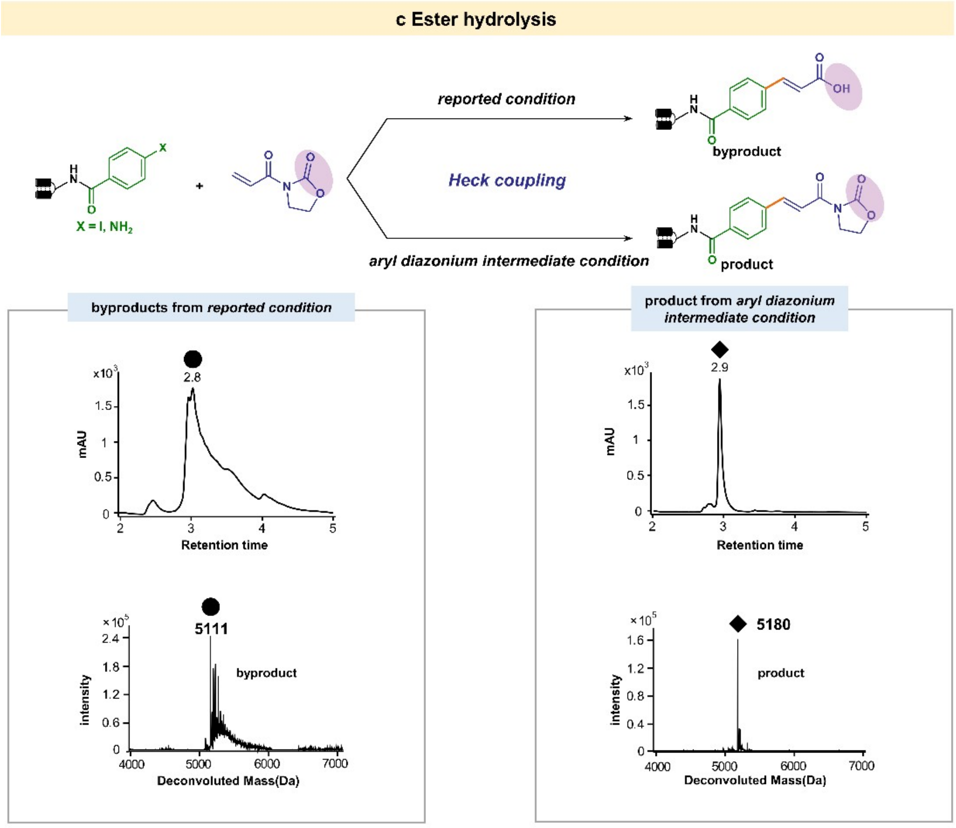

Supplementary Fig. 20 The effects of two different Heck reactions on the oxazolidone. Calculated Mass: 5180 Da; **Found Mass**:5111 Da (byproducts); 5180 Da (product)

Similarly, on-DNA library synthesis utilizing the previously reported method will cause the hydrolysis of the ester or oxazolidone[6] functional group into the carboxyl group, thus failing to obtain the target product. These data together demonstrated the mild transformation/coupling condition of the aryl diazonium-based approach was superior in aspects of substrate compatibility and functional group tolerance.

7.2 Comparative study of on-DNA pharmacophore (HDAC inhibitor) synthesis

Synthesis of the SAHA derivative.

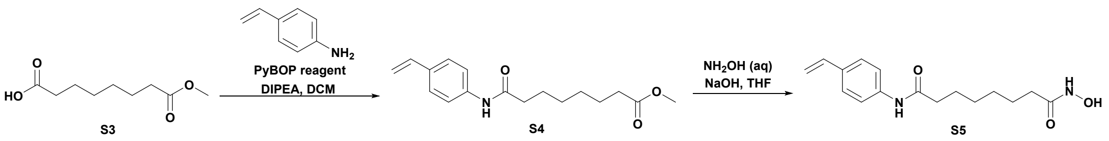

To a stirred solution of 8-methoxy-8-oxooctanoic acid (S3, 188.1 mg, 1.0 mmol) in anhydrous DCM (10 mL) were sequentially added 4-vinylaniline (119.1 mg, 1.0 mmol), PyBOP hexafluorophosphate (624.5 mg, 1.2 mmol), and DIPEA (258.6 mg, 2.0 mmol) at 0 ºC. The reaction mixture was warmed to room temperature gradually, and was stirred for 4 h, the resulting solution was extracted three times with dichloromethane (3×10 mL) and the combined organic layers were dried over sodium sulfate. The dried solution was filtered and concentrated in vacuo. The residue was purified by flash column chromatography to afford the product methyl 8-oxo-8-((4-vinylphenyl)amino)-octanoate as a white solid (S4, 202.4 mg, 70%), which was used in the next step of synthesis without further characterization.

To a stirred solution of S4 (144.6 mg, 0.5 mmol) in THF (10 mL) were added freshly prepared aqueous hydroxylamine (330.0 mg, 10.0 mmol) and sodium hydroxide (40.0 mg, 1.0 mmol) in methanol (1.0 mL) at 0 ºC. After stirring for 3 h at the same temperature, white precipitate was formed. The solvent was removed and the solid was added with water (3 mL) and hydrochloric acid was added drop-wisely until the solution was neutralized. The resulting solution was extracted three times with EA (3×10 mL) and the combined organic layers were washed with saturated sodium chloride solution (3×10 mL). The water layer was removed and the organic layer was then dried over sodium sulfate. The dried solution was concentrated in vacuo to afford the product as a white solid (S5, 108.8 mg, 75%).[7] HRMS (*m/z*, $C_{16}H_{23}N_2O_3$, ESI): calculated $[M+H]^+$: 291.1703; found: 291.1700. ^1H NMR (400 MHz, DMSO-d_6) δ 10.33 (s, 1H), 9.91 (s, 1H), 8.66 (s, 1H), 7.57 (d, J = 8.4 Hz, 2H), 7.39 (d, J = 8.4 Hz, 2H), 6.65 (dd, J = 17.6, 10.9 Hz, 1H), 5.71 (d, J = 17.7 Hz, 1H), 5.15 (d, J = 11.0 Hz, 1H), 2.28 (t, J = 7.3 Hz, 2H), 1.93 (t, J = 7.3 Hz, 2H), 1.56 (d, J = 6.8 Hz, 2H), 1.51 – 1.44 (m, 2H), 1.27 (d, J

= 2.4 Hz, 4H). ^{13}C NMR (101 MHz, DMSO-d_6) δ 171.70 (s), 169.59 (s), 139.16 (s), 136.38 (s), 132.23 (s), 126.77 (s), 119.31 (s), 112.95 (s), 36.60 (s), 32.47 (s), 28.59 (s), 25.26 (s).

^{1}H-NMR of **S5**

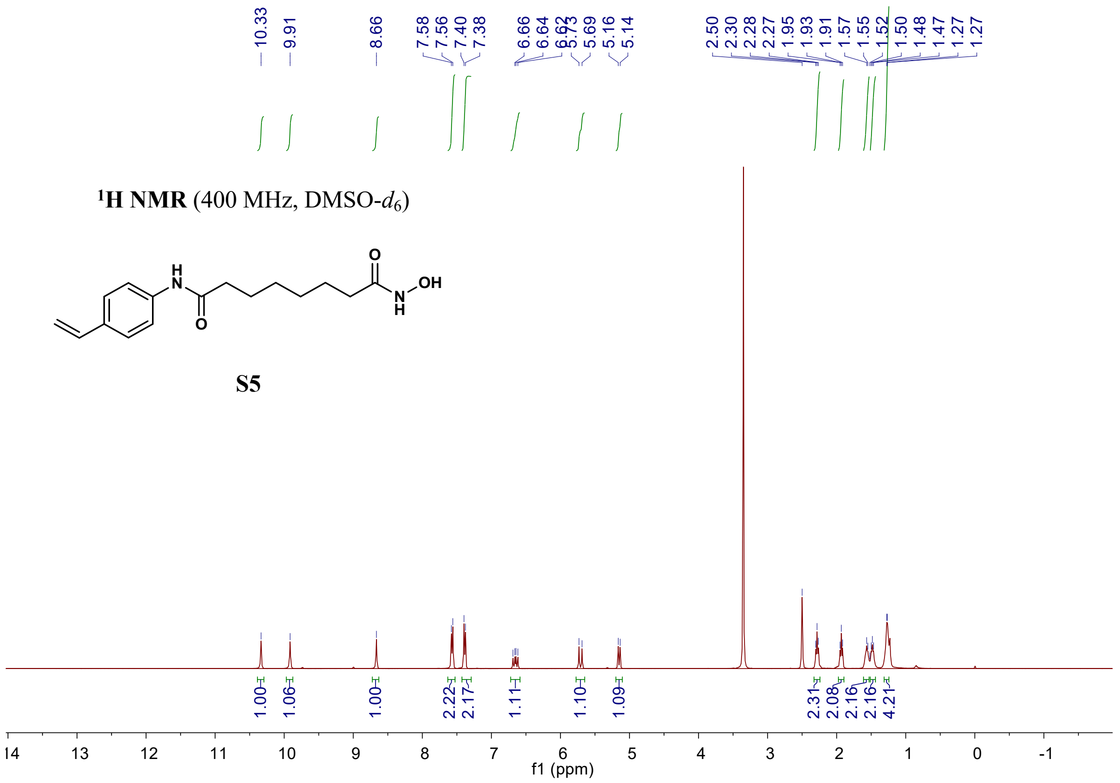

^{13}C-NMR of **S5**

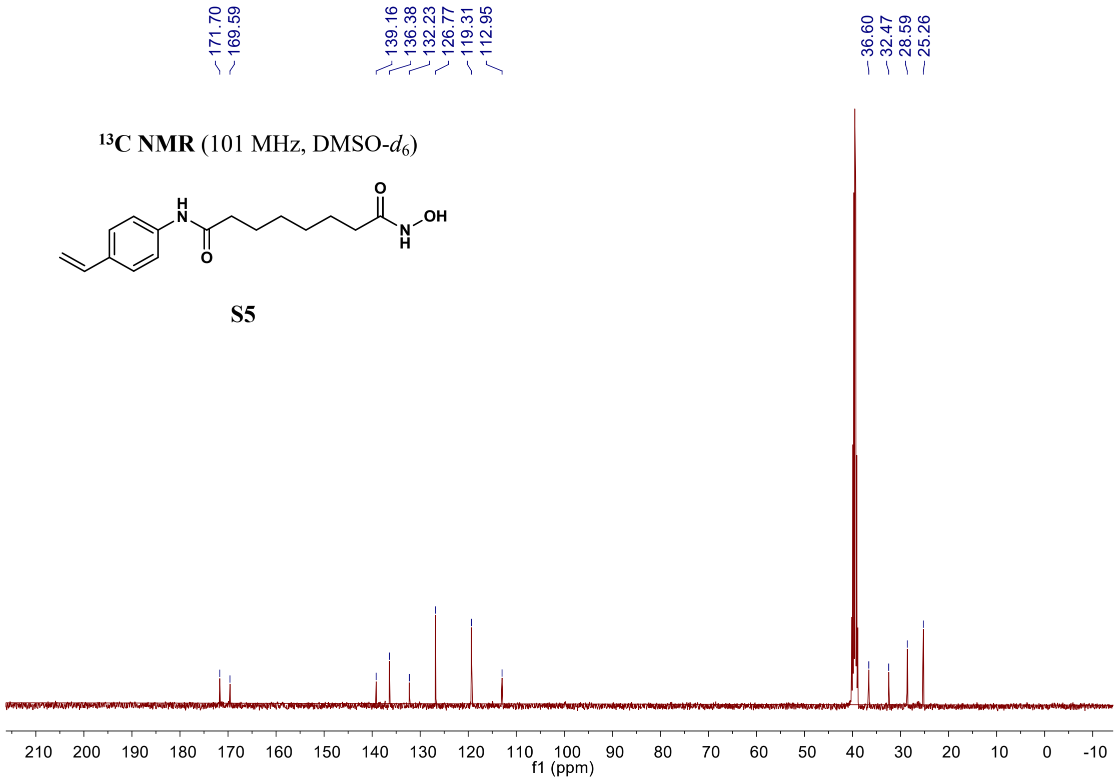
171.70
169.59
139.16
136.38
132.23
126.77
119.31
112.95
36.60
32.47
28.59
25.26
13C NMR (101 MHz, DMSO-d_6)
H
N
O
O
N
OH
H
S5
210 200 190 180 170 160 150 140 130 120 110 100 90 80 70 60 50 40 30 20 10 0 -10
f1 (ppm)

7.3 General method for DNA Programmed Affinity Labeling (DPAL)

A mixture containing the corresponding BP (1 μM), CP (1 μM), 1x PBS buffer (pH 7.4), 0.1 M NaCl, and CA II protein (1 μM) was incubated at 4 ºC for 2 h. For the competition experiments, acetazolamide was also added to the mixture at a concentration of 20 μM and incubated at 4 ºC for 30 min prior to BP/CP addition. The total sample volume was typically 50 μL. The mixture was irradiated under UV at 365 nm for 10 minutes over ice. The sample was then added with 5x Loading buffer, heated to 95 ºC for 10 min, and then analyzed with SDS-PAGE.[8] Gel images were captured and analyzed by Bio-Rad Chemidoc™ imaging system.

8. UPLC chromatograph and deconvoluted MS

8.1 Substrate scope of DNA-conjugated arylamines for the On-DNA azide transformation

UPLC chromatograph and deconvoluted MS of **1a'.**

Conversion: >95%

Calculated Mass: 5082 Da; Found Mass: 5082 Da

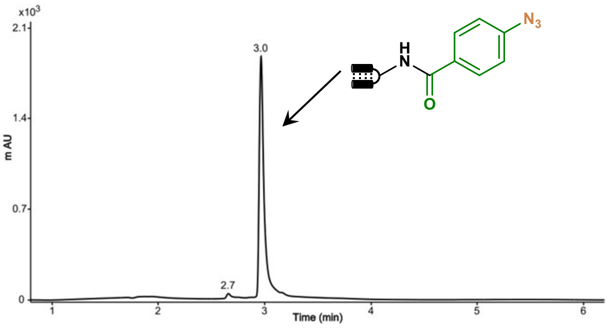

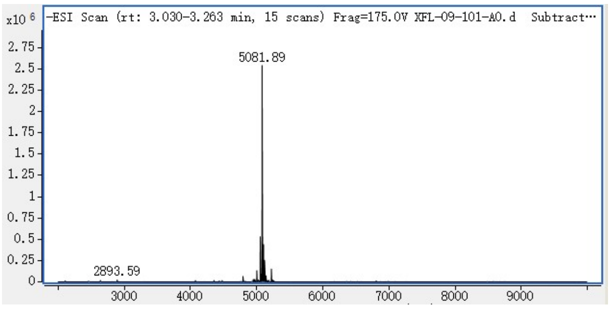

UPLC chromatograph and deconvoluted MS of **1c’.**

Conversion: >95%

Calculated Mass: 5150 Da; Found Mass: 5150 Da

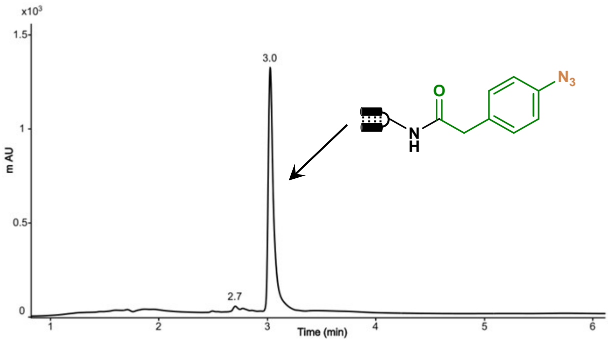

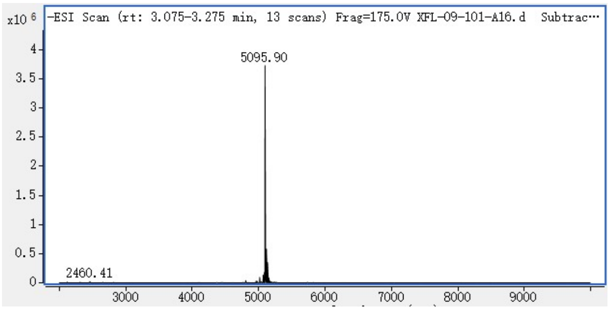

UPLC chromatograph and deconvoluted MS of **1i’.**

Conversion: 83%

Calculated Mass: 5132 Da; Found Mass: 5132 Da

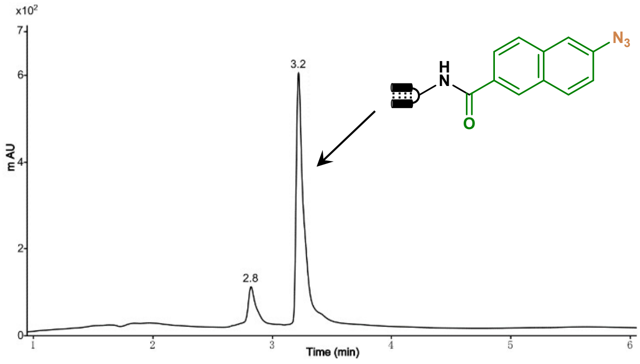

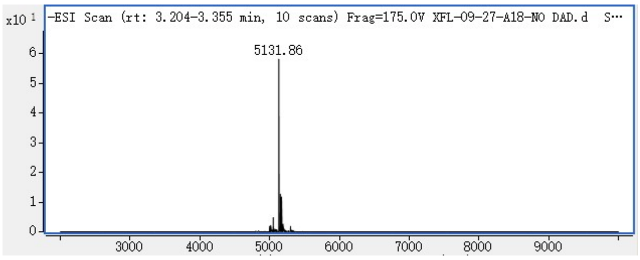

UPLC chromatograph and deconvoluted MS of **1j'.**

Conversion: >95%

Calculated Mass: 5118 Da; Found Mass: 5118 Da

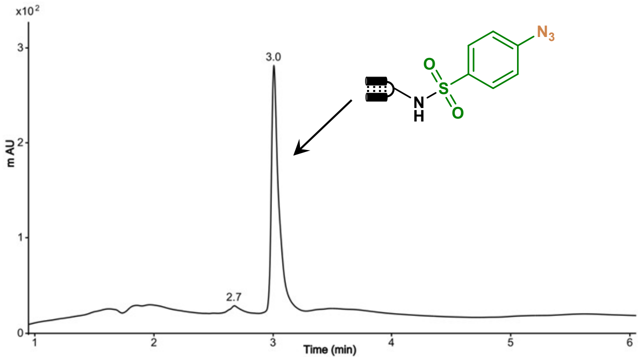

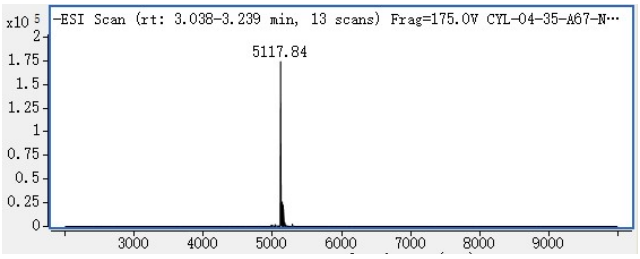

UPLC chromatograph and deconvoluted MS of **1v'.**

Conversion: >95%

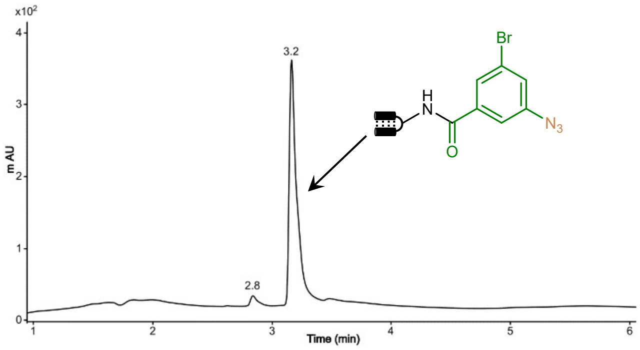

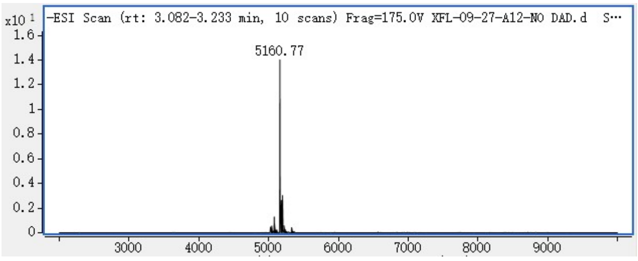

Calculated Mass: 5160 Da; Found Mass: 5161 Da

UPLC chromatograph and deconvoluted MS of **1w’.**

Conversion: 75%

Calculated Mass: 5121 Da; Found Mass: 5121 Da

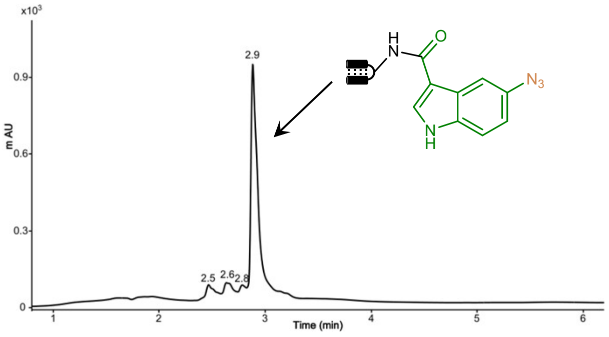

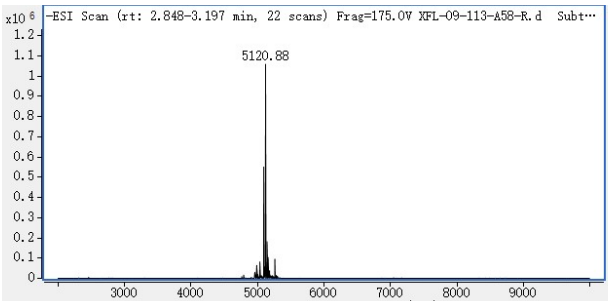

8.2 Substrate scope of DNA-conjugated azide for the On-DNA CuAAC reaction

UPLC chromatograph and deconvoluted MS of **9a.**

Conversion: >90%

Calculated Mass: 5184 Da; Found Mass: 5184 Da

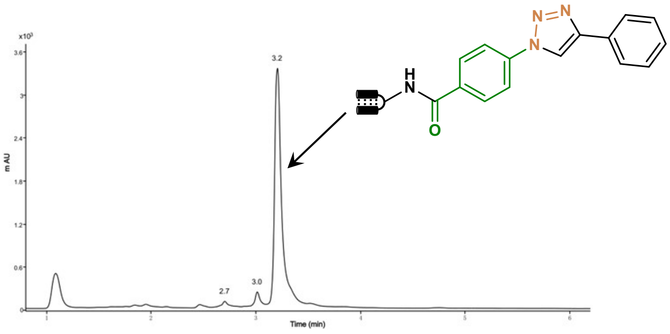

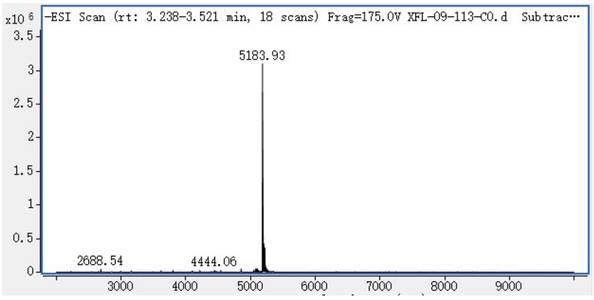

UPLC chromatograph and deconvoluted MS of **9c.**

Conversion: >90%

Calculated Mass: 5234 Da; Found Mass: 5234 Da

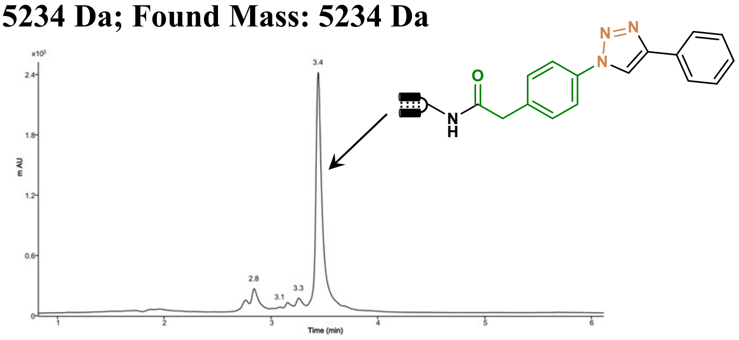

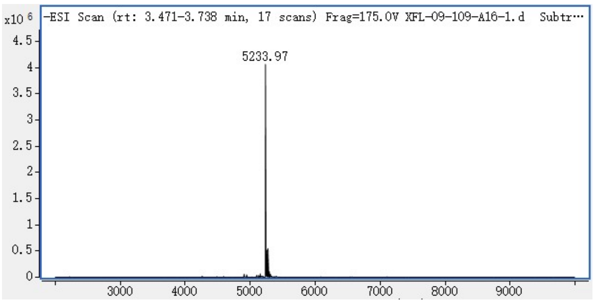

UPLC chromatograph and deconvoluted MS of **9i.**

Conversion: >90%

Calculated Mass: 5220 Da; Found Mass: 5220 Da

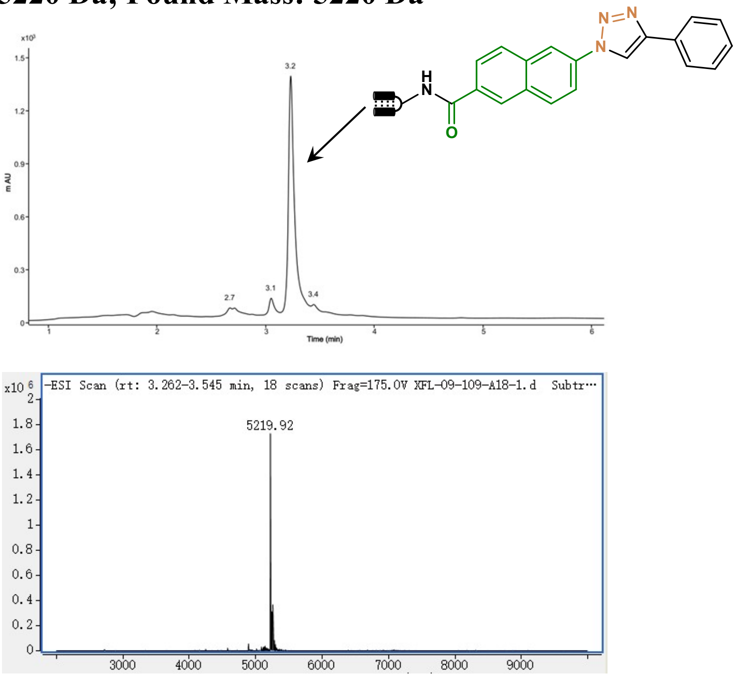

UPLC chromatograph and deconvoluted MS of **9j.**

Conversion: >90%

Calculated Mass: 5198 Da; Found Mass: 5198 Da

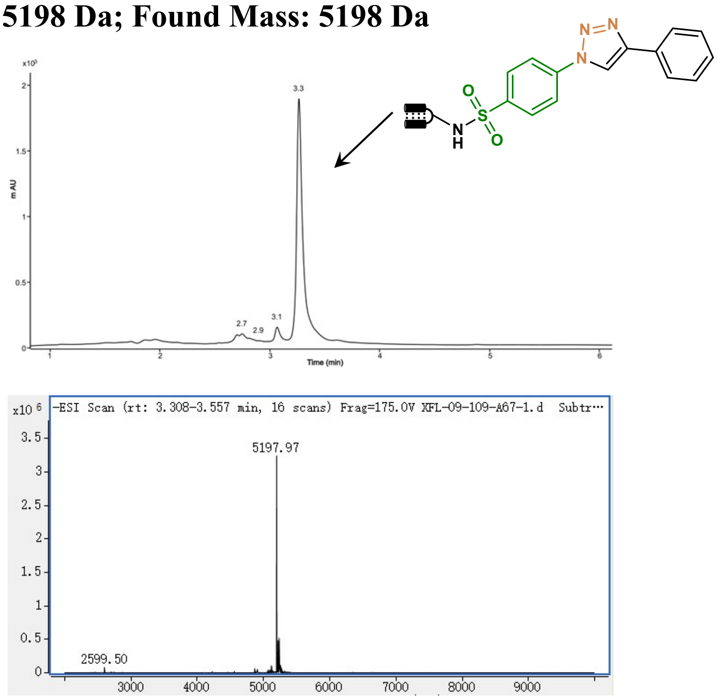

UPLC chromatograph and deconvoluted MS of **9v.**

Conversion: >90%

Calculated Mass: 5263 Da; Found Mass: 5263 Da

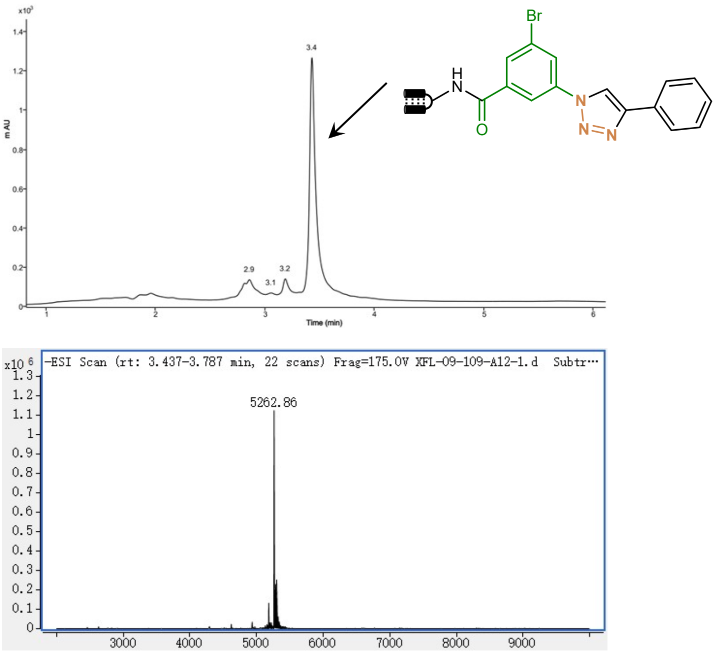

UPLC chromatograph and deconvoluted MS of **9w.**

Conversion: >90%

Calculated Mass: 5223 Da; Found Mass: 5223 Da

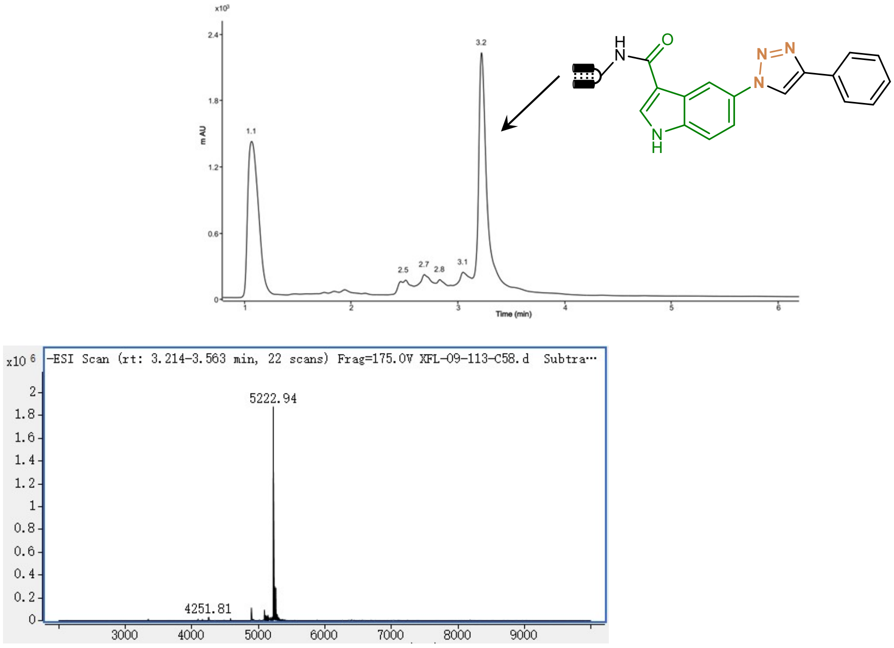

8.3 Substrate scope of boronic acids for the on-DNA Suzuki-Miyaura coupling with 1a

UPLC chromatograph and deconvoluted MS of **3a.**

Conversion: 73%

Calculated Mass: 5117 Da; Found Mass: 5117 Da

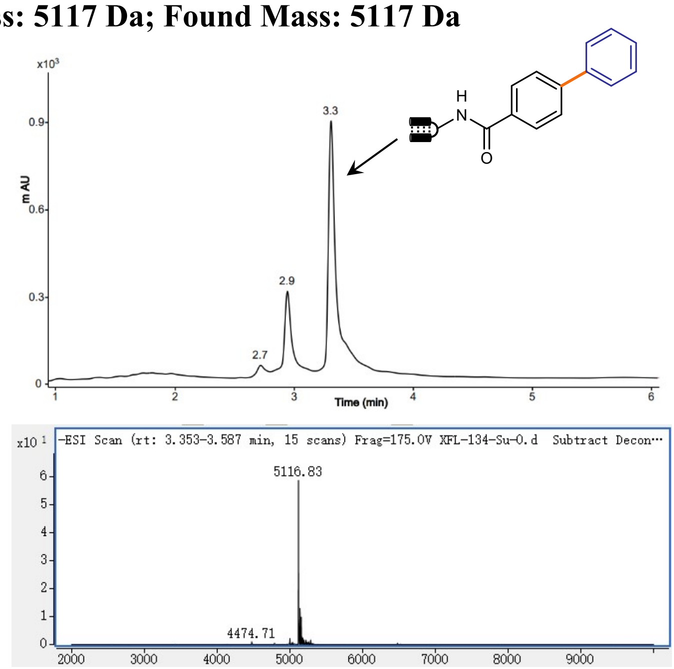

UPLC chromatograph and deconvoluted MS of **3b.**

Conversion: 62%

Calculated Mass: 5131 Da; Found Mass: 5131 Da

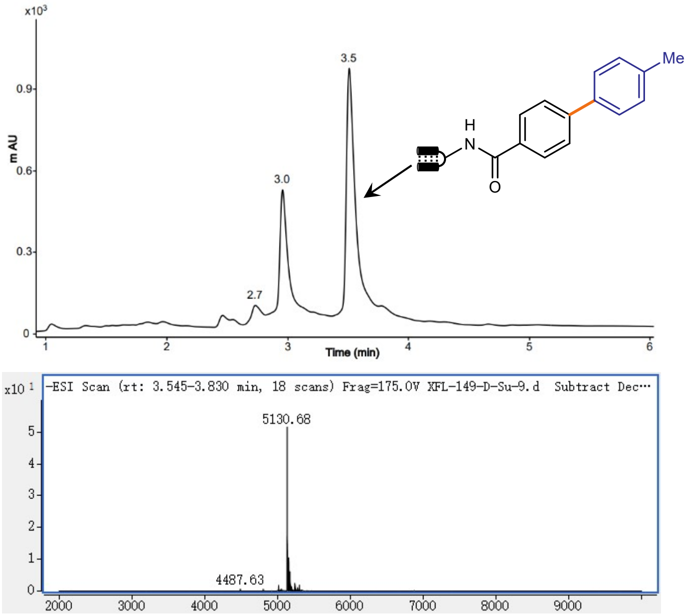

UPLC chromatograph and deconvoluted MS of **3c.**

Conversion: 77%

Calculated Mass: 5147 Da; Found Mass: 5147 Da

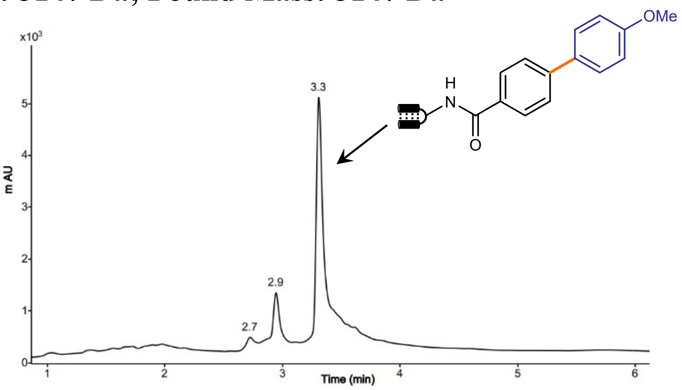

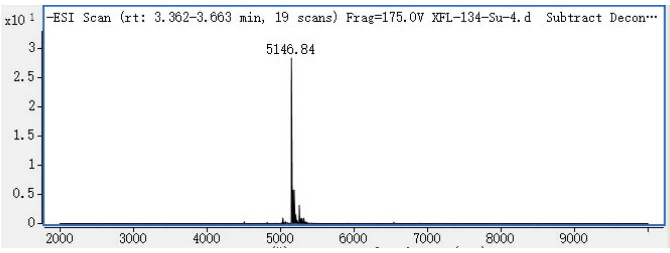

UPLC chromatograph and deconvoluted MS of **3d.**

Conversion: 63%

Calculated Mass: 5173 Da; Found Mass: 5173 Da

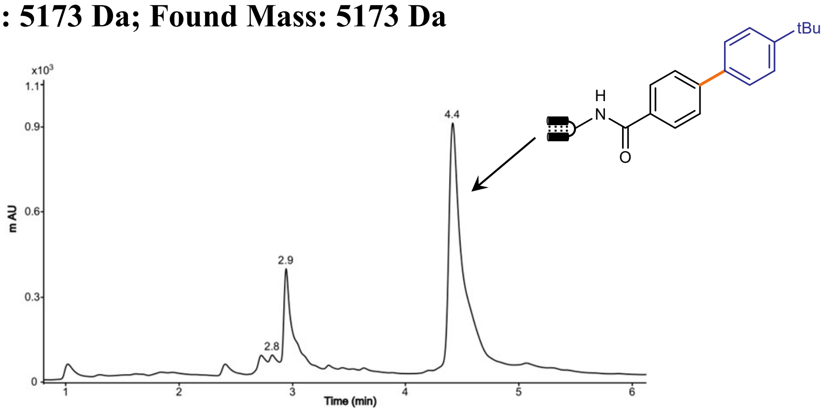

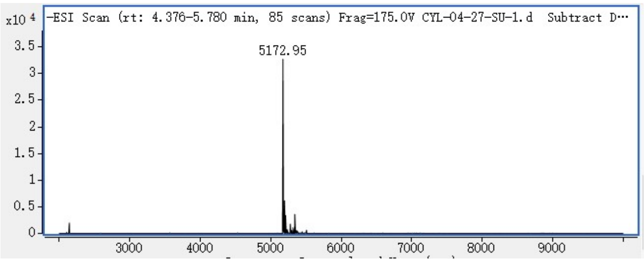

UPLC chromatograph and deconvoluted MS of **3e.**

Conversion: 91%

Calculated Mass: 5135 Da; Found Mass: 5135 Da

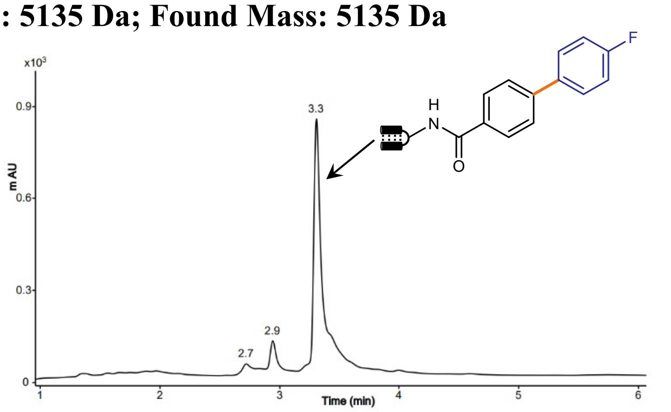

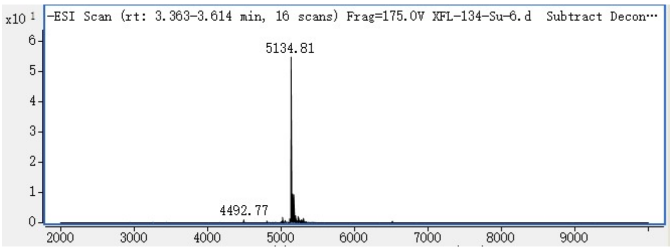

UPLC chromatograph and deconvoluted MS of **3f.**

Conversion: 65%

Calculated Mass: 5161 Da; Found Mass: 5161 Da

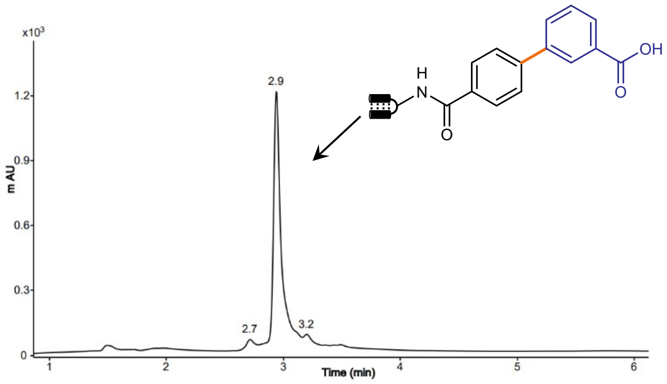

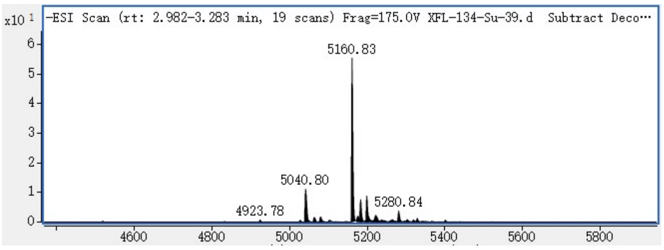

UPLC chromatograph and deconvoluted MS of **3g.**

Conversion: 75%

Calculated Mass: 5195 Da; Found Mass: 5195 Da

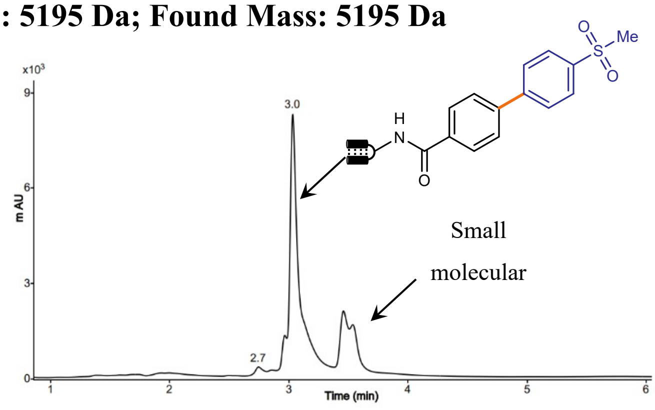

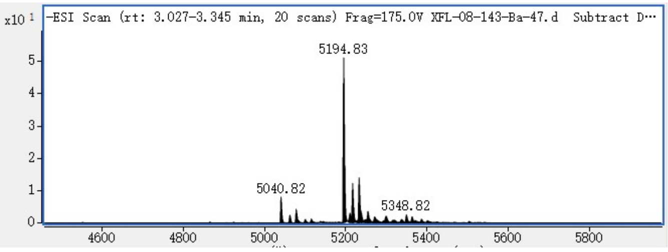

UPLC chromatograph and deconvoluted MS of **3h.**

Conversion: 70%

Calculated Mass: 5131 Da; Found Mass: 5131 Da

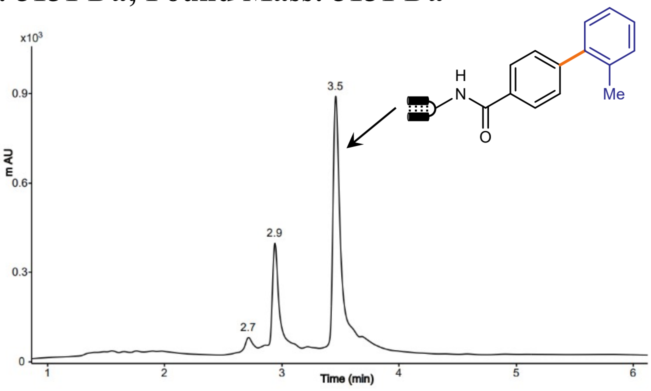

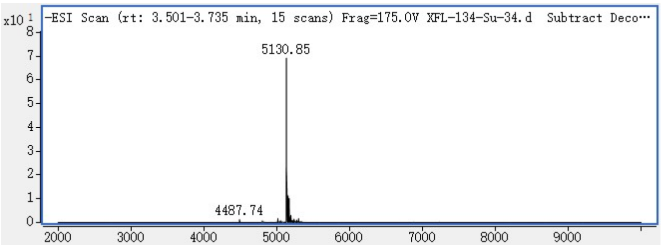

UPLC chromatograph and deconvoluted MS of **3i.**

Conversion: 68%

Calculated Mass: 5131 Da; Found Mass: 5131 Da

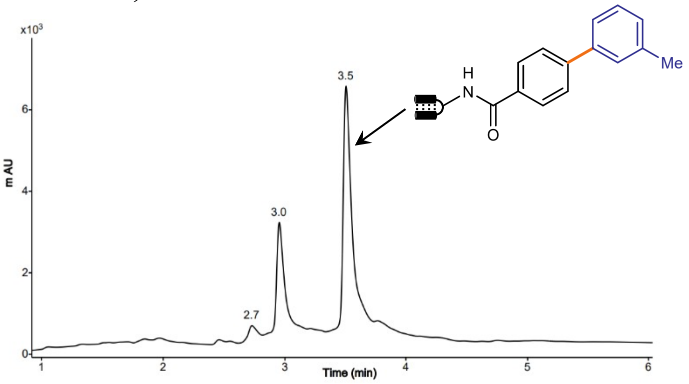

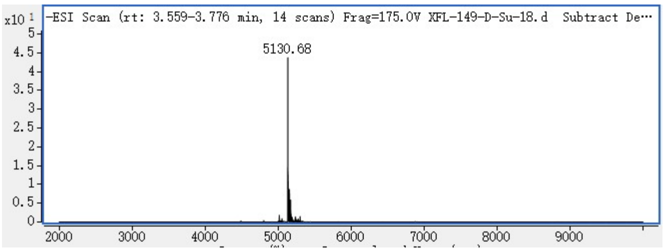

UPLC chromatograph and deconvoluted MS of **3j.**

Conversion: 82%

Calculated Mass: 5188 Da; Found Mass: 5188 Da

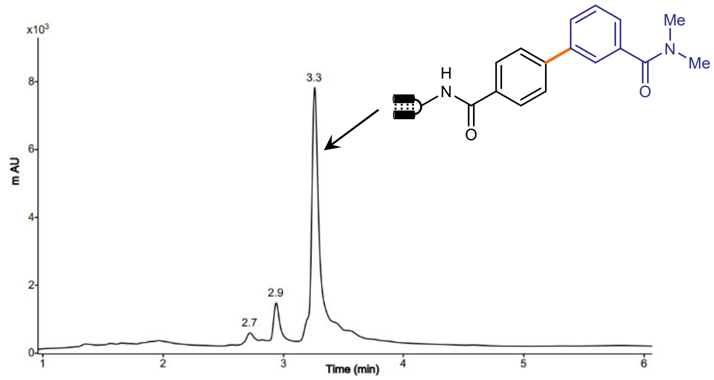

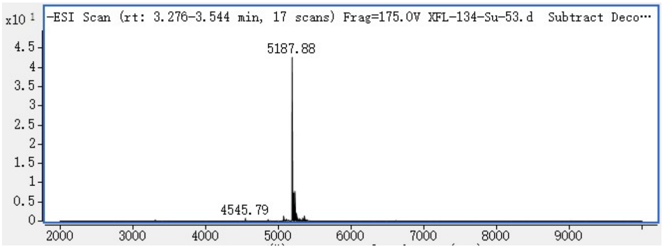

UPLC chromatograph and deconvoluted MS of **3k.**

Conversion: 78%

Calculated Mass: 5175 Da; Found Mass: 5175 Da

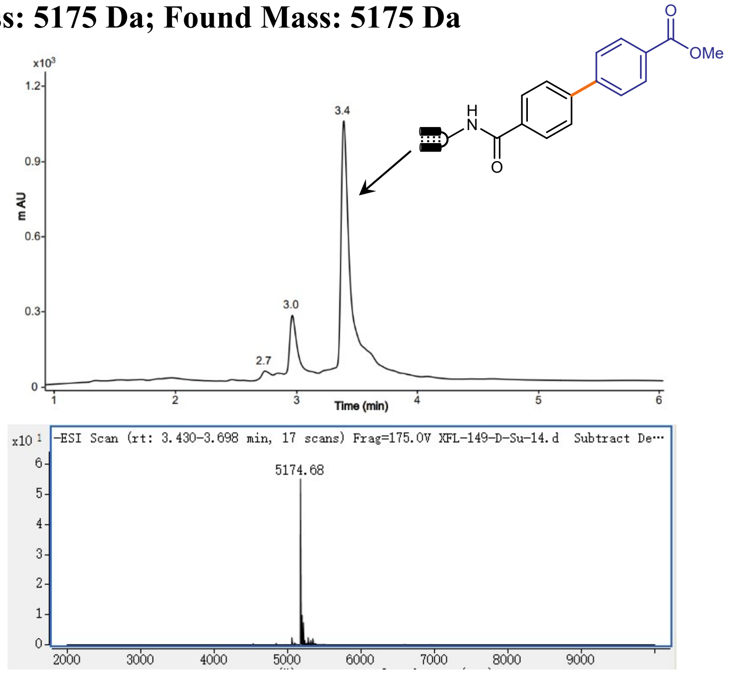

UPLC chromatograph and deconvoluted MS of **3l.**

Conversion: 63%

Calculated Mass: 5169 Da; Found Mass: 5169 Da

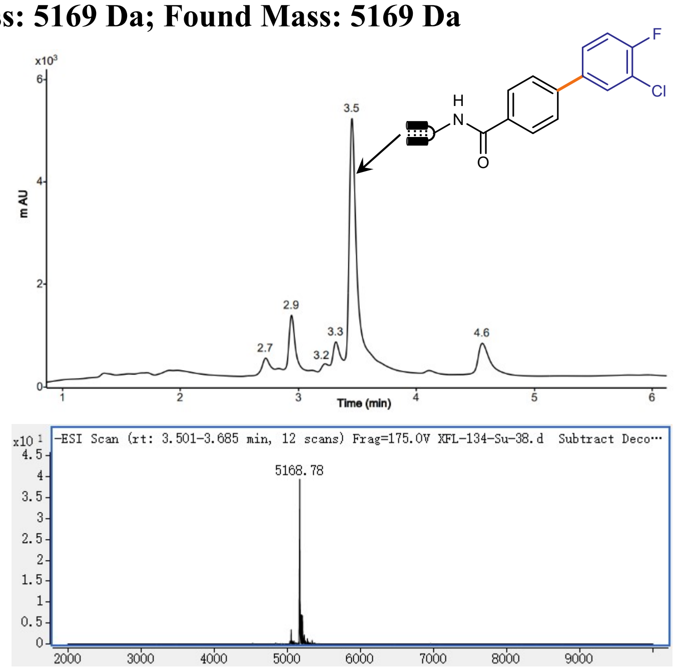

UPLC chromatograph and deconvoluted MS of **3m.**

Conversion: 85%

Calculated Mass: 5165 Da; Found Mass: 5165 Da

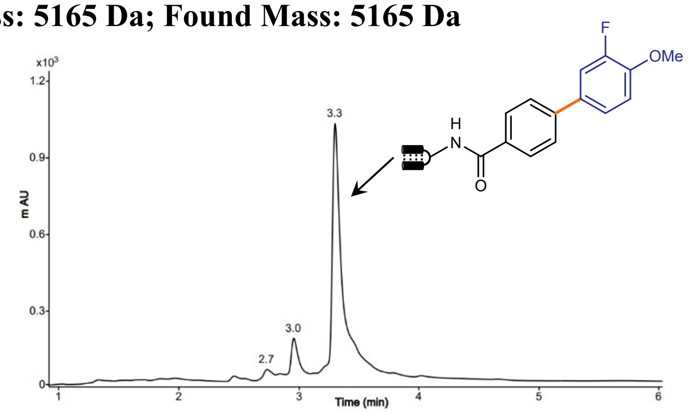

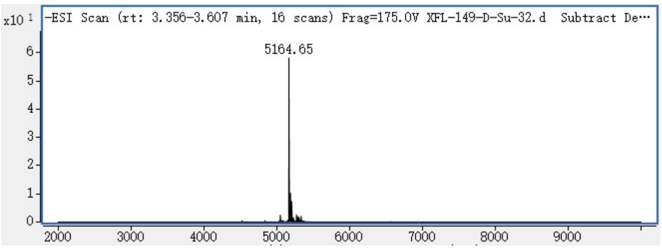

UPLC chromatograph and deconvoluted MS of **3n.**

Conversion: 83%

Calculated Mass: 5167 Da; Found Mass: 5167 Da

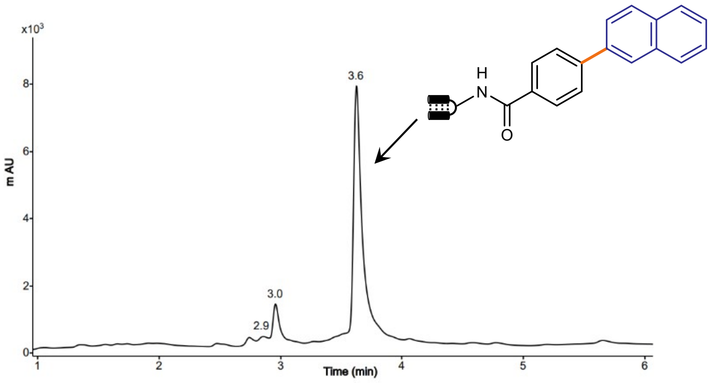

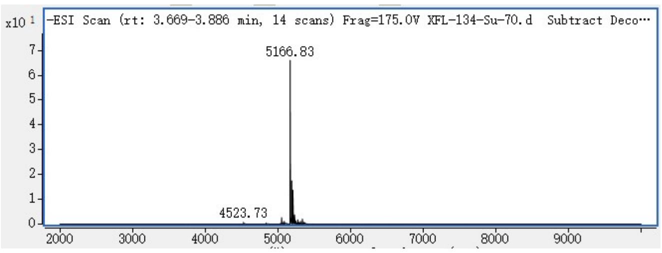

UPLC chromatograph and deconvoluted MS of **3o.**

Conversion: 85%

Calculated Mass: 5161 Da; Found Mass: 5161 Da

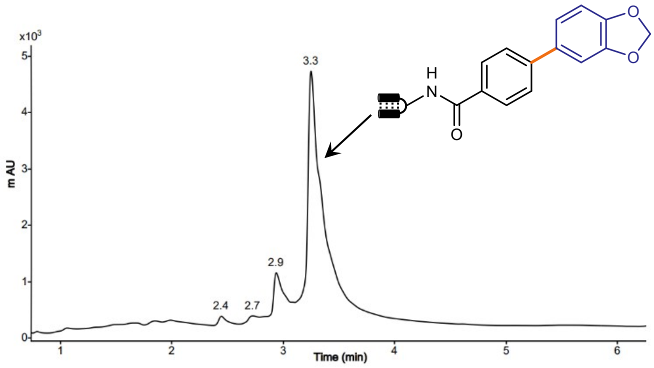

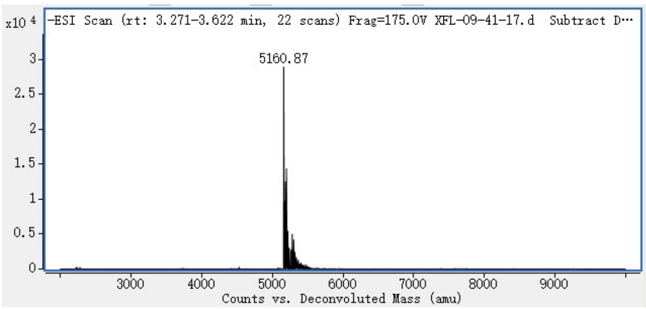

UPLC chromatograph and deconvoluted MS of **3p.**

Conversion: 80%

Calculated Mass: 5207 Da; Found Mass: 5207 Da

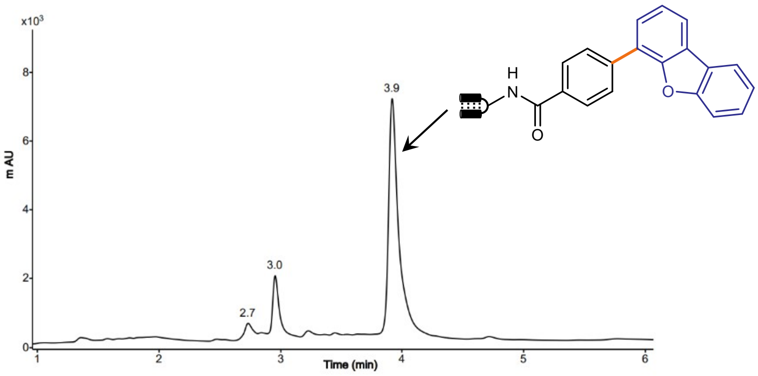

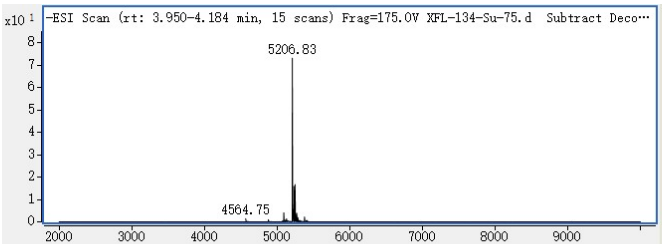

UPLC chromatograph and deconvoluted MS of **3q.**

Conversion: 82%

Calculated Mass: 5157 Da; Found Mass: 5157 Da

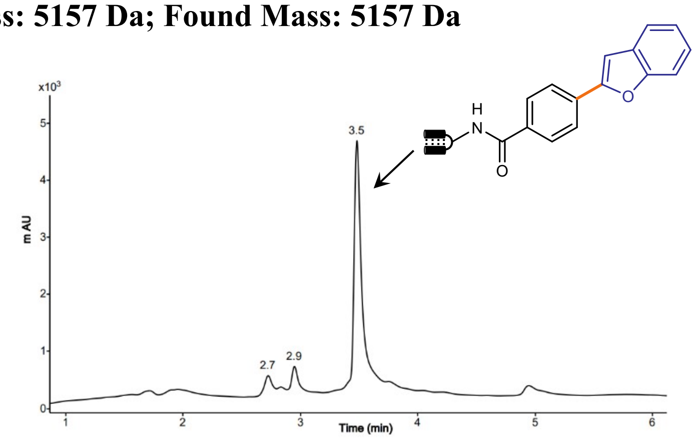

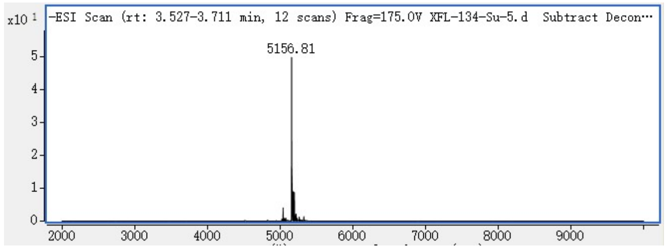

UPLC chromatograph and deconvoluted MS of **3r.**

Conversion: 77%

Calculated Mass: 5123 Da; Found Mass: 5123 Da

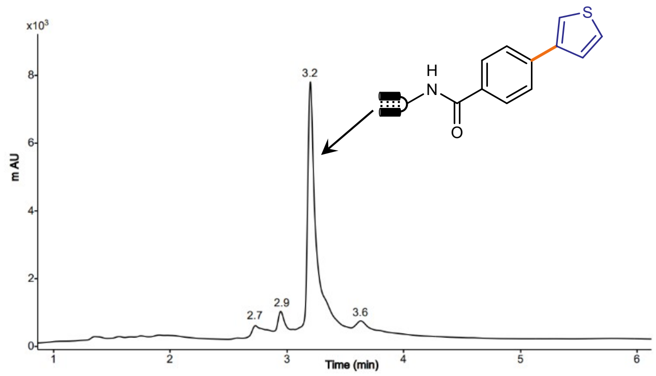

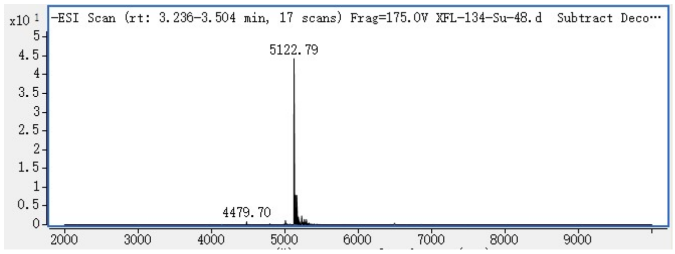

UPLC chromatograph and deconvoluted MS of **3s.**

Conversion: 60%

Calculated Mass: 5173 Da; Found Mass: 5173 Da

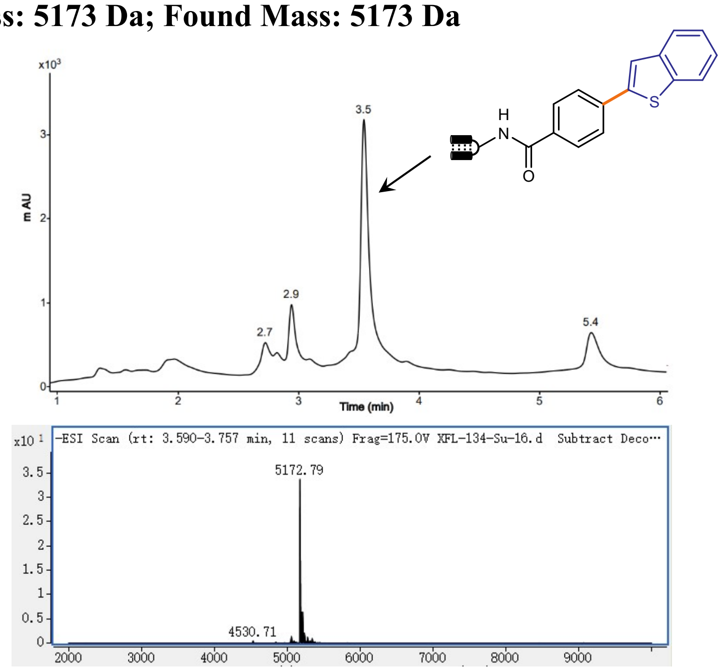

UPLC chromatograph and deconvoluted MS of **3t.**

Conversion: 78%

Calculated Mass: 5223 Da; Found Mass: 5223 Da

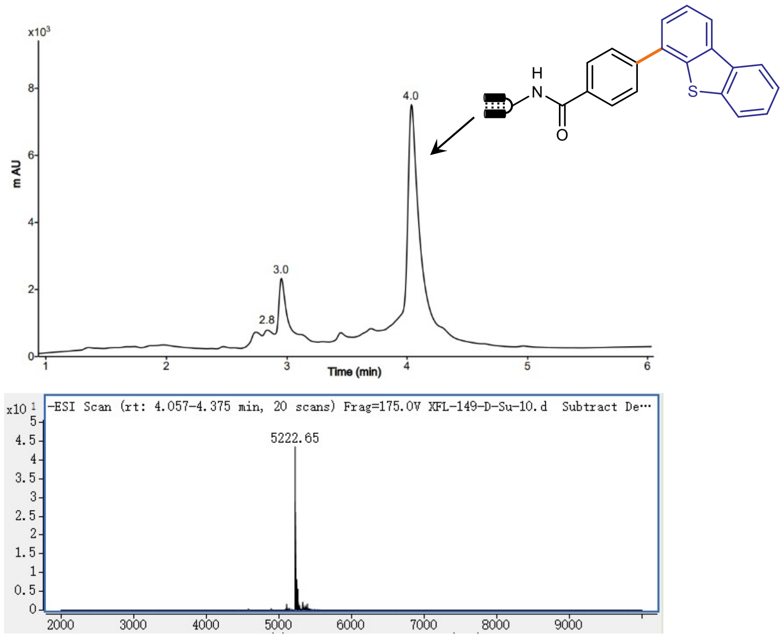

UPLC chromatograph and deconvoluted MS of **3u.**

Conversion: 83%

Calculated Mass: 5117 Da; Found Mass: 5117 Da

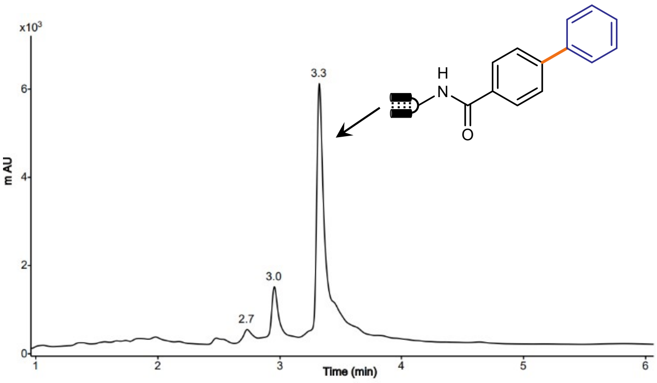

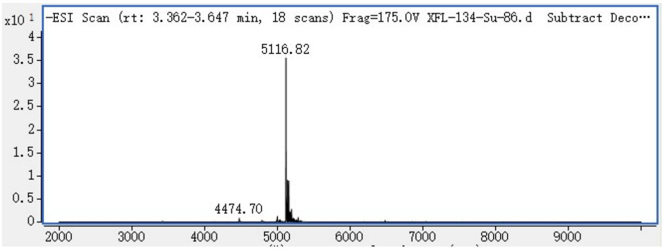

UPLC chromatograph and deconvoluted MS of **3v.**

Conversion: 70%

Calculated Mass: 5117 Da; Found Mass: 5117 Da

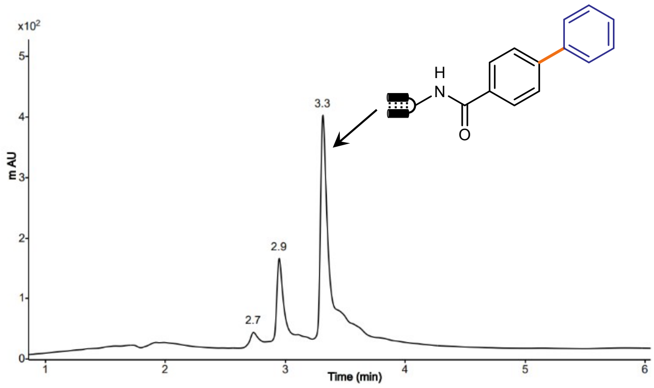

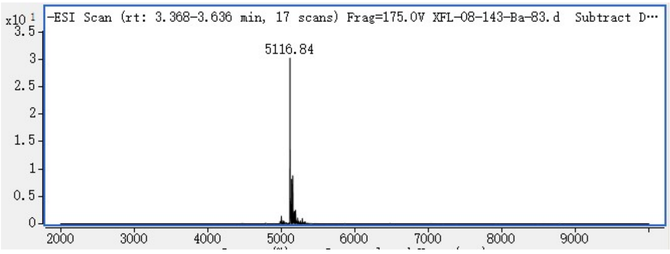

UPLC chromatograph and deconvoluted MS of **3w.**

Conversion: 83%

Calculated Mass: 5167 Da; Found Mass: 5167 Da

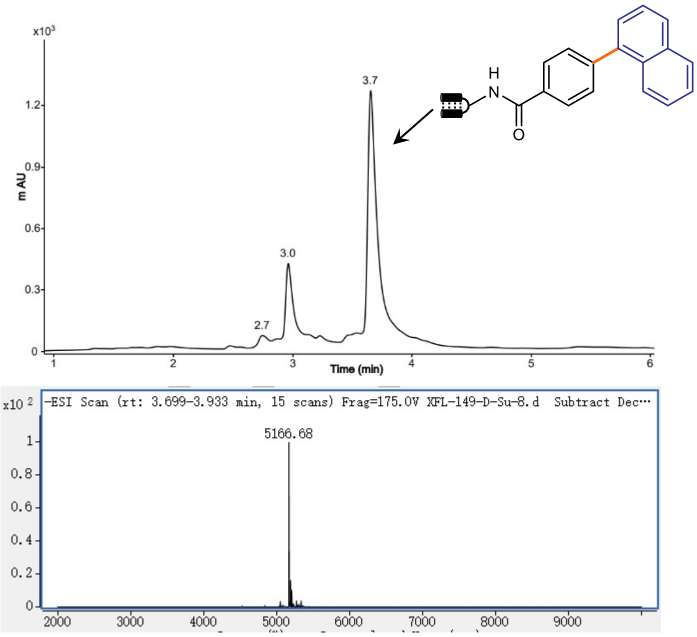

UPLC chromatograph and deconvoluted MS of **3x.**

Conversion: 50%

Calculated Mass: 5163 Da; Found Mass: 5163 Da

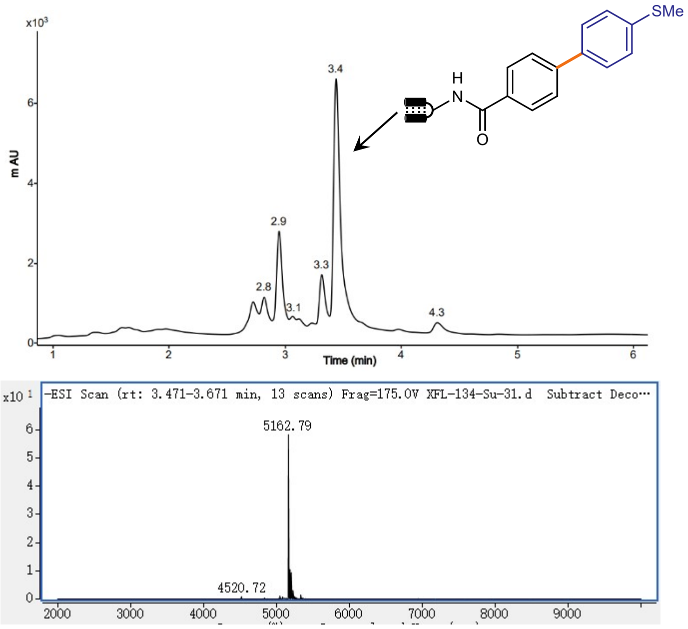

UPLC chromatograph and deconvoluted MS of **3y.**

Conversion: 79%

Calculated Mass: 5135 Da; Found Mass: 5135 Da

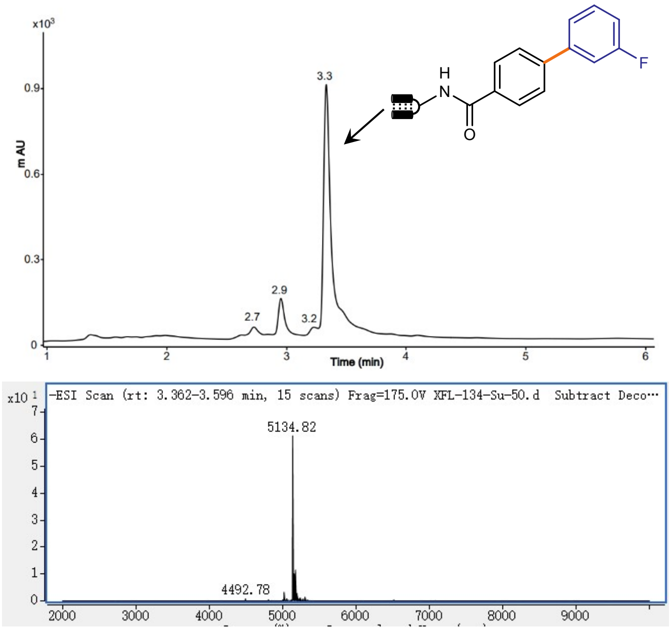

UPLC chromatograph and deconvoluted MS of **3z.**

Conversion: 66%

Calculated Mass: 5135 Da; Found Mass: 5135 Da

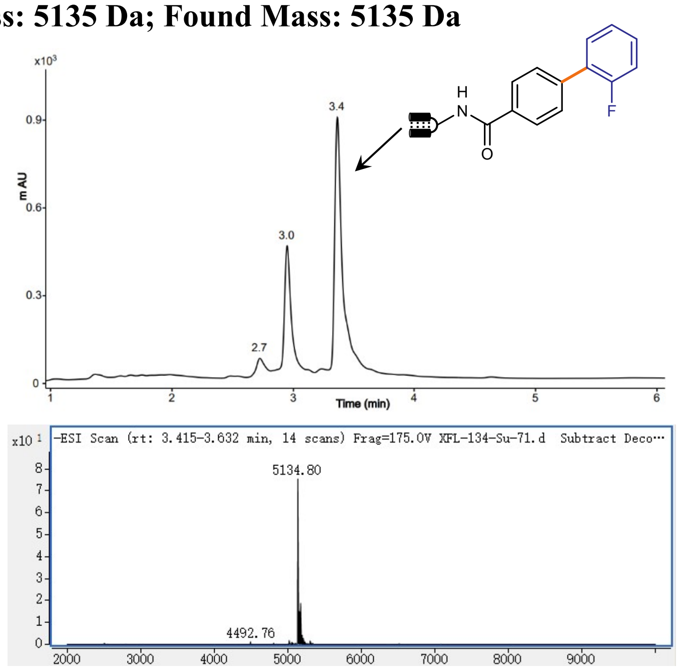

UPLC chromatograph and deconvoluted MS of **3aa.**

Conversion: 86%

Calculated Mass: 5153 Da; Found Mass: 5153 Da

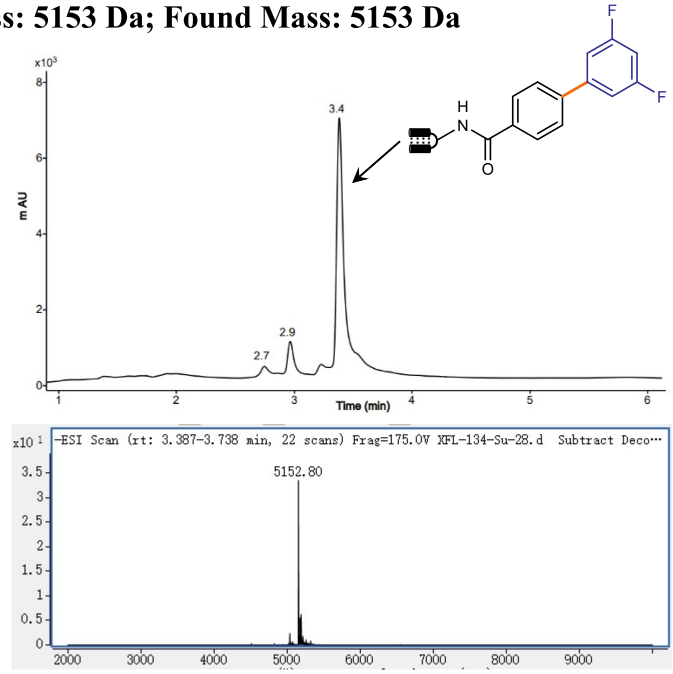

UPLC chromatograph and deconvoluted MS of **3ab.**

Conversion: 80%

Calculated Mass: 5185 Da; Found Mass: 5185 Da

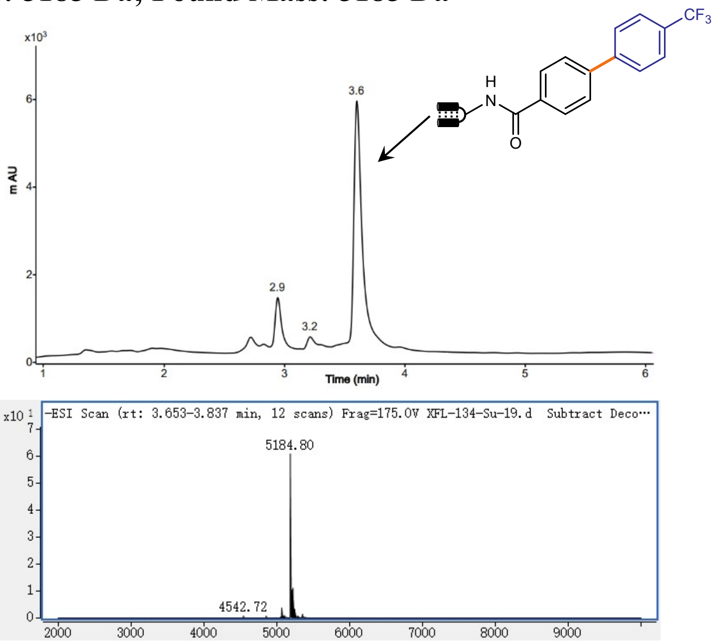

UPLC chromatograph and deconvoluted MS of **3ac.**

Conversion: 59%

Calculated Mass: 5162 Da; Found Mass: 5162 Da

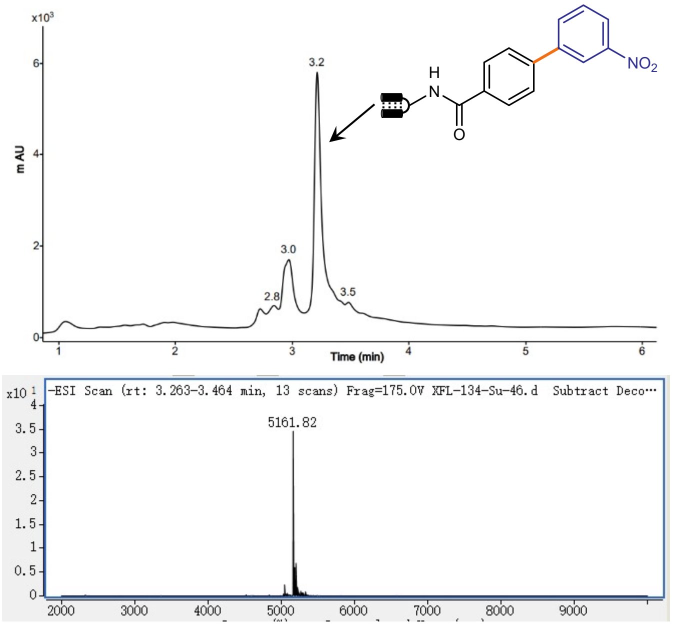

UPLC chromatograph and deconvoluted MS of **3ad.**

Conversion: 65%

Calculated Mass: 5201 Da; Found Mass: 5201 Da

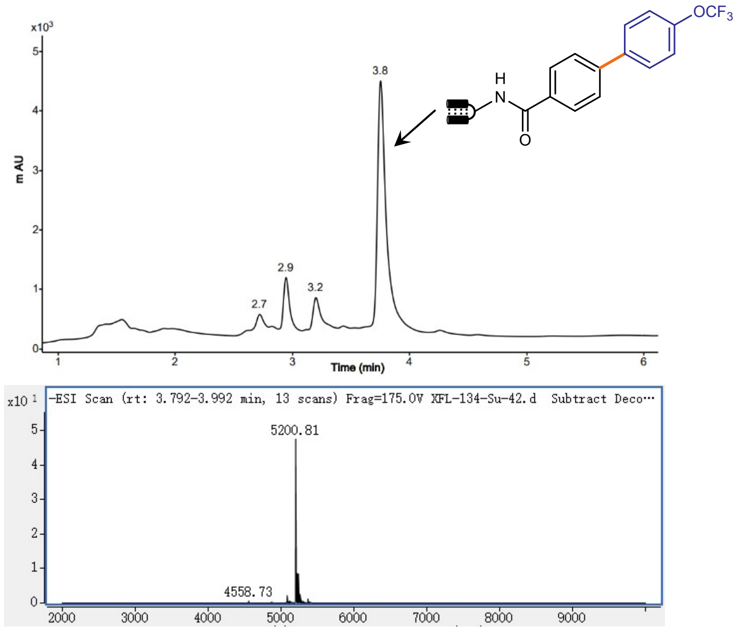

UPLC chromatograph and deconvoluted MS of **3ae.**

Conversion: 57%

Calculated Mass: 5149 Da; Found Mass: 5149 Da

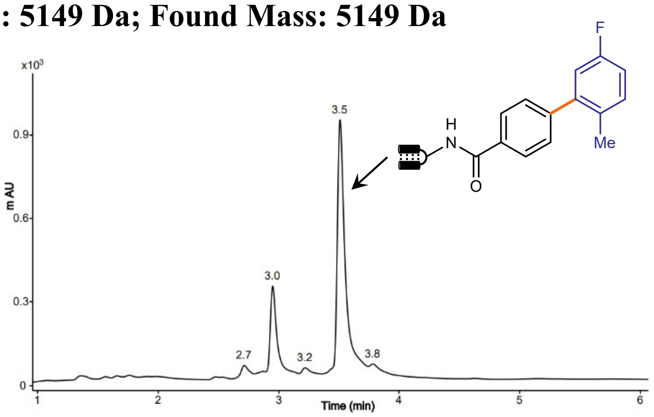

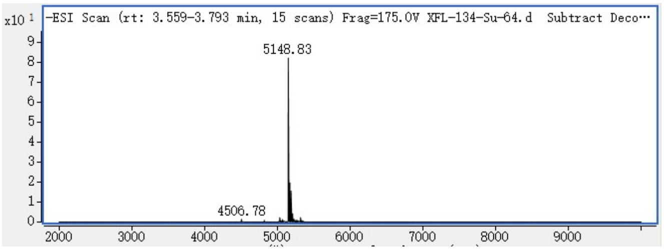

UPLC chromatograph and deconvoluted MS of **3af.**

Conversion: 67%

Calculated Mass: 5165 Da; Found Mass: 5165 Da

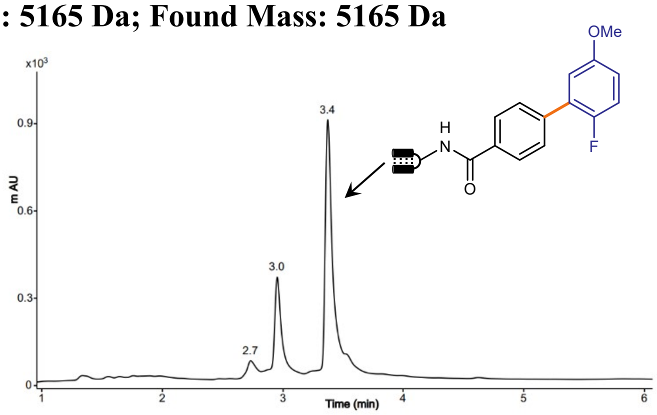

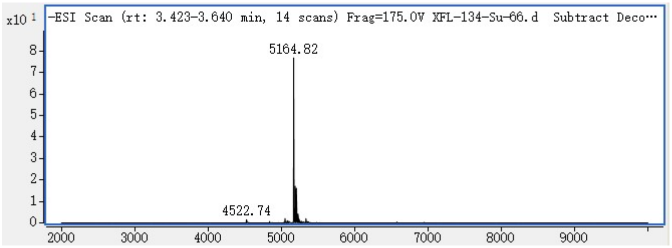

UPLC chromatograph and deconvoluted MS of **3ag.**

Conversion: 75%

Calculated Mass: 5185 Da; Found Mass: 5185 Da

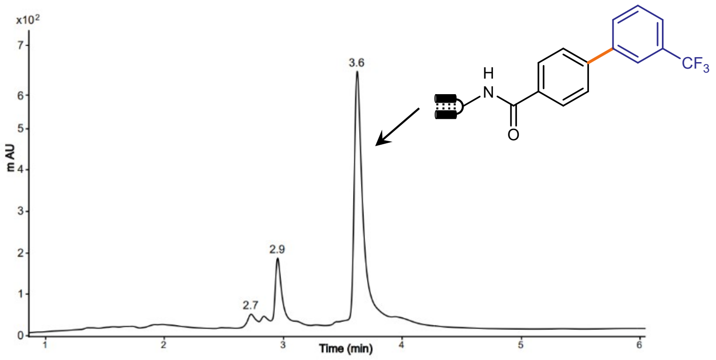

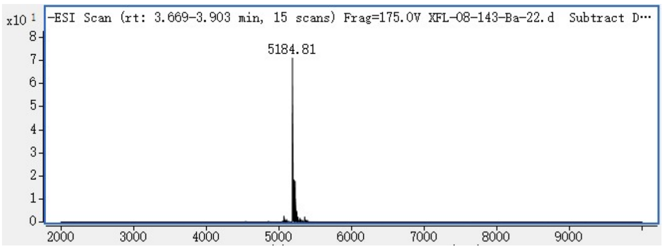

8.4 Substrate scope of DNA-conjugated arylamines for the On-DNA Suzuki-Miyaura coupling

UPLC chromatograph and deconvoluted MS of **3k.**

Conversion: 78%

Calculated Mass: 5175 Da; Found Mass: 5175 Da

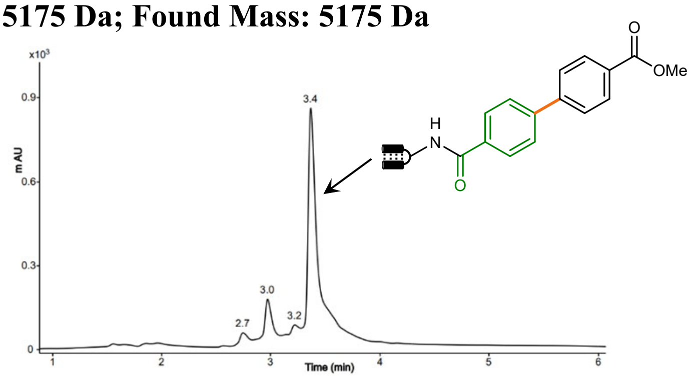

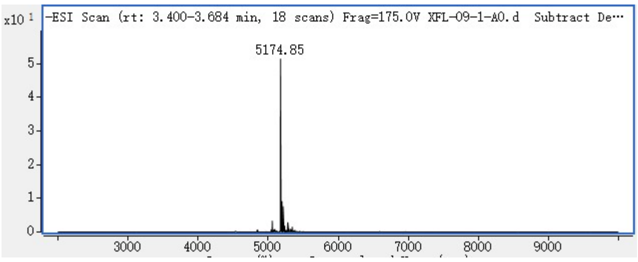

UPLC chromatograph and deconvoluted MS of **3ah.**

Conversion: 79%

Calculated Mass: 5175 Da; Found Mass: 5175 Da

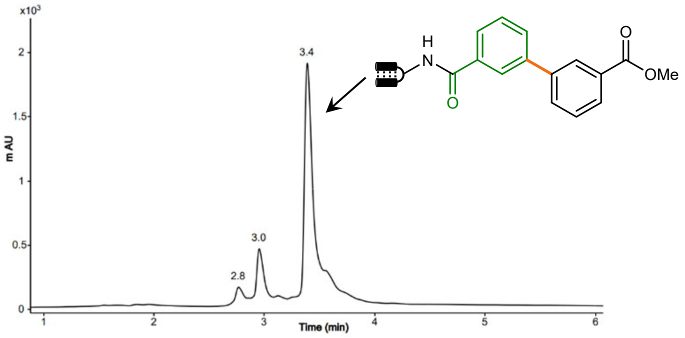

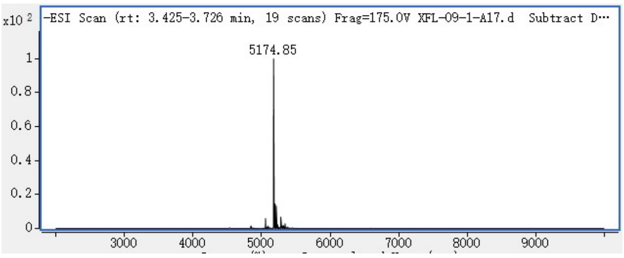

UPLC chromatograph and deconvoluted MS of **3ai.**

Conversion: 73%

Calculated Mass: 5189 Da; Found Mass: 5189 Da

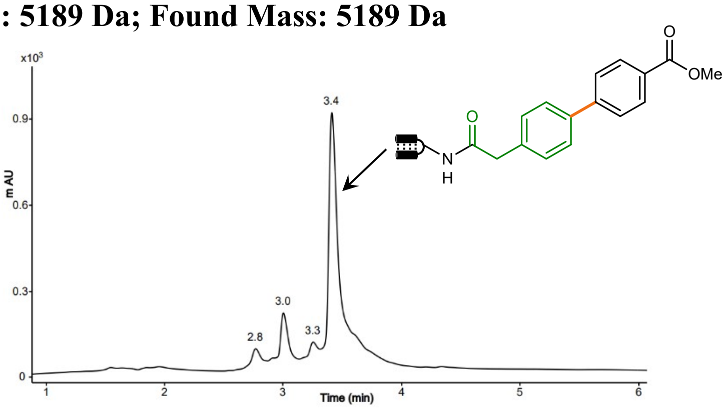

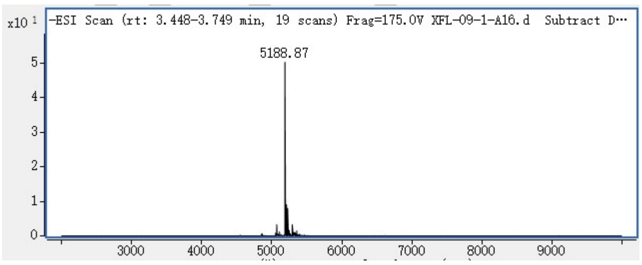

UPLC chromatograph and deconvoluted MS of **3aj.**

Conversion: 67%

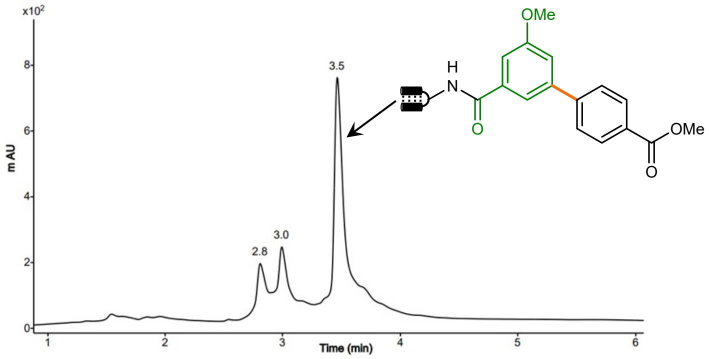

Calculated Mass: 5205 Da; Found Mass: 5205 Da

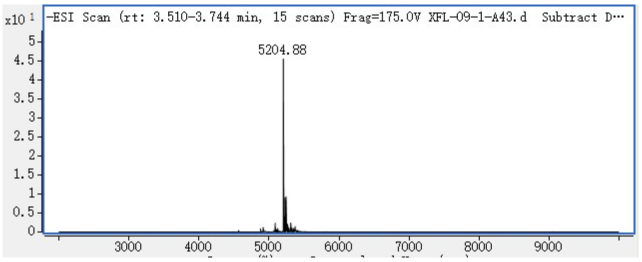

UPLC chromatograph and deconvoluted MS of **3ak.**

Conversion: 62%

Calculated Mass: 5243 Da; Found Mass: 5243 Da

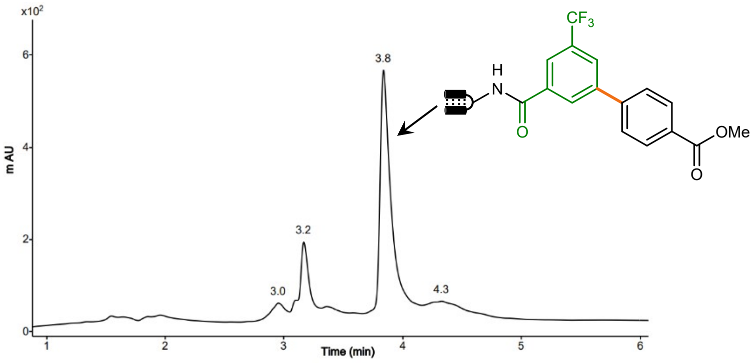

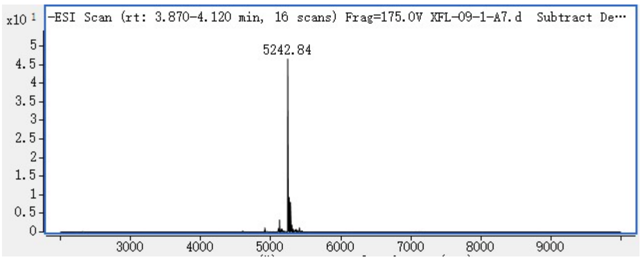

UPLC chromatograph and deconvoluted MS of **3al.**

Conversion: 80%

Calculated Mass: 5205 Da; Found Mass: 5205 Da

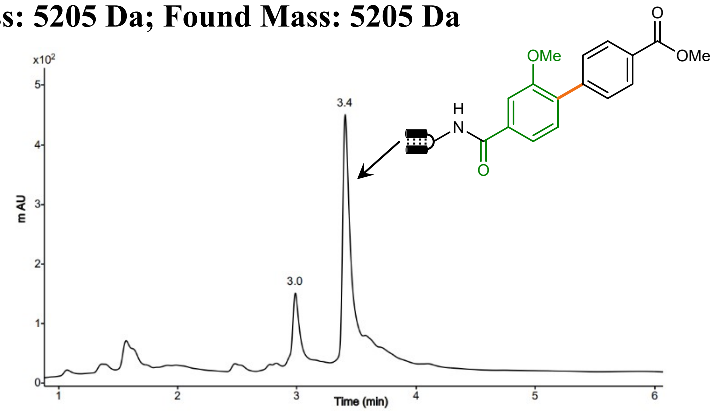

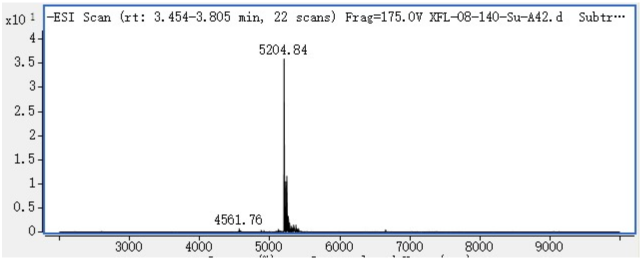

UPLC chromatograph and deconvoluted MS of **3am.**

Conversion: 66%

Calculated Mass: 5233 Da; Found Mass: 5233 Da

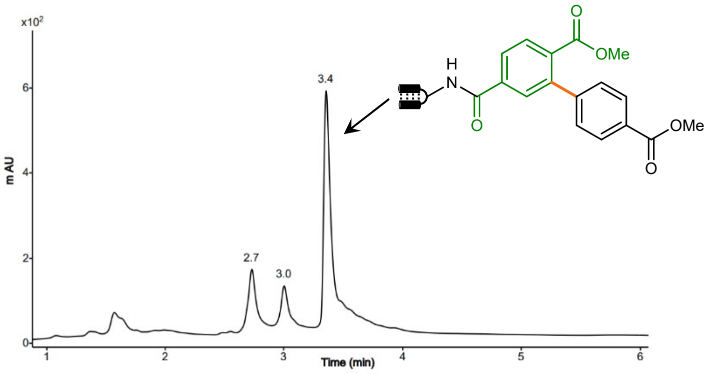

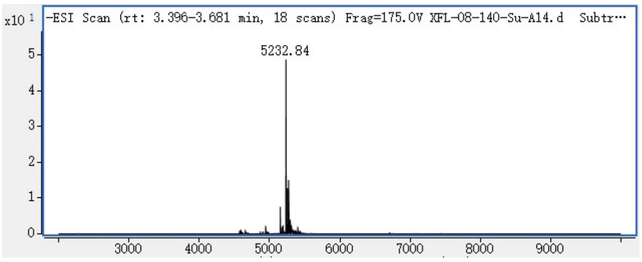

UPLC chromatograph and deconvoluted MS of **3an.**

Conversion: 70%

Calculated Mass: 5251 Da; Found Mass: 5251 Da

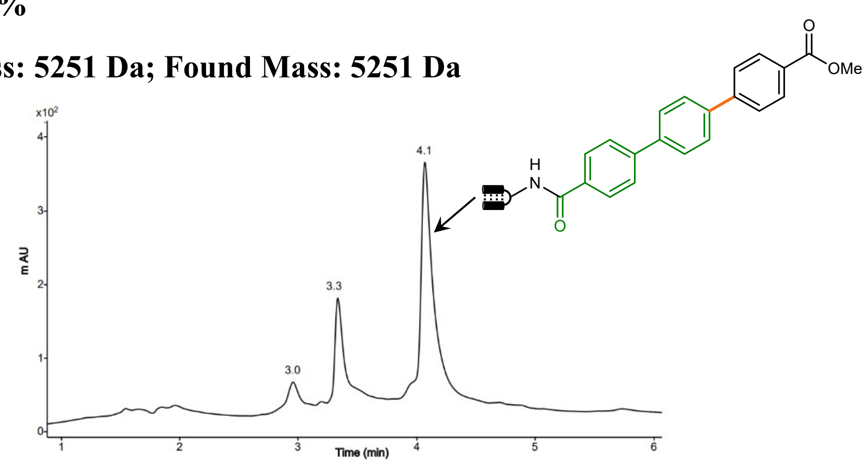

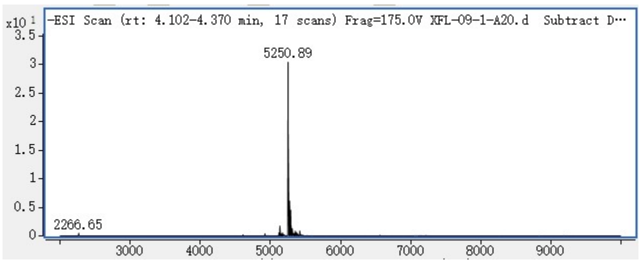

UPLC chromatograph and deconvoluted MS of **3ao.**

Conversion: 66%

Calculated Mass: 5225 Da; Found Mass: 5225 Da

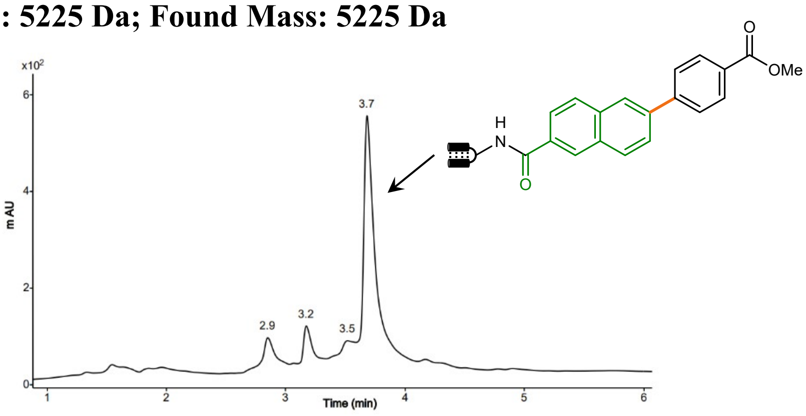

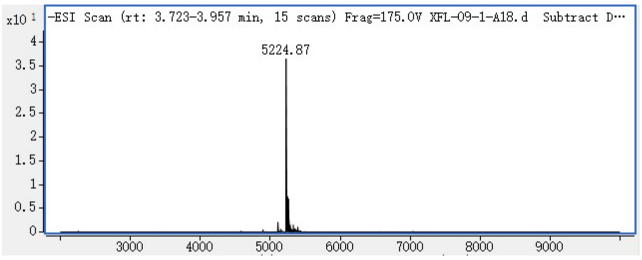

UPLC chromatograph and deconvoluted MS of **3ap.**

Conversion: 67%

Calculated Mass: 5211 Da; Found Mass: 5211 Da

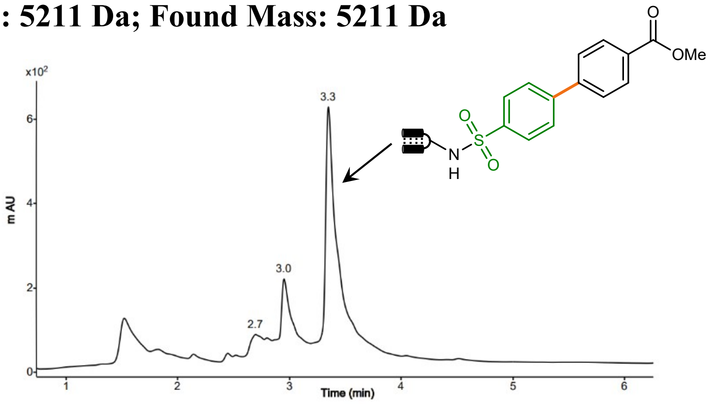

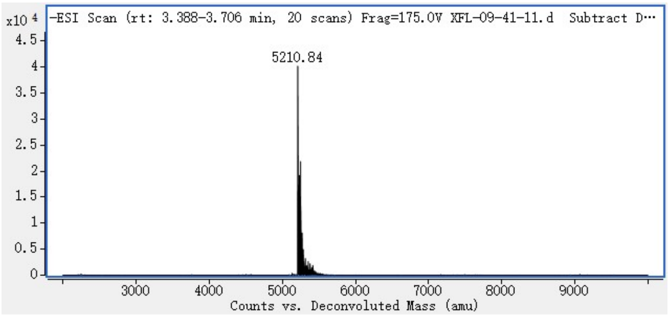

UPLC chromatograph and deconvoluted MS of **3aq.**

Conversion: 85%

Calculated Mass: 5193 Da; Found Mass: 5193 Da

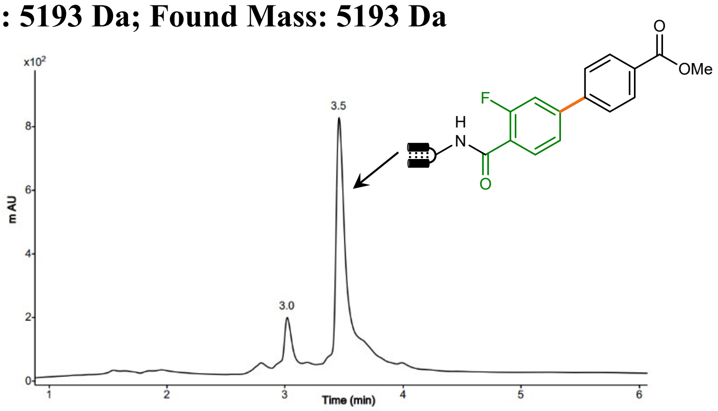

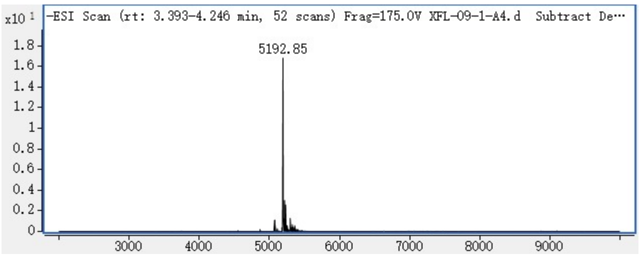

UPLC chromatograph and deconvoluted MS of **3ar.**

Conversion: 72%

Calculated Mass: 5207 Da; Found Mass: 5207 Da

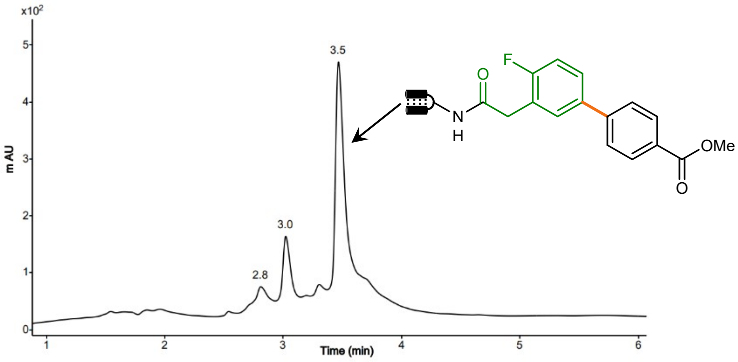

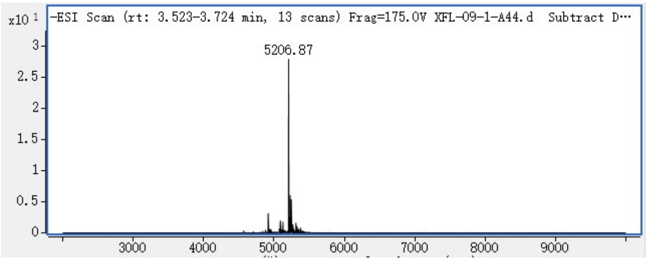

UPLC chromatograph and deconvoluted MS of **3as.**

Conversion: 56%

Calculated Mass: 5203 Da; Found Mass: 5203 Da

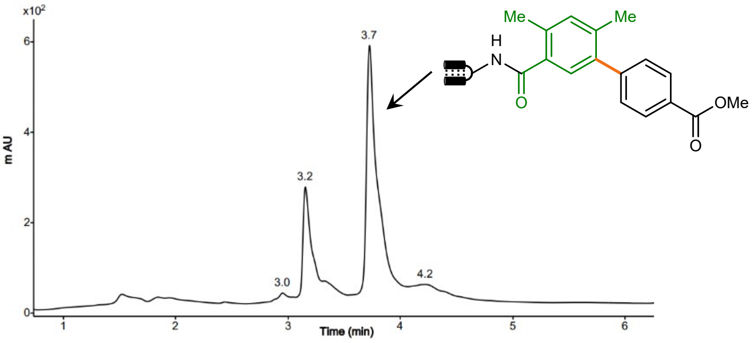

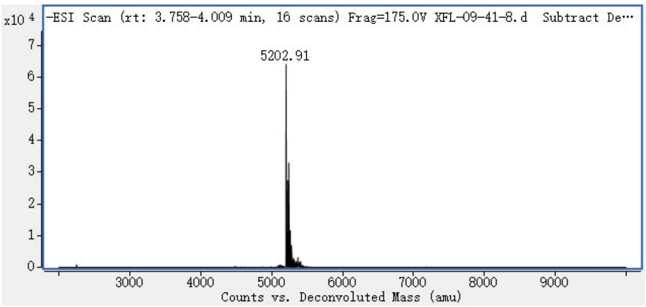

UPLC chromatograph and deconvoluted MS of **3at.**

Conversion: 51%

Calculated Mass: 5207 Da; Found Mass: 5207 Da

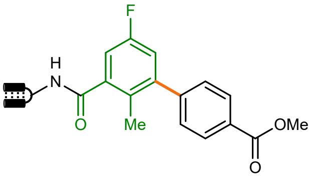

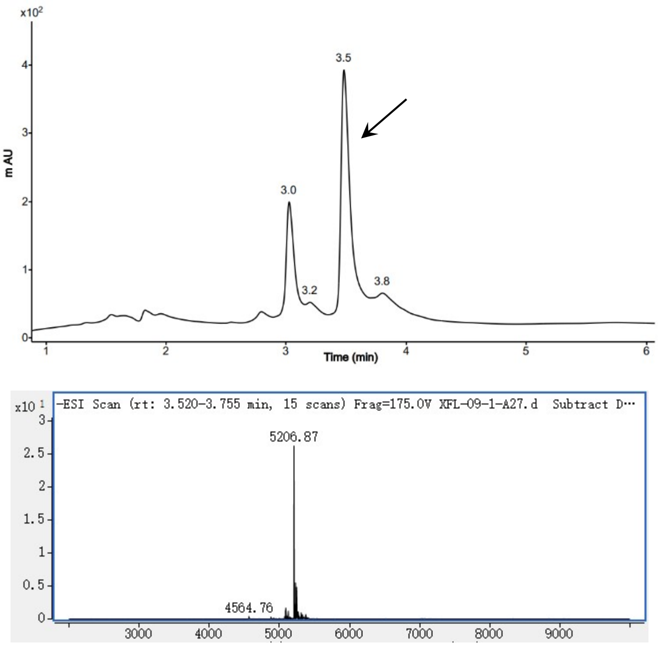

UPLC chromatograph and deconvoluted MS of **3au.**

Conversion: 61%

Calculated Mass: 5205 Da; Found Mass: 5205 Da

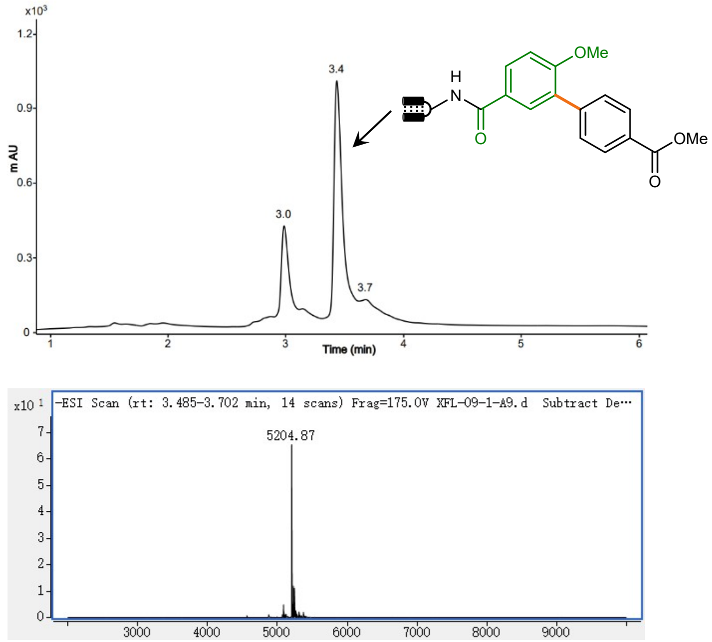

UPLC chromatograph and deconvoluted MS of **3av.**

Conversion: 65%

Calculated Mass: 5193 Da; Found Mass: 5193 Da

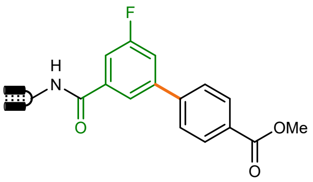

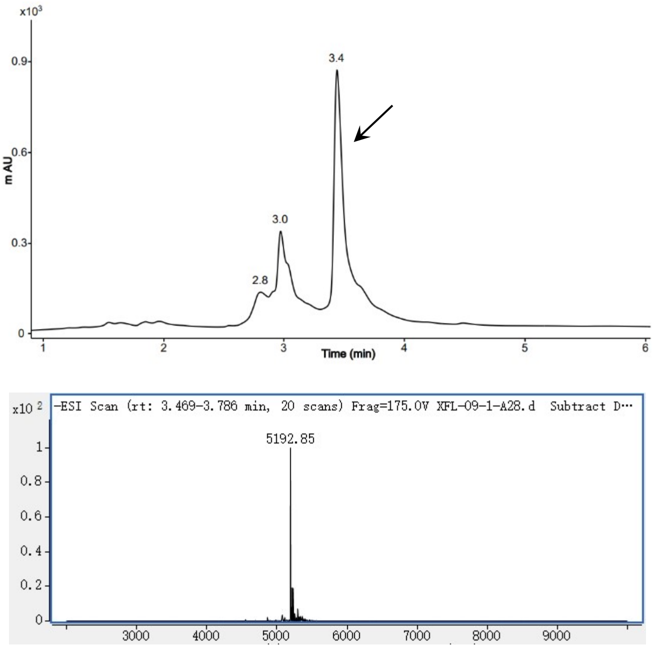

UPLC chromatograph and deconvoluted MS of **3aw.**

Conversion: 54%

Calculated Mass: 5207 Da; Found Mass: 5207 Da

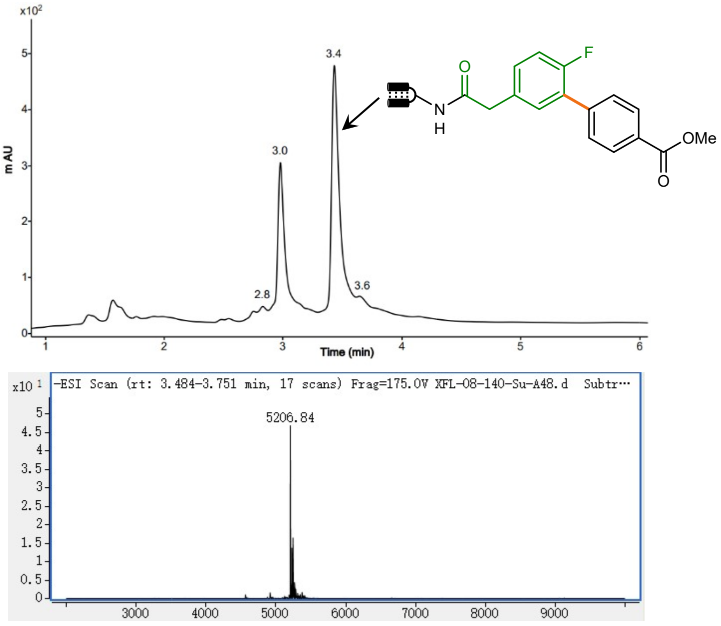

UPLC chromatograph and deconvoluted MS of **3ax.**

Conversion: 67%

Calculated Mass: 5205 Da; Found Mass: 5205 Da

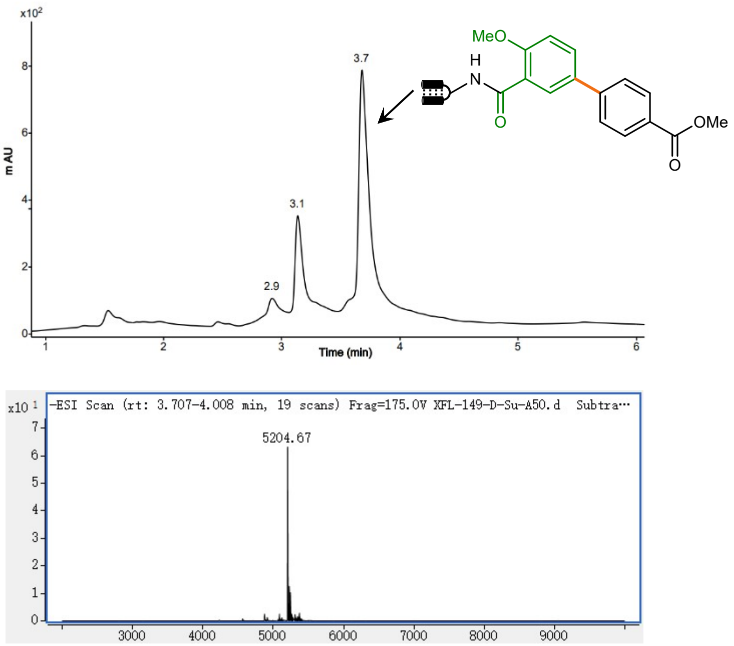

UPLC chromatograph and deconvoluted MS of **3ay.**

Conversion: 56%

Calculated Mass: 5193 Da; Found Mass: 5193 Da

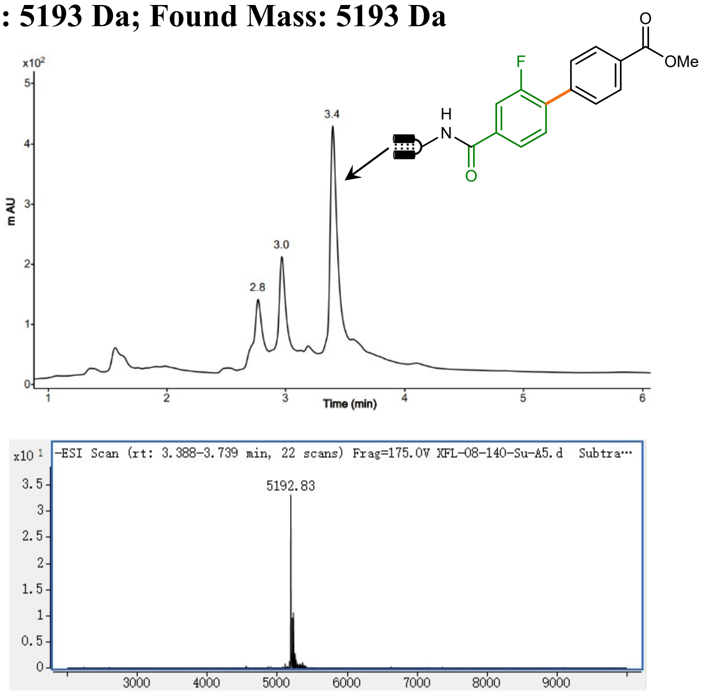

UPLC chromatograph and deconvoluted MS of **3az.**

Conversion: 79%

Calculated Mass: 5189 Da; Found Mass: 5189 Da

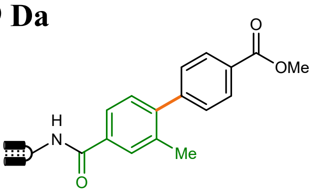

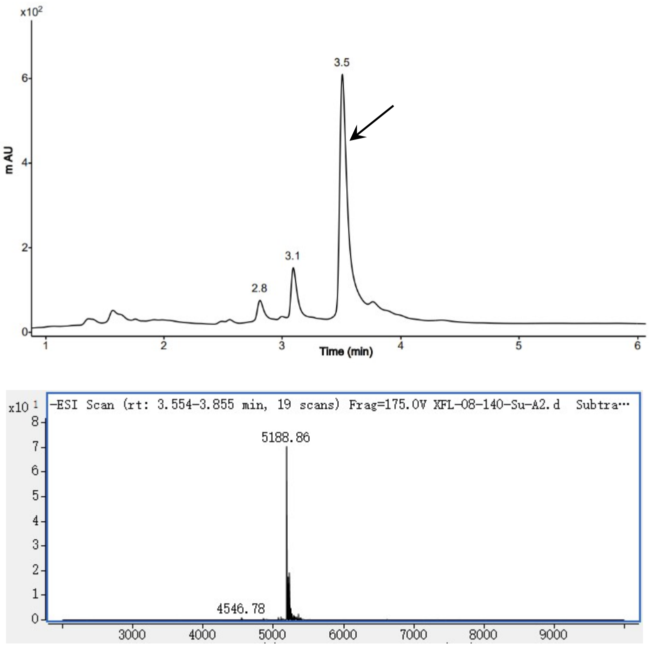

UPLC chromatograph and deconvoluted MS of **3ba.**

Conversion: 51%

Calculated Mass: 5189 Da; Found Mass: 5189 Da

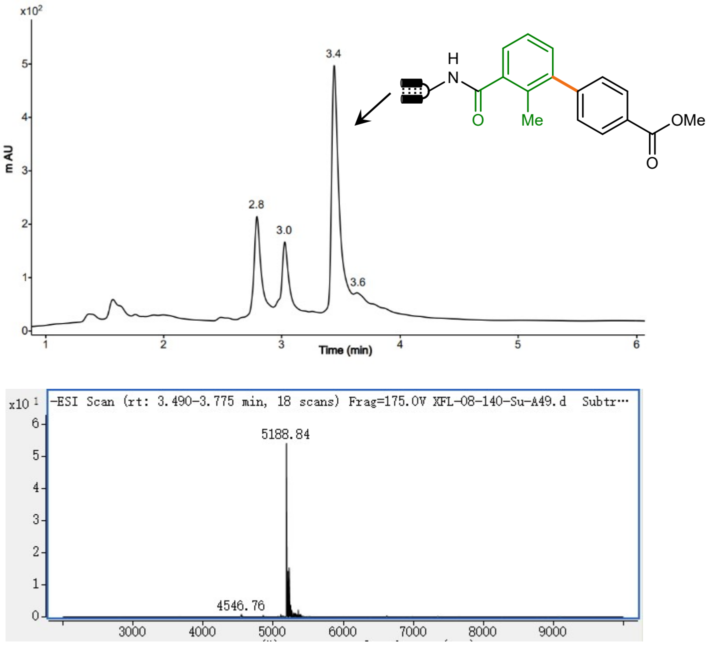

8.5 Substrate scope of olefins for the on-DNA Heck reaction with 1a

UPLC chromatograph and deconvoluted MS of **5a.**

Conversion: 83%

Calculated Mass: 5143 Da; Found Mass: 5143 Da

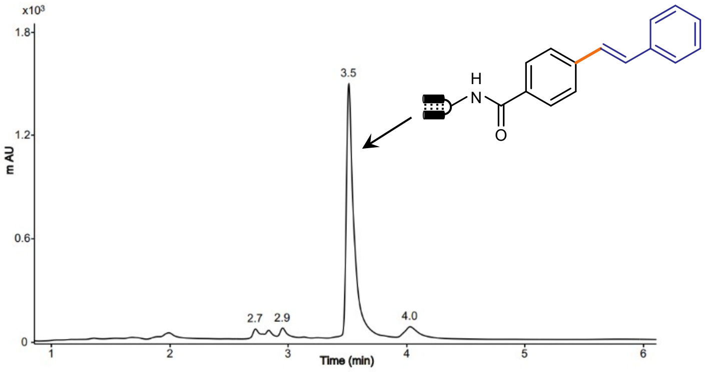

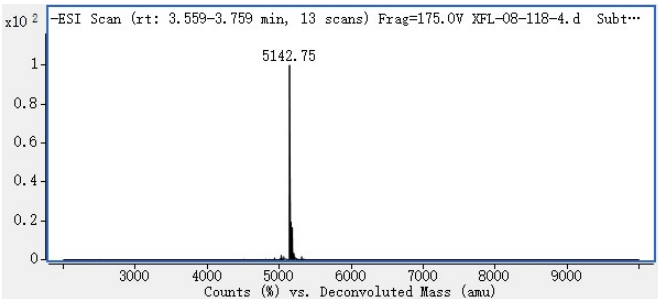

UPLC chromatograph and deconvoluted MS of **5b.**

Conversion: 84%

Calculated Mass: 5157 Da; Found Mass: 5157 Da

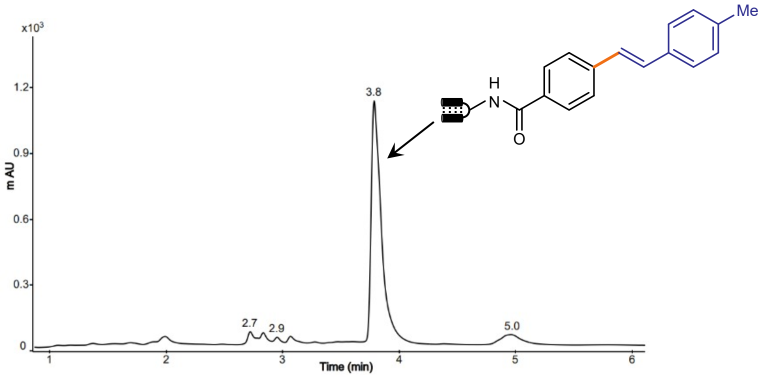

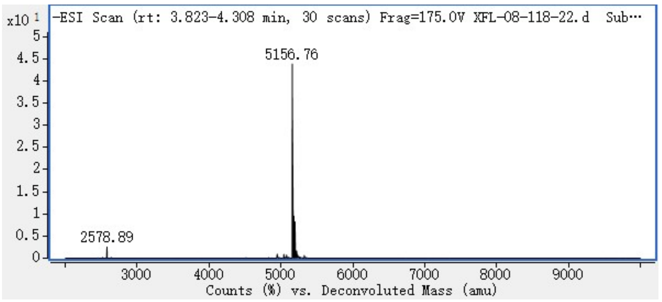

UPLC chromatograph and deconvoluted MS of **5c.**

Conversion: 78%

Calculated Mass: 5173 Da; Found Mass: 5173 Da

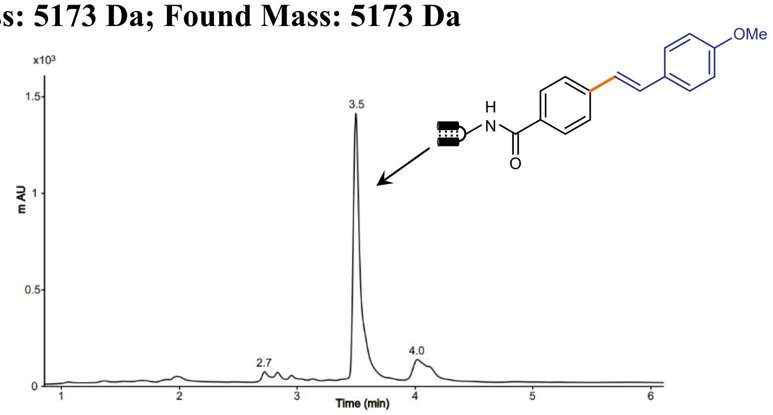

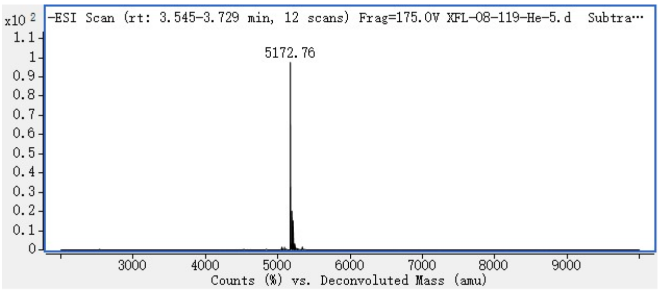

UPLC chromatograph and deconvoluted MS of **5d.**

Conversion: 83%

Calculated Mass: 5161 Da; Found Mass: 5161 Da

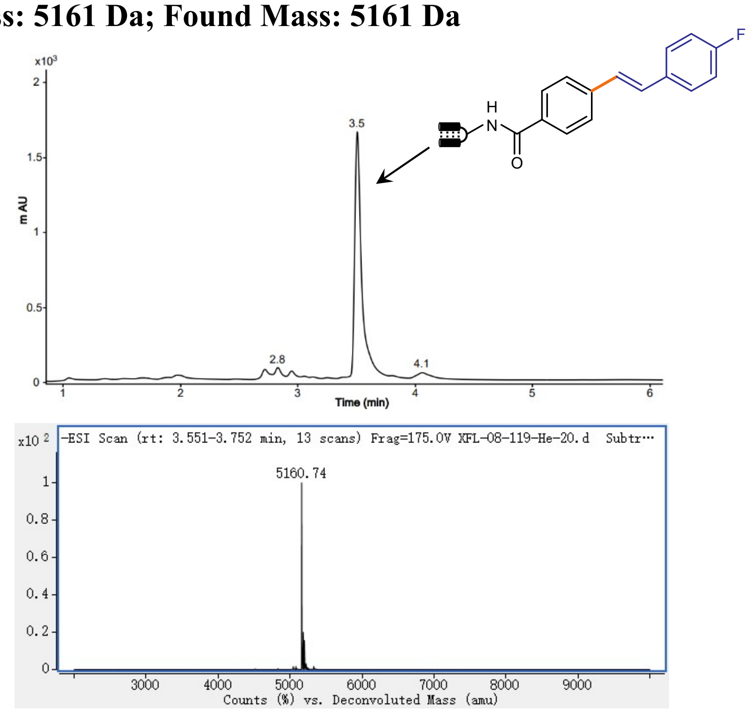

UPLC chromatograph and deconvoluted MS of **5e.**

Conversion: 83%

Calculated Mass: 5177 Da; Found Mass: 5177 Da

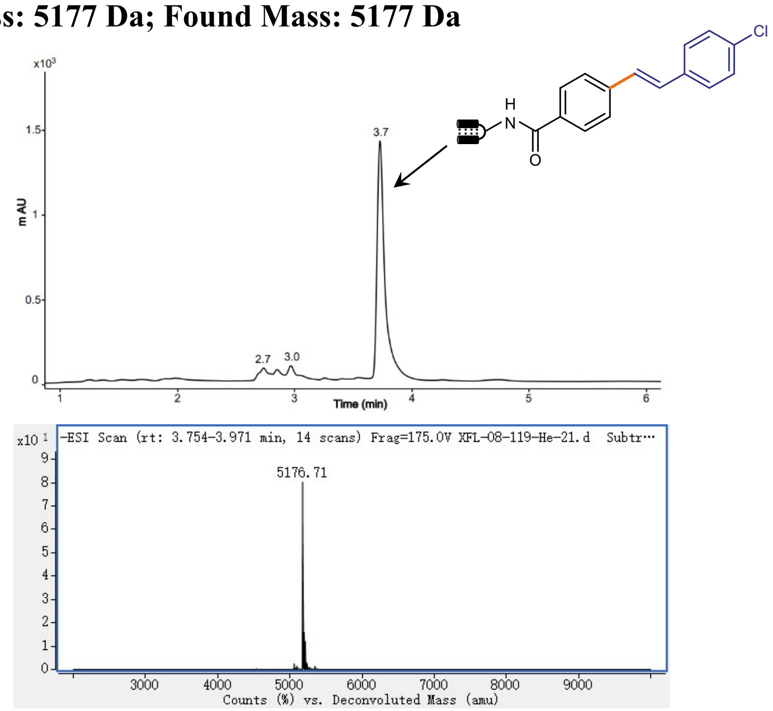

UPLC chromatograph and deconvoluted MS of **5f.**

Conversion: 84%

Calculated Mass: 5222 Da; Found Mass: 5222 Da

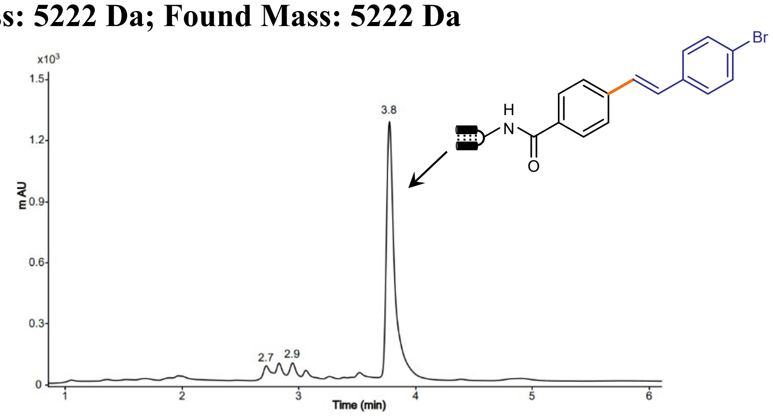

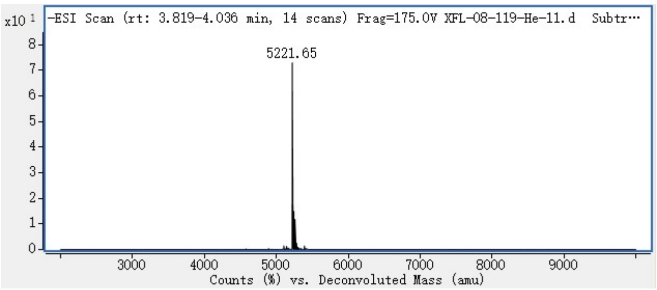

UPLC chromatograph and deconvoluted MS of **5g.**

Conversion: 80%

Calculated Mass: 5188 Da; Found Mass: 5188 Da

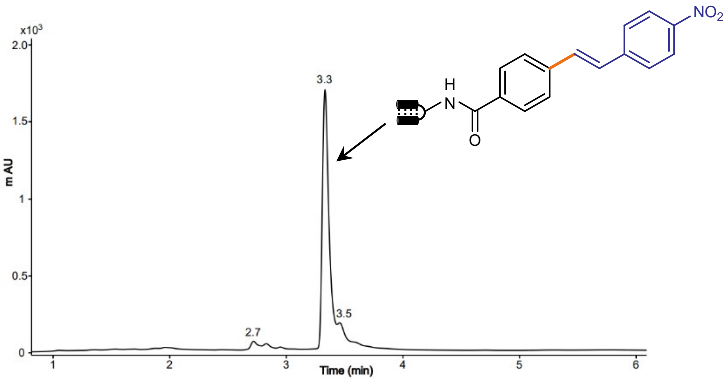

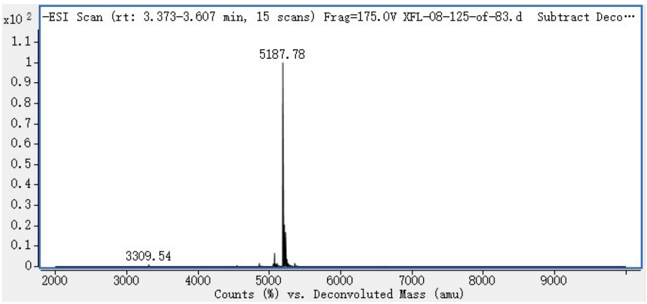

UPLC chromatograph and deconvoluted MS of **5h.**

Conversion: 91%

Calculated Mass: 5157 Da; Found Mass: 5157 Da

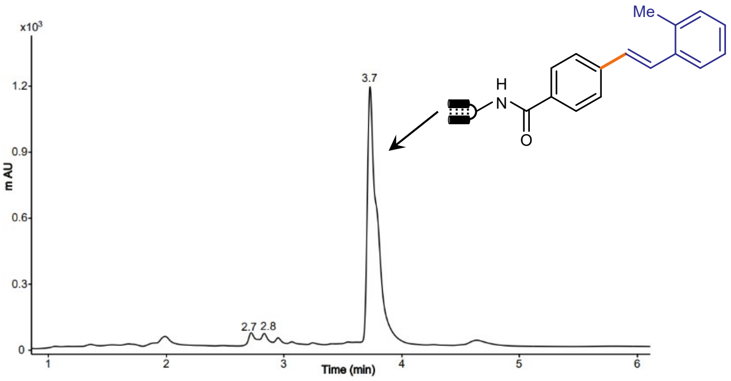

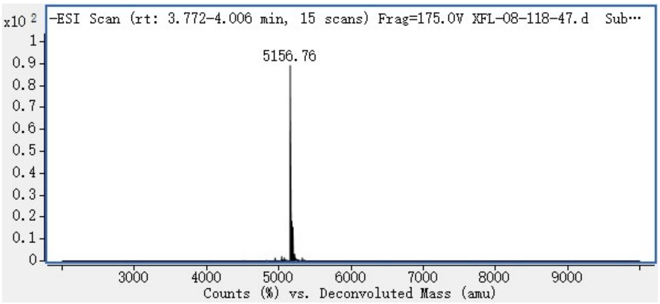

UPLC chromatograph and deconvoluted MS of **5i.**

Conversion: 87%

Calculated Mass: 5157 Da; Found Mass: 5157 Da

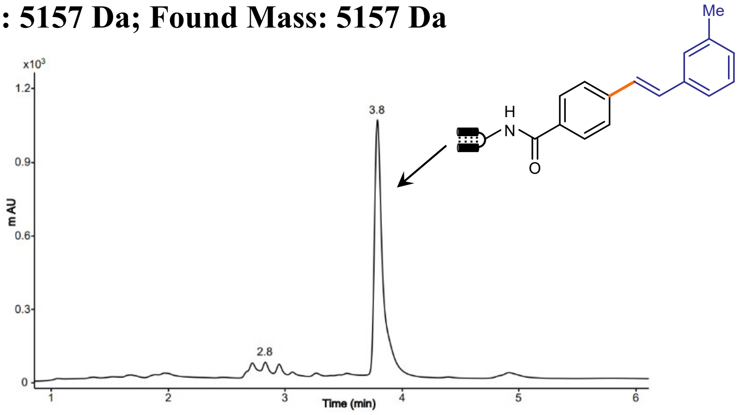

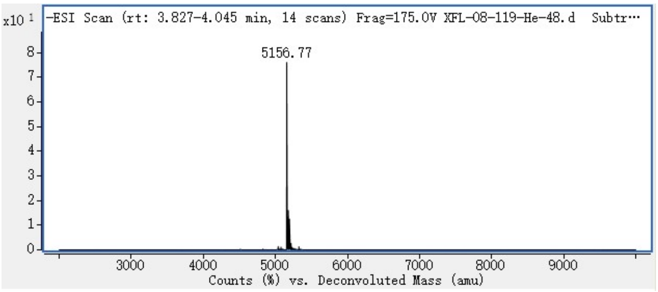

UPLC chromatograph and deconvoluted MS of **5j.**

Conversion: 91%

Calculated Mass: 5222 Da; Found Mass: 5222 Da

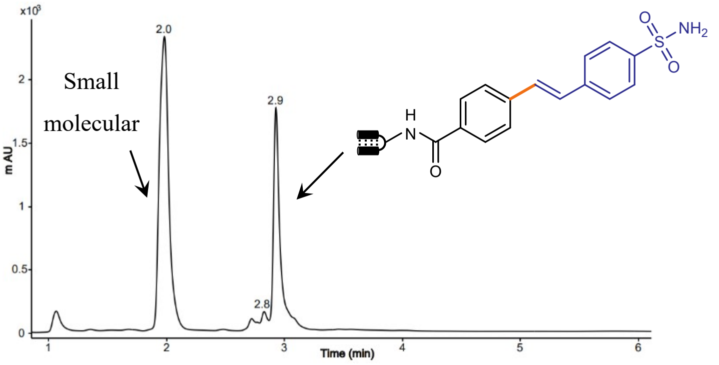

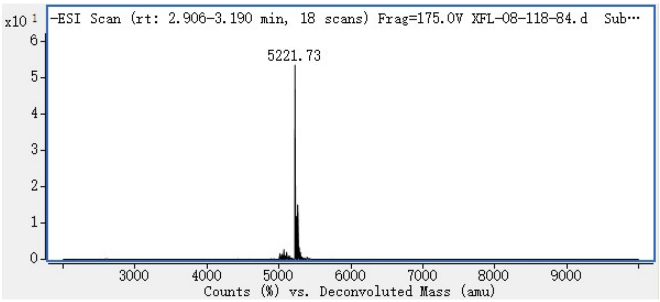

UPLC chromatograph and deconvoluted MS of **5k.**

Conversion: 83%

Calculated Mass: 5168 Da; Found Mass: 5168 Da

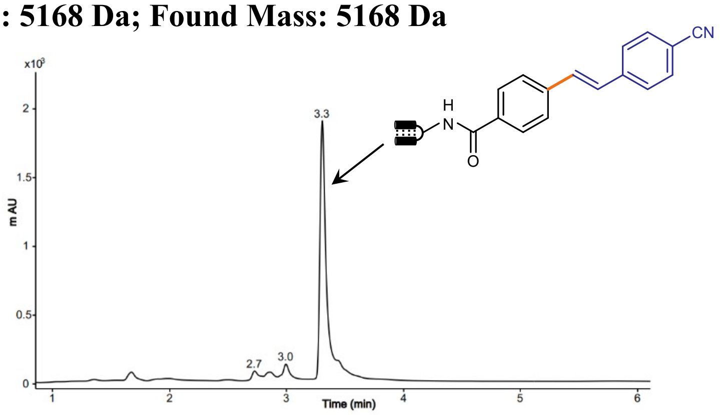

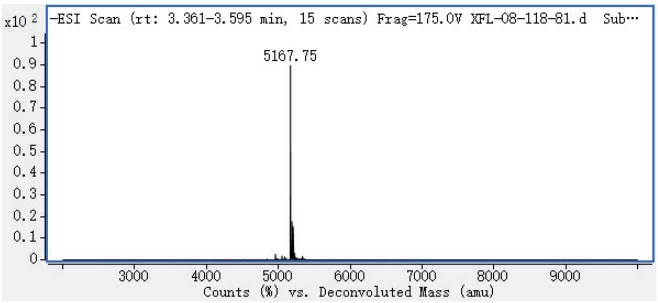

UPLC chromatograph and deconvoluted MS of **5l.**

Conversion: 63%

Calculated Mass: 5159 Da; Found Mass: 5159 Da

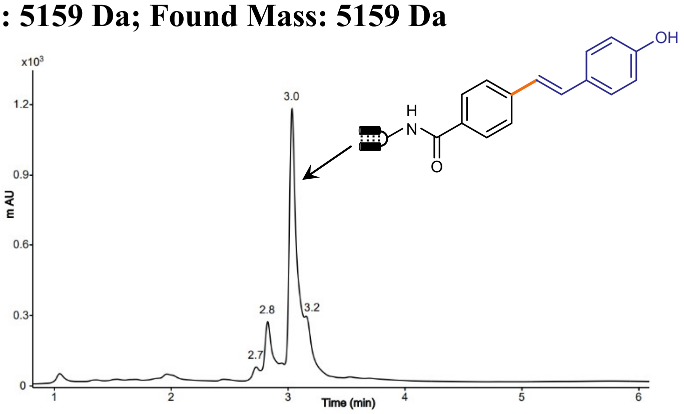

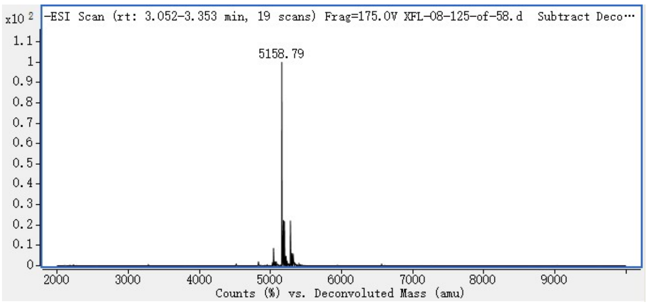

UPLC chromatograph and deconvoluted MS of **5m.**

Conversion: 81%

Calculated Mass: 5187 Da; Found Mass: 5187 Da

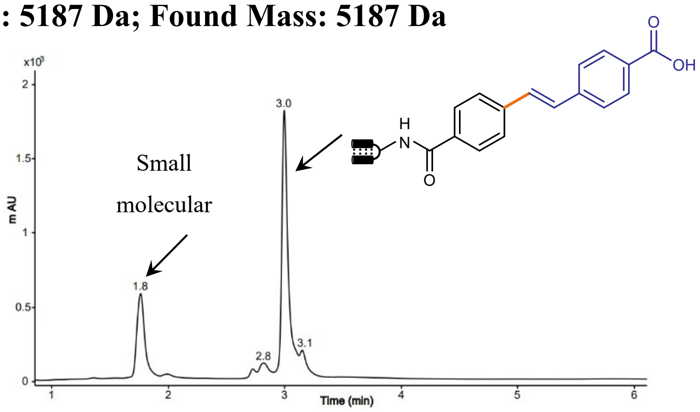

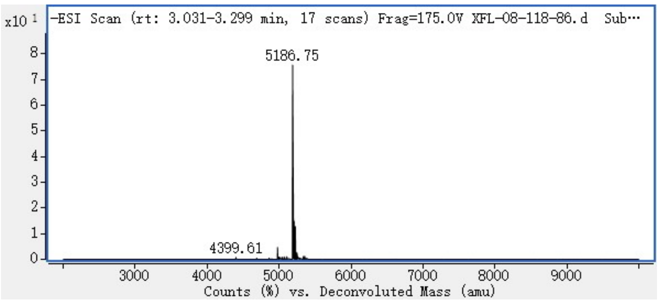

UPLC chromatograph and deconvoluted MS of **5n.**

Conversion: 84%

Calculated Mass: 5171 Da; Found Mass: 5171 Da

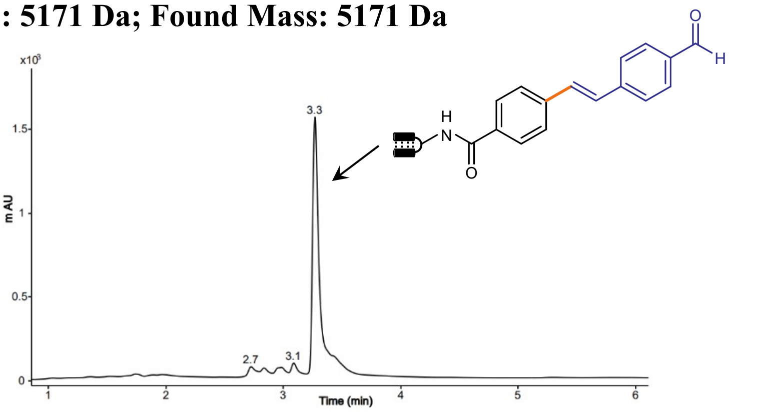

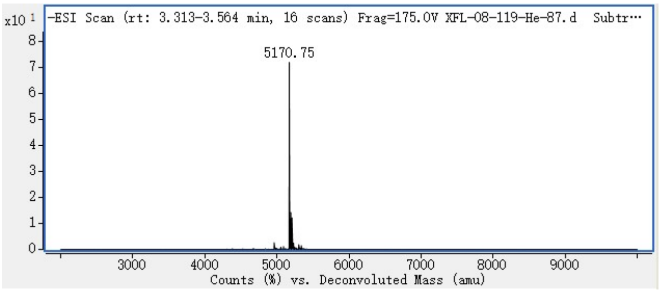

UPLC chromatograph and deconvoluted MS of **5o.**

Conversion: 84%

Calculated Mass: 5201 Da; Found Mass: 5201 Da

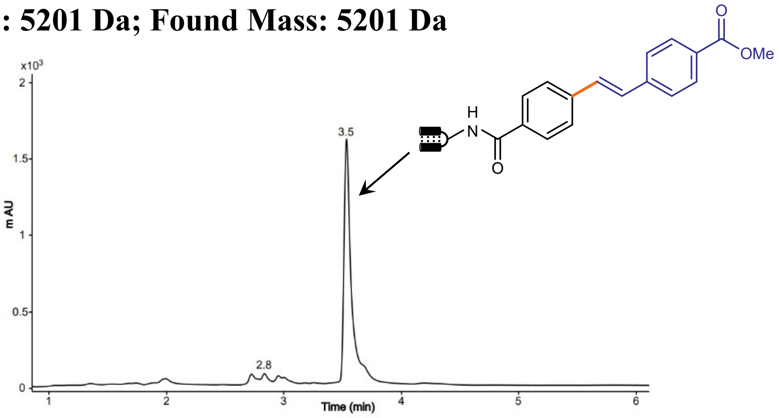

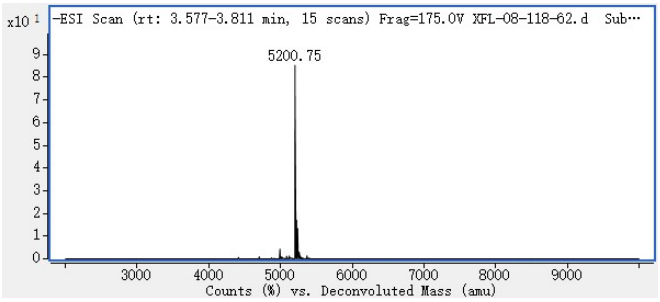

UPLC chromatograph and deconvoluted MS of **5p.**

Conversion: 80%

Calculated Mass: 5201 Da; Found Mass: 5201 Da

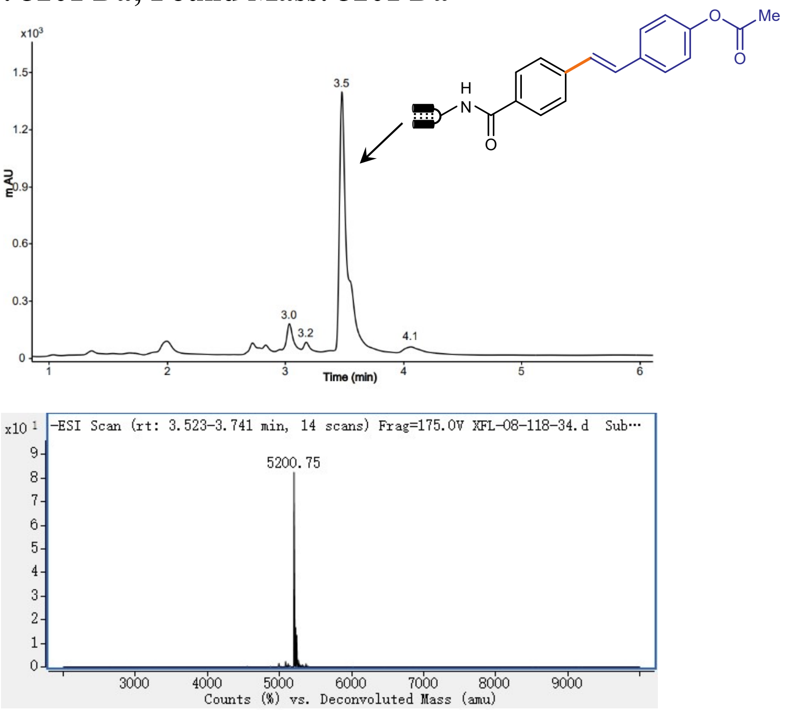

UPLC chromatograph and deconvoluted MS of **5q.**

Conversion: 85%

Calculated Mass: 5329 Da; Found Mass: 5329 Da

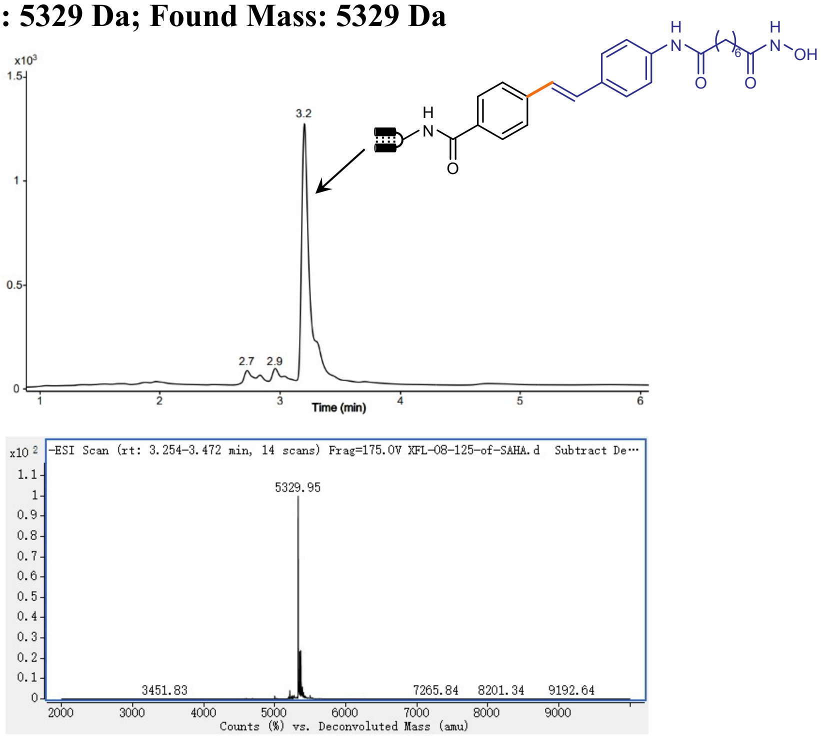

UPLC chromatograph and deconvoluted MS of **5r.**

Conversion: 94%

Calculated Mass: 5125 Da; Found Mass: 5126 Da

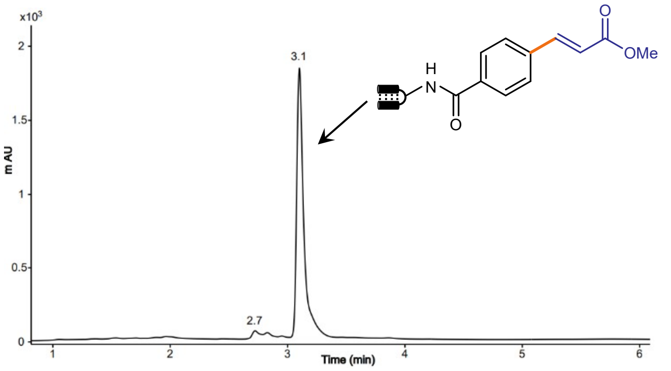

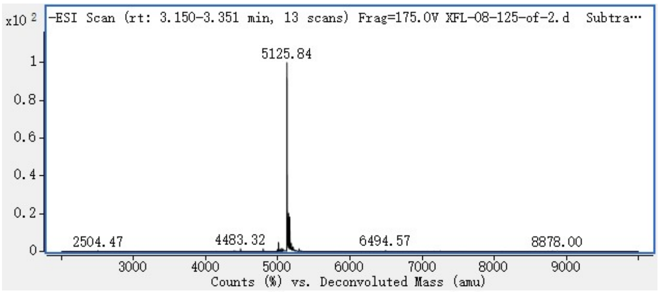

UPLC chromatograph and deconvoluted MS of **5s.**

Conversion: 87%

Calculated Mass: 5180 Da; Found Mass: 5180 Da

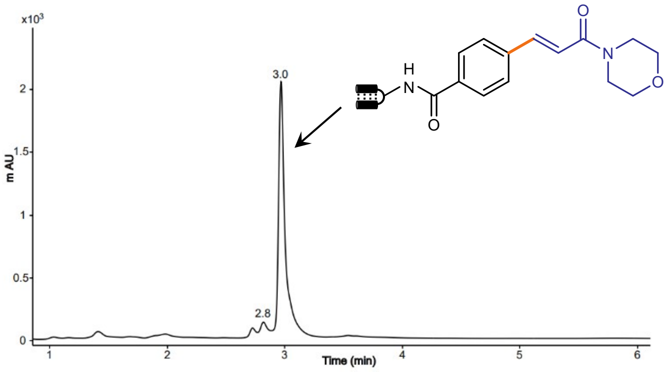

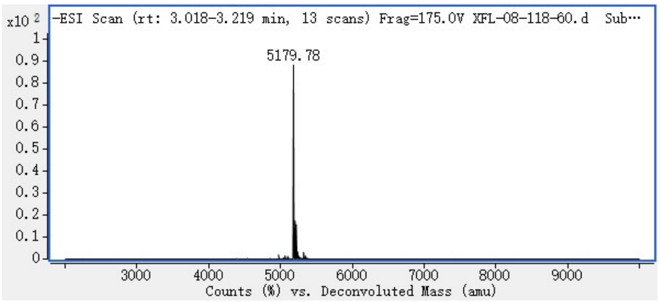

UPLC chromatograph and deconvoluted MS of **5t.**

Conversion: 89%

Calculated Mass: 5180 Da; Found Mass: 5180 Da

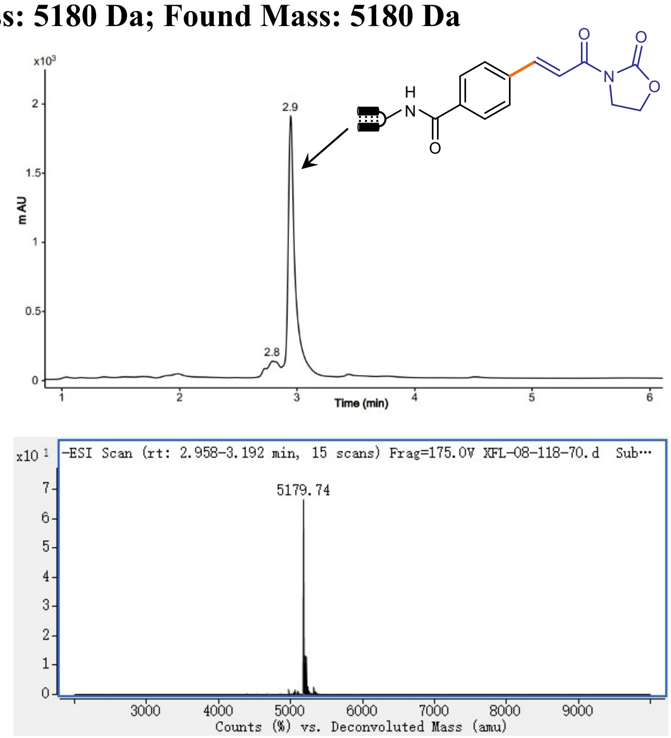

UPLC chromatograph and deconvoluted MS of **5u.**

Conversion: 82%

Calculated Mass: 5139 Da; Found Mass: 5139 Da

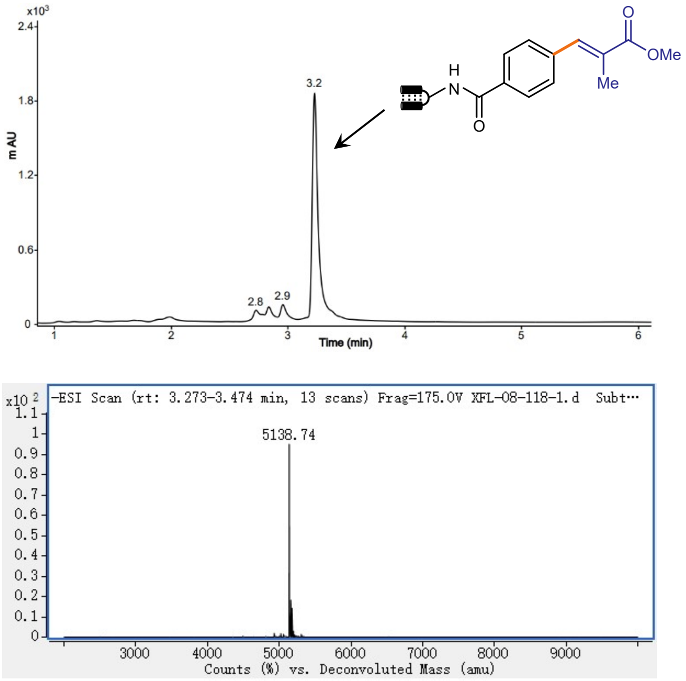

UPLC chromatograph and deconvoluted MS of **5v.**

Conversion: 77%

Calculated Mass: 5139 Da; Found Mass: 5139 Da

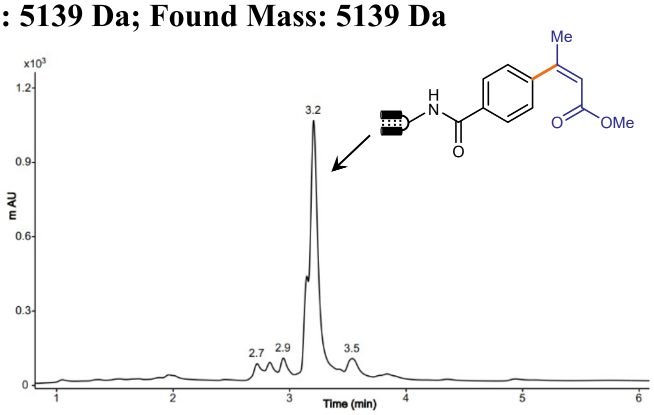

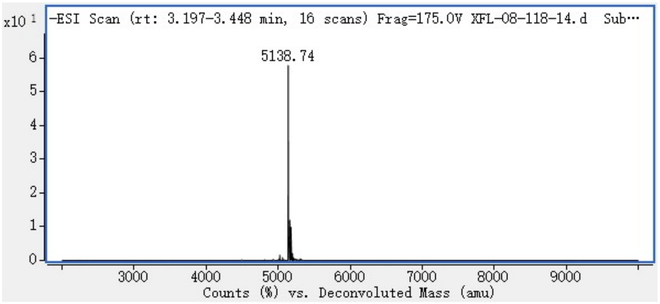

UPLC chromatograph and deconvoluted MS of **5w.**

Conversion: 88%

Calculated Mass: 5203 Da; Found Mass: 5203 Da

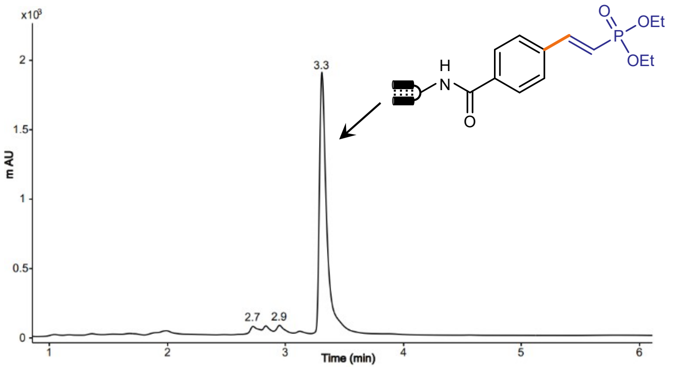

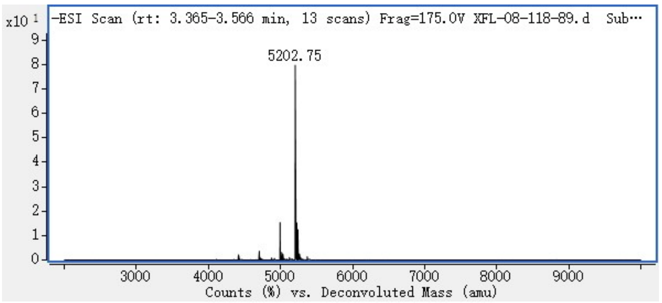

UPLC chromatograph and deconvoluted MS of **5x.**

Conversion: 86%

Calculated Mass: 5149 Da; Found Mass: 5149 Da

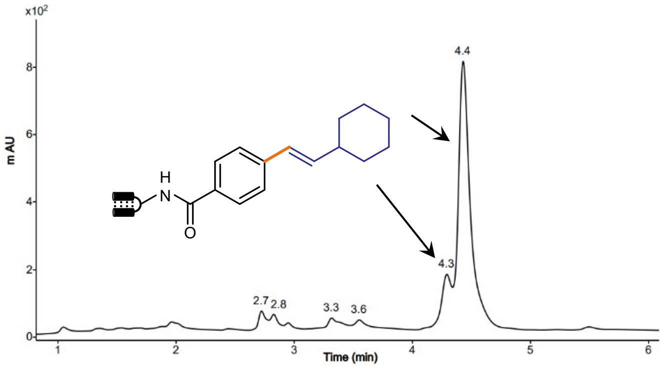

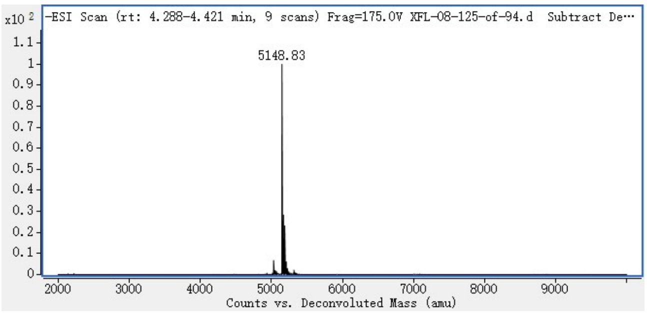

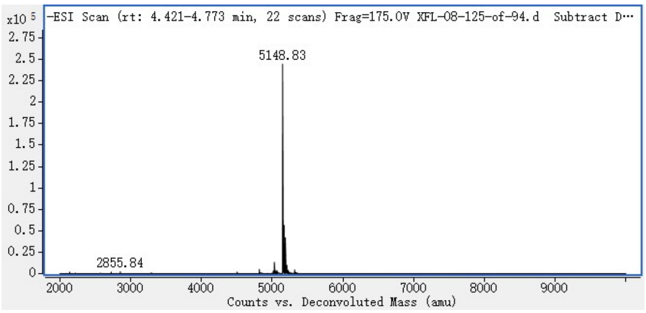

UPLC chromatograph and deconvoluted MS of **5y.**

Conversion: 90%

Calculated Mass: 5157 Da; Found Mass: 5157 Da

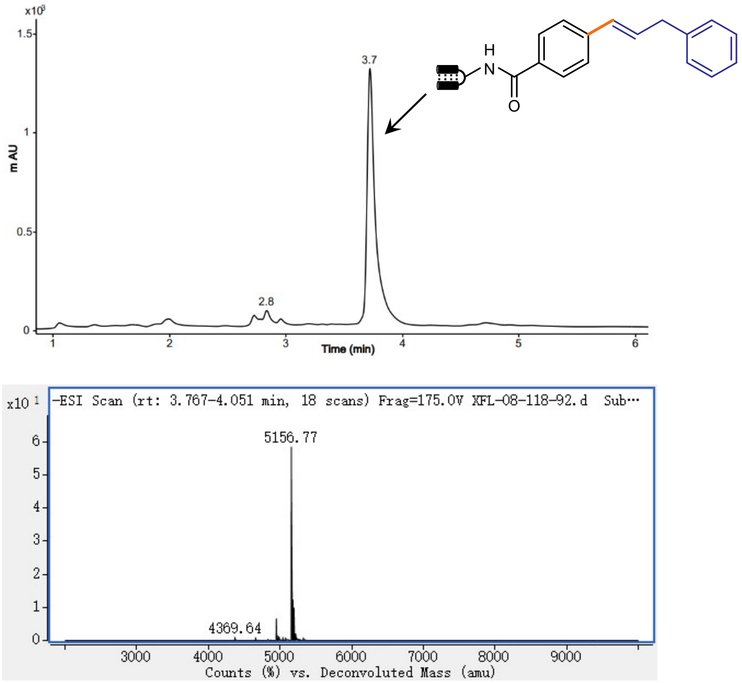

UPLC chromatograph and deconvoluted MS of **5z.**

Conversion: 88%

Calculated Mass: 5139 Da; Found Mass: 5139 Da

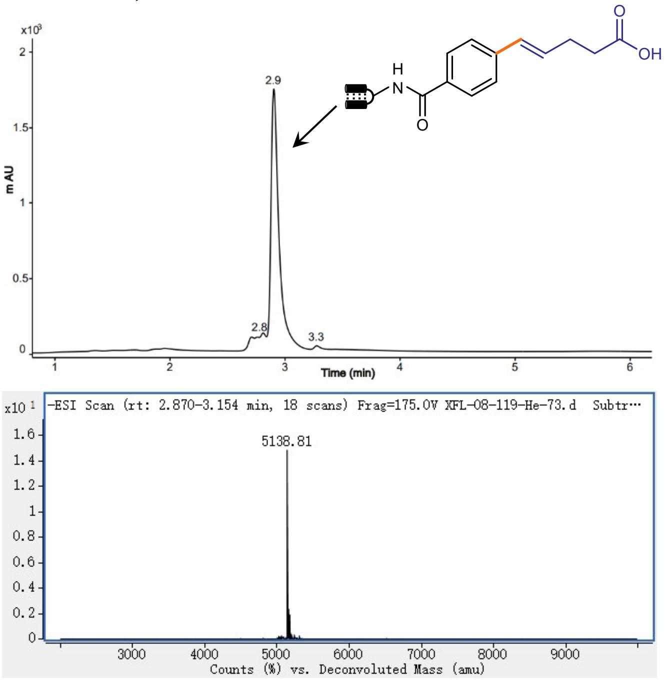

UPLC chromatograph and deconvoluted MS of **5aa.**

Conversion: 85%

Calculated Mass: 5177 Da; Found Mass: 5177 Da

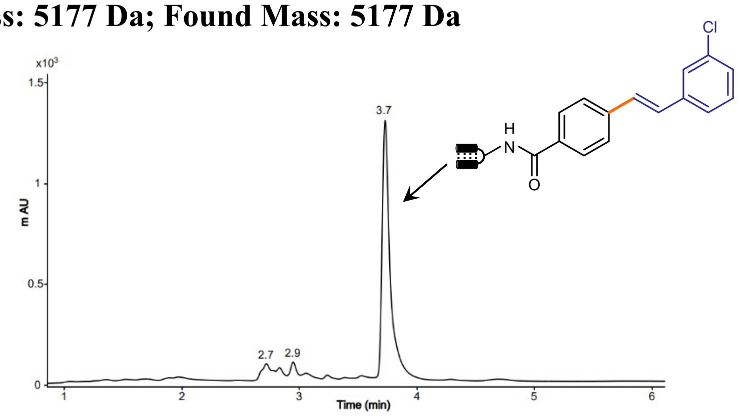

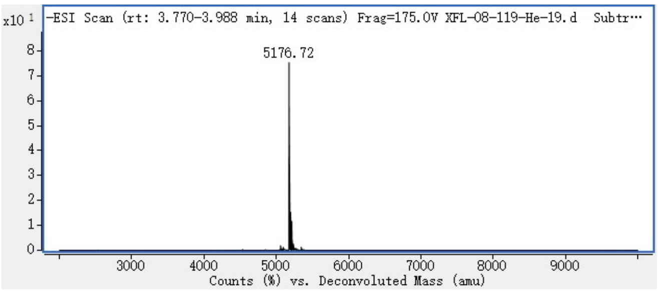

UPLC chromatograph and deconvoluted MS of **5ab.**

Conversion: 91%

Calculated Mass: 5177 Da; Found Mass: 5177 Da

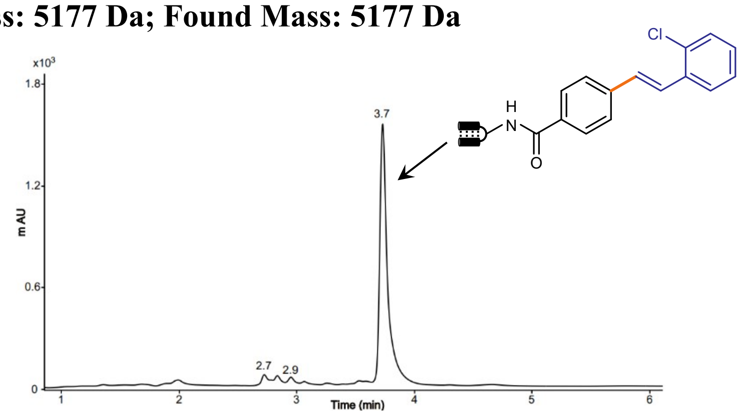

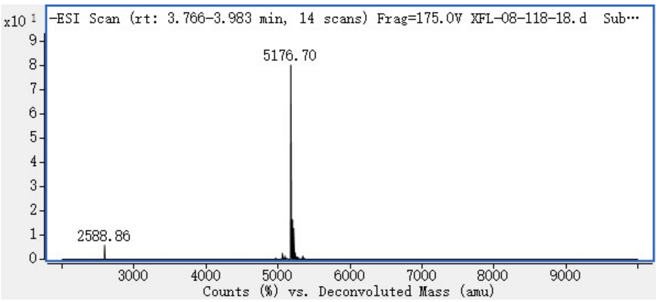

UPLC chromatograph and deconvoluted MS of **5ac.**

Conversion: 67%

Calculated Mass: 5222 Da; Found Mass: 5222 Da

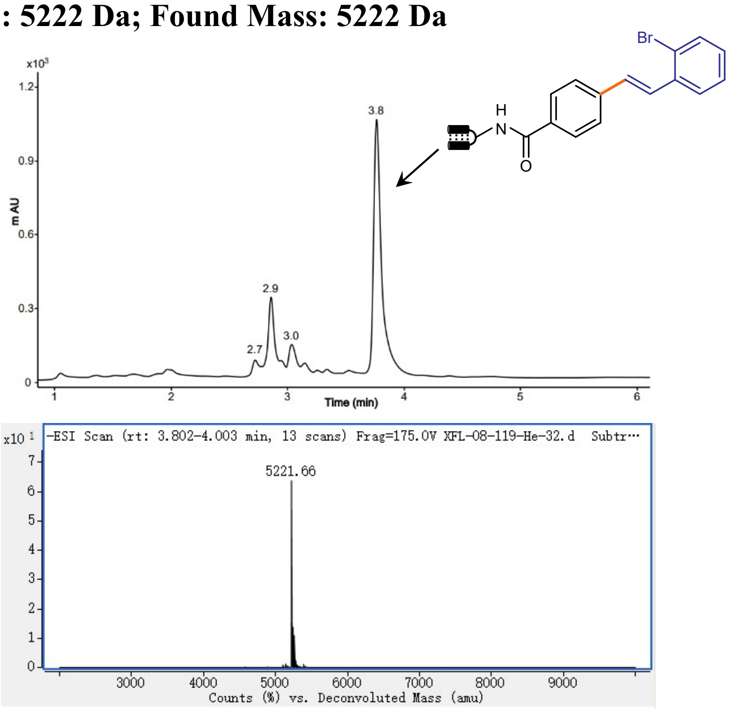

UPLC chromatograph and deconvoluted MS of **5ad.**

Conversion: 83%

Calculated Mass: 5222 Da; Found Mass: 5222 Da

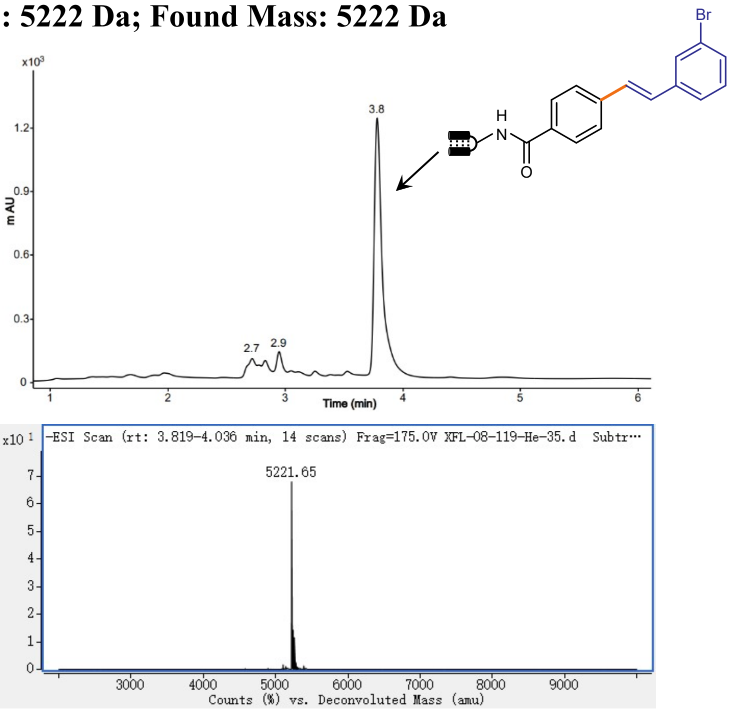

UPLC chromatograph and deconvoluted MS of **5ae.**

Conversion: 75%

Calculated Mass: 5199 Da; Found Mass: 5199 Da

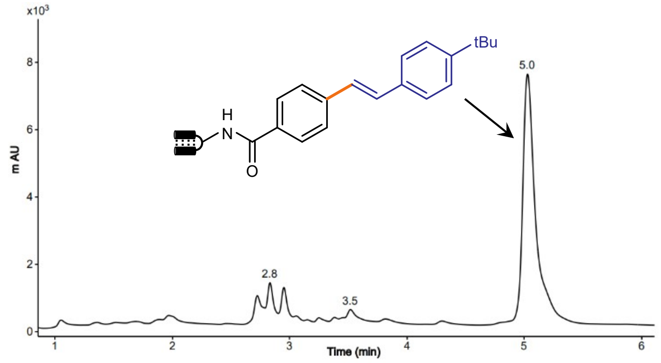

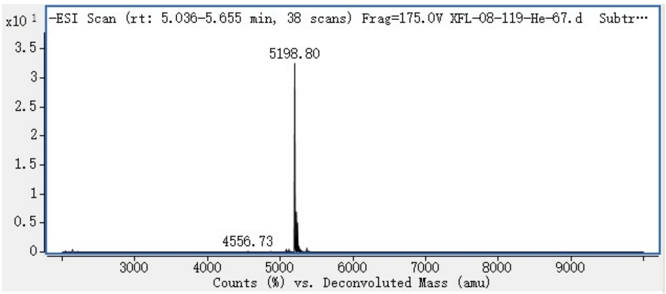

UPLC chromatograph and deconvoluted MS of **5af.**

Conversion: 89%

Calculated Mass: 5110 Da; Found Mass: 5110 Da

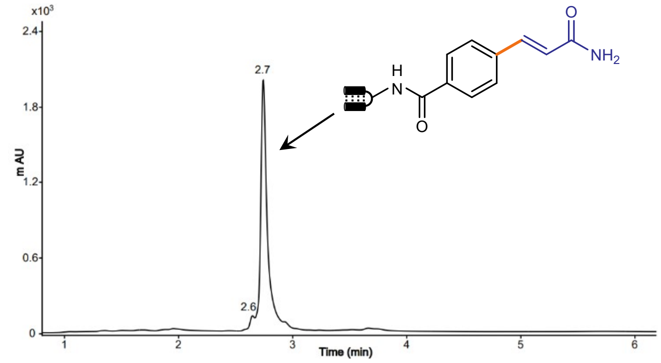

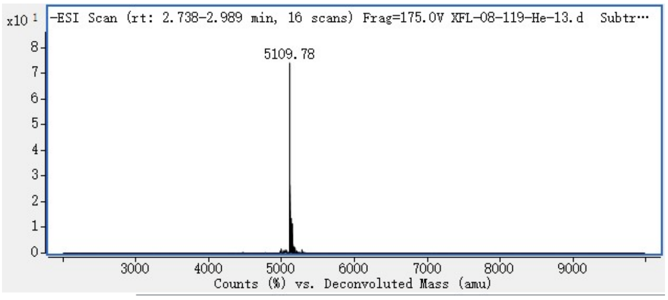

UPLC chromatograph and deconvoluted MS of **5ag.**

Conversion: 90%

Calculated Mass: 5166 Da; Found Mass: 5166 Da

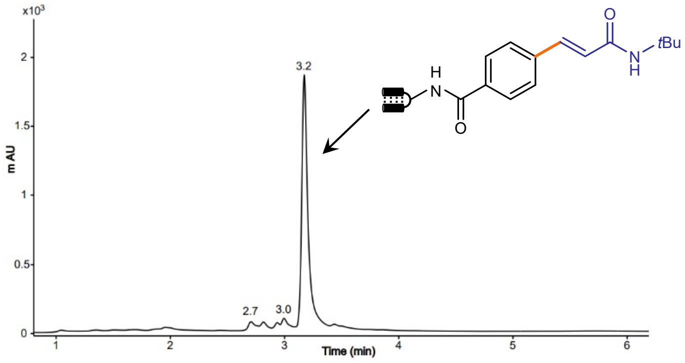

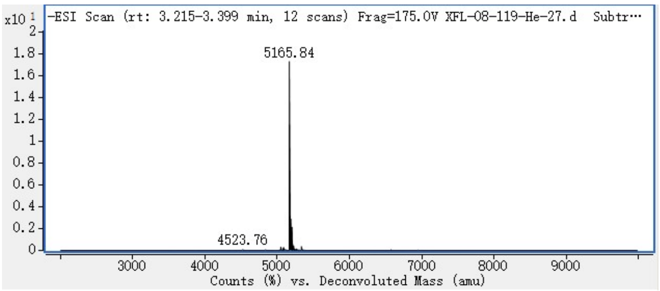

UPLC chromatograph and deconvoluted MS of **5ah.**

Conversion: 85%

Calculated Mass: 5152 Da; Found Mass: 5152 Da

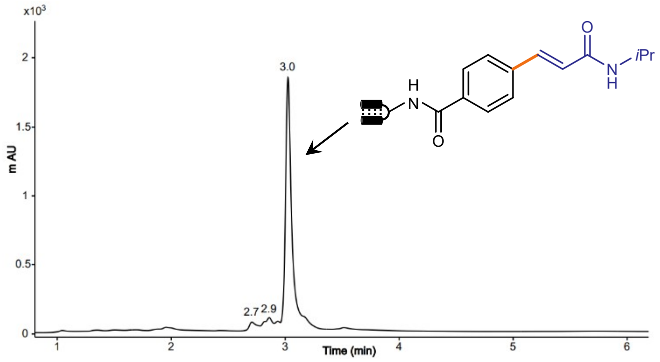

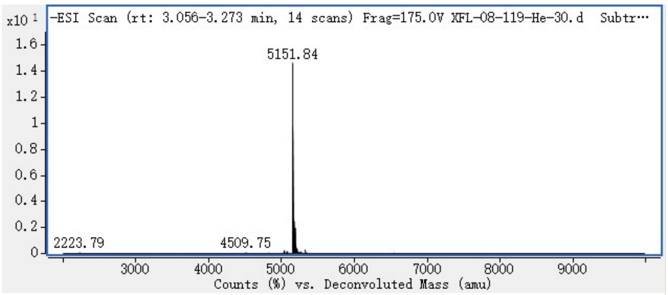

UPLC chromatograph and deconvoluted MS of **5ai.**

Conversion: 87%

Calculated Mass: 5187 Da; Found Mass: 5187 Da

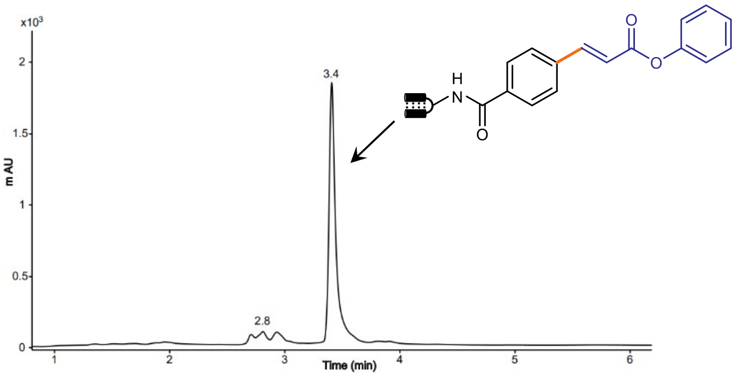

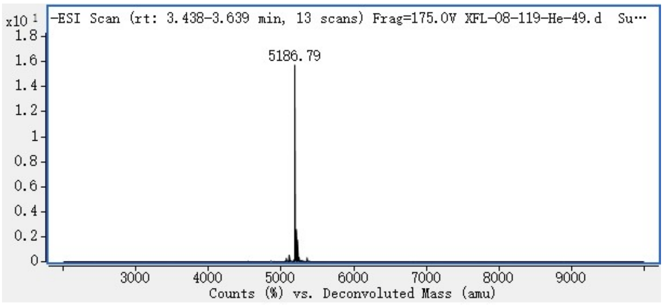

UPLC chromatograph and deconvoluted MS of **5aj**

Conversion: 87%

Calculated Mass: 5186 Da; Found Mass: 5186 Da

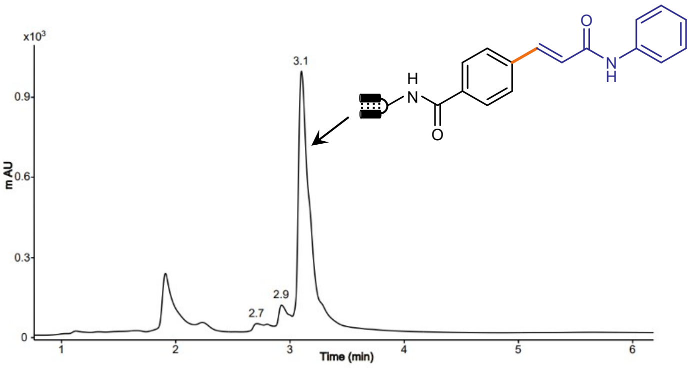

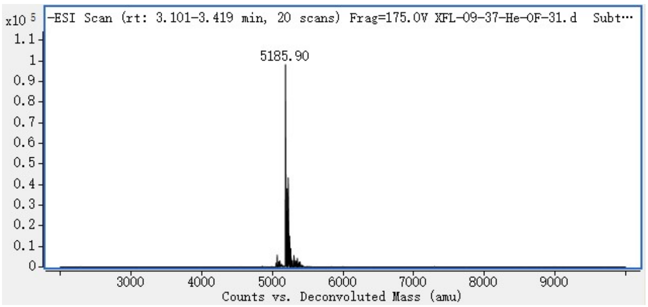

UPLC chromatograph and deconvoluted MS of **5ak.**

Conversion: 80%

Calculated Mass: 5256 Da; Found Mass: 5256 Da

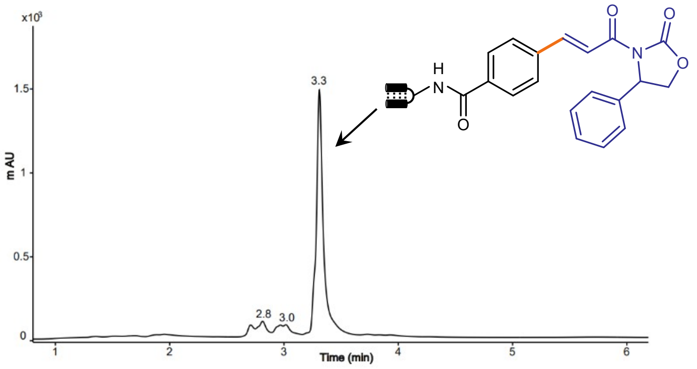

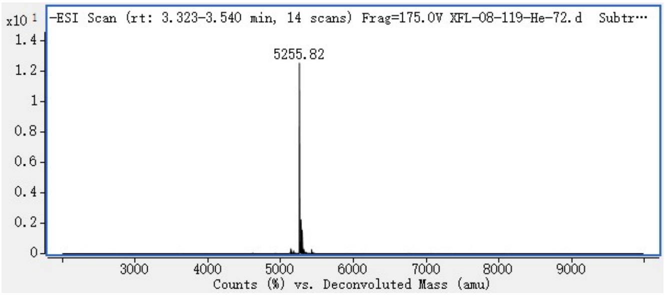

UPLC chromatograph and deconvoluted MS of **5al.**

Conversion: 83%

Calculated Mass: 5246 Da; Found Mass: 5246 Da

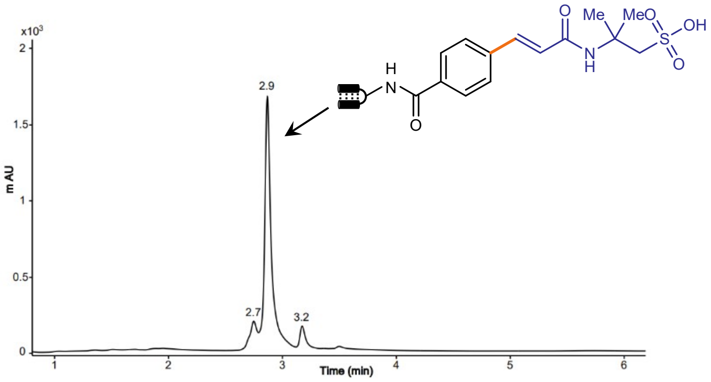

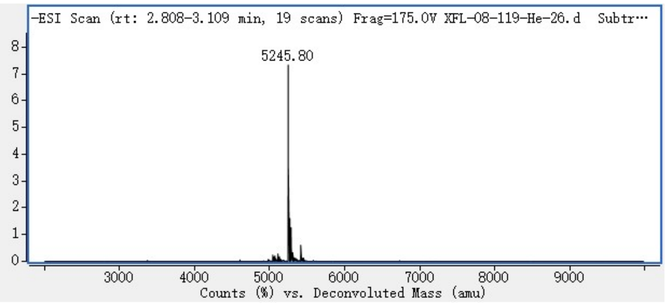

UPLC chromatograph and deconvoluted MS of **5am.**

Conversion: 87%

Calculated Mass: 5138 Da; Found Mass: 5138 Da

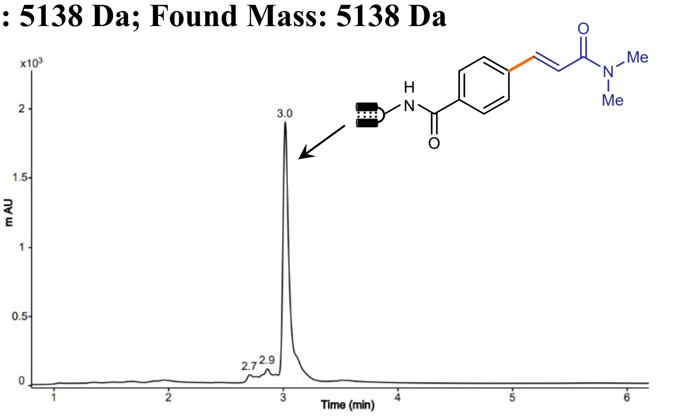

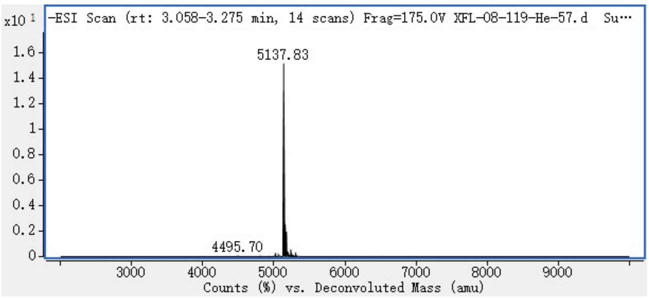

UPLC chromatograph and deconvoluted MS of **5an.**

Conversion: 87%

Calculated Mass: 5166 Da; Found Mass: 5166 Da

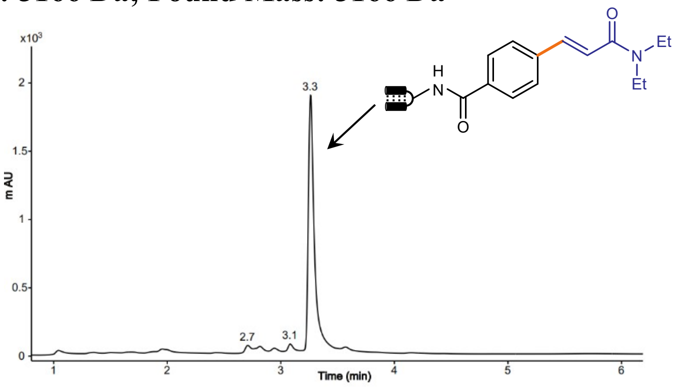

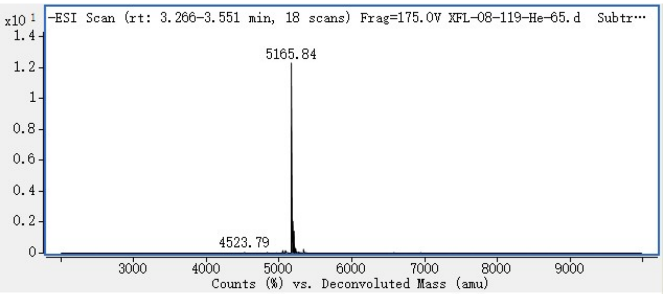

UPLC chromatograph and deconvoluted MS of **5ao.**

Conversion: 86%

Calculated Mass: 5169 Da; Found Mass: 5169 Da

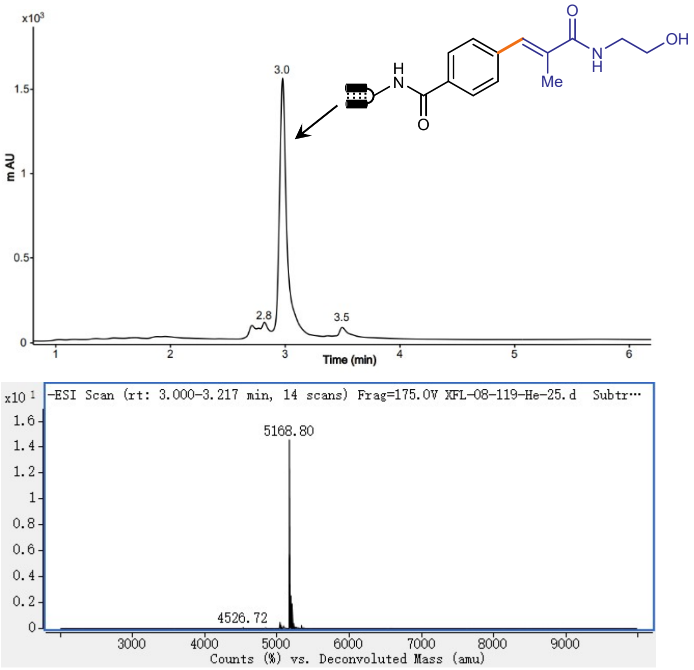

UPLC chromatograph and deconvoluted MS of **5ap.**

Conversion: 68%

Calculated Mass: 5181 Da; Found Mass: 5181 Da

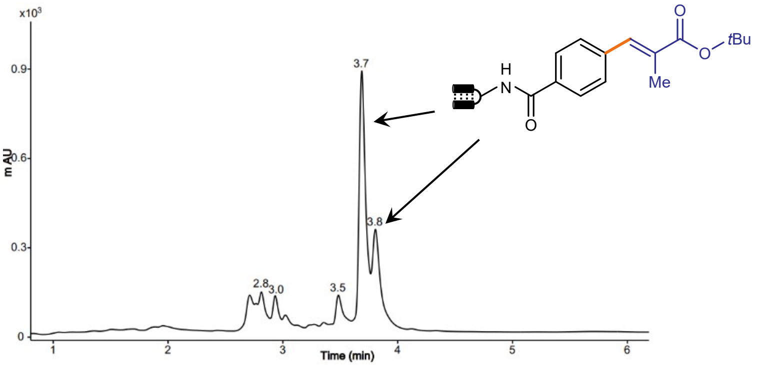

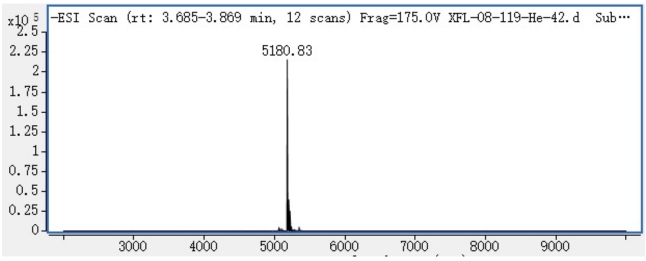

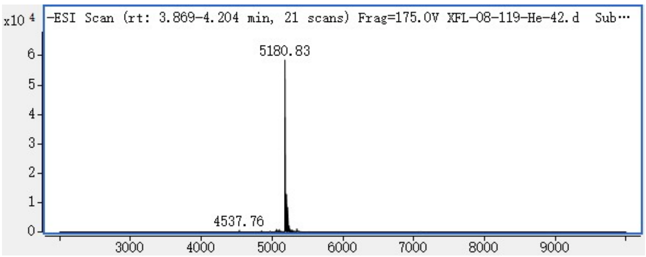

UPLC chromatograph and deconvoluted MS of **5aq.**

Conversion:62%

Calculated Mass: 5109 Da; Found Mass: 5109 Da

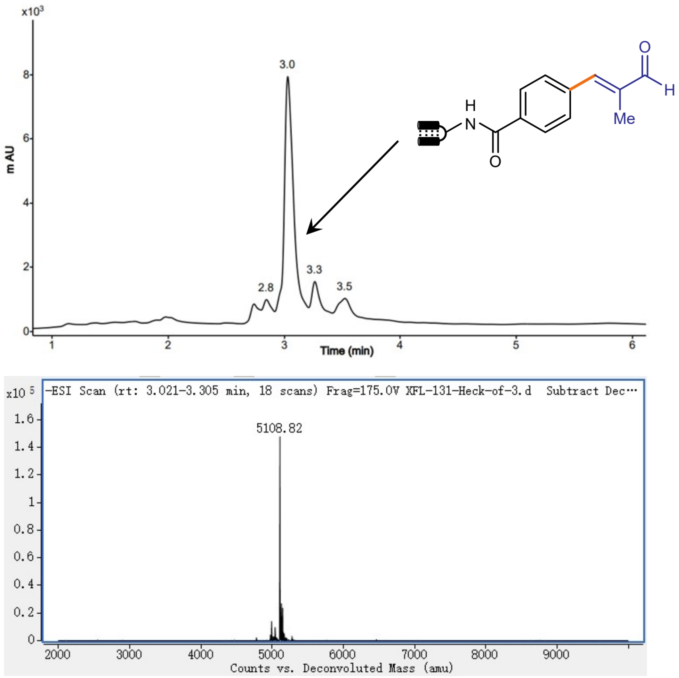

UPLC chromatograph and deconvoluted MS of **5ar.**

Conversion: 82%

Calculated Mass: 5166 Da; Found Mass: 5166 Da

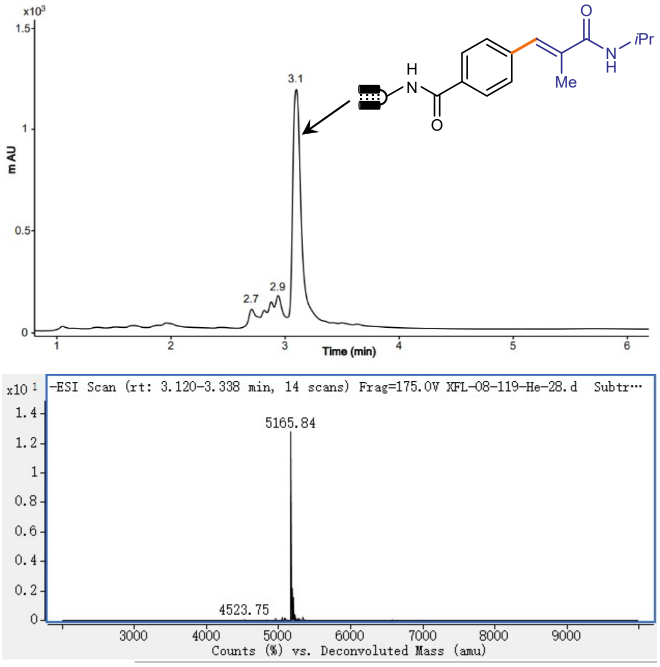

UPLC chromatograph and deconvoluted MS of **5as.**

Conversion: 58%

Calculated Mass: 5153 Da; Found Mass: 5153 Da

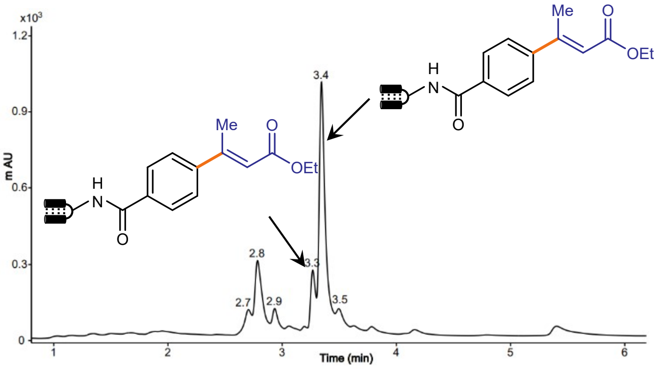

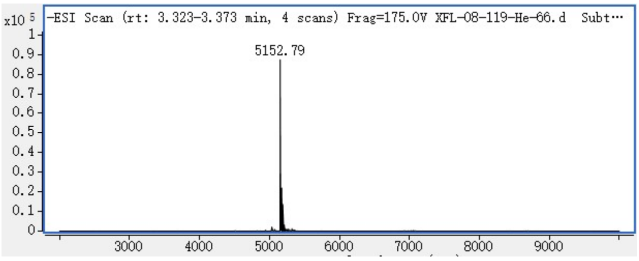

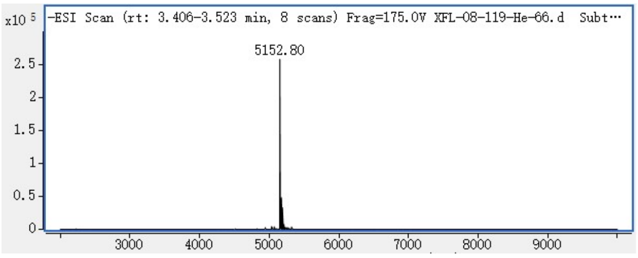

UPLC chromatograph and deconvoluted MS of **5at.**

Conversion: 84%

Calculated Mass: 5147 Da; Found Mass: 5147 Da

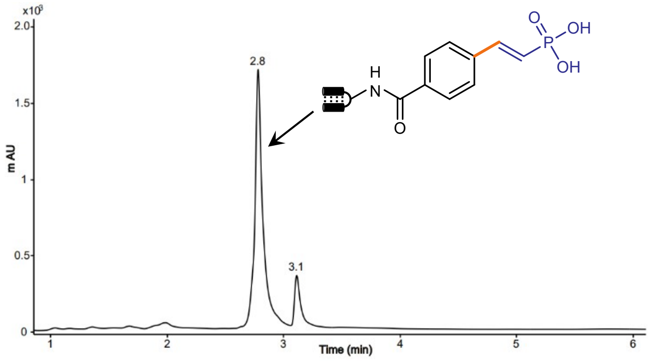

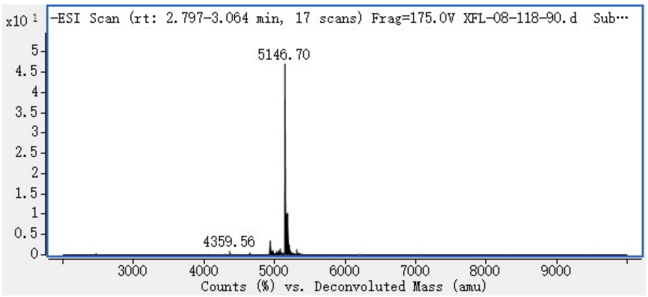

UPLC chromatograph and deconvoluted MS of **5au.**

Conversion: 82%

Calculated Mass: 5245 Da; Found Mass: 5245 Da

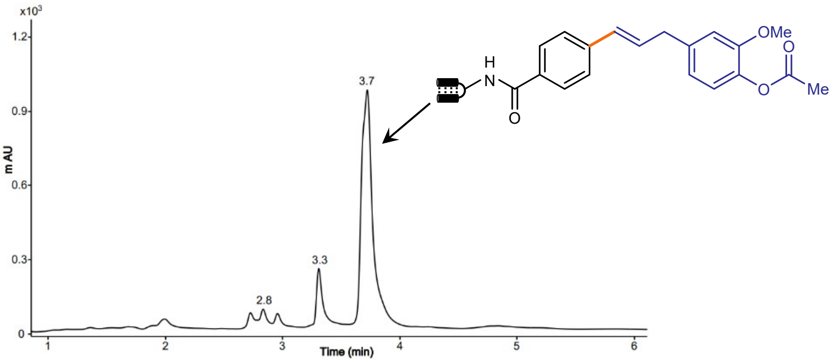

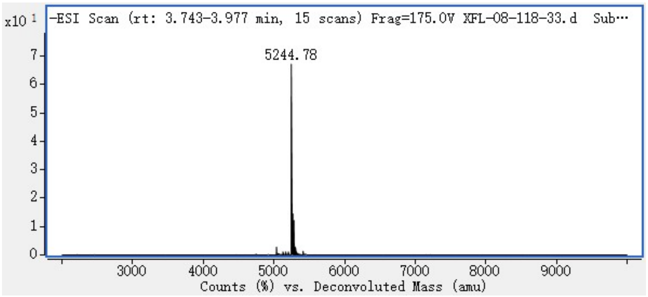

UPLC chromatograph and deconvoluted MS of **5av.**

Conversion: 78%

Calculated Mass: 5157 Da; Found Mass: 5157 Da

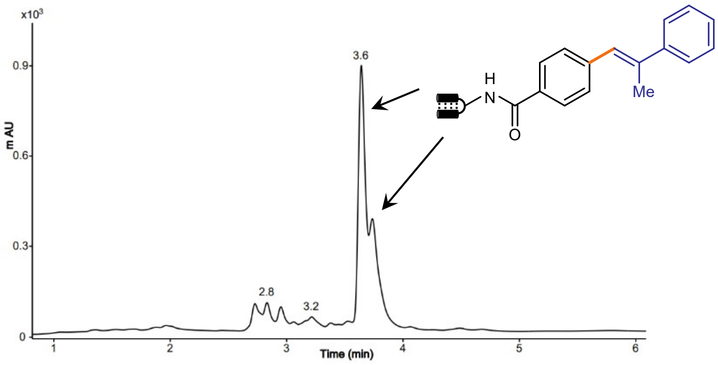

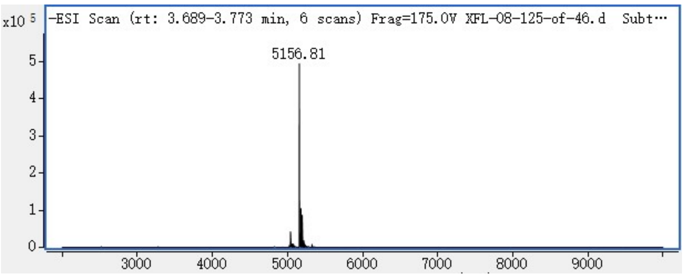

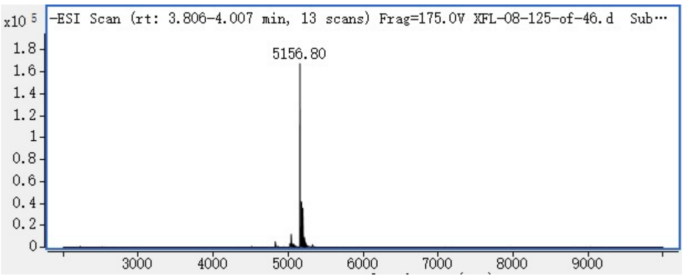

8.6 Substrate scope of DNA-conjugated arylamines for the on-DNA Heck reaction with 4o

UPLC chromatograph and deconvoluted MS of **5o.**

Conversion: 86%

Calculated Mass: 5201 Da; Found Mass: 5201 Da

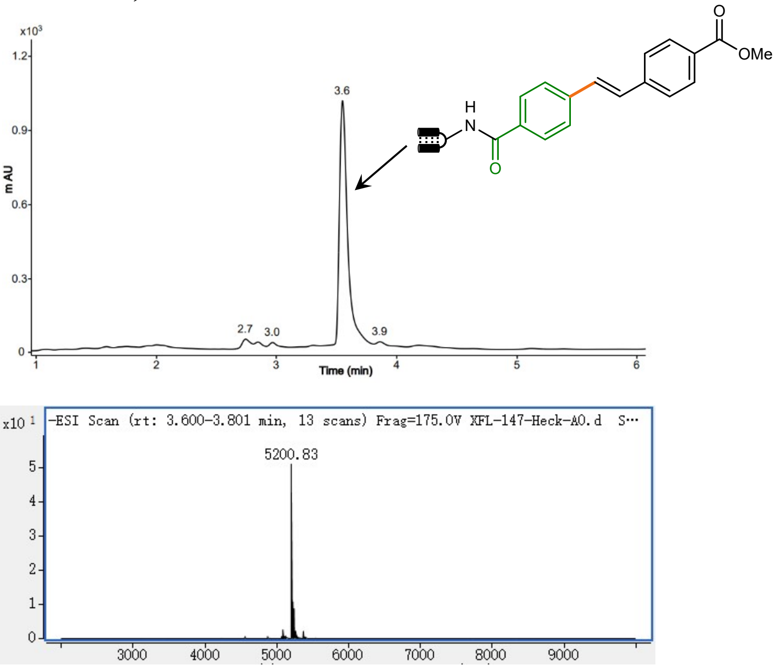

UPLC chromatograph and deconvoluted MS of **5aw.**

Conversion: 90%

Calculated Mass: 5201 Da; Found Mass: 5201 Da

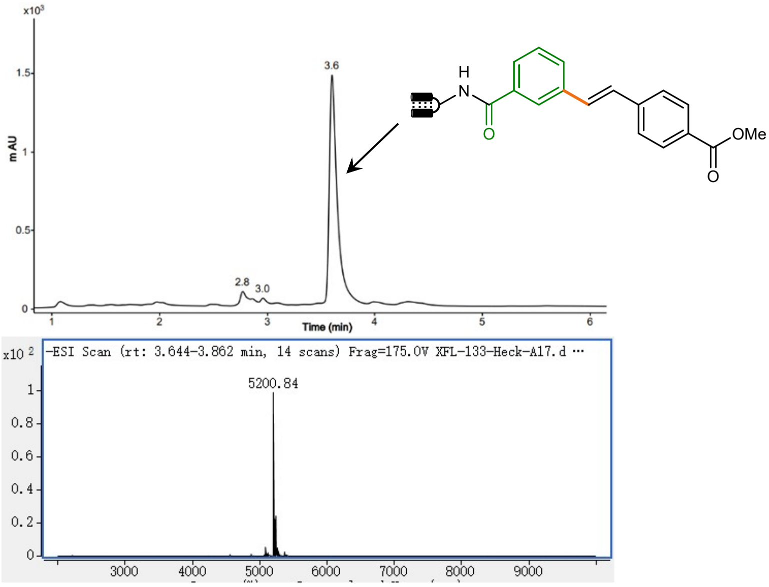

UPLC chromatograph and deconvoluted MS of **5ax.**

Conversion: 77%

Calculated Mass: 5215 Da; Found Mass: 5215 Da

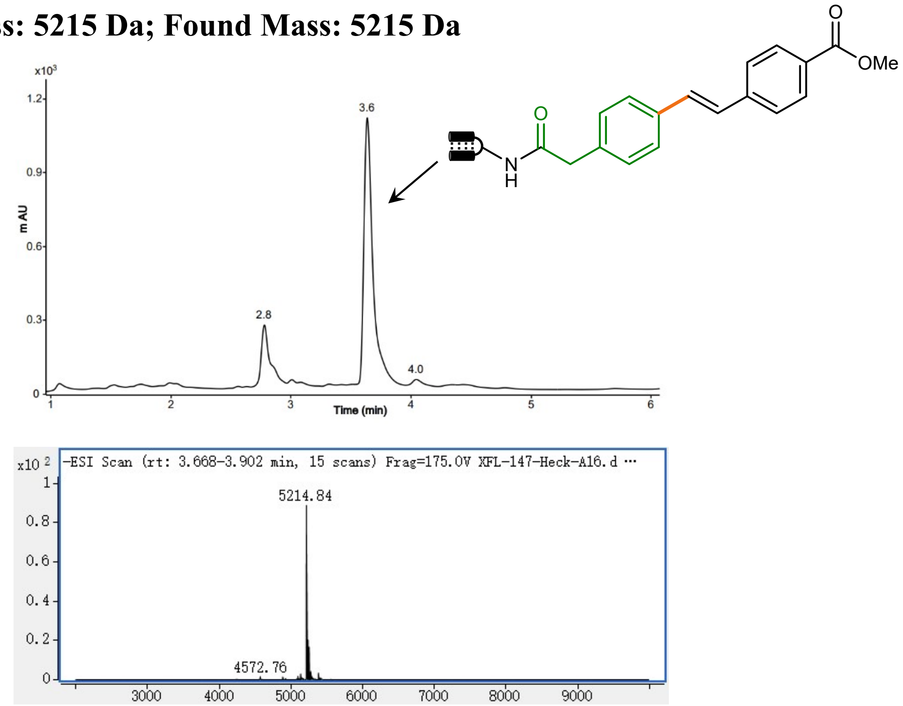

UPLC chromatograph and deconvoluted MS of **5ay.**

Conversion: 70%

Calculated Mass: 5231 Da; Found Mass: 5231 Da

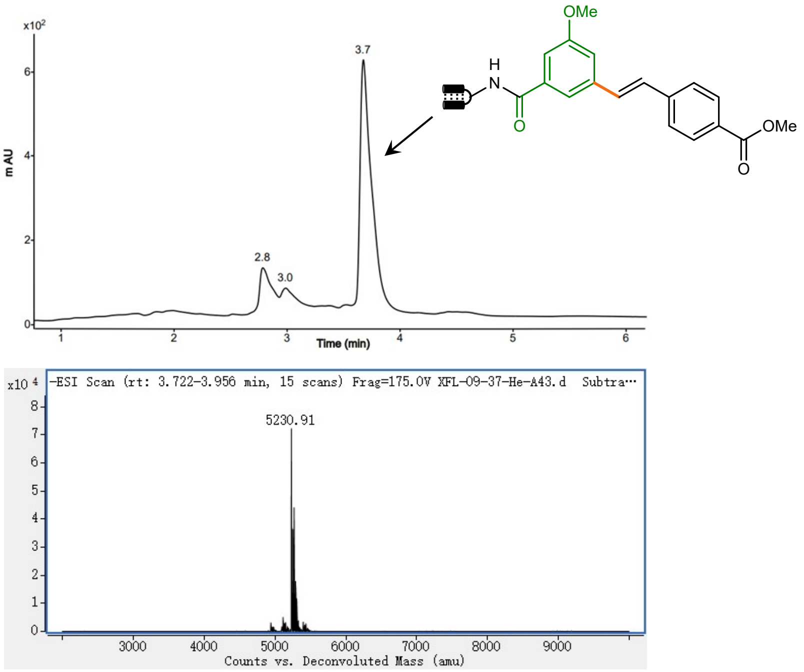

UPLC chromatograph and deconvoluted MS of **5az.**

Conversion: 86%

Calculated Mass: 5269 Da; Found Mass: 5269 Da

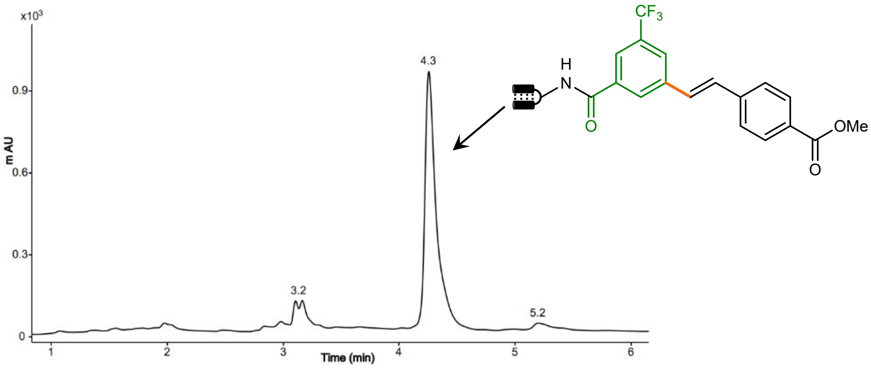

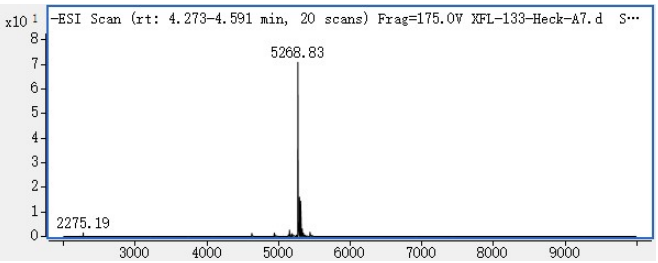

UPLC chromatograph and deconvoluted MS of **5ba.**

Conversion: 92%

Calculated Mass: 5231 Da; Found Mass: 5231 Da

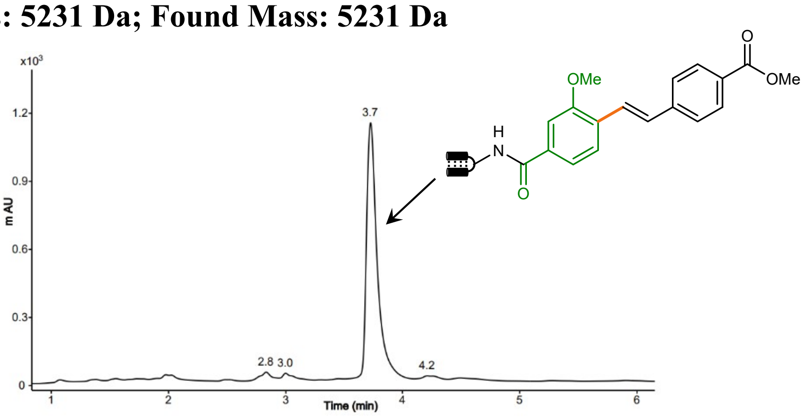

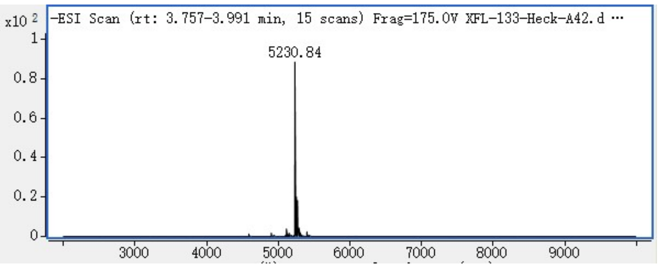

UPLC chromatograph and deconvoluted MS of **5bb.**

Conversion: 76%

Calculated Mass: 5259 Da; Found Mass: 5259 Da

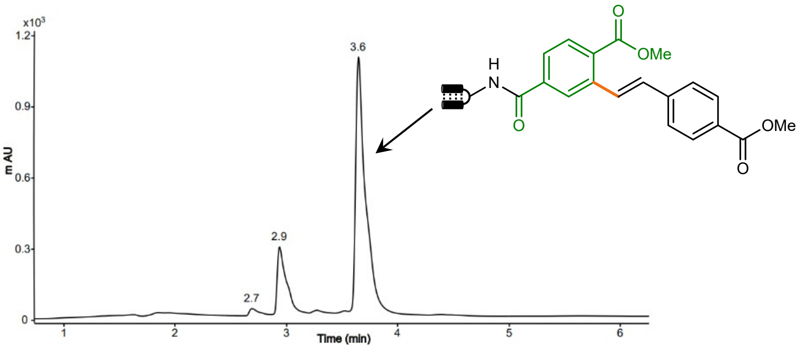

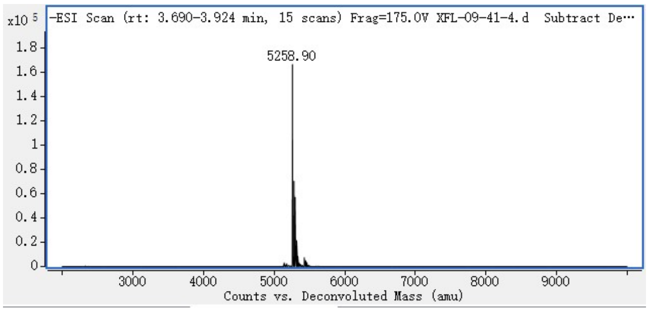

UPLC chromatograph and deconvoluted MS of **5bc.**

Conversion: 92%

Calculated Mass: 5277 Da; Found Mass: 5277 Da

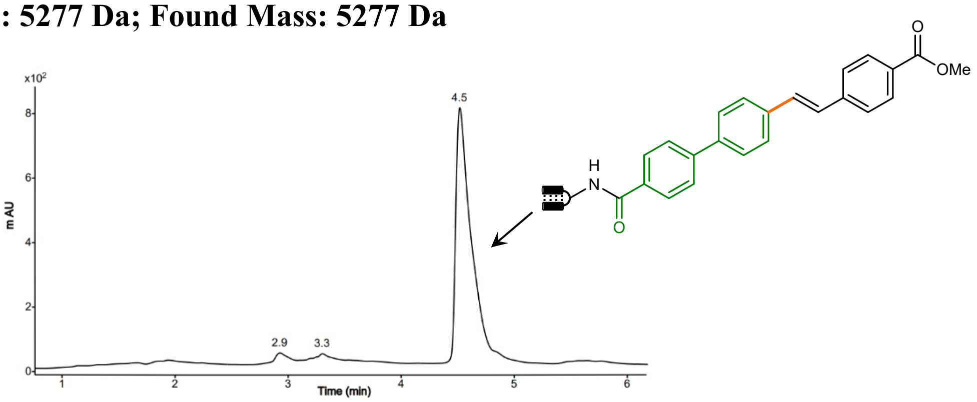

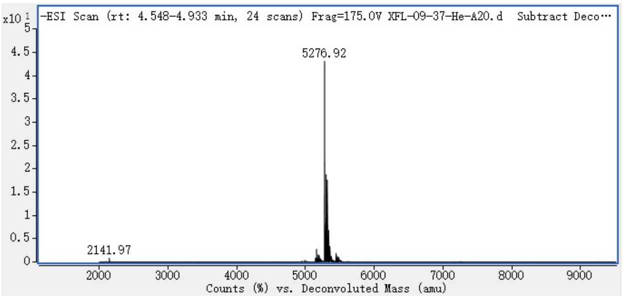

UPLC chromatograph and deconvoluted MS of **5bd.**

Conversion: 81%

Calculated Mass: 5251 Da; Found Mass: 5251 Da

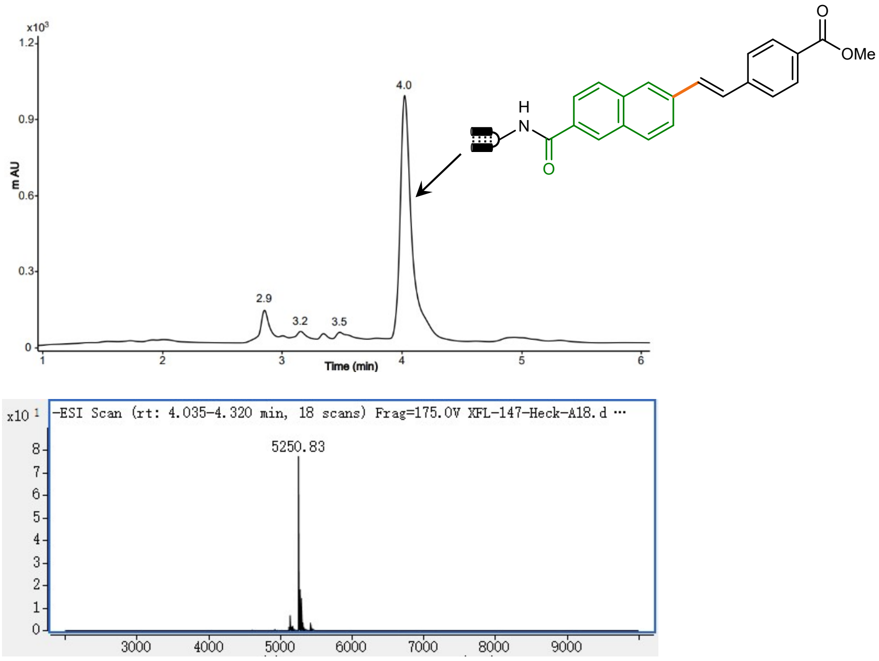

UPLC chromatograph and deconvoluted MS of **5be.**

Conversion: 83%

Calculated Mass: 5237 Da; Found Mass: 5237 Da

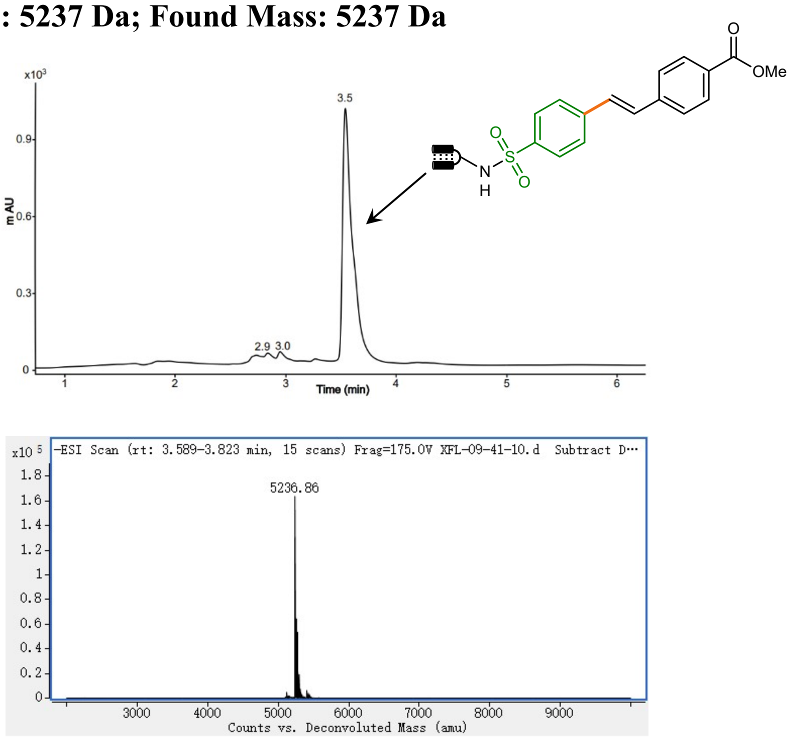

UPLC chromatograph and deconvoluted MS of **5bf.**

Conversion: 86%

Calculated Mass: 5219 Da; Found Mass: 5219 Da

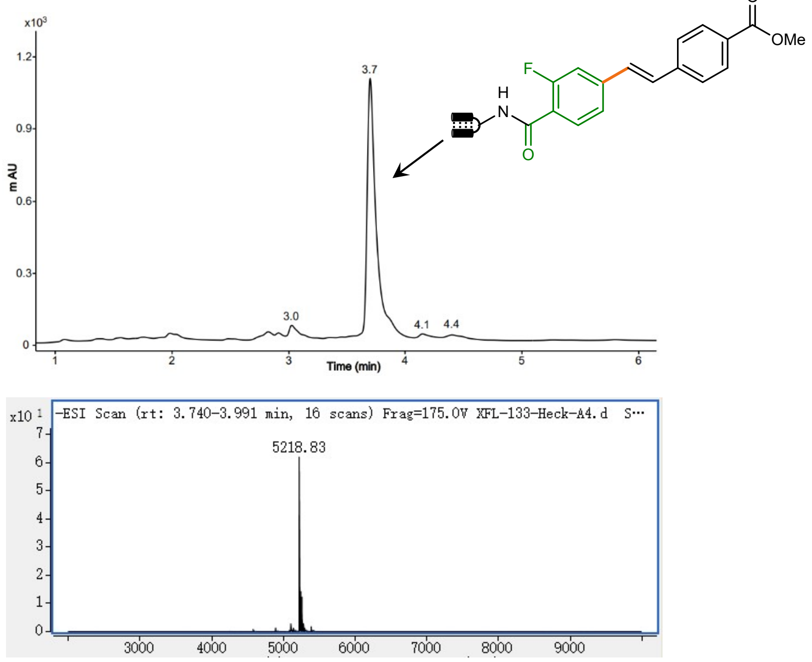

UPLC chromatograph and deconvoluted MS of 5**bg.**

Conversion: 89%

Calculated Mass: 5233 Da; Found Mass: 5233 Da

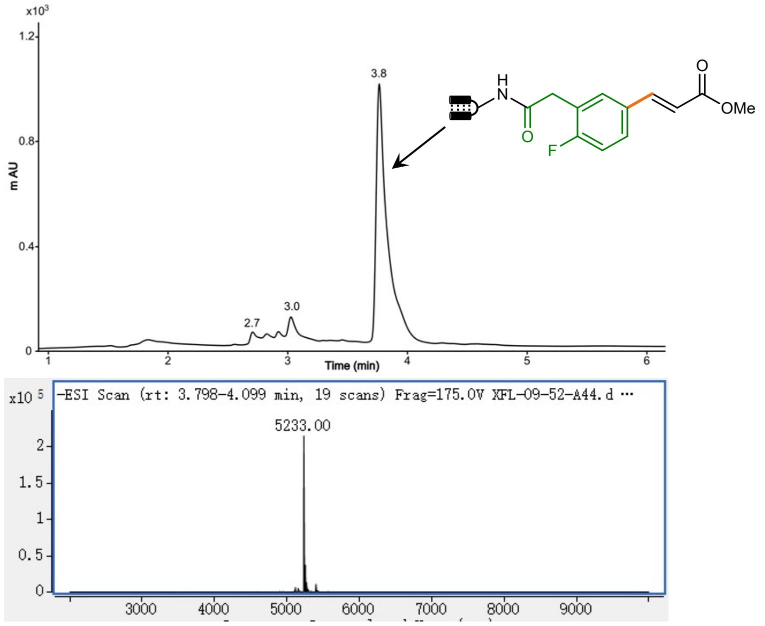

UPLC chromatograph and deconvoluted MS of **5bh.**

Conversion: 96%

Calculated Mass: 5229 Da; Found Mass: 5229 Da

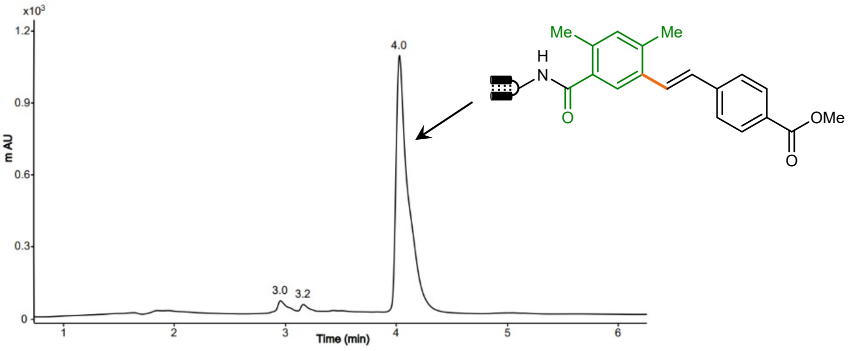

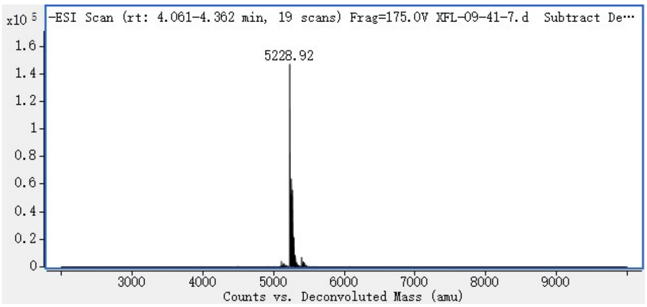

UPLC chromatograph and deconvoluted MS of **5bi.**

Conversion: 84%

Calculated Mass: 5233 Da; Found Mass: 5233 Da

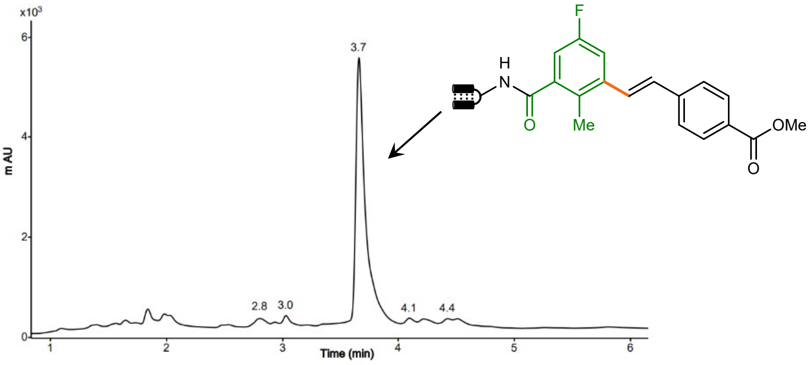

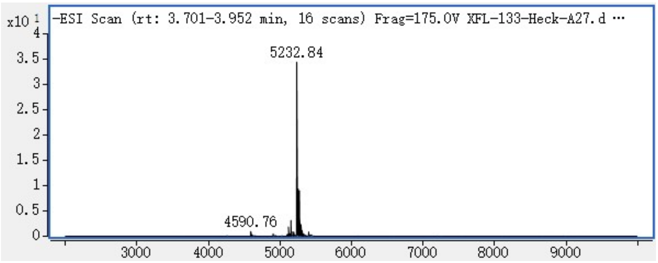

UPLC chromatograph and deconvoluted MS of **5bj.**

Conversion: 82%

Calculated Mass: 5231 Da; Found Mass: 5231 Da

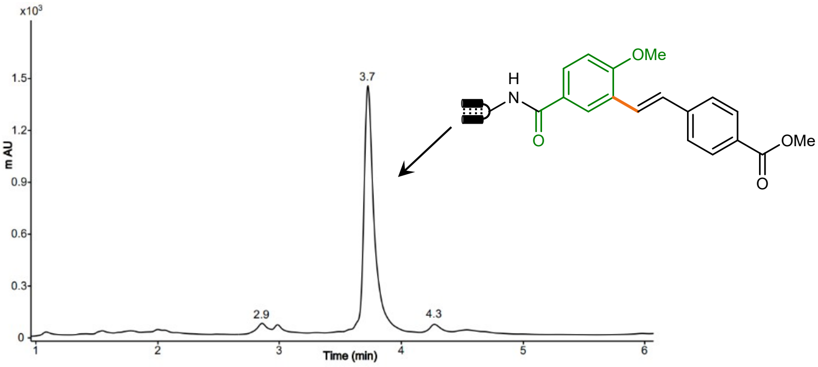

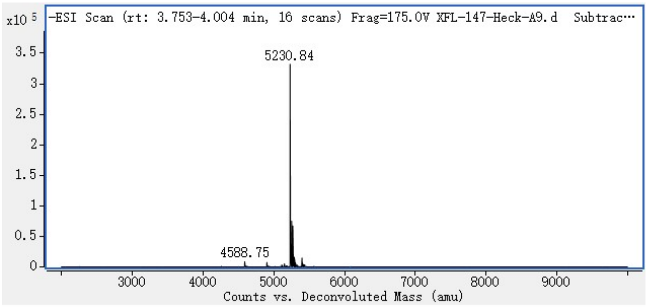

UPLC chromatograph and deconvoluted MS of **5bk.**

Conversion: 64%

Calculated Mass: 5219 Da; Found Mass: 5219 Da

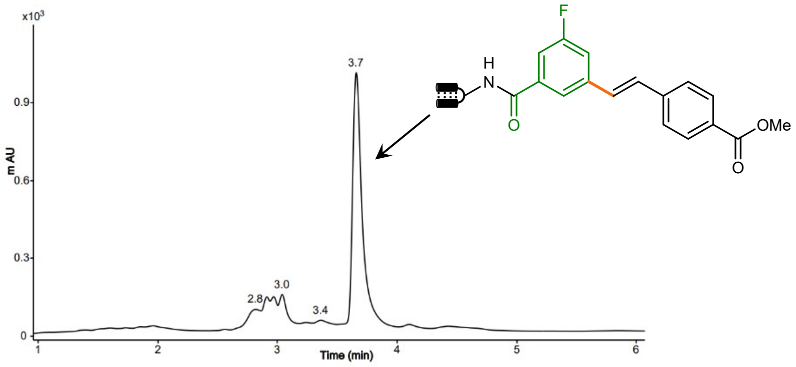

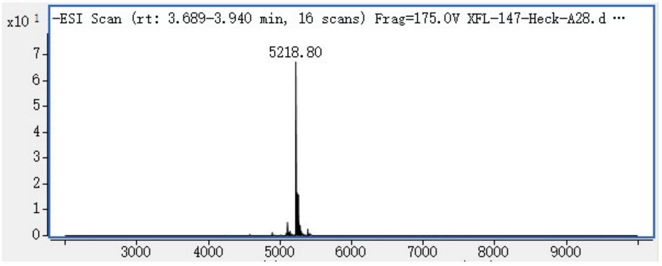

UPLC chromatograph and deconvoluted MS of **5bl.**

Conversion: 84%

Calculated Mass: 5233 Da; Found Mass: 5233 Da

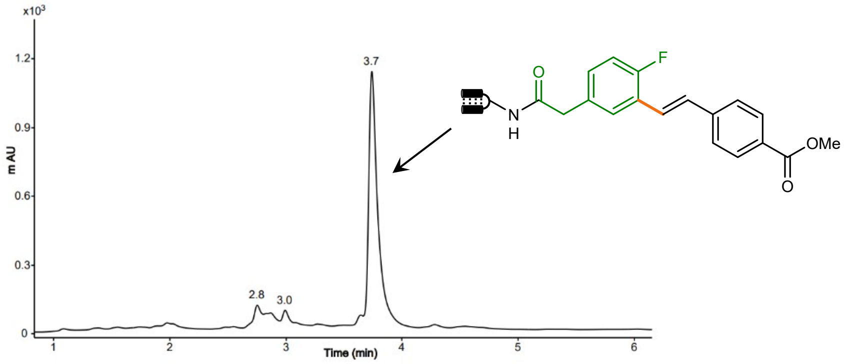

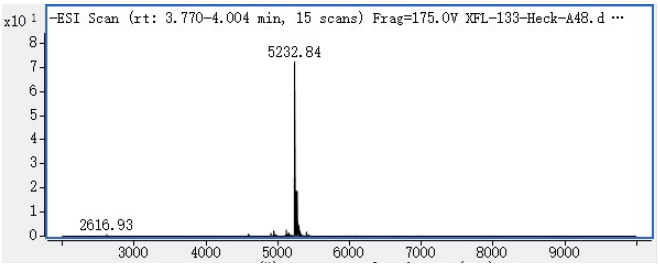

UPLC chromatograph and deconvoluted MS of **5bm.**

Conversion: 90%

Calculated Mass: 5231 Da; Found Mass: 5231 Da

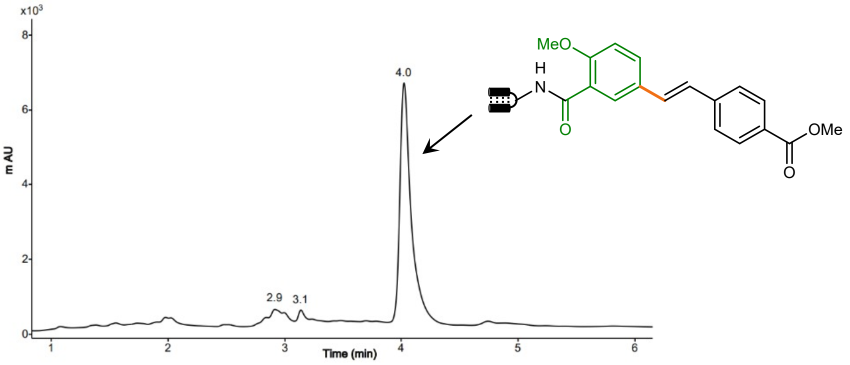

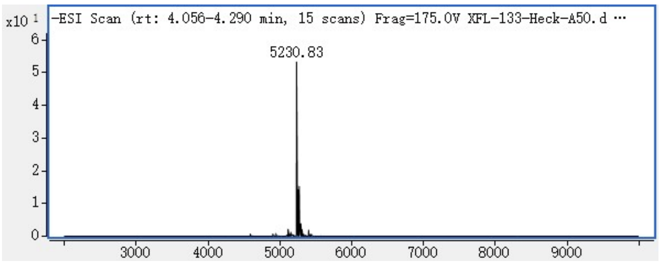

UPLC chromatograph and deconvoluted MS of **5bn.**

Conversion: 50%

Calculated Mass: 5219 Da; Found Mass: 5219 Da

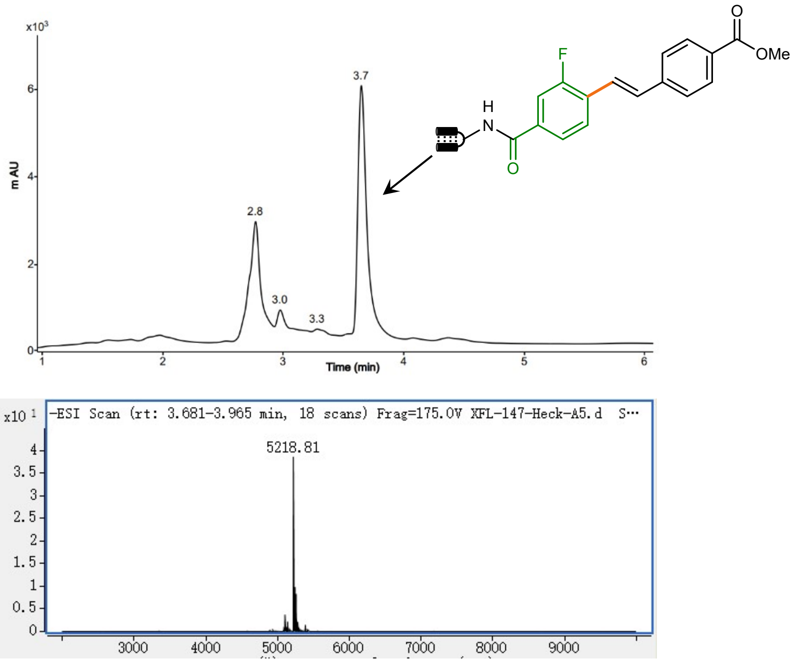

UPLC chromatograph and deconvoluted MS of **5bo.**

Conversion: 87%

Calculated Mass: 5215 Da; Found Mass: 5215 Da

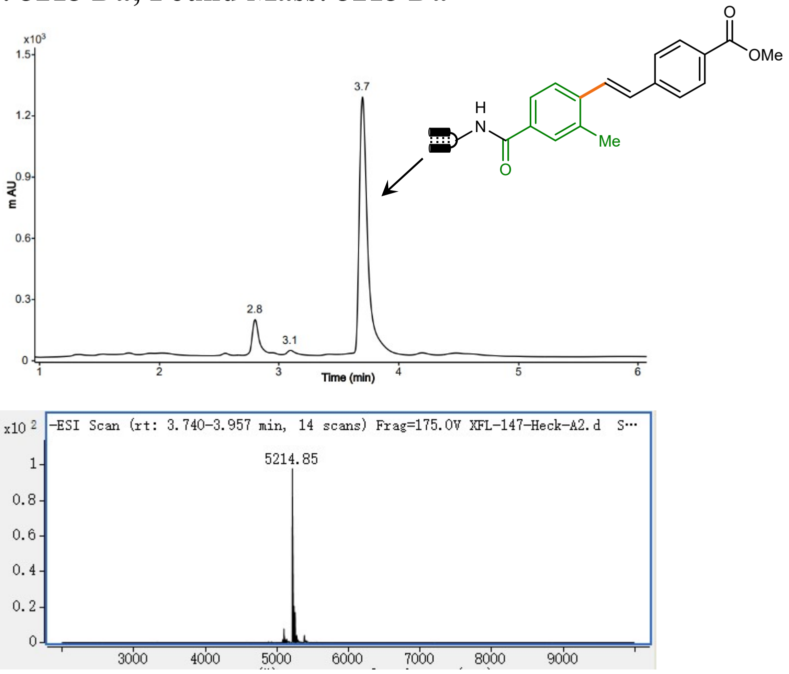

UPLC chromatograph and deconvoluted MS of **5bp.**

Conversion: 84%

Calculated Mass: 5215 Da; Found Mass: 5215 Da

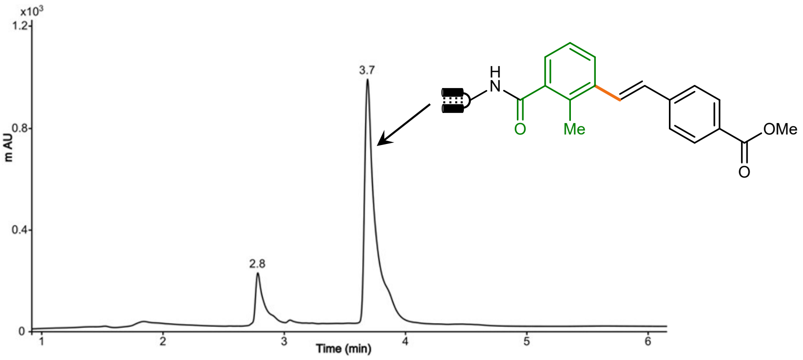

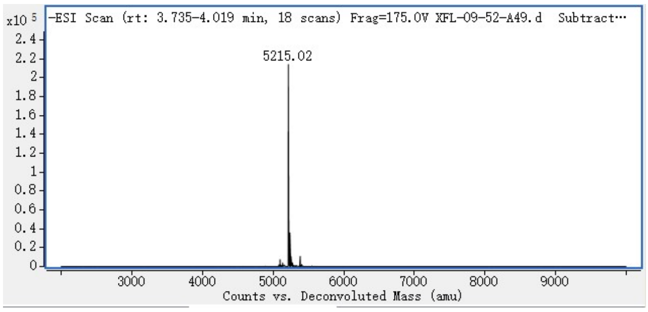

UPLC chromatograph and deconvoluted MS of **5bq.**

Conversion: 90%

Calculated Mass: 5280 Da; Found Mass: 5280 Da

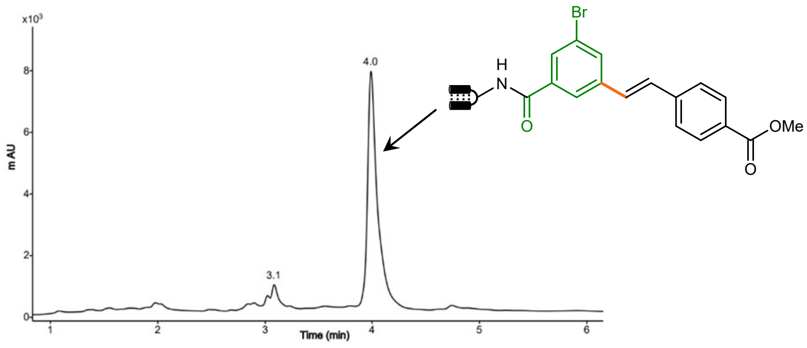

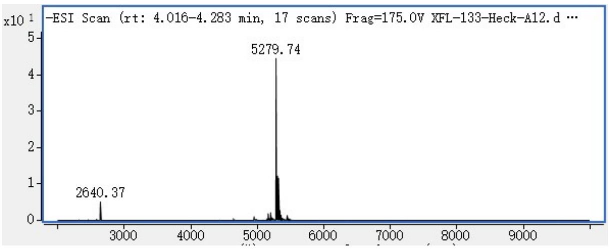

UPLC chromatograph and deconvoluted MS of **5br.**

Conversion: 82%

Calculated Mass: 5240 Da; Found Mass: 5240 Da

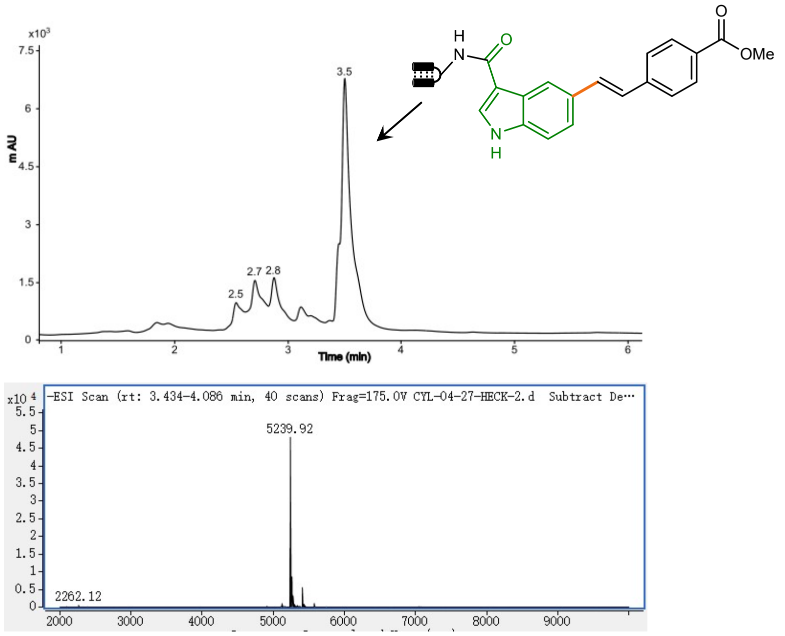

UPLC chromatograph and deconvoluted MS of **5bs.**

Conversion: 70%

Calculated Mass: 5257 Da; Found Mass: 5257 Da

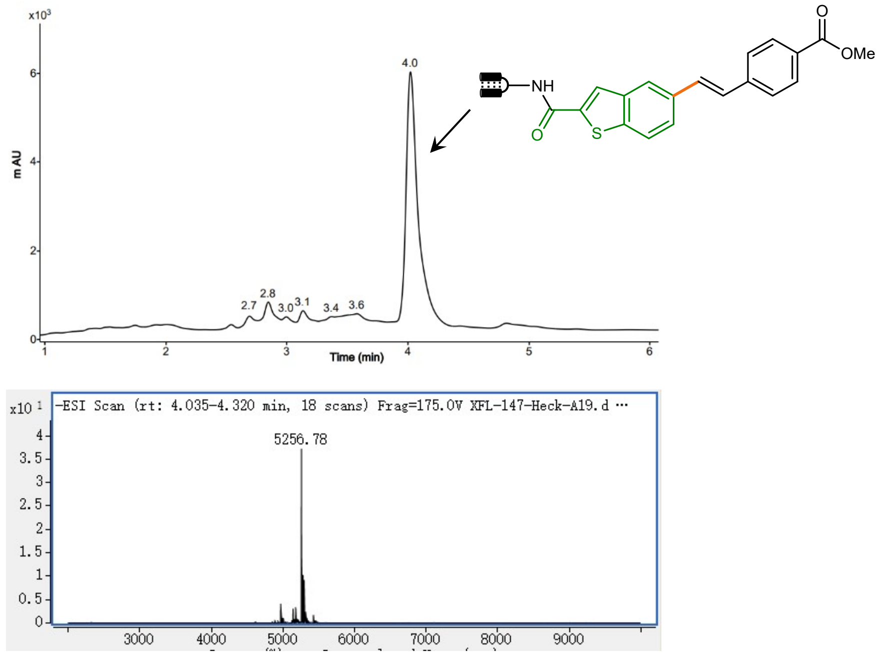

UPLC chromatograph and deconvoluted MS of **5bt.**

Conversion: 73%

Calculated Mass: 5245 Da; Found Mass: 5245 Da

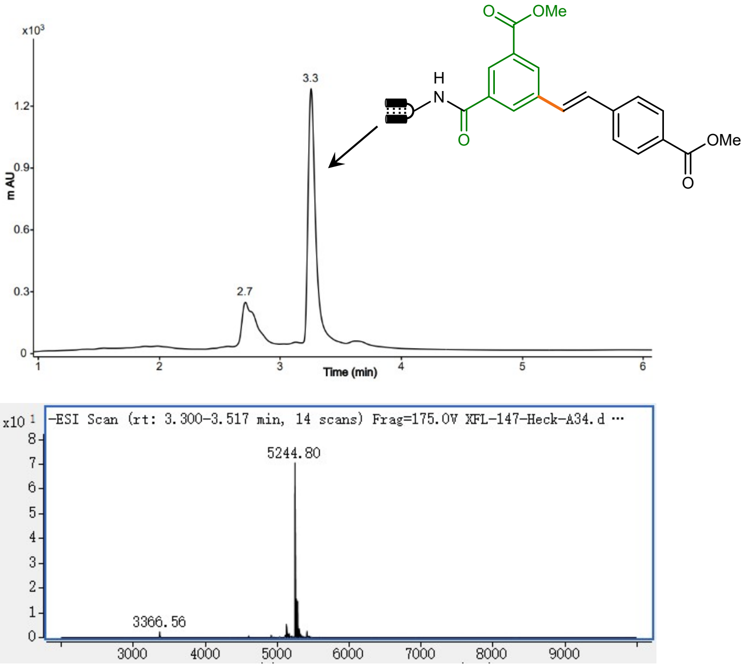

UPLC chromatograph and deconvoluted MS of **5bu.**

Conversion: 81%

Calculated Mass: 5236 Da; Found Mass: 5235 Da

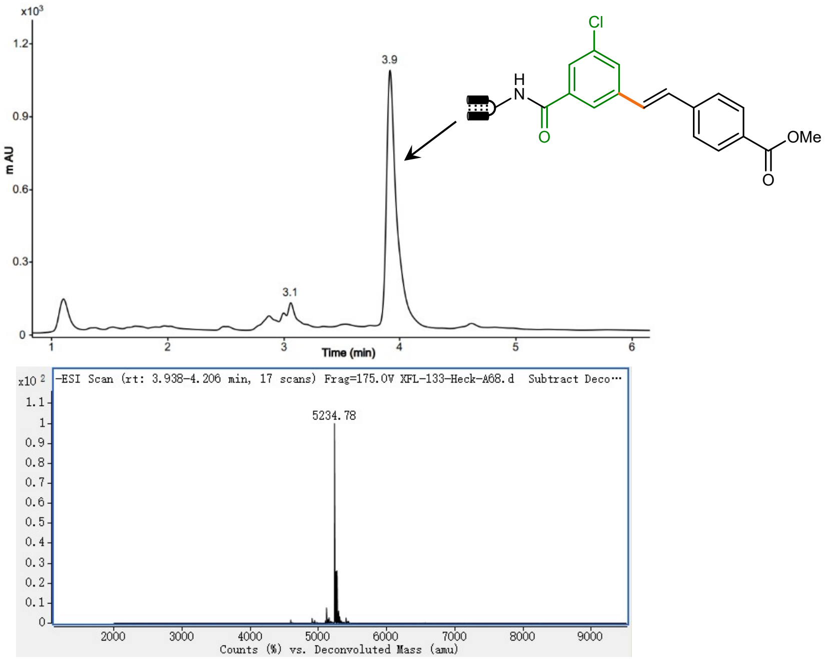

UPLC chromatograph and deconvoluted MS of **5bv.**

Conversion: 84%

Calculated Mass: 5236 Da; Found Mass: 5236 Da

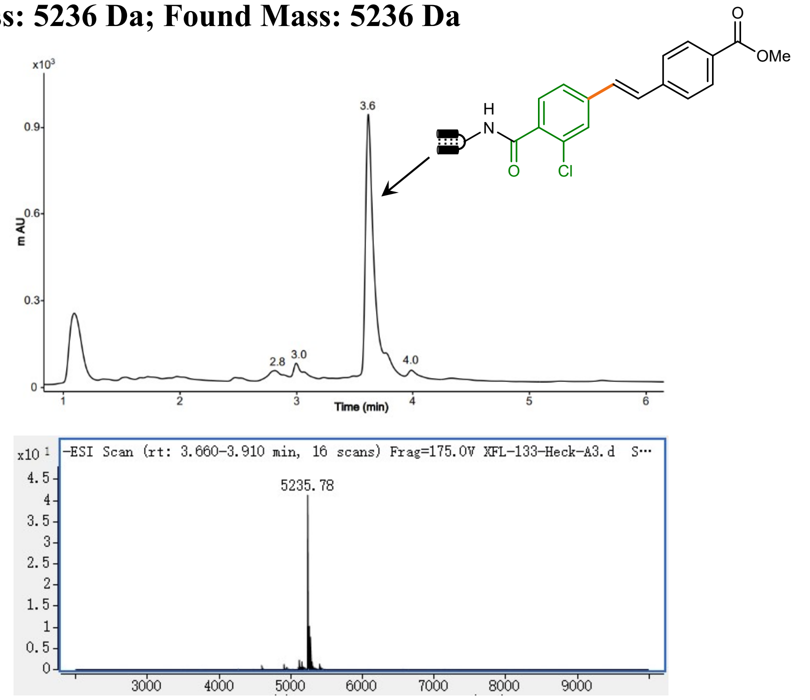

UPLC chromatograph and deconvoluted MS of **5bw.**

Conversion: 88%

Calculated Mass: 5236 Da; Found Mass: 5236 Da

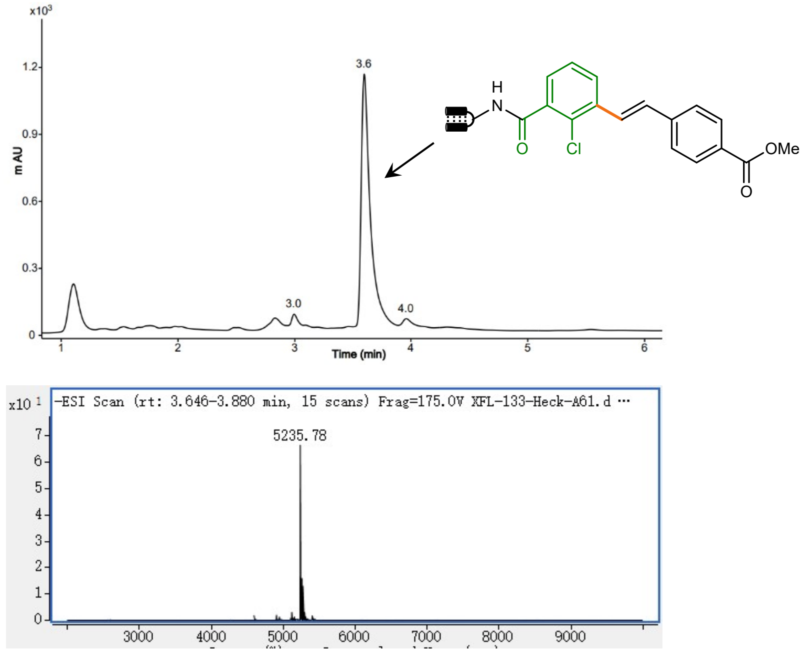

UPLC chromatograph and deconvoluted MS of **5bx.**

Conversion: 82%

Calculated Mass: 5236 Da; Found Mass: 5236 Da

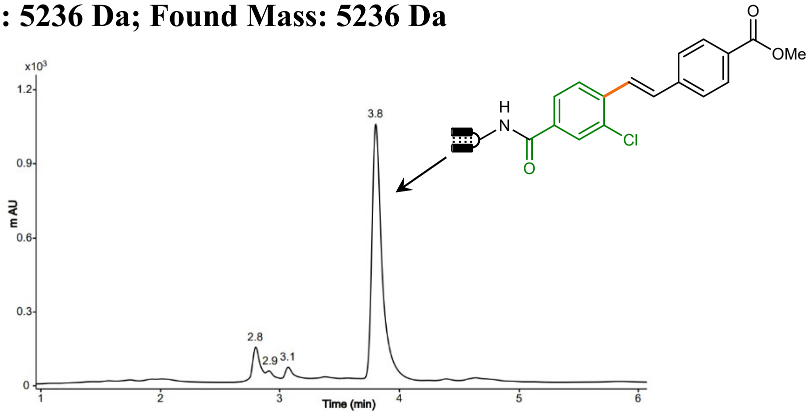

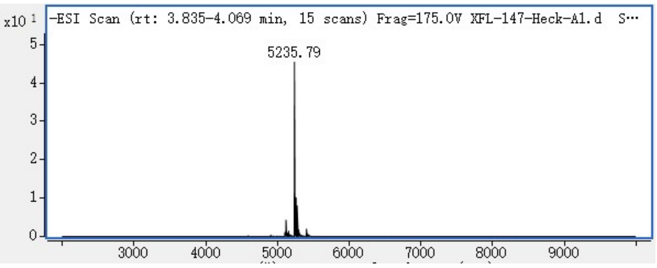

UPLC chromatograph and deconvoluted MS of **5by.**

Conversion: 62%

Calculated Mass: 5215 Da; Found Mass: 5215 Da

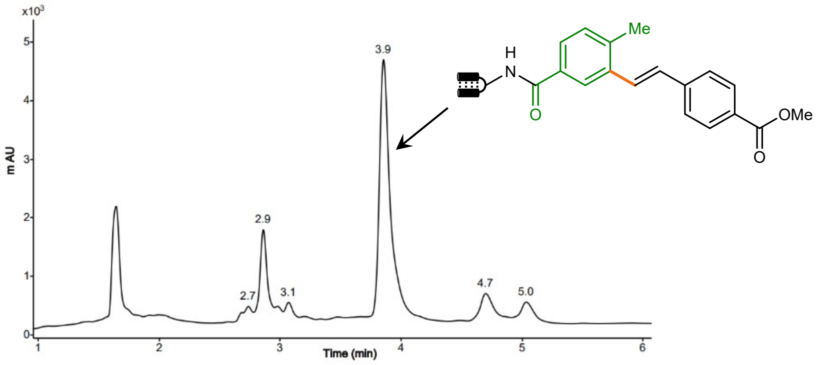

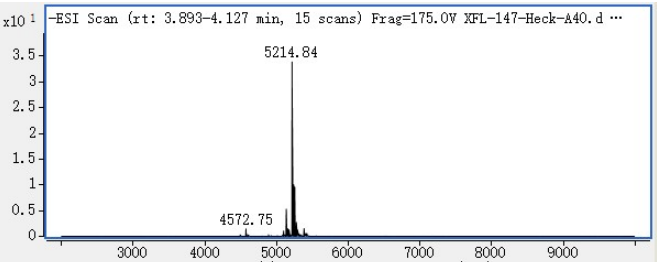

UPLC chromatograph and deconvoluted MS of **5bz.**

Conversion: 80%

Calculated Mass: 5269 Da; Found Mass: 5269 Da

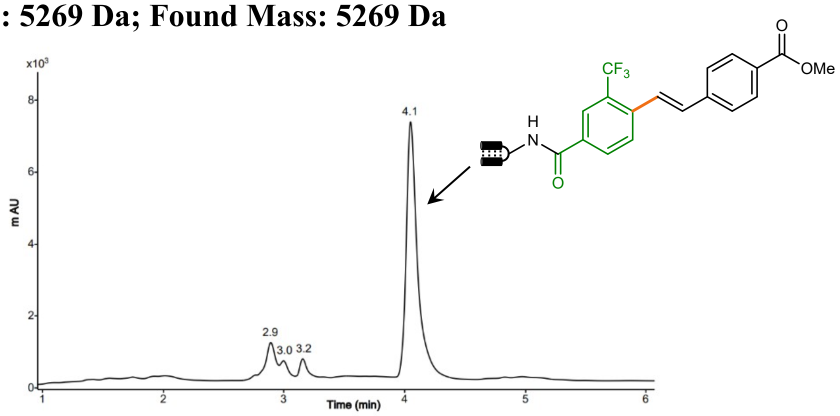

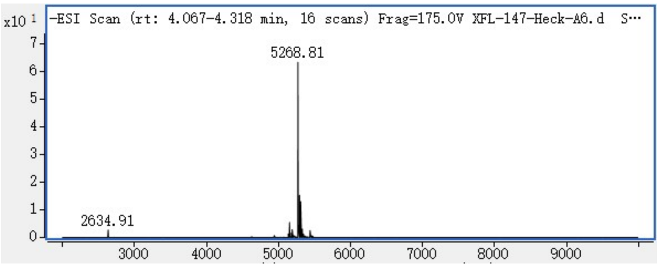

UPLC chromatograph and deconvoluted MS of **5ca.**

Conversion: 81%

Calculated Mass: 5215 Da; Found Mass: 5215 Da

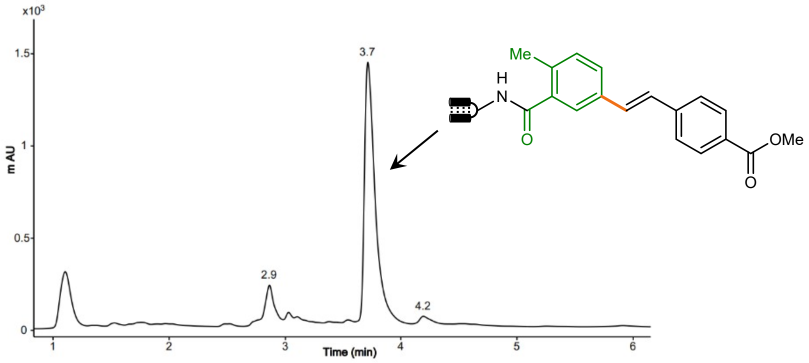

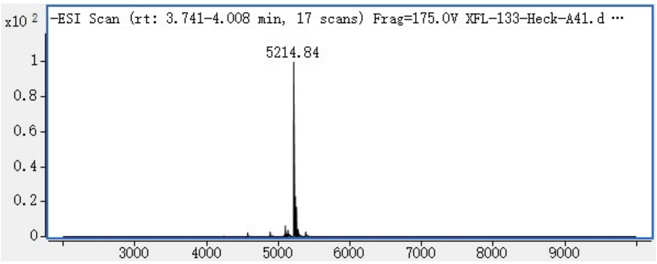

UPLC chromatograph and deconvoluted MS of **5cb.**

Conversion: 43%

Calculated Mass: 5229 Da; Found Mass: 5229 Da

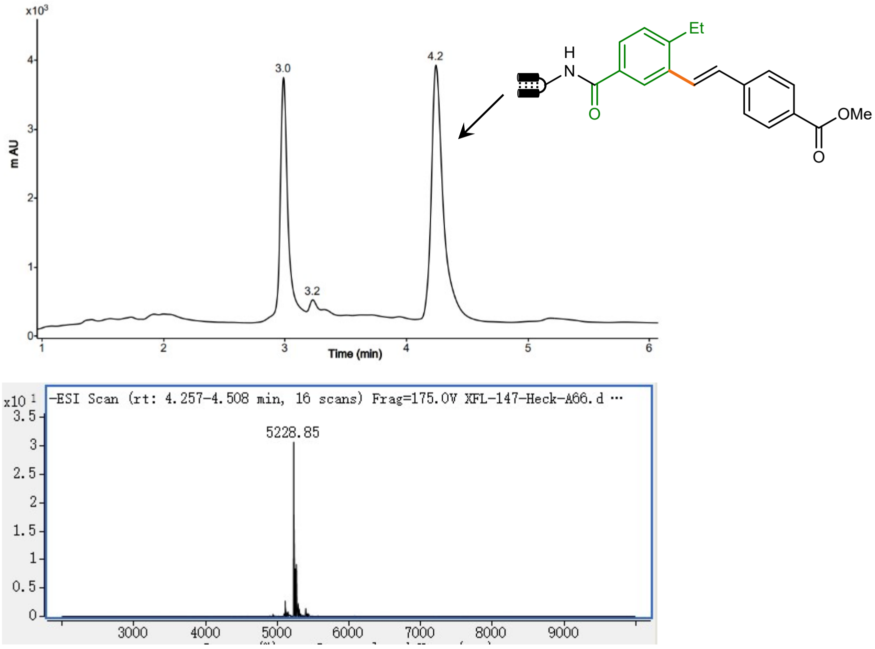

UPLC chromatograph and deconvoluted MS of **5cc.**

Conversion: 67%

Calculated Mass: 5246 Da; Found Mass: 5246 Da

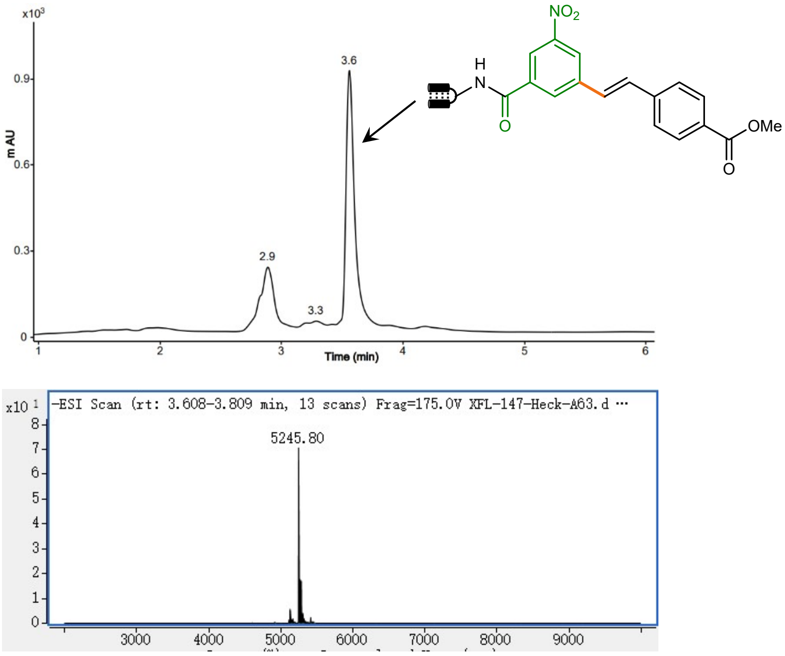

8.7 Substrate scope of DNA-conjugated arylamines for the on-DNA Heck reaction with 4r

UPLC chromatograph and deconvoluted MS of **5r.**

Conversion: 86%

Calculated Mass: 5125 Da; Found Mass: 5125 Da

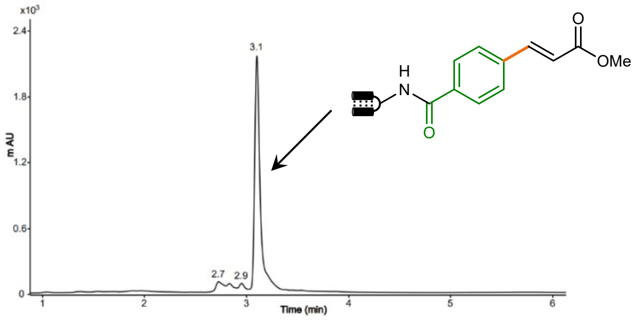

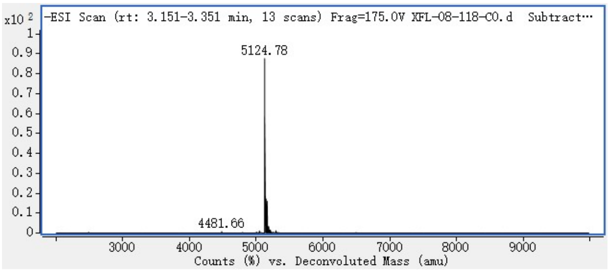

UPLC chromatograph and deconvoluted MS of **5cd.**

Conversion: 84%

Calculated Mass: 5125 Da; Found Mass: 5125 Da

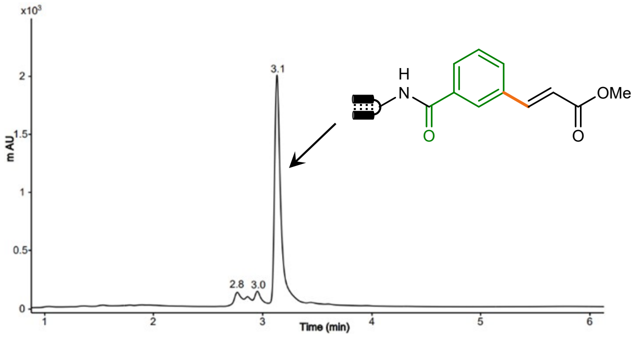

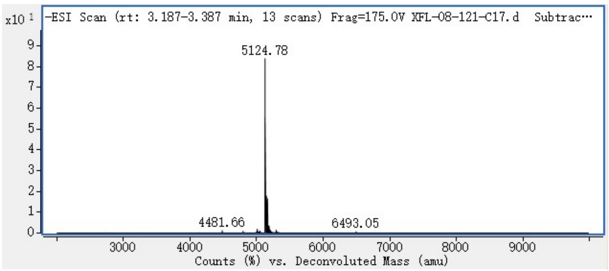

UPLC chromatograph and deconvoluted MS of **5ce.**

Conversion: 92%

Calculated Mass: 5139 Da; Found Mass: 5139 Da

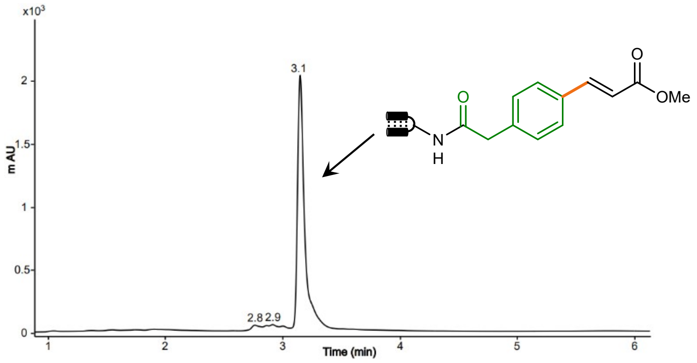

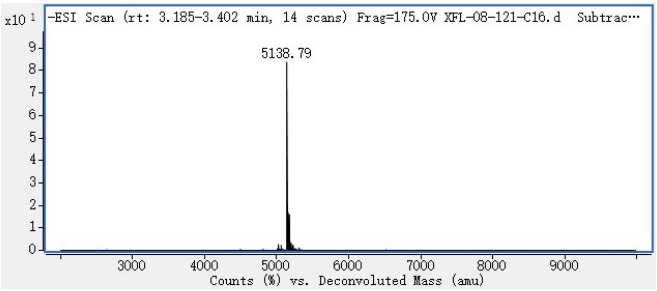

UPLC chromatograph and deconvoluted MS of **5cf.**

Conversion: 80%

Calculated Mass: 5155 Da; Found Mass: 5155 Da

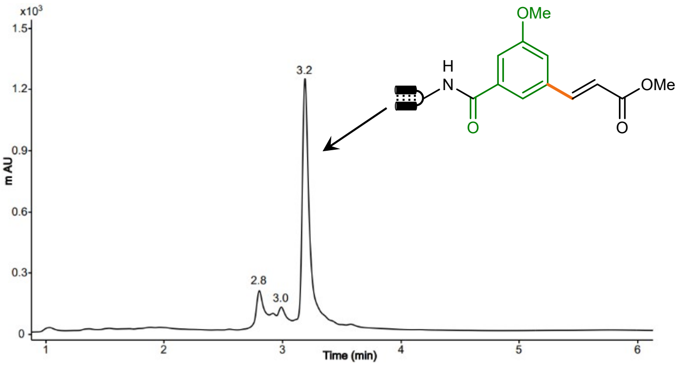

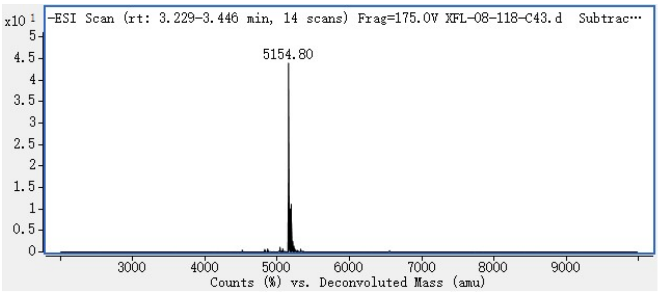

UPLC chromatograph and deconvoluted MS of **5cg.**

Conversion: 72%

Calculated Mass: 5193 Da; Found Mass: 5193 Da

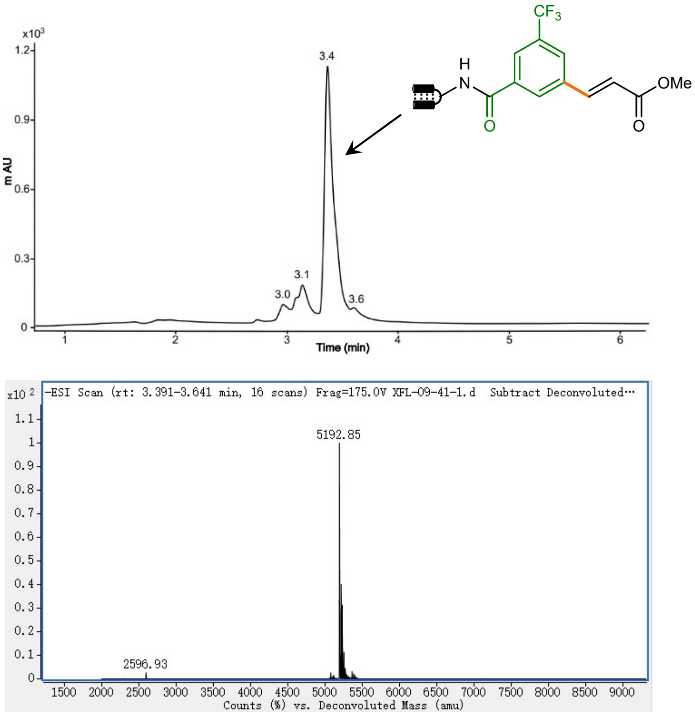

UPLC chromatograph and deconvoluted MS of **5ch.**

Conversion: 92%

Calculated Mass: 5155 Da; Found Mass: 5155 Da

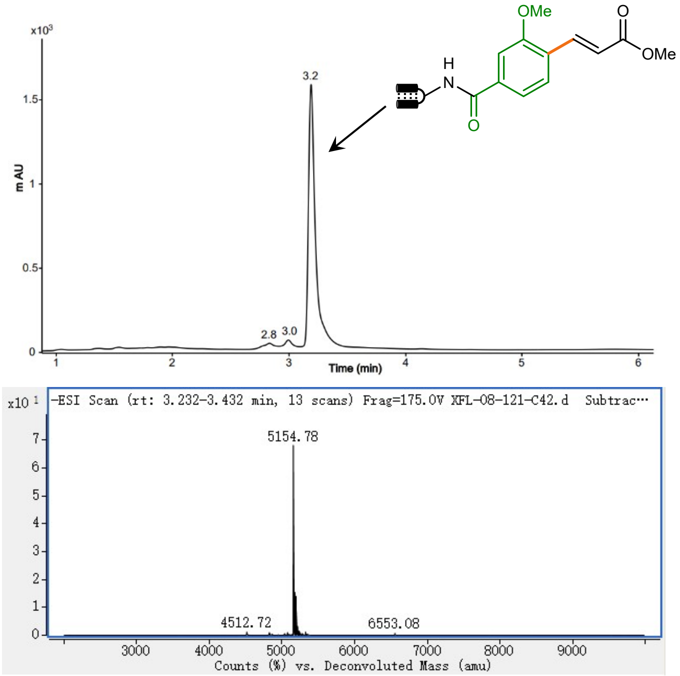

UPLC chromatograph and deconvoluted MS of **5ci.**

Conversion: 89%

Calculated Mass: 5183 Da; Found Mass: 5184 Da

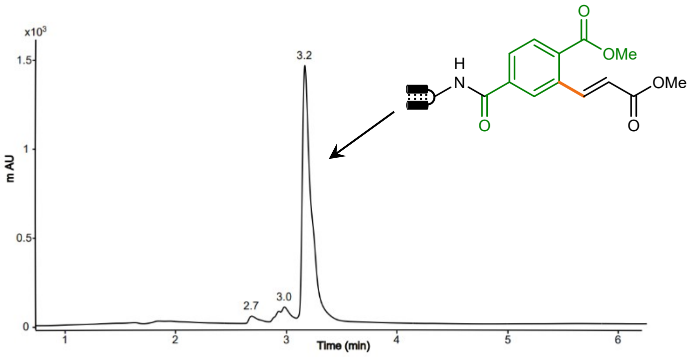

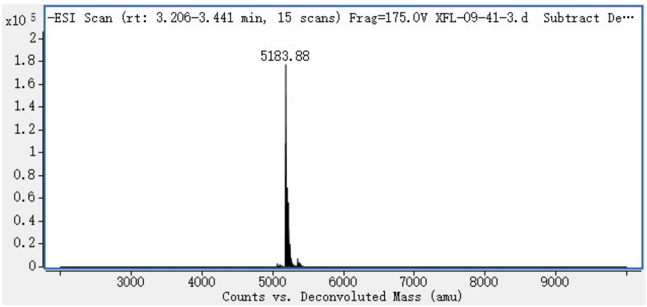

UPLC chromatograph and deconvoluted MS of **5cj.**

Conversion: 95%

Calculated Mass: 5201 Da; Found Mass: 5201 Da

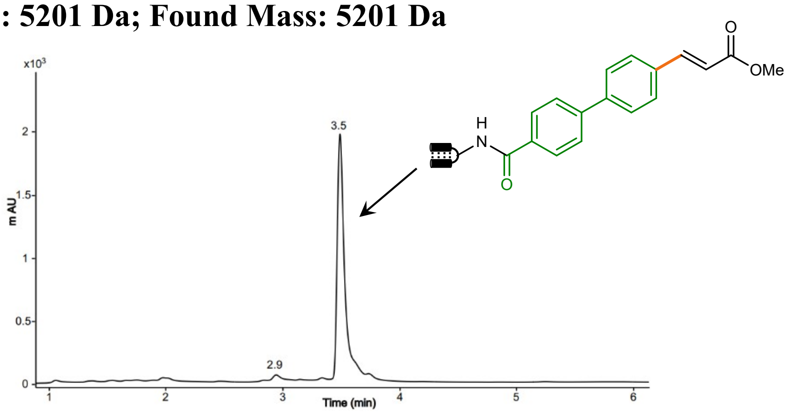

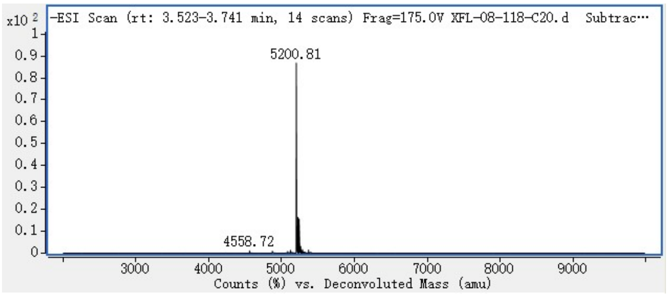

UPLC chromatograph and deconvoluted MS of **5ck.**

Conversion: 87%

Calculated Mass: 5175 Da; Found Mass: 5175 Da

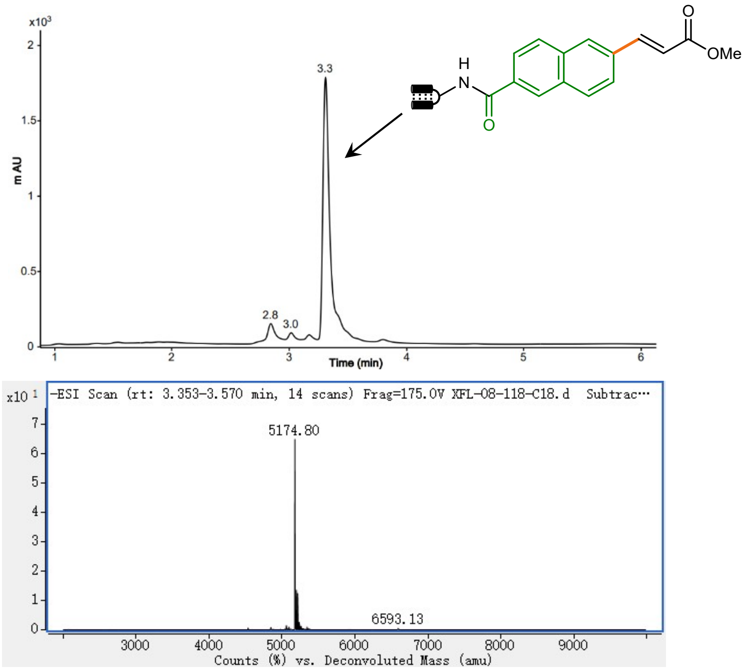

UPLC chromatograph and deconvoluted MS of **5cl.**

Conversion: 86%

Calculated Mass: 5161 Da; Found Mass: 5161 Da

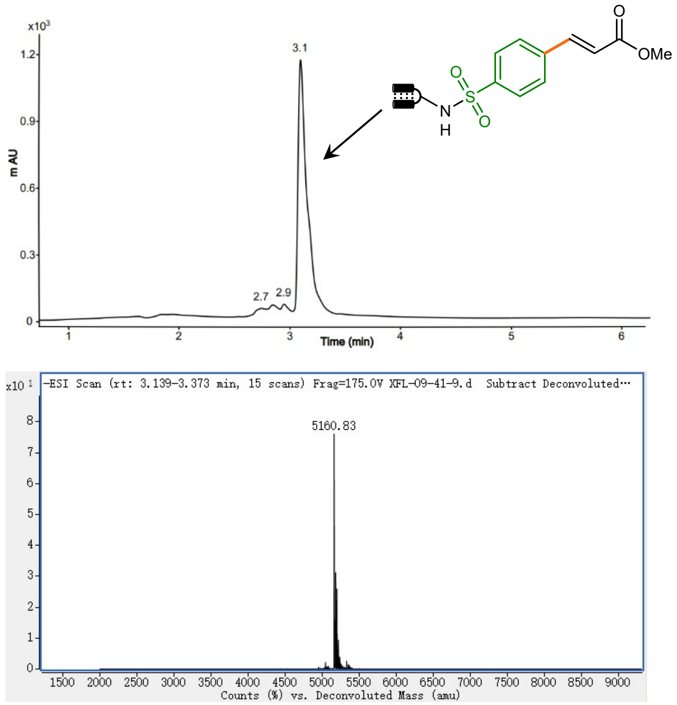

UPLC chromatograph and deconvoluted MS of **5cm.**

Conversion: 86%

Calculated Mass: 5143 Da; Found Mass: 5143 Da

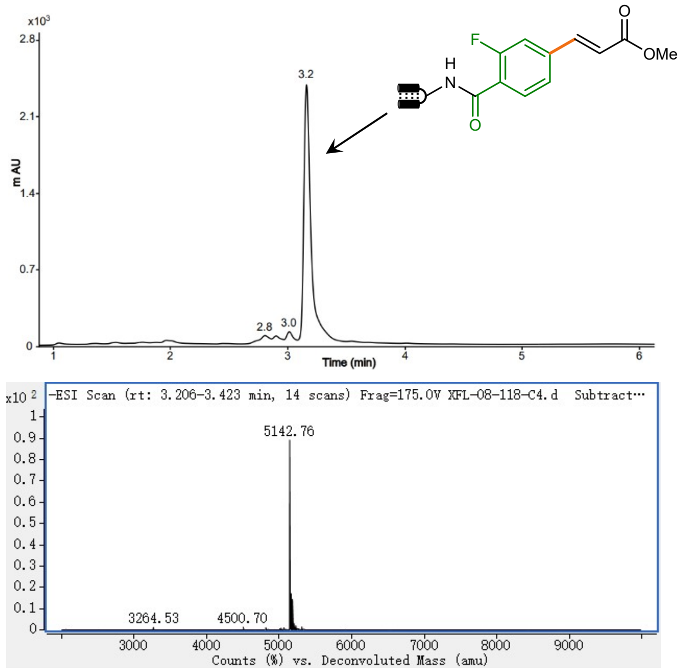

UPLC chromatograph and deconvoluted MS of **5cn.**

Conversion: 88%

Calculated Mass: 5157 Da; Found Mass: 5157 Da

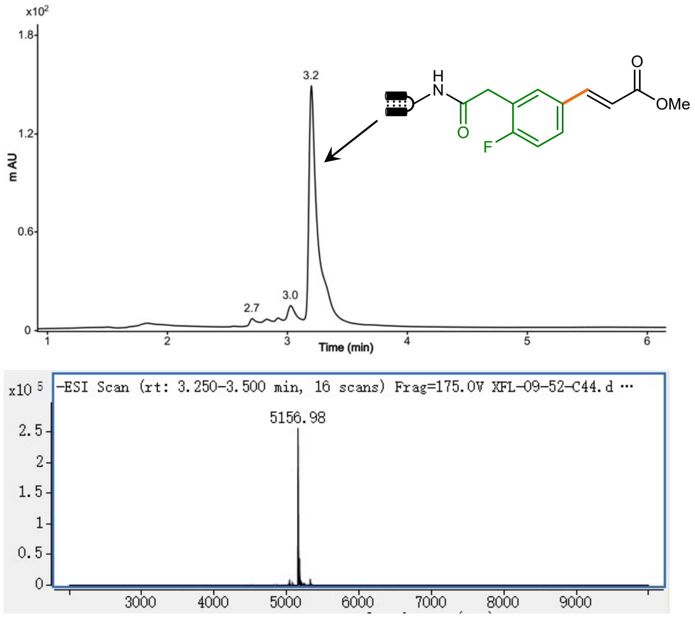

UPLC chromatograph and deconvoluted MS of **5co.**

Conversion: 93%

Calculated Mass: 5153 Da; Found Mass: 5153 Da

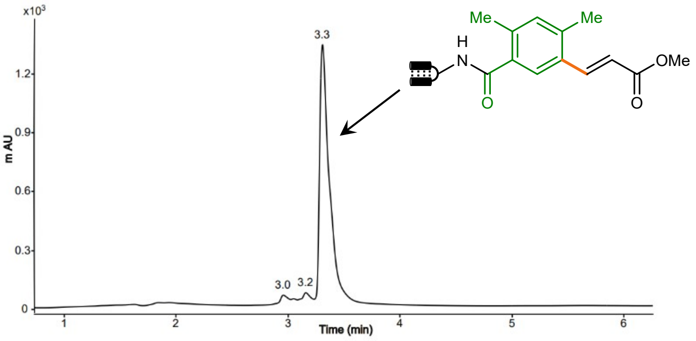

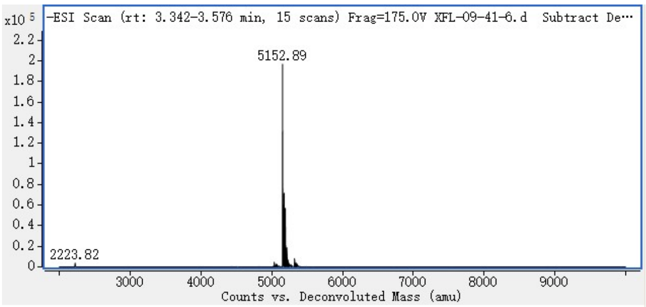

UPLC chromatograph and deconvoluted MS of **5cp.**

Conversion: 88%

Calculated Mass: 5157 Da; Found Mass: 5157 Da

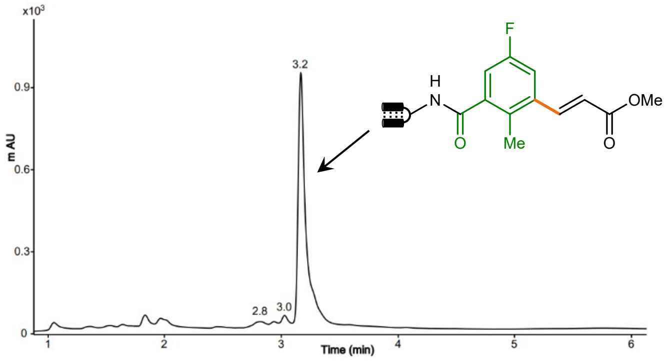

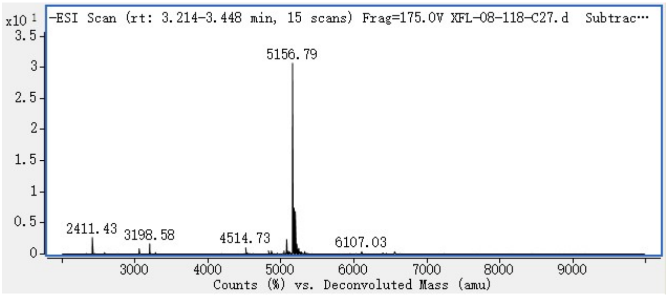

UPLC chromatograph and deconvoluted MS of **5cq.**

Conversion: 94%

Calculated Mass: 5155 Da; Found Mass: 5155 Da

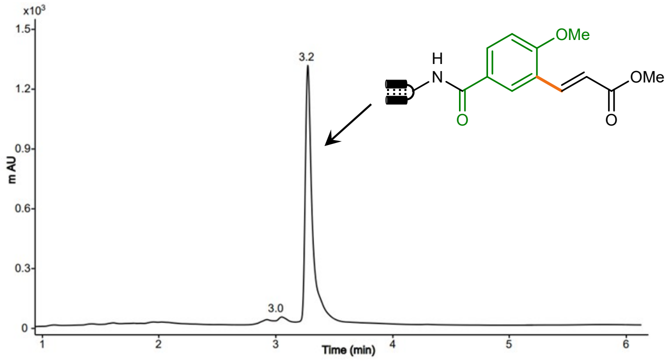

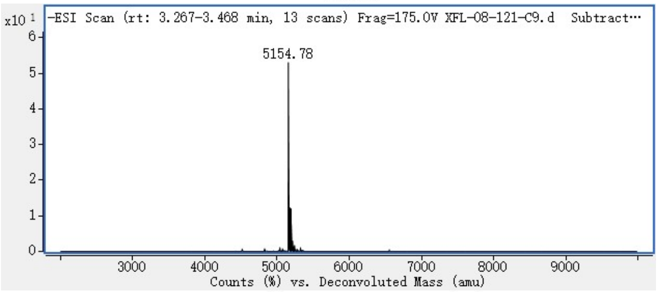

UPLC chromatograph and deconvoluted MS of **5cr.**

Conversion: 75%

Calculated Mass: 5143 Da; Found Mass: 5143 Da

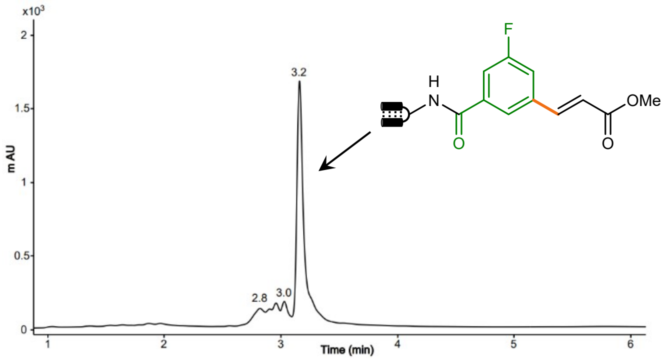

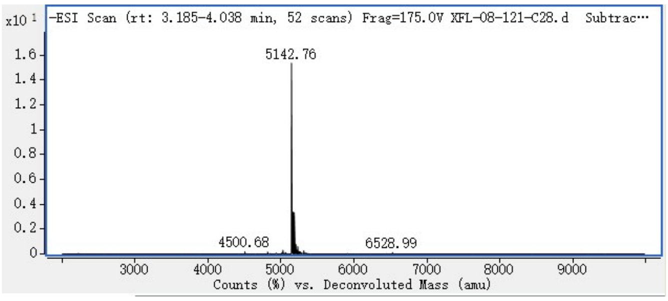

UPLC chromatograph and deconvoluted MS of **5cs.**

Conversion: 85%

Calculated Mass: 5157 Da; Found Mass: 5157 Da

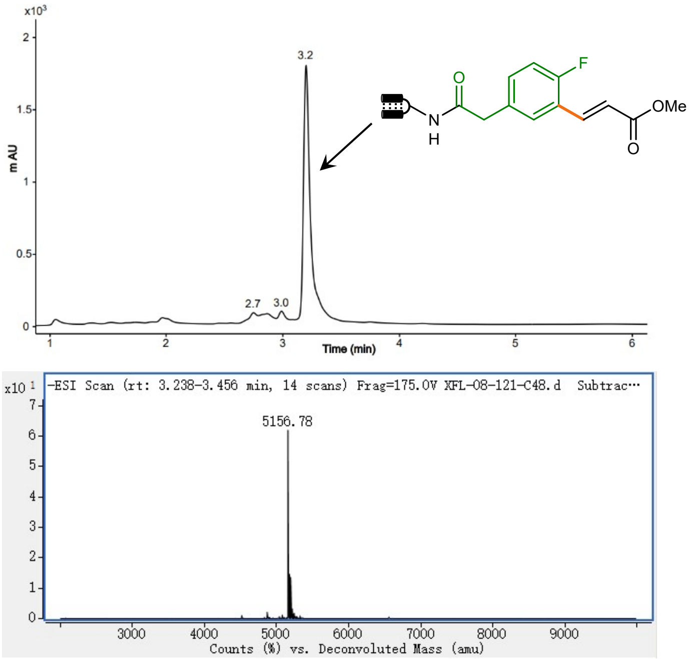

UPLC chromatograph and deconvoluted MS of **5ct.**

Conversion: 87%

Calculated Mass: 5155 Da; Found Mass: 5155 Da

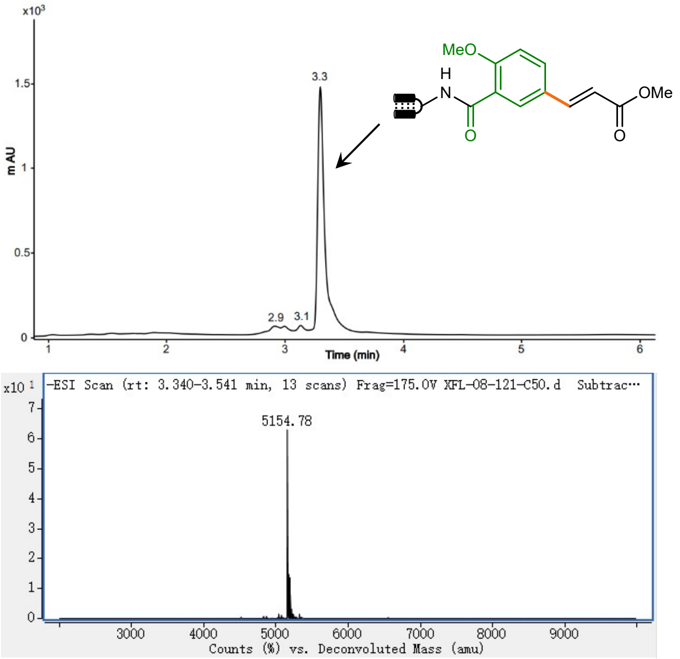

UPLC chromatograph and deconvoluted MS of **5cu.**

Conversion: 75%

Calculated Mass: 5143 Da; Found Mass: 5143 Da

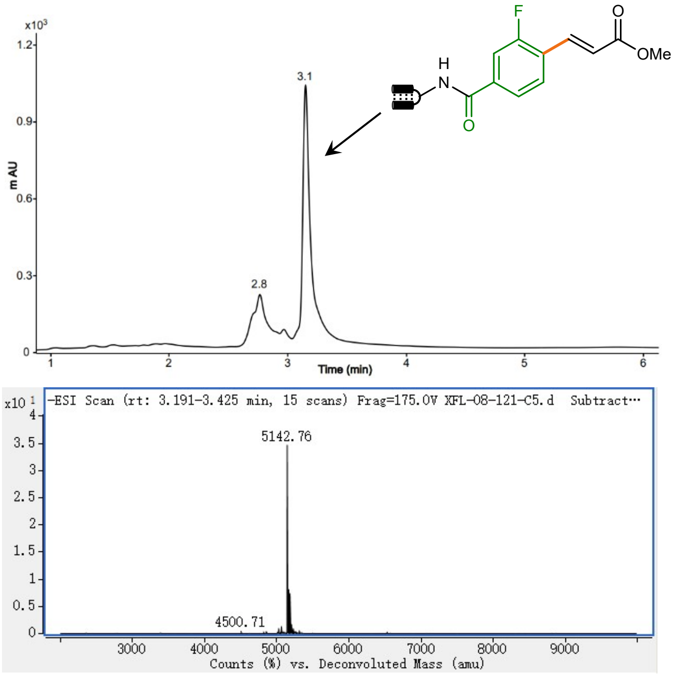

UPLC chromatograph and deconvoluted MS of **5cv.**

Conversion: 92%

Calculated Mass: 5139 Da; Found Mass: 5139 Da

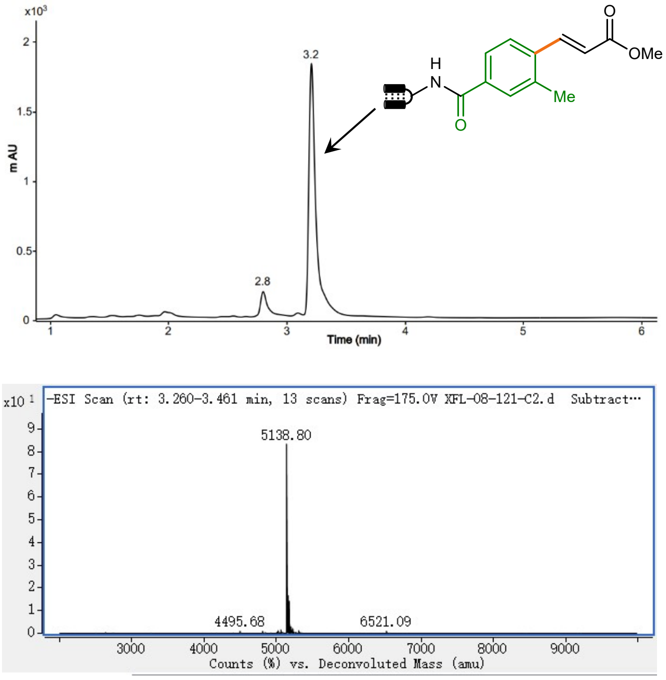

UPLC chromatograph and deconvoluted MS of **5cw.**

Conversion: 85%

Calculated Mass: 5139 Da; Found Mass: 5139 Da

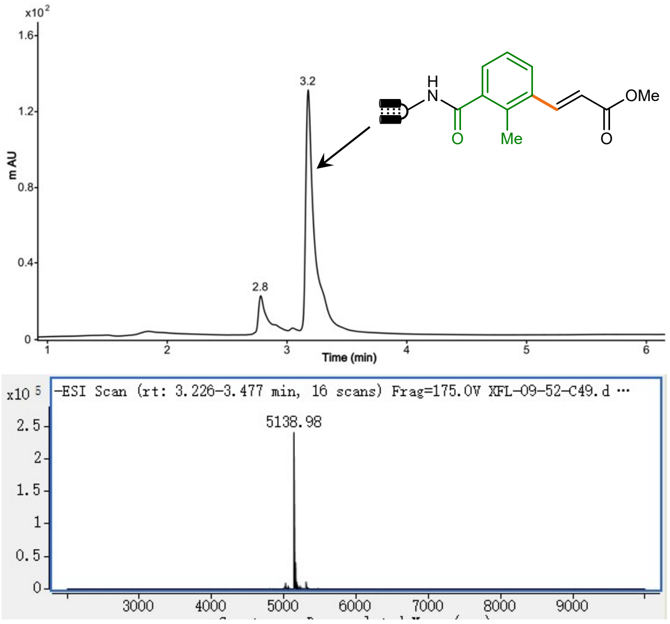

UPLC chromatograph and deconvoluted MS of **5cx.**

Conversion: 91%

Calculated Mass: 5204 Da; Found Mass: 5204 Da

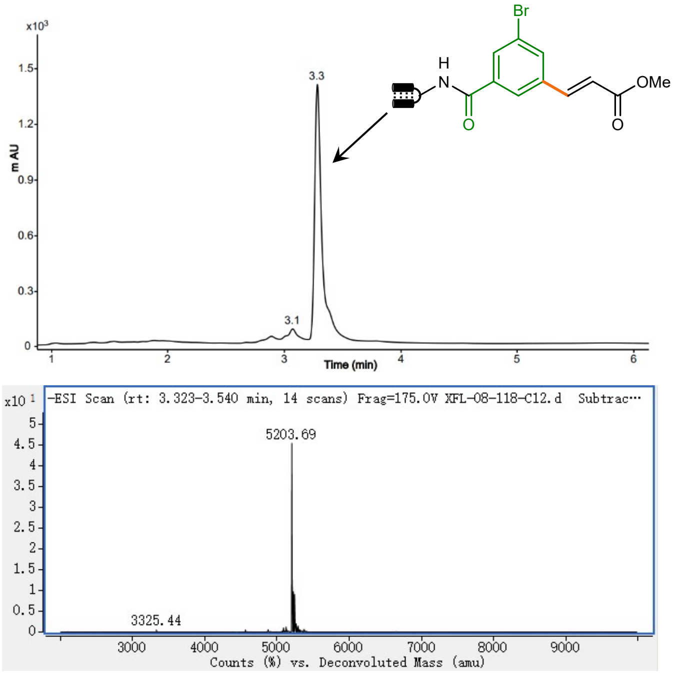

UPLC chromatograph and deconvoluted MS of **5cy.**

Conversion: 50%

Calculated Mass: 5164 Da; Found Mass: 5164 Da

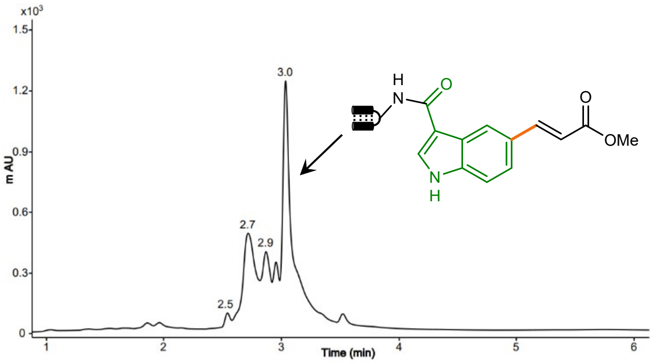

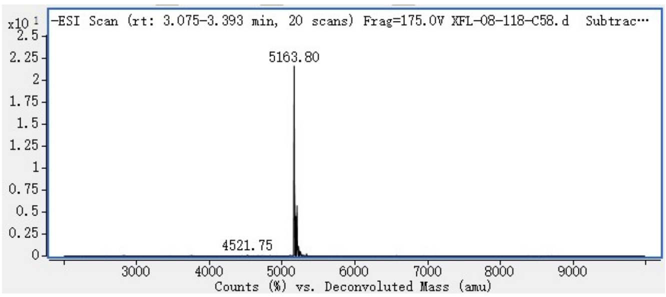

UPLC chromatograph and deconvoluted MS of **5cz.**

Conversion: 75%

Calculated Mass: 5181 Da; Found Mass: 5181 Da

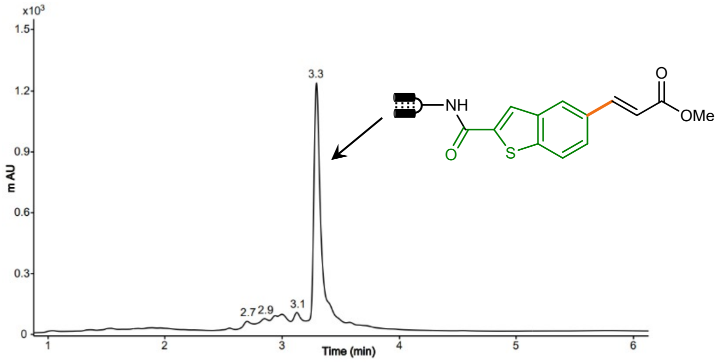

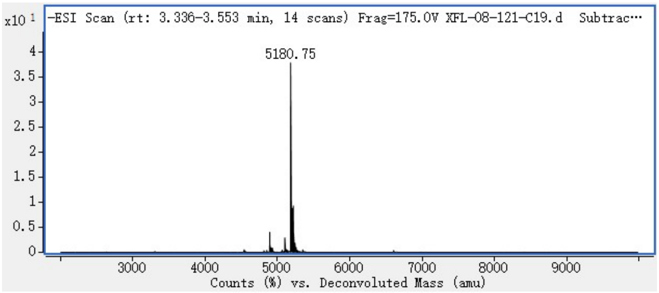

UPLC chromatograph and deconvoluted MS of **5da.**

Conversion: 79%

Calculated Mass: 5169 Da; Found Mass: 5169 Da

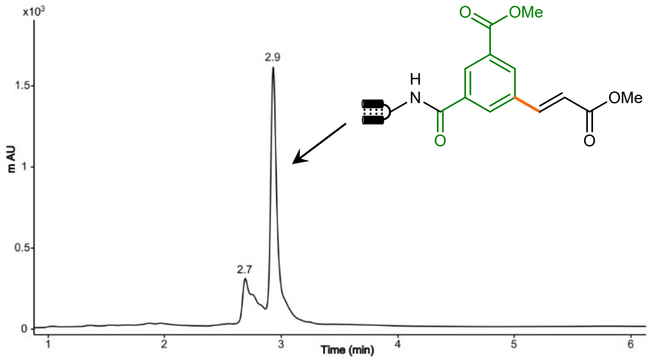

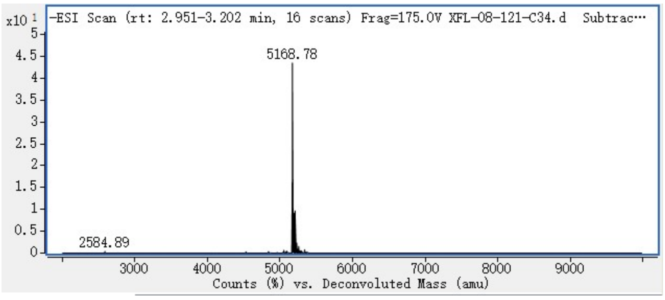

UPLC chromatograph and deconvoluted MS of **5db.**

Conversion: 89%

Calculated Mass: 5160 Da; Found Mass: 5160 Da

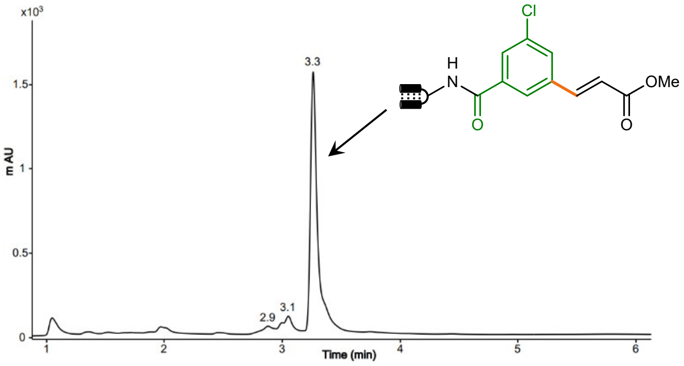

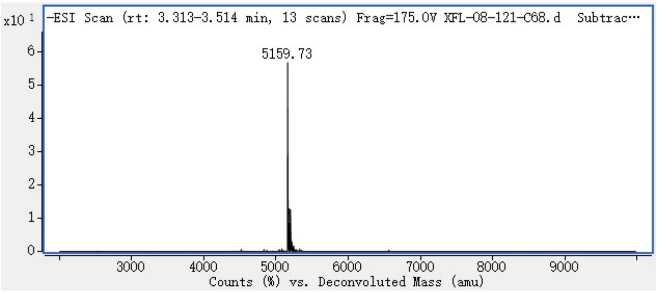

UPLC chromatograph and deconvoluted MS of **5dc.**

Conversion: 90%

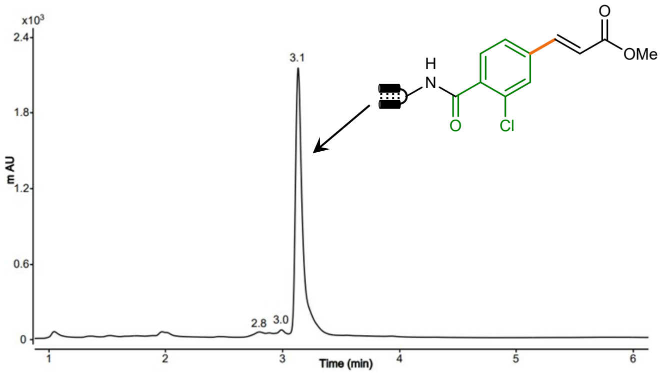

Calculated Mass: 5160 Da; Found Mass: 5160 Da

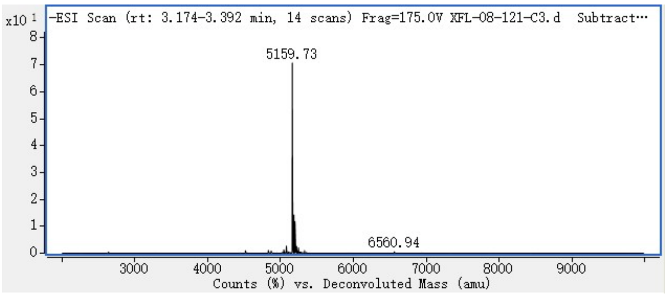

UPLC chromatograph and deconvoluted MS of **5dd**

Conversion: 92%

Calculated Mass: 5160 Da; Found Mass: 5160 Da

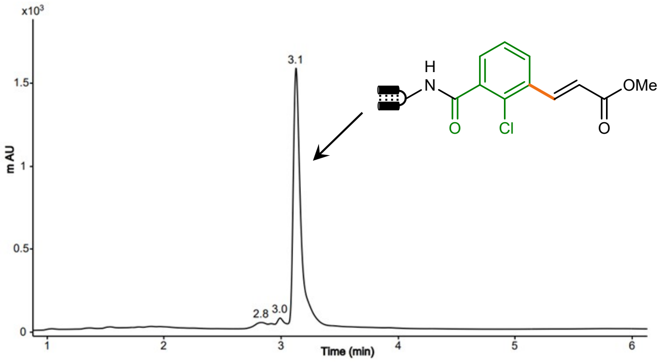

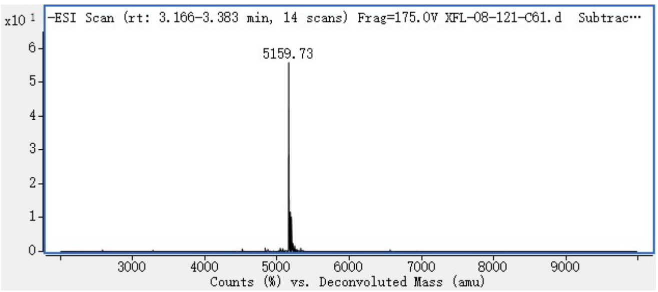

UPLC chromatograph and deconvoluted MS of **5de.**

Conversion: 90%

Calculated Mass: 5160 Da; Found Mass: 5160 Da

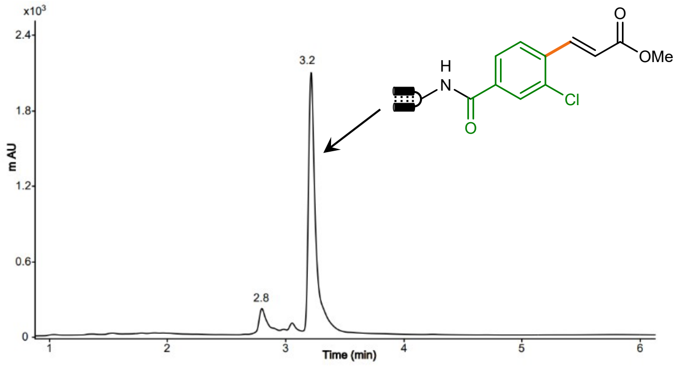

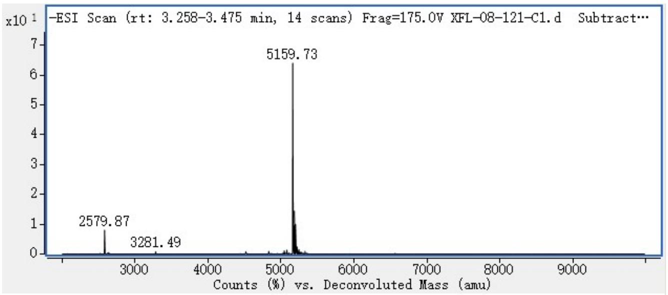

UPLC chromatograph and deconvoluted MS of **5df.**

Conversion: 63%

Calculated Mass: 5139 Da; Found Mass: 5139 Da

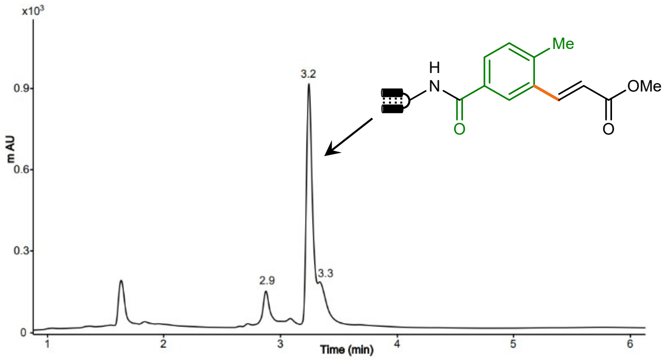

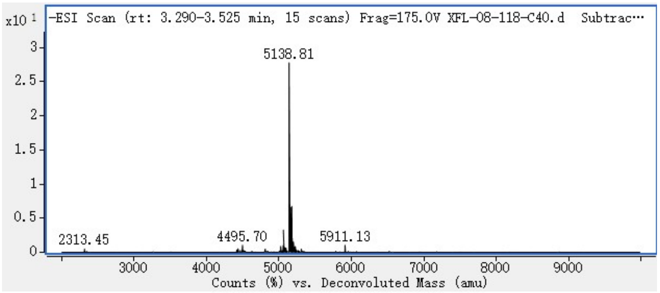

UPLC chromatograph and deconvoluted MS of **5dg.**

Conversion: 73%

Calculated Mass: 5193 Da; Found Mass: 5194 Da

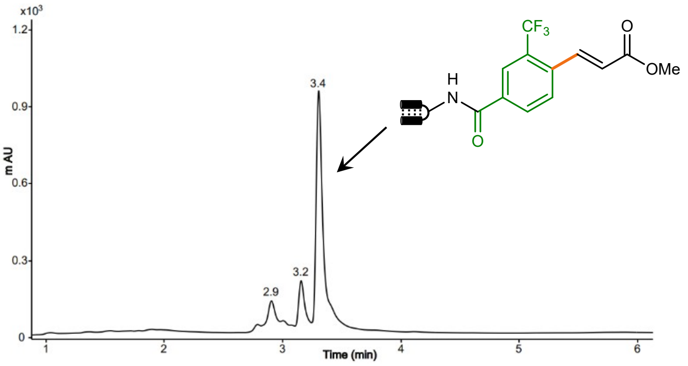

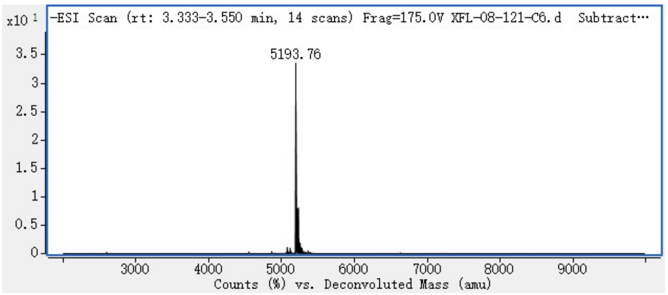

UPLC chromatograph and deconvoluted MS of **5dh.**

Conversion: 92%

Calculated Mass: 5139 Da; Found Mass: 5139 Da

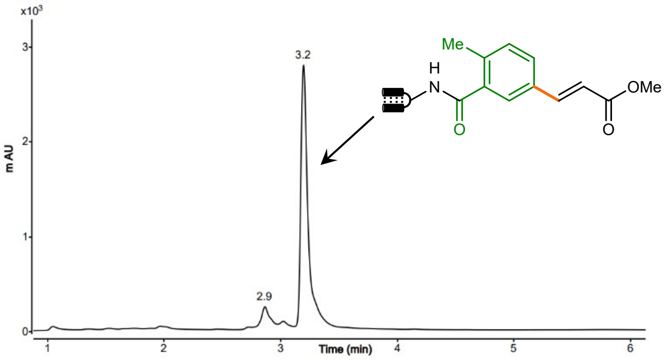

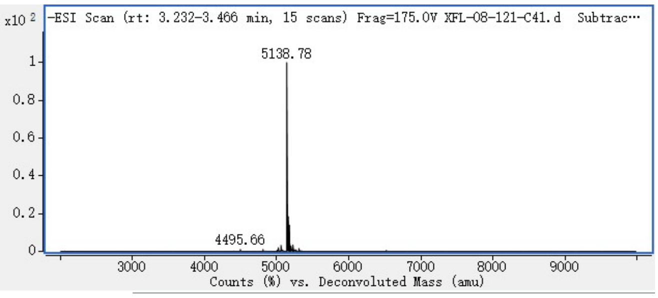

UPLC chromatograph and deconvoluted MS of **5di.**

Conversion: 67%

Calculated Mass: 5153 Da; Found Mass: 5153 Da

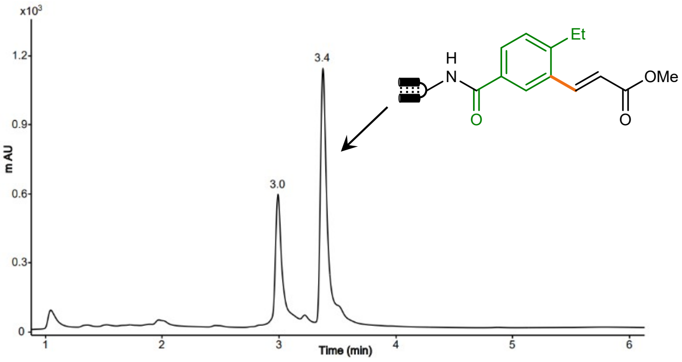

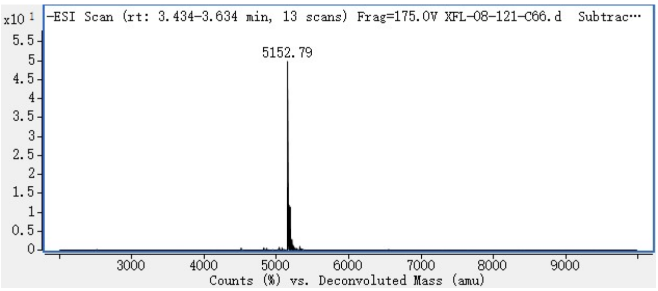

UPLC chromatograph and deconvoluted MS of **5dj.**

Conversion: 76%

Calculated Mass: 5170 Da; Found Mass: 5170 Da

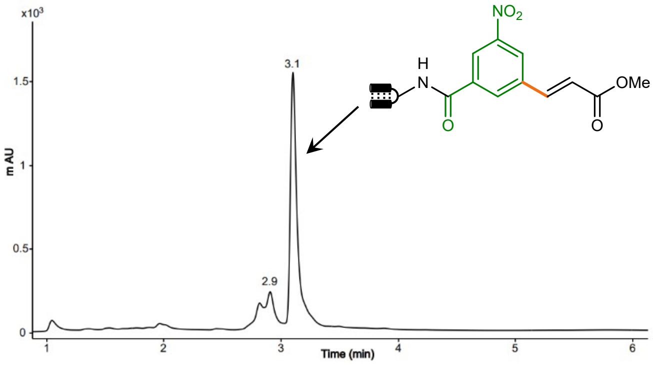

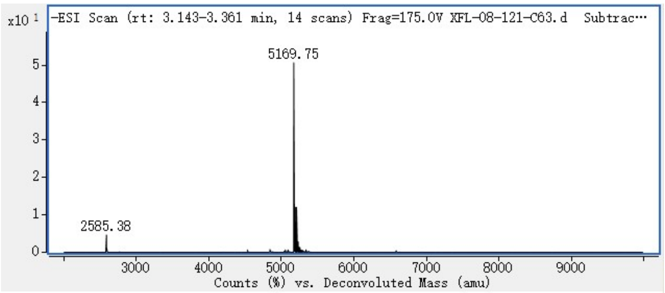

x10 1
-ESI Scan (rt: 3.143-3.361 min, 14 scans) Frag=175.0V XFL-08-121-C63.d Subtrac…
5169.75
2585.38
5
4
3
2
1
0
3000
4000
5000
6000
7000
8000
9000
Counts (%) vs. Deconvoluted Mass (amu)

8.8 Substrate scope of anilines for the reverse on-DNA Heck reaction with 6a

UPLC chromatograph and deconvoluted MS of **5a.**

Conversion: 80%

Calculated Mass: 5143 Da; Found Mass: 5143 Da

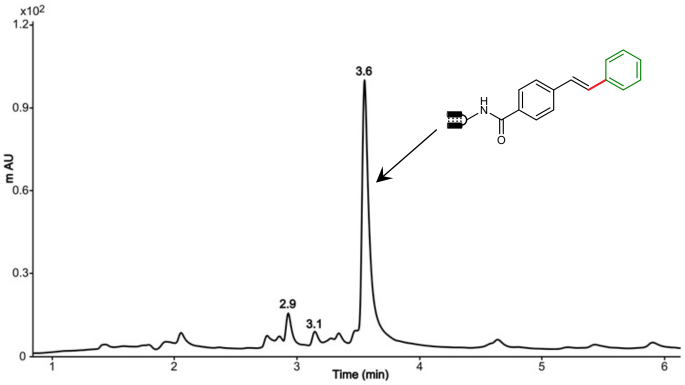

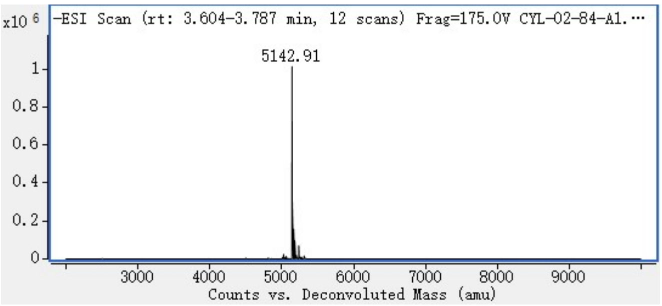

UPLC chromatograph and deconvoluted MS of **5c.**

Conversion: 77%

Calculated Mass: 5173 Da; Found Mass: 5173 Da

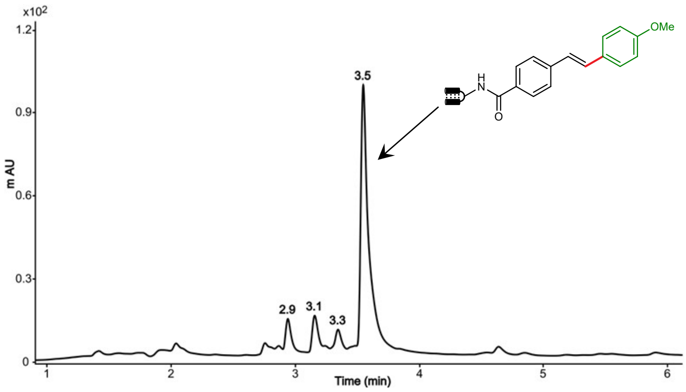

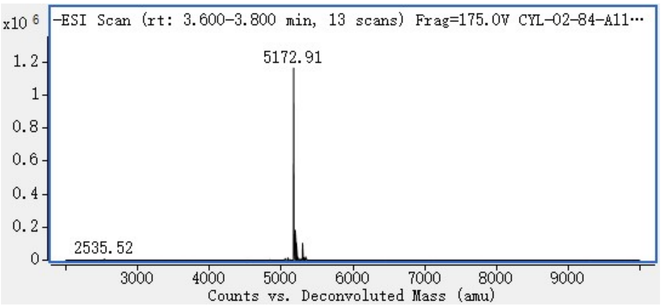

UPLC chromatograph and deconvoluted MS of **5g.**

Conversion: 89%

Calculated Mass: 5188 Da; Found Mass: 5188 Da

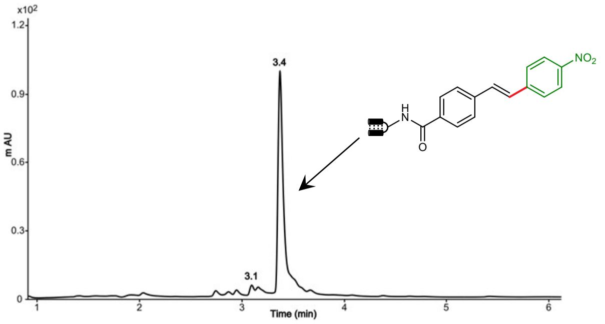

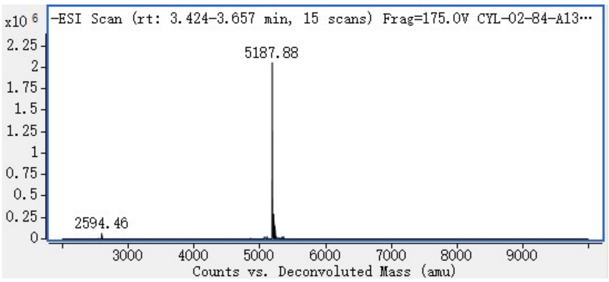

UPLC chromatograph and deconvoluted MS of **5m.**

Conversion: 57%

Calculated Mass: 5187 Da; Found Mass: 5187 Da

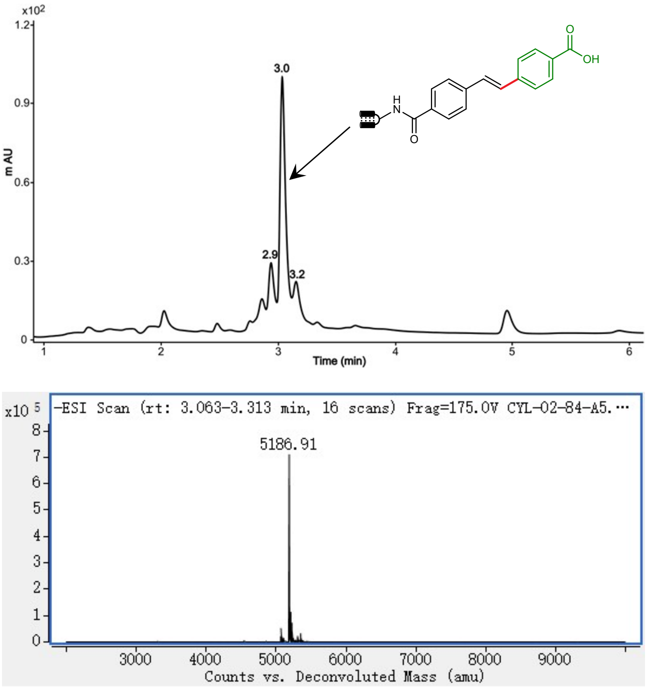

UPLC chromatograph and deconvoluted MS of **5k.**

Conversion: 55%

Calculated Mass: 5168 Da; Found Mass: 5168 Da

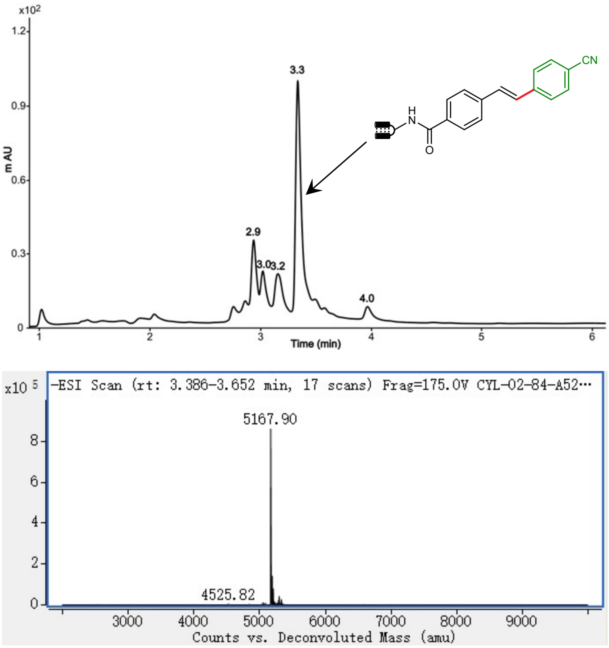

UPLC chromatograph and deconvoluted MS of **5o.**

Conversion: 85%

Calculated Mass: 5201 Da; Found Mass: 5201 Da

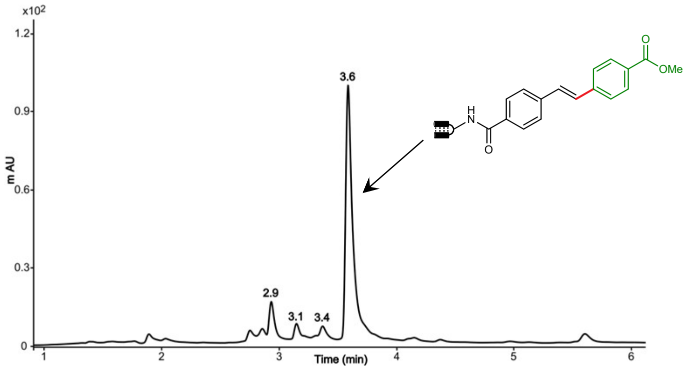

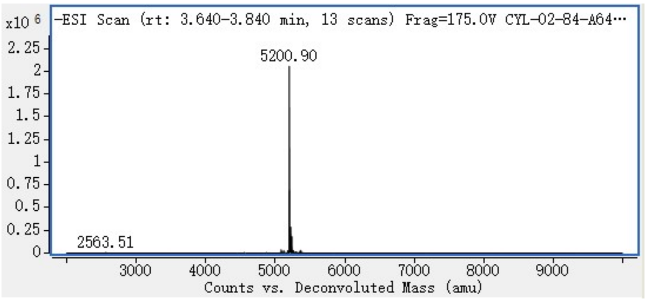

UPLC chromatograph and deconvoluted MS of **5aa.**

Conversion: 72%

Calculated Mass: 5177 Da; Found Mass: 5177 Da

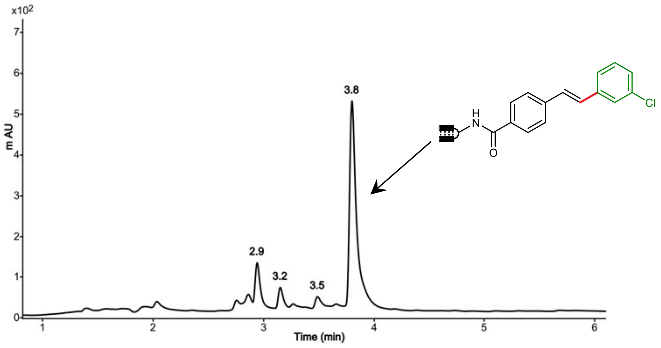

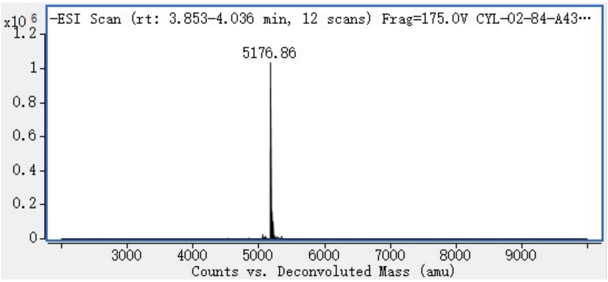

UPLC chromatograph and deconvoluted MS of **5di.**

Conversion: 70%

Calculated Mass: 5173 Da; Found Mass: 5173 Da

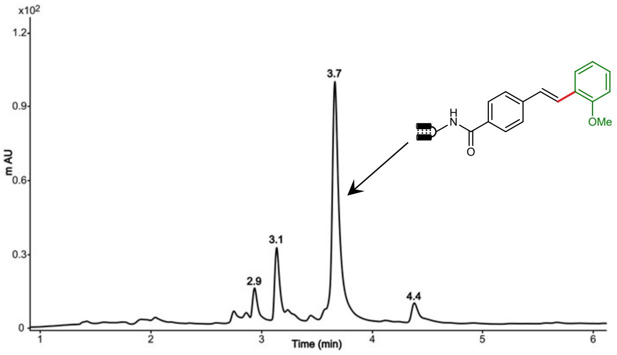

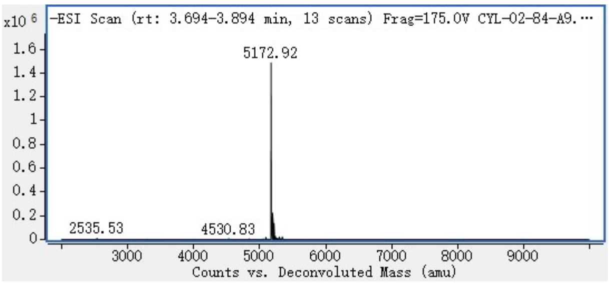

UPLC chromatograph and deconvoluted MS of **5dj.**

Conversion: 89%

Calculated Mass: 5211 Da; Found Mass: 5211 Da

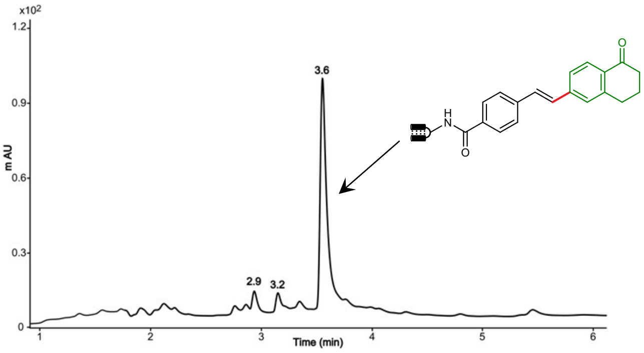

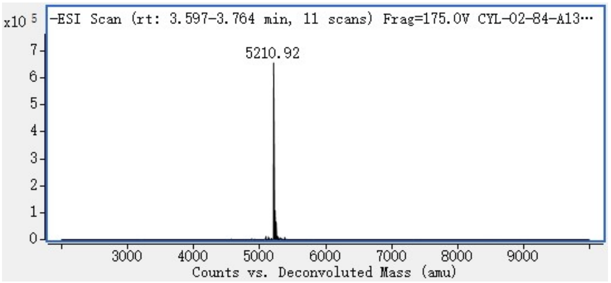

UPLC chromatograph and deconvoluted MS of **5dk.**

Conversion: 77%

Calculated Mass: 5221 Da; Found Mass: 5221 Da

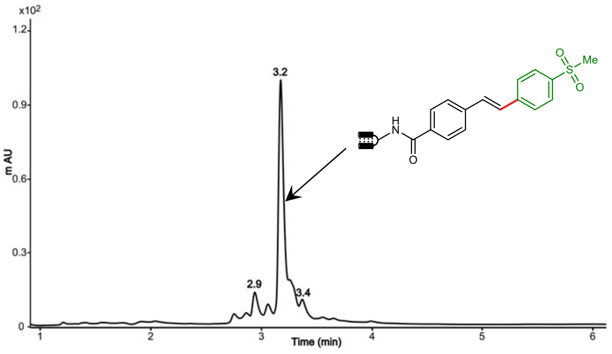

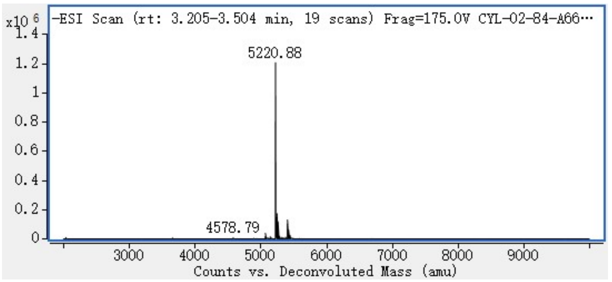

UPLC chromatograph and deconvoluted MS of **5dl.**

Conversion: 64%

Calculated Mass: 5211 Da; Found Mass: 5211 Da

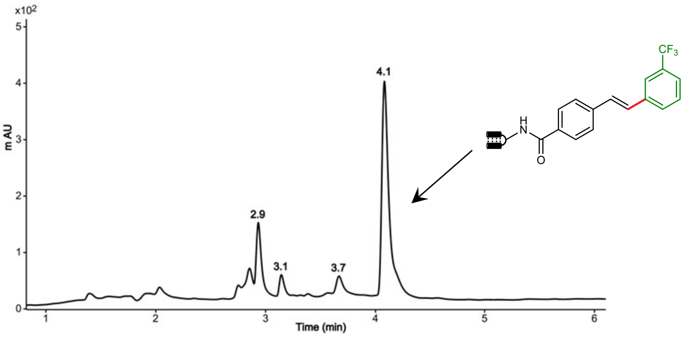

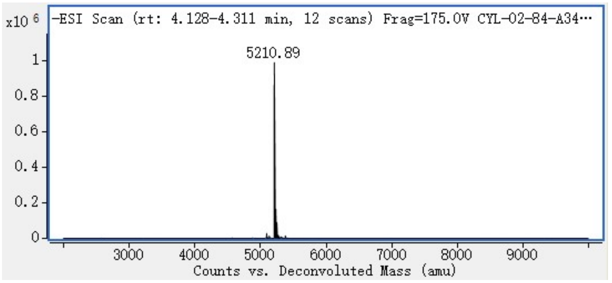

UPLC chromatograph and deconvoluted MS of **5dm.**

Conversion: 68%

Calculated Mass: 5211 Da; Found Mass: 5211 Da

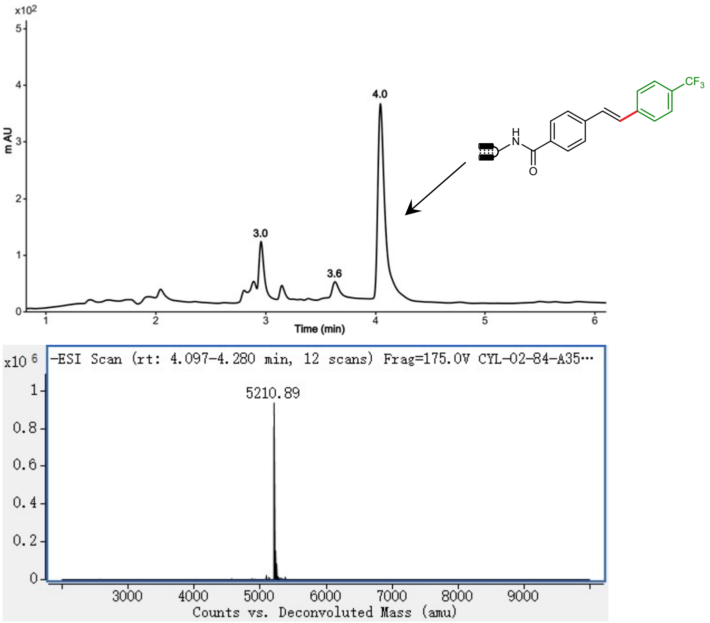

UPLC chromatograph and deconvoluted MS of **5dn.**

Conversion: 76%

Calculated Mass: 5161 Da; Found Mass: 5161 Da

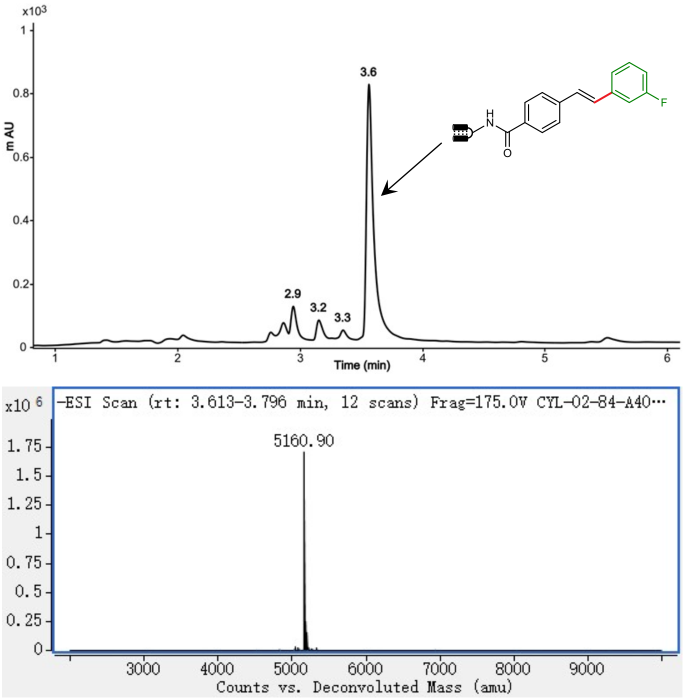

8.9 Substrate scope of DNA-conjugated arylamines for the On-DNA Hiyama coupling

UPLC chromatograph and deconvoluted MS of **10a.**

Conversion: 67%

Calculated Mass: 5117 Da; Found Mass: 5117 Da

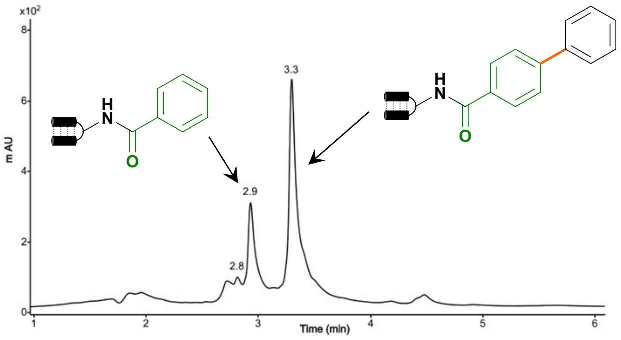

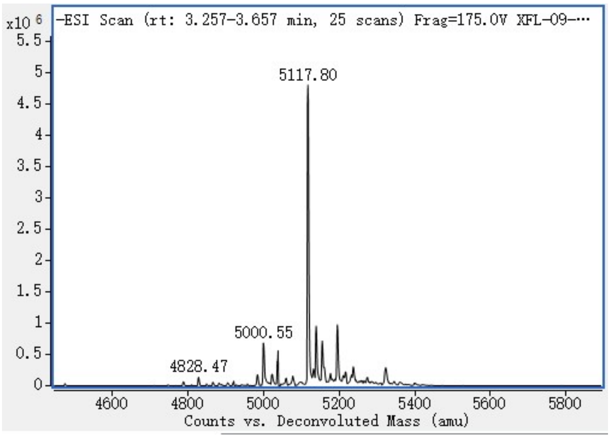

UPLC chromatograph and deconvoluted MS of **10b.**

Conversion: 61%

Calculated Mass: 5117 Da; Found Mass: 5117 Da

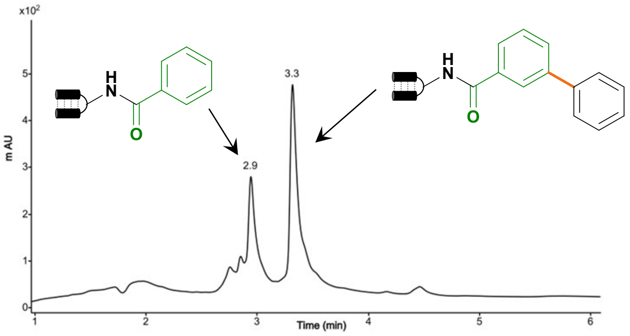

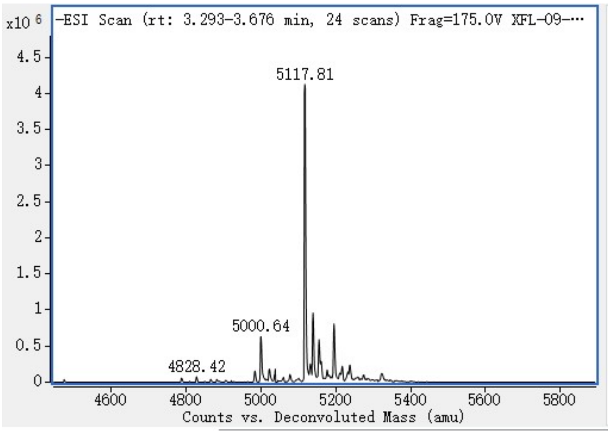

UPLC chromatograph and deconvoluted MS of **10c.**

Conversion: 66%

Calculated Mass: 5131 Da; Found Mass: 5131 Da

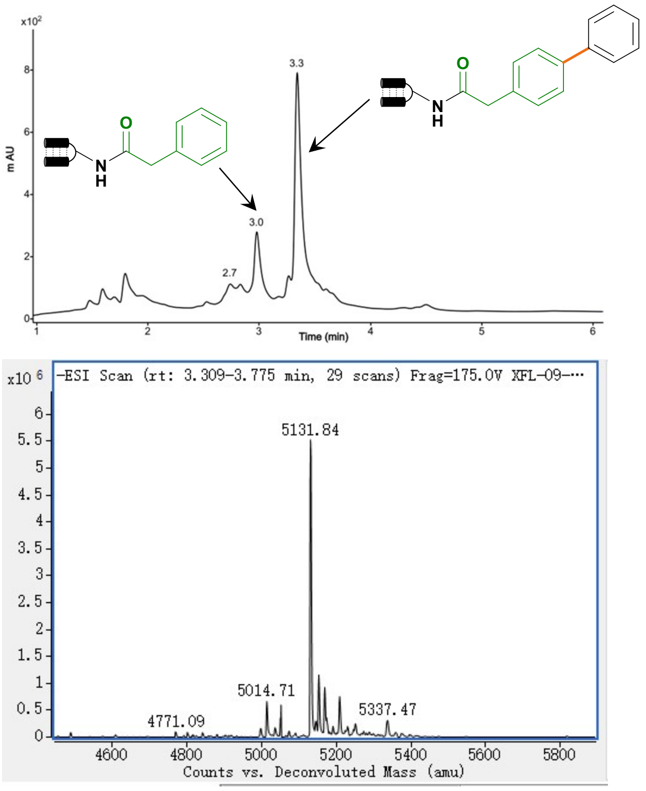

UPLC chromatograph and deconvoluted MS of **10d.**

Conversion: 63%

Calculated Mass: 5149 Da; Found Mass: 5149 Da

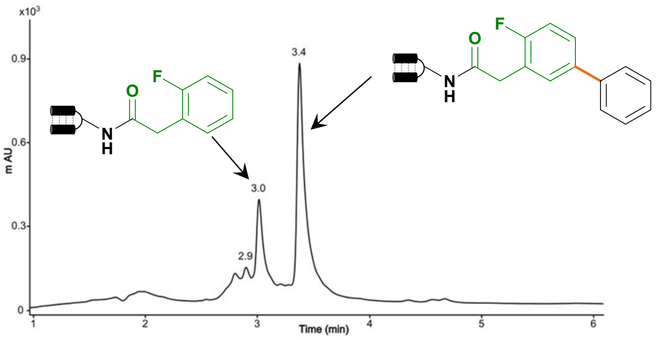

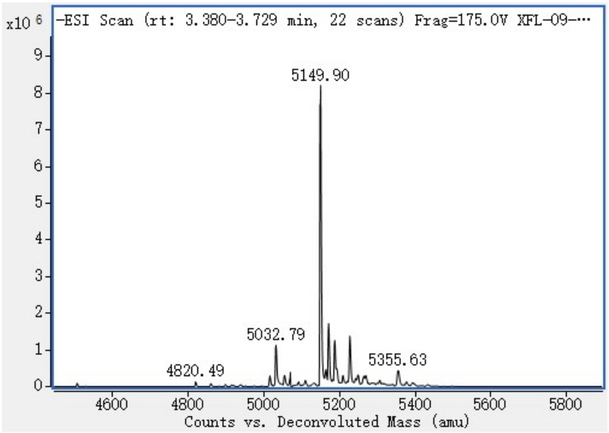

UPLC chromatograph and deconvoluted MS of **10e.**

Conversion: 57%

Calculated Mass: 5173 Da; Found Mass: 5173 Da

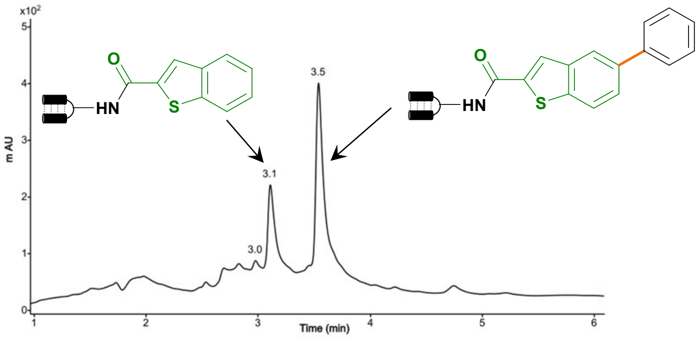

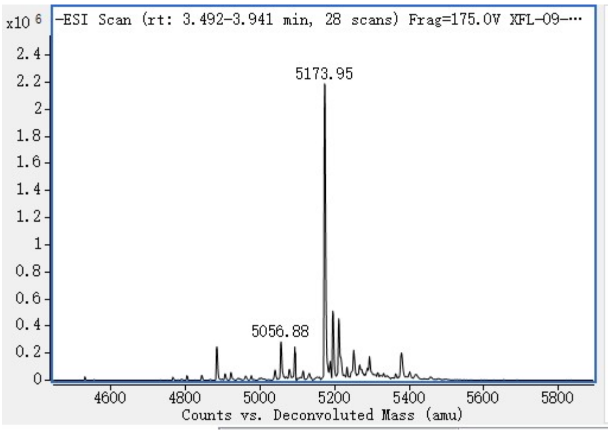

UPLC chromatograph and deconvoluted MS of **10f.**

Conversion: 51%

Calculated Mass: 5147 Da; Found Mass: 5147 Da

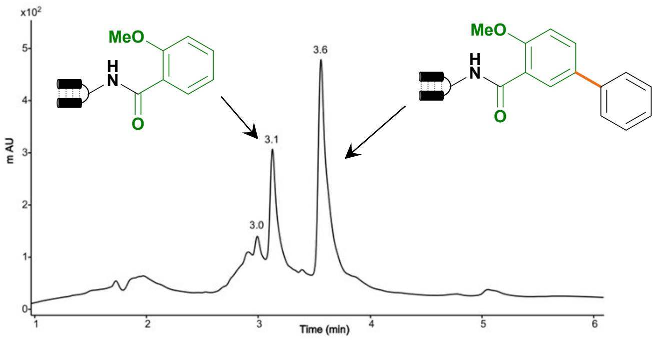

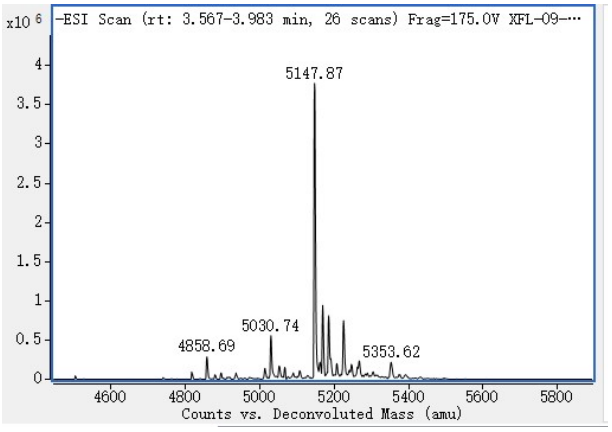

9. Reference

1. P. Yang, X. Wang, B. Li, Y. Yang, J. Yue, Y. Suo, H. Tong, G. He, X. Lu, G. Chen, *Chem. Sci*. 2021, **12**, 5804-5810.
2. A. Harada, *J. Am. Chem. Soc.* 2008, ***130***.
3. Y. Ding, J. L. DeLorey, M. A. Clark, *Bioconjugate Chem.* 2016, ***27***, 2597-2600.
4. X. Wang, H. Sun, J. Liu, W. Zhong, M. Zhang, H. Zhou, D. Dai, X. Lu, *Org. Lett.* 2019, ***21***, 719-723.
5. N. Ohtsuka, M. Okuno, Y. Hoshino, K. Honda, *Org. Biomol. Chem.* 2016, ***14***, 9046-9054.
6. N. L. Villadsen, K. M. Jacobsen, U. B. Keiding, E. T. Weibel, B. Christiansen, T. Vosegaard, M. Bjerring, F. Jensen, M. Johannsen, T. Tørring, T. B. Poulsen, *Nat. Chem.* 2016, ***9***, 264-272.
7. J. Zhang, J. Peng, Y. Huang, L. Meng, Q. Li, F. Xiong, X. Li, *Angew. Chem. Int. Ed.* 2020, ***59***, 17525-17532.
8. G. Li, Y. Liu, Y. Liu, L. Chen, S. Wu, Y. Liu, X. Li, *Angew. Chem. Int. Ed.* 2013, ***52***, 9544-9549.
